# From *De Novo* Conceived Small Molecules to Multifunctional Supramolecular Nanoparticles: Dual Biofilm and T3SS Intervention, Enhanced Foliar Affinity, and Effective Rice Disease Control

**DOI:** 10.1002/advs.202410878

**Published:** 2025-03-27

**Authors:** Xianfu Mu, Kongjun Liu, Jinghan Yang, Juan Liu, Fengpei Du, Gefei Hao, Peiyi Wang

**Affiliations:** ^1^ State Key Laboratory of Green Pesticide Key Laboratory of Green Pesticide and Agricultural Bioengineering Ministry of Education Center for Research and Development of Fine Chemicals of Guizhou University Guiyang 550025 China; ^2^ Key Laboratory of Basic Pharmacology of Ministry of Education and Joint International Research Laboratory of Ethnomedicine of Ministry of Education Zunyi Medical University Zunyi 563006 China; ^3^ Department of Applied Chemistry College of Science China Agricultural University Beijing 100193 China

**Keywords:** biofilms and T3SS, crop protection, dufulin analogues, foliar affinity, supramolecular chemistry

## Abstract

Conventional antimicrobials typically exhibit suboptimal deposition on rice leaves, resulting in poor efficacy, further impaired by biofilms and Type III Secretion Systems (T3SS). Herein, this study presents a supramolecular strategy to fabricate BtP27@*β*‐CD, a sunflower‐like material engineered through host‐guest recognition between *de novo* designed molecule BtP27 and *β*‐cyclodextrin. BtP27@*β*‐CD manifests enhanced foliar affinity and in vivo efficiency, demonstrating superior protective (62.67%) and curative (51.16%) activities against bacterial leaf blight at a low‐dose of 200 µg mL^−1^ compared to commercial thiodiazole‐copper (37.78%/38.13%) without compromising safety. This multifunctional material, structurally derived from dufulin, inherit progenitor's systemic and conductive properties, alongside the capacity to activate salicylic acid‐mediated plant defense pathways. Moreover, it is endowed with the anticipated abilities to disorganize biofilm barriers, annihilate encased pathogens, and inhibit T3SS. This constitutes the inaugural report of a supramolecular‐based biofilm/T3SS dual inhibitor. An expanded investigation into substrate and indication screening identified additional molecules that self‐assemble with *β*‐cyclodextrin to form supramolecular materials, exhibiting superior potency against other rice diseases, with protective potency ranging from 63.53% to 73.30% and curative efficacy spanning 42.18% to 60.41% at 200 µg mL^−1^. In brief, this work establishes a paradigm for designing guest molecules from scratch to construct supramolecular materials with tailored characteristics.

## Graphical abstract

From *de novo* engineered bioactive molecules BtP27 to the multifunctional supramolecular material BtP27@*β*‐CD with a sunflower‐shaped morphology: its pragmatic applications in augmenting foliar affinity, disrupting biofilms and T3SS, and managing rice pathologies.

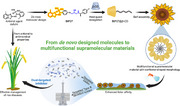

## 1 Introduction

As the staple cereal crop, *Oryza sativa* serves as the caloric foundation for ≈67% of the global population.^[^ ^1^, ^2^ ^]^ However, its susceptibility to bacterial (leaf blight, leaf streak) and fungal (sheath blight, blast) diseases poses significant threats to global food security.^[^ ^3^, ^4^, ^5^, ^6^ ^]^ Despite advances in biopesticides and resistant cultivars, chemical control remains essential.^[^ ^7^ ^]^ Nonetheless, the superhydrophobic rice leaf surface from waxy cuticles causes ineffective spraying through droplet rebound, reducing pesticide uptake and translocation.^[^ ^8^, ^9^ ^]^ Consequently, the actual utilization by the target organism is suboptimal, with runoff generating environmental and health hazards.^[^ ^10^ ^]^ Current strategies primarily focus on the use of additives and the development of nanocarriers. While enhancing pesticide adhesion, additives incur ecological trade‐offs.^[^ ^11^ ^]^ Meanwhile, nanocarriers face challenges in complex manufacturing processes and high costs.^[^ ^12^ ^]^ Therefore, neither strategy satisfactorily addresses multiple facets of pesticide application, necessitating the development of cost‐effective, eco‐friendly formulations with enhanced bioavailability.

Supramolecular chemistry presents a promising solution to address these challenges. Driven by non‐covalent forces, custom‐designed molecules self‐assemble in aqueous media, forming multifunctional supramolecular materials.^[^ ^13^, ^14^ ^]^ These assemblies streamline traditional formulation processes while reducing additive‐related environmental and health risks, thereby improving sustainability and eco‐friendliness.^[^ ^15^ ^]^ Moreover, supramolecular assemblies integrate host‐guest components, enabling functional diversification.^[^ ^16^ ^]^ This synergy creates emergent properties absent in individual building blocks while combining their complementary advantages. Cyclodextrin (*β*‐CD), a pivotal building block, represents an economically viable and environmentally benign cyclic oligosaccharide host.^[^ ^17^, ^18^ ^]^ Its structure combines a hydrophobic central cavity with hydrophilic hydroxyl groups at the portals, establishing spontaneous encapsulation of active compounds in aqueous environments.^[^ ^19^ ^]^ With regard to the guest molecules, however, current supramolecular systems predominantly utilize pre‐existing structures, preventing the artificial customization of molecular architectures and tailored characteristics.

In pursuit of a resolution to this issue, our endeavors are focused on the engineering of guest molecules with tailored mechanisms of action, specifically targeting biofilms and the Type III secretion system (T3SS), which are pivotal in orchestrating bacterial pathogenicity.^[^ ^20^ ^]^ As established virulence factors, biofilms protect bacterial communities from host defenses and bactericides.^[^ ^21^ ^]^ The well‐characterized T3SS mediates effector translocation into host cells, hijacking molecular mechanisms to enhance pathogen virulence. Emerging evidence reveals its pivotal role in biofilm formation on plant surfaces, indicating its importance in persistent colonization of pathogen.^[^ ^22^ ^]^ The combined action of biofilms and T3SS, along with the multifactorial nature of pathogenicity, challenges traditional antibacterial therapies, necessitating concurrent targeting strategies.^[^ ^23^ ^]^ Nevertheless, to the best of our knowledge, supramolecular‐based dual inhibitors capable of concurrently targeting both items remain unreported.

Herein, we initiated the rational design of functional guest molecules structurally derived from dufulin, a commercial antiviral agent celebrated for its systemic and conductive properties, as well as its ability to potentiate salicylic acid‐mediated plant defense pathways.^[^ ^24^, ^25^, ^26^ ^]^ The selection of dufulin as the foundational scaffold is primarily driven by its success in agricultural applications, underscoring its potential for agricultural utilization, with its inherent attributes particularly suited for this domain. Additionally, both the agricultural and pharmaceutical industries have increasingly repurposed established agents for new indications and mechanisms of action. It has become increasingly common for familiar core structures to appear across various agents.^[^ ^27^ ^]^ This trend is exemplified by the benzothiazole moiety in dufulin, which reappears in pesticides such as dichlobentiazox (bactericide) and methabenzthiazuron (herbicide), while the *α*‐aminophosphate group is increasingly found in agrochemicals, including a cinnam‐based biofilm inhibitor reported by the Ghazanfari group and a coumarin‐based biofilm inhibitor developed by the Zhou group (Figure S1, Supporting Information).^[^ ^27^, ^28^ ^]^ Despite these advantages, dufulin does not modulate biofilm or T3SS, both critical virulence factors in bacterial pathogenicity.

**Scheme 1:**
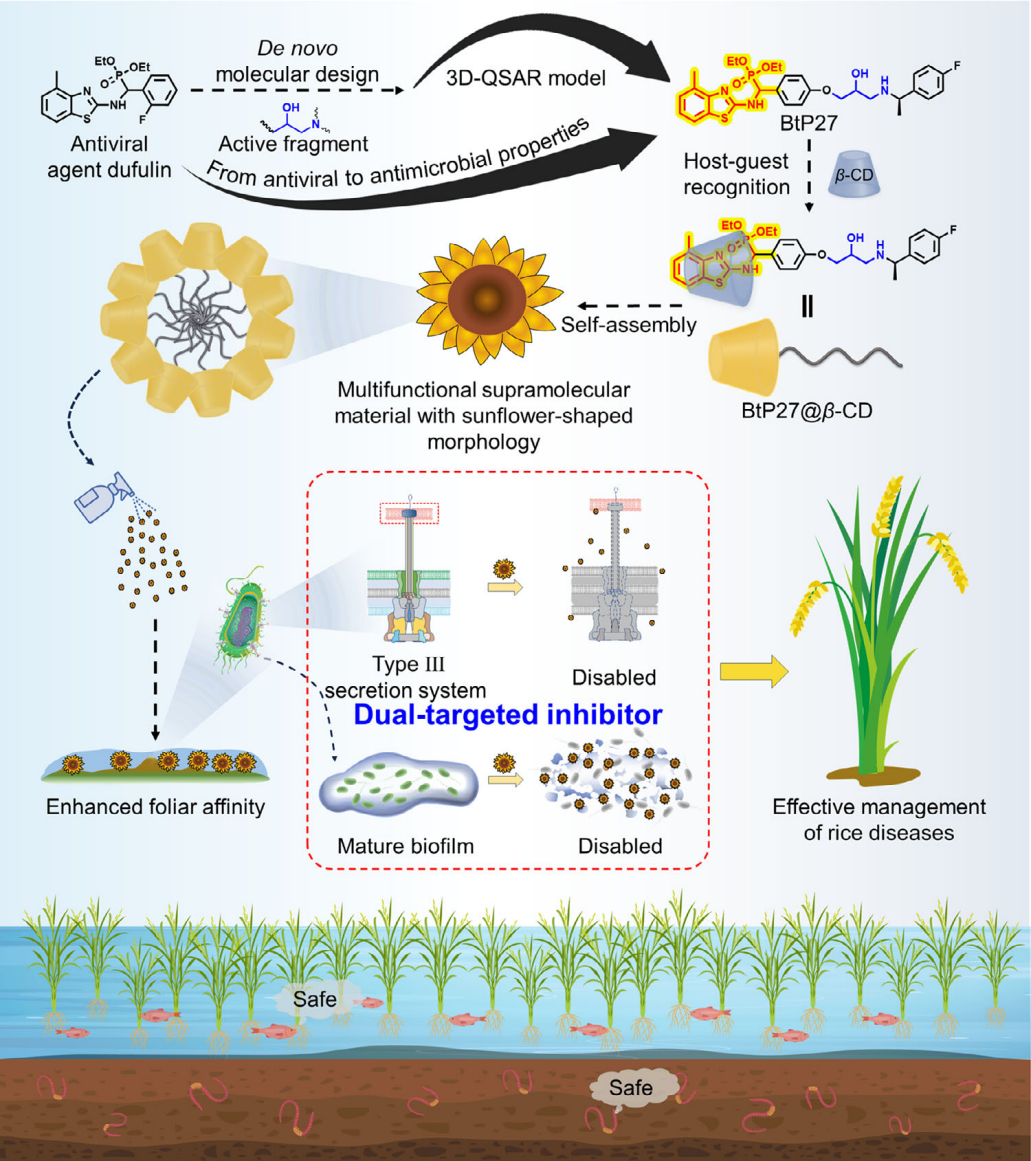
Schematic depiction of the preparation of potent multifunctional supramolecular bactericidal materials with dual biofilm and T3SS intervention, featuring superior foliar affinity for the effective management of rice diseases.

Our objective was to retain the intrinsic benefits of dufulin while imparting the capacity to target biofilm and T3SS. To this end, isopropanolamine, commonly encountered in biofilm and T3SS studies (see Figure S1, Supporting Information for details),^[^ ^29^, ^30^, ^31^, ^32^, ^33^, ^34^ ^]^ was conjugated to the dufulin core via a phenyl linker, resulting in a series of novel derivatives through a concise three‐step process. Among these derivatives, BtP27 exhibited the highest in vitro antibacterial potency against *Xanthomonas oryzae* pv. *oryzae* (*Xoo*), and was thus selected for self‐assembly with *β*‐CD to form the supramolecular complex, BtP27@*β*‐CD. This engineered complex emerges as a multifunctional material, characterized by its sunflower‐like morphology, which reveals enhanced foliar affinity and superior in vivo efficacy against *Xoo*‐induced bacterial leaf blight in rice, outperforming both conventional thiodiazole copper (TC) and the individual guest molecule without compromising safety. Additionally, it exhibits the anticipated dual intervention, effectively disrupting biofilm and inhibiting T3SS (**Scheme** **1**). Furthermore, an expanded exploration of substrates and indication screening revealed that structurally analogous functional molecules, including BtP7, BtP11, and BtP20, also self‐assemble with *β*‐CD to form supramolecular materials. These assemblies exhibited superior in vivo efficacy against fungal rice sheath blight and blast, along with bacterial rice leaf streak, outperforming both the guest molecules themselves and their commercial counterparts. In summary, this supramolecular assembly strategy, grounded in *de novo* molecular design, proves both simplicity and high efficacy. By tailoring molecular structures to modulate mechanisms of action, it holds promise in addressing the escalating threat of bacterial and fungal diseases in rice, whose pathogenic mechanisms are incessantly evolving. This methodology not only showcases the potential for developing next‐generation antimicrobial agents but also provides a blueprint for designing materials with predefined properties for enhanced plant protection.

## 2 Results and Discussion

### 2.1 Identification of Optimal Guest Molecules Through In Vitro Antimicrobial Activity Evaluation

**Scheme 2:**
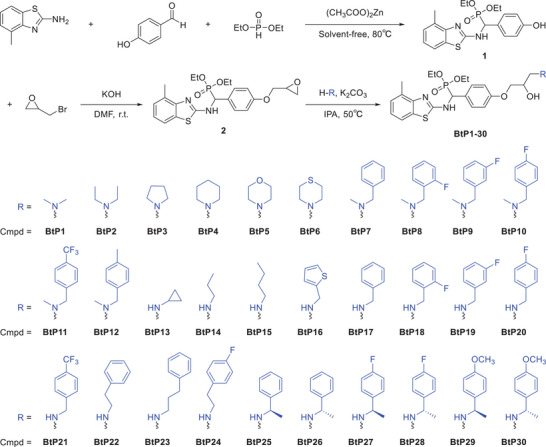
General synthetic procedure for target compounds BtP1‐BtP30.

Given the imperative for agrochemicals that are efficient, potent, and cost‐effective for agricultural applications, the use of *α*‐aminophosphate‐containing benzothiazole, derived from the antiviral agent dufulin, as a scaffold was proposed.^[^ ^35^, ^36^ ^]^ Subsequently, the introduction of an isopropylamine moiety, potentially imparting biofilm and T3SS intervention, into the scaffold led to the preparation of structurally novel small molecules BtP1‐BtP30 through an efficient three‐step synthesis process (**Scheme** **2**). The target molecules were characterized by nuclear magnetic resonance (NMR) spectroscopy and high‐resolution mass spectrometry (HRMS) (Figures S26–S156, Supporting Information). Their in vitro potency against the destructive bacterium *Xoo* was assessed via the classic turbidimetric method, with commercially available TC serving as a positive control. The results indicated that these compounds, with the exception of BtP16 (EC_50_ = 77.89 µg mL^−1^), exhibited superior antibacterial potency compared to TC (EC_50_ = 74.82 µg mL^−1^) (**Table** **1**).

**Table 1:** EC_50_ values of BtP1‐BtP30 against *Xoo* ^a)^.

Compd.	EC_50_[*µ*g mL^−1^]	Regression equation	R^2^	Compd.	EC_50_[*µ*g mL^−1^]	Regression equation	R^2^
BtP1	10.66 ± 0.13	y = 8.5884x – 3.8284	0.9107	BtP16	77.89 ± 1.65	y = 1.9816x + 1.2518	0.9968
BtP2	10.80 ± 0.91	y = 3.0676x + 1.8288	0.9740	BtP17	3.64 ± 0.24	y = 3.9308x + 2.7927	0.9545
BtP3	14.09 ± 1.24	y = 6.4353x – 2.3952	0.9983	BtP18	25.19 ± 1.62	y = 3.9308x – 0.5080	0.9989
BtP4	17.29 ± 0.62	y = 3.6847x + 0.4389	0.9997	BtP19	4.06 ± 0.22	y = 6.3170x + 1.1582	0.9043
BtP5	12.98 ± 1.74	y = 3.3389x + 1.2822	0.9578	BtP20	9.59 ± 0.49	y = 6.3070x – 1.1901	0.9928
BtP6	13.83 ± 1.71	y = 1.4731x + 3.3194	09949	BtP21	4.89 ± 0.89	y = 3.4633x + 2.3218	0.9210
BtP7	7.35 ± 0.04	y = 3.8392x + 1.6731	0.9938	BtP22	7.29 ± 0.24	y = 4.6684x + 0.9715	0.9687
BtP8	4.53 ± 0.12	y = 4.1614x + 2.2667	0.9127	BtP23	7.79 ± 0.22	y = 5.6005x + 0.0040	0.9045
BtP9	8.68 ± 0.01	y = 3.8226x + 1.4115	0.9968	BtP24	7.11 ± 0.20	y = 5.3062x + 0.4796	0.9263
BtP10	7.91 ± 0.36	y = 4.4021x + 1.0453	0.9844	BtP25	4.27 ± 0.23	y = 5.1959x + 1.7199	0.9639
BtP11	6.71 ± 0.18	y = 3.0160x + 2.5059	0.9924	BtP26	4.31 ± 0.13	y = 3.8360x + 2.5632	0.9071
BtP12	7.44 ± 0.34	y = 4.3960x + 1.1662	0.9121	BtP27	0.87 ± 0.09	y = 2.2001x + 5.1306	0.9259
BtP13	7.32 ± 0.59	y = 4.3582x + 1.2319	0.9334	BtP28	1.17 ± 0.04	y = 1.9752x + 4.5068	0.9711
BtP14	14.74 ± 1.12	y = 2.2378x + 2.3845	0.9894	BtP29	6.09 ± 0.70	y = 3.0357x + 2.6160	1
BtP15	6.53 ± 0.90	y = 1.4939x + 3.7824	0.9711	BtP30	6.70 ± 0.21	y = 2.9093x + 2.5964	0.9062
*β*‐CD	>100			dufulin^b)^	> 100		
BtP27@*β*‐CD	0.77 ± 0.05	y = 2.5618x + 5.2941	0.9598	TC ^b)^	74.82 ± 3.01	y = 2.6587x + 0.0174	0.9714

^a)^ The statistical analysis was conducted by ANOVA method at the condition of equal variances assumed (*P* > 0.05) and equal variances not assumed (*P* < 0.05);

^b)^ Commercially available agrochemicals as the positive control. Abbreviation: TC, thiodiazole copper.

To investigate the structure‐activity relationship (SAR), a 3D‐QSAR model was developed using Comparative Molecular Field Analysis (CoMFA) (Figure S2, Tables S1 and S2, Supporting Information) and Comparative Molecular Similarity Index Analysis (CoMSIA) (Figure S3, Tables S3 and S4, Supporting Information).^[^ ^37^ ^]^ Molecular alignment was performed prior to modeling to ensure optimal spatial correspondence, thereby improving computational efficiency and accuracy. To construct the 3D‐QSAR model, 23 molecules were selected as the training set, with the remainder serving as the test set. Both the CoMFA and CoMSIA models exhibited a coefficient of determination (R^2^) and R^2^ Scramble greater than 0.94, indicating a robust fit and reliable predictive performance (Table S5, Supporting Information). In the CoMFA model, the steric and electrostatic fields contributed 69.00% and 31.00% to the bioactivity, respectively, highlighting the predominant role of steric interactions in determining antibacterial potency (Figure S2, Supporting Information). Visual analysis revealed green contours around the diethyl phosphonate fragment in the steric field, indicating that larger substituents enhance activity against *Xoo*. Consequently, molecules with bulky diethyl phosphonate groups demonstrated high in vitro potency. In the CoMSIA model, the contributions from Gaussian Steric, Hydrophobic, H‐bond Acceptor, H‐bond Donor, and Electrostatic fields were 46.20%, 21.60%, 13.70%, 10.10%, and 8.50%, respectively, underscoring the considerable impact of the steric field on in vitro activity relative to the others (Figure S3, Supporting Information).

Similar to CoMFA, the steric field analysis in CoMSIA indicated that bulky substituents located in green regions correlate with enhanced antibacterial potency. For instance, compounds BtP1‐BtP6 with smaller substituents exhibited EC_50_ values between 10.66 and 17.29 µg mL^−1^, whereas BtP7‐BtP12 with larger substituents displayed EC_50_ values ranging from 4.53 to 8.68 µg mL^−1^. Notably, chiral amine‐substituted compounds BtP25‐BtP30, possessing favorable spatial effects, demonstrated enhanced antibacterial activity, with EC_50_ values between 0.87 and 6.70 µg mL^−1^. Among these, BtP27 exhibited the strongest activity against *Xoo* with an EC_50_ of 0.87 µg mL^−1^, ≈1/90th of that TC (EC_50_ = 74.82 µg mL^−1^). Given its high potency, BtP27 emerges as a prime candidate for the development of host‐guest assemblies, warranting further investigations into its assembly behavior, foliar affinity, in vivo efficacy, and underlying antibacterial mechanisms.

### 2.2 Successful Fabrication and Characterization of Host‐Guest Complex BtP27@*β*‐CD

**Figure 1:**
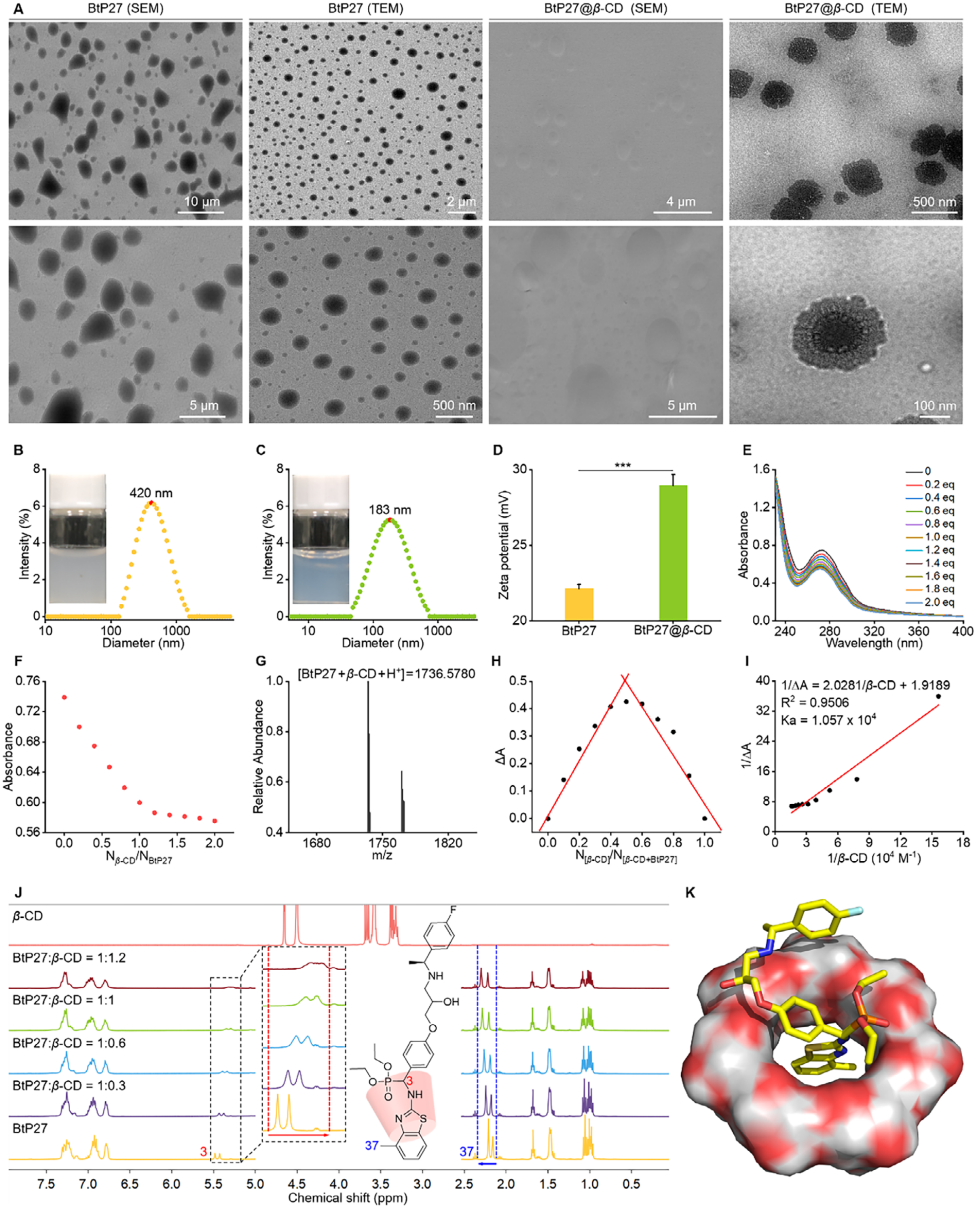
Characterization of BtP27@*β*‐CD. A) SEM and TEM images of BtP27 and BtP27@*β*‐CD. B) Appearance and particle size distributions of BtP27 at 200 µg mL^−1^. C) Appearance and particle size distributions of BtP27@*β*‐CD at 200 µg mL^−1^. D) Zeta potential of BtP27 and BtP27@*β*‐CD at 200 µg mL^−1^. E) UV–vis titration curves of BtP27 (40 µM) with increasing molar equivalents of *β*‐CD (0–2.0 eq). F) Linearity of absorption spectra at 270 nm of 40 µM BtP27 with respect to *β*‐CD at various concentrations. G) HRMS spectrum of BtP27@*β*‐CD. H) Job's plots for ΔA at 270 nm, with a total concentration of 80 µM for BtP27 and *β*‐CD in aqueous solution. I) Benesi–Hildebrand plots of 1/ΔA versus 1/*β*‐CD. J) ^1^H NMR spectra of BtP27, *β*‐CD, and their mixtures at various molar ratios (1:0.3, 1:0.6, 1:1, and 1:1.2) in D_2_O, with a BtP27 concentration of 4.0 mM. K) Molecular docking of BtP27 and *β*‐CD. Statistically significant differences between the means were analyzed with one‐way ANOVA, followed by the least significant difference (LSD) post‐hoc test in (D) (n = 3; *p < 0.05, **p < 0.01, ***p < 0.001; n.s. = no significance).

The supramolecular material BtP27@*β*‐CD was prepared as follows. Initially, BtP27 (16.62 µM) dissolved in 0.2 mL of THF was added dropwise to 2.0 mL of deionized water containing *β*‐CD (16.62 µM). As THF evaporated naturally, *β*‐CD encapsulated the hydrophobic segment of BtP27, preventing its direct exposure to water and facilitating the formation of self‐assembled aggregates, which exhibited comparable in vitro antibacterial activity to BtP27, with an EC_50_ value of 0.77 µg mL^−1^ (Table 1). Scanning electron microscope (SEM) and transmission electron microscope (TEM) images revealed the morphology of both the guest molecule, which manifested as spherical outlines, and the supramolecular complex, which exhibited sunflower‐shaped morphology, providing direct evidence of supramolecular formation (**Figure** **1**A).^[^ ^38^ ^]^ Moreover, BtP27@*β*‐CD solutions exhibited Tyndall scattering across varying concentrations upon laser irradiation (Figure S4, Supporting Information), alongside a discernible inflection point in surface tension at 0.41 µg mL^−1^ (Figure S5, Supporting Information). These are characteristic features of micelle formation,^[^ ^39^, ^40^ ^]^ indicating that BtP27@*β*‐CD can form supramolecular micelles in aqueous media, with the observed inflection point corresponding to the critical micelle concentration (CMC).

Further characterization studies demonstrate that BtP27@*β*‐CD achieves enhanced optical transparency in aqueous media, accompanied by a reduced average particle size (from 420 to 183 nm, Figure 1B,C) and an elevated Zeta potential (from 22.14 to 28.96 mV, Figure 1D) compared to the guest molecule alone. These findings suggest that the encapsulation by *β*‐CD, with its abundance hydrophilic hydroxyl groups, improves the aqueous solubility, dispersity, and stability of the guest in aqueous media. To further substantiate the enhancement in solubility conferred by *β*‐CD, a detailed phase solubility analysis was performed. As illustrated in Figure S6 (Supporting Information), the solubility of BtP27 in aqueous solution increases linearly with increasing *β*‐CD concentration, presenting an A_L_‐type solubility curve. This indicates that the addition of *β*‐CD enhances solubility and supports that *β*‐CD and BtP27 may form a 1:1 complex to achieve this effect.

Following the confirmation of the host‐guest complex formation, varying concentrations of *β*‐CD were gradually added to a 40 µM BtP27 solution for UV–vis titration to determine the stoichiometry of the host‐guest complex (Figure 1E). The guest molecule alone exhibited a characteristic absorption peak at 270 nm, attributed to the π→π* electron transition of benzothiazole. Upon addition of *β*‐CD, absorbance at 270 nm decreased progressively until reaching an equimolar concentration, indicating a 1:1 stoichiometry for the host‐guest binding (Figure 1F). This conclusion was confirmed by HRMS (Figure 1G; Figure S16, Supporting Information), which revealed a molecular weight of 1736.5780 closely matching the theoretical value of [BtP27@*β*‐CD + H^+^] with a minimal deviation of merely −9.5552 ppm, and by Job's plot (Figure 1H), which exhibited a peak in ΔA at a *β*‐CD molar fraction of 0.5. Moreover, the Benesi‐Hildebrand equation derived from the titration experiment yielded a binding affinity of 1.057 × 10^4^ M^−^¹ (Figure 1I), underscoring the strong binding interaction between BtP27 and *β*‐CD.

To elucidate the encapsulation position of BtP27 within the *β*‐CD cavity and to preliminarily capture the driving force for self‐assembly, ^1^H NMR titration was performed at various molar ratios (Figure 1J; Table S6, Supporting Information). Upon the addition of 0.6 equivalents of *β*‐CD, the signal peak of H‐3 was blunted from its sharp profile and shifted upfield, accompanied by a chemical shift change (Δ*δ*) of ‐0.088 ppm. This shift is attributed to the shielding effect of the hydrophobic cavity of *β*‐CD on the H‐3 protons, suggesting successful encapsulation of the molecular segment containing the H‐3 proton within the *β*‐CD cavity. Concurrently, the protons of the methyl group on the benzothiazole (H‐37) suffered from a downfield shift, with a Δ*δ* of 0.052 ppm, providing compelling evidence of a deshielding effect dominated by hydroxyl groups, indicating these protons reside outside the cavity and near the exit. As the concentration of *β*‐CD increased to one equivalent, the observed chemical shift changes were further amplified, while other proton shifts remained consistent. These observations collectively indicate that portions of the guest core, including the methylamino, thiazole, and part of the phenyl group, are encapsulated within the *β*‐CD cavity, with the remaining portions outside. These experimental observations are further corroborated by computational simulations (Figure 1K), providing additional insights into the encapsulation position.

**Figure 2:**
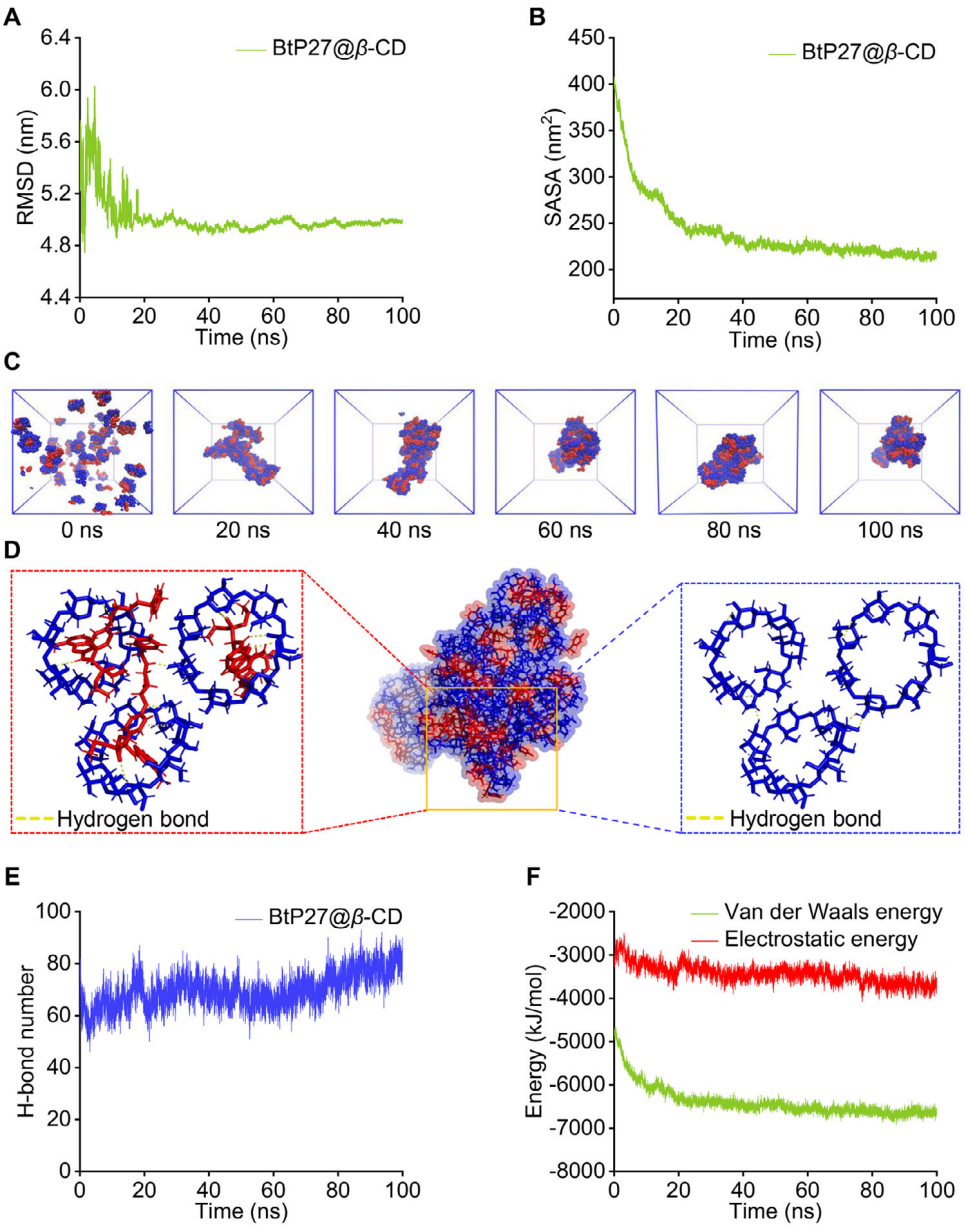
MD simulations during self‐assembly of Btp27 and *β*‐CD. A) RMSD variation of the system over simulation time. B) SASA variation of the system over simulation time. C) Structural evolution of the supramolecular system during simulations. D) Intermolecular interactions within the supramolecular system. E) Variation in the number of intermolecular hydrogen bonds over simulation time. F) Variation in intermolecular binding energies over simulation time.

To investigate the mechanisms and driving forces underlying the self‐assembly between BtP27 and *β*‐CD, we conducted further analysis via computer simulations using the GROMACS 2023.3 software package with the GAFF force field parameters (details are provided in Section 2.4.9 of the Supporting Information).^[^ ^41^, ^42^ ^]^ Over the course of a 100 ns molecular dynamics (MD) simulation, the RMSD value of the BtP27@*β*‐CD system exhibited minimal fluctuations, averaging at 5.007 ± 0.1375 nm (**Figure** **2**A), indicating stability in the supramolecular system throughout the aggregation process. The solvent‐accessible surface area (SASA) of the system exhibited a sharp decline at the beginning of the simulation, subsequently reaching a plateau, with a mean value of 240.5960 ± 34.7808 nm^2^ (Figure 2B). This trend indicates the progressive formation of compact and stable nanoclusters. To elucidate the conformational dynamics and the compactness of the final composite structure throughout the simulation, cluster analysis was conducted on the simulated trajectories of the composite system utilizing the Gromacs clustering tool. Representative conformations were extracted at 20 ns intervals to serve as average conformations for those periods (Figure 2C). The findings revealed that BtP27 and *β*‐CD self‐assembled into spherical nanoclusters exhibiting a sunflower‐like morphology.

Further simulation analysis of intermolecular interactions within the composite system unveiled the presence of hydrogen bonds, hydrophobic interactions, and hydrophilic interactions throughout the host‐guest recognition process (Figure 2D,E). In the intricate supramolecular aggregation, the mean van der Waals energy was recorded at −6393.12 ± 344.8735 kJ mol^−1^, compared to the mean electrostatic energy of −3436.4500 ± 219.1312 kJ mol^−1^ (Figure 2F), indicating that van der Waals forces are the predominant driving force for this assembly. Additionally, numerous hydrogen bond interactions were identified, corresponding to the abundance of hydrogen bond donors and acceptors within the structures of BtP27 and *β*‐CD. Grounded in these driving force observations, we propose a plausible mechanism for the self‐assembly process of BtP27@*β*‐CD. Initially, BtP27 and *β*‐CD form binary building blocks through host‐guest recognition driven by hydrogen bonds, hydrophobic interactions, and hydrophilic interactions. Subsequently, the aggregation of these building blocks primarily occurs under the aegis of van der Waals forces and hydrogen bonding interactions, with the hydrophilic *β*‐CD moieties oriented outwardly to optimize the encapsulation of hydrophobic termini inside, minimizing exposure to the aqueous environment. This self‐assembly process gradually leads to the formation of sunflower‐shaped material.

### 2.3 Superior Foliar Affinity of BtP27@*β*‐CD on Superhydrophobic Rice Leaves

**Figure 3:**
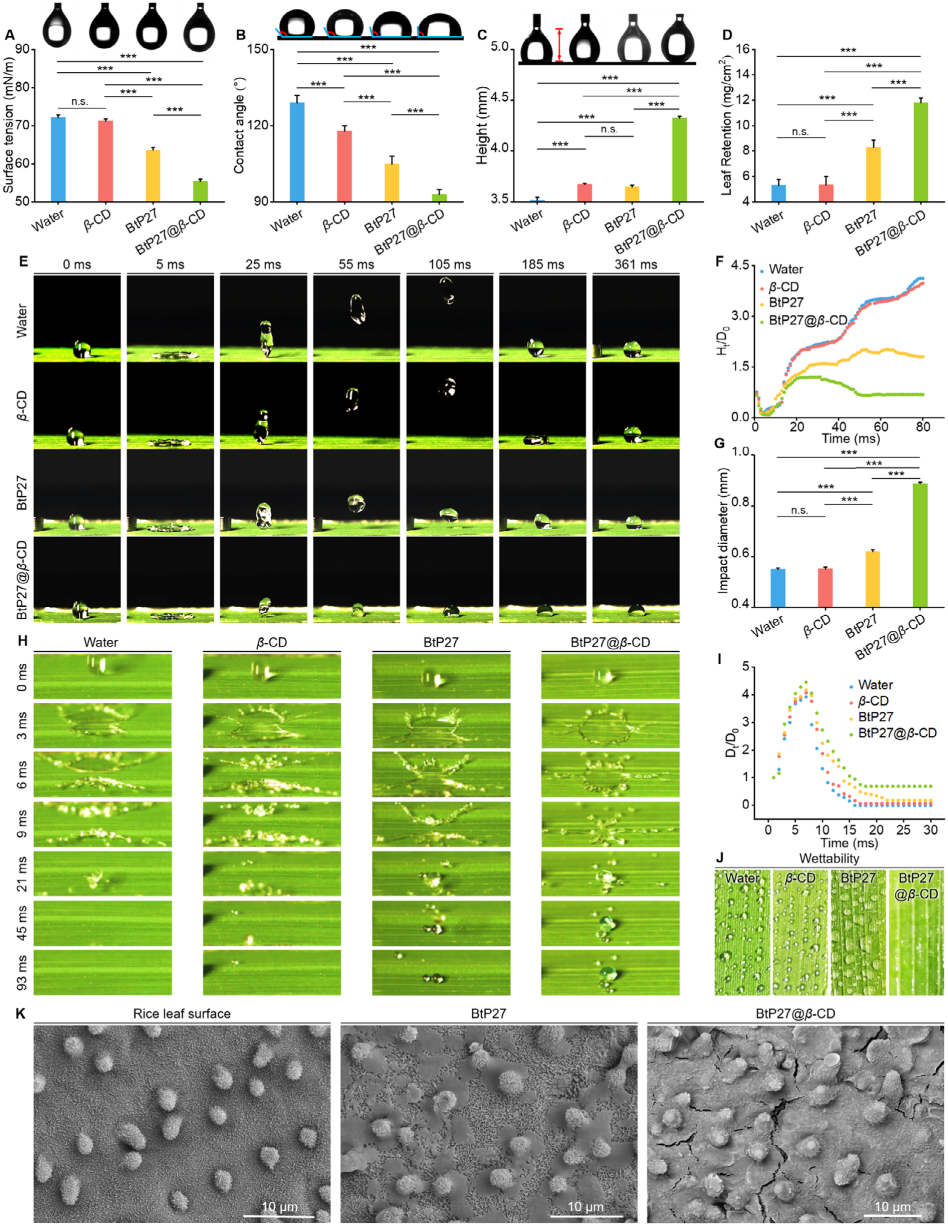
Physicochemical properties and foliar affinity of BtP27 and BtP27@*β*‐CD. A) Surface tension measurements of water, *β*‐CD, BtP27, and BtP27@*β*‐CD solutions at 200 µg mL^−1^ (n = 6). B) Contact angles of water, *β*‐CD, BtP27, and BtP27@*β*‐CD solutions at 200 µg mL^−1^ on rice leaves (n = 6). C) Lift‐off height of water, *β*‐CD, BtP27, and BtP27@*β*‐CD droplets at 200 µg mL^−1^ (n = 6). D) Liquid holding capacity of rice leaves after immersion in water, *β*‐CD, BtP27, and BtP27@*β*‐CD solutions at 200 µg mL^−1^ (n = 6). E) Images depicting the droplet bouncing behavior of water, *β*‐CD, BtP27, and BtP27@*β*‐CD solutions (200 µg mL^−1^) on the rice surface (from a height of 10 cm). F) Time‐resolved normalized rebound height (H_t_/D_0_) of water, *β*‐CD, BtP27, and BtP27@*β*‐CD droplets, where H_t_ denotes the distance between the rice leaf surface and the top of the droplet and D_0_ represents the droplet diameter at the moment of initial contact with the leaf surface. G) Final spreading diameters of water, *β*‐CD, BtP27, and BtP27@*β*‐CD droplets from Video S1 (Supporting Information) (n = 3). H) Images illustrating the droplet splashing behavior of water, *β*‐CD, BtP27, and BtP27@*β*‐CD (200 µg mL^−1^) on the rice leaves (from a height of 30 cm). I) Time‐resolved normalized contact diameter (D_t_/D_0_) of water, *β*‐CD, BtP27, and BtP27@*β*‐CD droplets over time, where D_t_ represents the contact diameter and D_0_ represents the droplet diameter at the moment of initial contact with the leaf surface. J) Visual documentation of the wetting and spreading behavior of water, *β*‐CD, BtP27, and BtP27@*β*‐CD solutions at 200 µg mL^−1^. K) SEM images of untreated rice leaf, rice leaf treated with BtP27 or BtP27@*β*‐CD. Statistically significant differences between the means were analyzed with one‐way ANOVA, followed by the least significant difference (LSD) post‐hoc test in (A‐D, G) (For all studies, n ≥ 3; *p < 0.05, **p < 0.01, ***p < 0.001; n.s. = no significance).

Effective foliar deposition and retention are of paramount importance for augmenting pesticide efficacy while mitigating environmental burden.^[^ ^43^, ^44^ ^]^ Thus, we analyzed the properties of BtP27@*β*‐CD and examined its deposition and retention on superhydrophobic rice leaves. Initially, the surface tension of the droplets was quantified (**Figure** **3**A), revealing that our engineered supramolecular material reduced surface tension by 8.18 mN m^−1^ compared to the guest molecule alone. This reduction implies improved spreading and wetting performance of BtP27@*β*‐CD‐laden droplets during application. This inference was further supported by a 12° decrease in contact angle (Figure 3B), a 0.68 mm reduction in lift‐off height from a needle tip (Figure 3C), and a 3.52 mg cm^−^^2^ of increase in liquid holding capacity (LHC) on rice leaves (Figure 3D; Figure S7, Supporting Information) for the supramolecular material compared to the guest molecule alone. Subsequently, the dynamics of droplet rebound (released from a height of 10 cm) were captured using a high‐speed camera (Figure 3E; Video S1, Supporting Information). During this observation, water droplets and those containing *β*‐CD or the guest molecule alone exhibited complete spherical rebound accompanied by higher rebound heights and smaller spreading diameters (Figure 3F,G). Conversely, droplets enriched with BtP27@*β*‐CD remained adhered to the leaf surface throughout the dynamic process following initial contact, characterized by diminished maximum rebound height (2.92 mm vs 11.11 mm for water, 11.07 mm for *β*‐CD, and 5.45 mm for BtP27) (Figure S8, Supporting Information) and augmented final spreading diameter (0.89 mm vs 0.55 mm for water, 0.54 mm for *β*‐CD, 0.62 mm for BtP27). The normalized rebound height over time (H_t_/D_0_) further underscored the superior rebound suppression of BtP27@*β*‐CD droplets relative to others (Figure 3F). In the splash dynamics analysis (Figure 3H; Video S2, Supporting Information), droplets of BtP27@*β*‐CD splashed within a restricted range and height, resulting in considerable residue on the leaf surface. In contrast, water droplets and those containing *β*‐CD or BtP27 splashed omnidirectionally, leaving negligible or no residue. This observation aligned with the normalized spreading diameter over time (D_t_/D_0_), where the final equilibrium values for all groups except BtP27@*β*‐CD approached zero (Figure 3I). It was proposed that the deposition of substantial BtP27@*β*‐CD on the leaf surface during the droplet impact process would modulate the wettability of the hydrophobic surface due to its inherent hydrophilicity, thus creating pinning points and delaying droplet retraction.^[^ ^45^ ^]^

The attributes exhibited in the experiments naturally bestow BtP27@*β*‐CD droplets with superior spreading and wetting performance, as visually confirmed by the application of water and various solutions onto rice leaves (Figure 3J). Upon sprayed directly onto the leaves, both water, *β*‐CD, and BtP27 solutions tended to form beads and failed to spread adequately, whereas BtP27@*β*‐CD droplets dispersed remarkably evenly, ensuring thorough leaf wetting. This phenomenon was ascribed to the abundance of hydroxyl functional groups on *β*‐CD, which established hydrogen bonds with fatty acids, alcohols, and aldehydes within the wax layer of leaves, thereby augmenting the interaction between BtP27@*β*‐CD and the leaf surface. SEM imaging was subsequently employed to observe the behavior of the guest molecule and supramolecular material on rice leaves (Figure 3K). Untreated rice leaves exhibited distinct features such as a waxy cuticle, trichomes, and stomata. The application of BtP27 alone resulted in heavy deposits unevenly distributed across the leaf surface. In contrast, the spherical aggregates of BtP27@*β*‐CD adhered firmly to the periodically distributed sinusoidal grooves, along with the orderly arranged micropapillae and nanosplinter structures on rice leaf surfaces, facilitating uniform distribution and coverage, with the potential to induce a wetting transition from the Cassie state to the Wenzel state. These findings collectively indicate enhanced adhesion, penetration, and deposition characteristics on hydrophobic leaves, a feat unattainable with conventional bactericides.

### 2.4 High In Vivo Efficacy of BtP27@*β*‐CD Against Bacterial Leaf Blight

**Figure 4:**
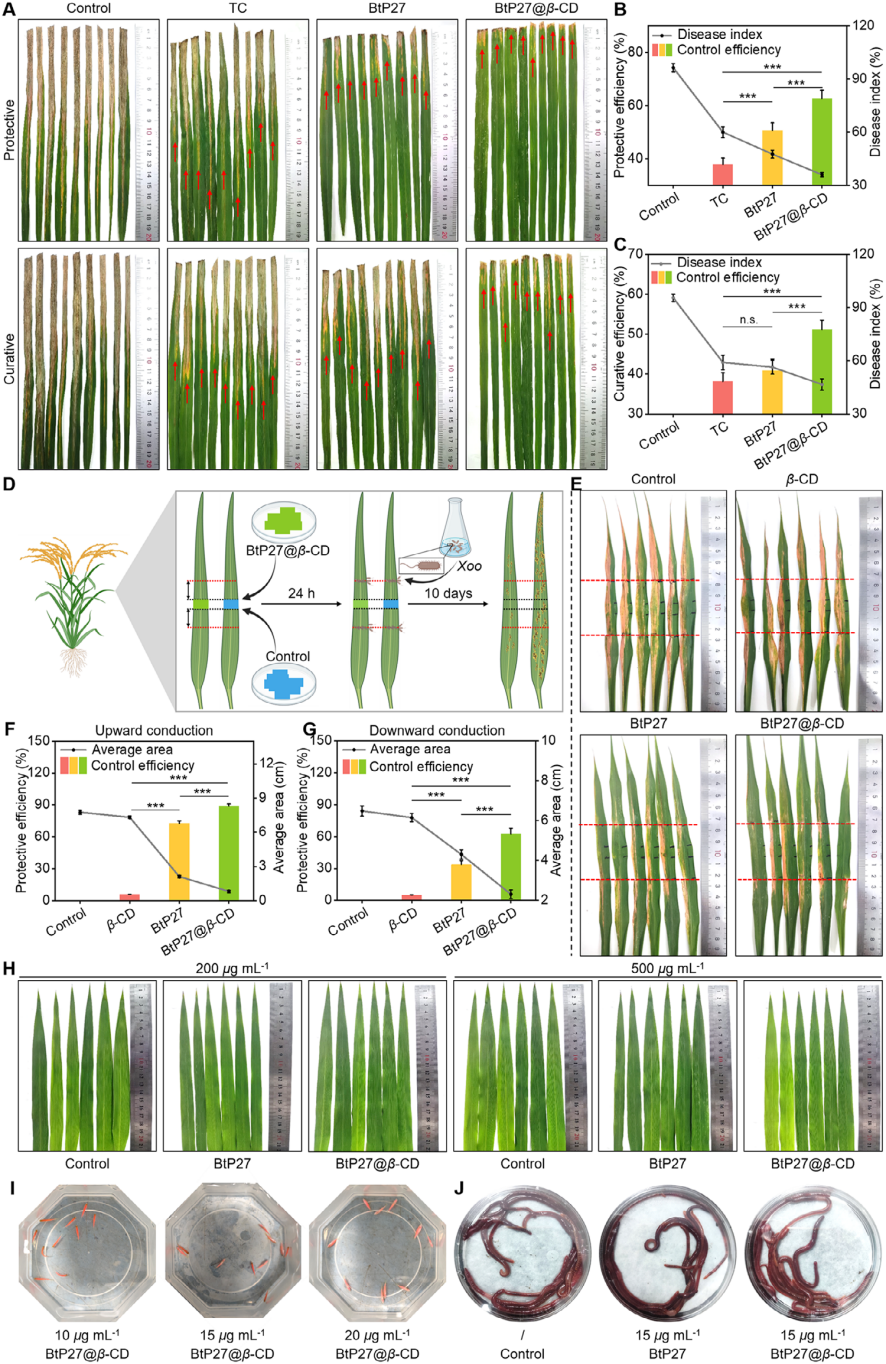
In vivo antibacterial potency of BtP27 and BtP27@*β*‐CD in rice. A) Photographic documentation of efficacy of BtP27, BtP27@*β*‐CD, and TC against rice bacterial leaf blight at 200 µg mL^−1^. B,C) Protective (B) and curative (C) efficiencies as determined from the above photographs. D) Schematic diagram of in vivo experiments evaluating uptake and translocation properties. E) Visual documentation of protective efficacy of BtP27 and BtP27@*β*‐CD (200 µg mL^−1^) post‐uptake and translocation. F) Protective efficiencies after upward conduction. G) Protective efficiencies after downward conduction. H) Photographs of phytotoxicity tests on rice leaves treated with BtP27 and BtP27@*β*‐CD at 200 and 500 µg mL^−1^. I,J) Photographs showing acute toxicity tests toward zebrafish (I) and earthworm (J), captured 96/48 h after exposure to control, BtP27, and BtP27@*β*‐CD at specified concentrations. Statistically significant differences between the means were analyzed with one‐way ANOVA, followed by the least significant difference (LSD) post‐hoc test in (B, C, F, G) (For all studies, n = 6; *p < 0.05, **p < 0.01, ***p < 0.001; n.s. = no significance).

Our primary objective is to identify highly effective bactericides with distinct physicochemical properties, necessitating in vivo experimentation. To this end, the efficacy of the selected molecules against *Xoo*‐induced bacterial leaf blight in rice was evaluated using the classical leaf‐cutting method.^[^ ^46^ ^]^ The results indicated severe disease symptoms on rice leaves in both the control group (**Figure** **4**A–C; Figure S20, Supporting Information). In contrast, treatment with a low‐dose of 200 µg mL^−1^ of BtP27@*β*‐CD alleviated the disease symptoms, demonstrating efficacy (62.67%) and curative efficacy (51.16%), surpassing those of BtP27 (protective activity: 50.69%, curative activity: 40.93%) and commonly used commercial bactericide TC (20% SC, protective activity: 37.78%, curative activity: 38.13%) at the same concentration (Table S7, Supporting Information).

### 2.5 Representative Characteristics of BtP27@*β*‐CD Mirroring Dufulin

To determine whether the guest molecule and the supramolecular material inherit the uptake and translocation characteristics of dufulin, we conducted an in vivo investigation to evaluate these properties by examining their efficacy in mitigating *Xoo*‐induced bacterial leaf blight, employing the leaf‐clipping inoculation method.^[^ ^47^ ^]^ Specifically, filter papers (1 × 1 cm) soaked with the treatment solutions were affixed to the middle section of rice leaves, with *Xoo* inoculated 2 cm above and below the application sites (Figure 4D). Ten days post‐inoculation, the control and *β*‐CD groups exhibited extensive orange lesions across the entire leaves, signifying severe infection. In contrast, pretreatment with 200 µg mL^−1^ of BtP27 and BtP27@*β*‐CD alleviated disease symptoms throughout the leaves, demonstrating distinct prophylactic efficacy on both the apical and basal regions of the leaves (Figure 4E–G; Figure S21, Supporting Information). In the BtP27@*β*‐CD‐treated cohort, lesion lengths measured 0.81 cm in the upper leaf section and 2.31 cm in the lower section, corresponding to protective efficacies of 88.90% and 62.47%. By comparison, lesion lengths in the BtP27‐treated group were recorded at 2.11 cm and 4.30 cm, with protective efficacies of 72.72% and 33.57%, respectively (Table S8, Supporting Information). These findings unequivocally demonstrate that both the supramolecular material and the guest molecule are effectively absorbed and translocated by rice leaves, with superior translocation toward the leaf apex compared to the base. Furthermore, the protective efficacy of BtP27@*β*‐CD post‐uptake and translocation exceeded that of BtP27 throughout the entire leaf. Furthermore, given that dufulin typically functions as an antiviral inducer capable of potentiating salicylic acid‐mediated plant disease resistance pathways, and considering that *OsNPR1*, cloned from *Oryza sativa*, serves as a pivotal regulator within this signaling cascade,^[^ ^48^ ^]^ we investigated whether BtP27 and BtP27@*β*‐CD inherit this activation profile of dufulin. This was accomplished by monitoring *OsNPR1* expression in rice leaves following treatment with aforementioned molecules. Our findings revealed that treatment with BtP27 and BtP27@*β*‐CD elicited a relative upregulation of *OsNPR1* expression by 76.04% and 114.01%, respectively, compared to control, indicating that both molecules have the capacity to activate salicylic acid‐induced plant defense mechanisms (Figure S9, Supporting Information).

### 2.6 Safety Assurance of BtP27@*β*‐CD for Target and Non‐Target Organisms

To evaluate the safety of the guest molecule and supramolecular material in target crops and non‐target organisms, toxicity experiments were conducted on rice, zebrafish, and earthworms. Initially, the guest molecule and supramolecular material were applied to rice leaves under comparable growth conditions (Figure 4H; Figure S22, Supporting Information). At both the effective concentration (200 µg mL^−1^) and a dose far exceeding the effective concentration (500 µg mL^−1^), rice plants exhibited normal growth with no visible signs of phytotoxicity, such as scorching, deformities, or chlorosis. Following a 96‐h exposure to BtP27 and BtP27@*β*‐CD, zebrafish exhibited no deleterious impact on survival or viability (Figure 4I). The LC_50_ values for BtP27 (13.86 µg mL^−1^) and BtP27@*β*‐CD (14.05 µg mL^−1^) both exceeding the 10.00 µg mL^−1^ threshold, which corresponds to low toxicity according to Baran Alper's criteria (LC_50_ > 10 µg mL^−1^), confirming their minimal toxicity (Tables S9 and S10, Supporting Information).^[^ ^49^ ^]^ Furthermore, at a concentration of 15 µg mL^−1^, both entities granted unaltered survival and vitality in earthworms after a 48‐h exposure period, with no significant differences from the control group (Figure 4J), highlighting their strong biosafety profile.^[^ ^50^ ^]^ These results collectively indicate that BtP27 and BtP27@*β*‐CD exhibit favorable safety profiles, with negligible adverse effects on both target and non‐target organisms. According to predictive analyses conducted via the ProTox 3.0 platform (https://tox‐new.charite.de),^[^ ^51^ ^]^ BtP27 demonstrated potential activity against merely 6 out of 46 toxicity targets, with probability scores remaining below 0.7 for all endpoints except respiratory toxicity. In contrast, its structural analog, dufulin, exhibited predicted interactions with 8 toxicity targets, three of which displayed probability values exceeding 0.7 (see Predictive Data Tables of BtP27 using ProTox‐3.0 and Predictive Data Tables of dufulin using ProTox‐3.0 for details). This comparative toxicological profile suggests that BtP27 offers a superior safety advantage relative to dufulin, particularly in terms of reduced off‐target pharmacological interactions.

### 2.7 Potent Inhibition of *Xoo* Biofilm Formation by BtP27@*β*‐CD

**Figure 5:**
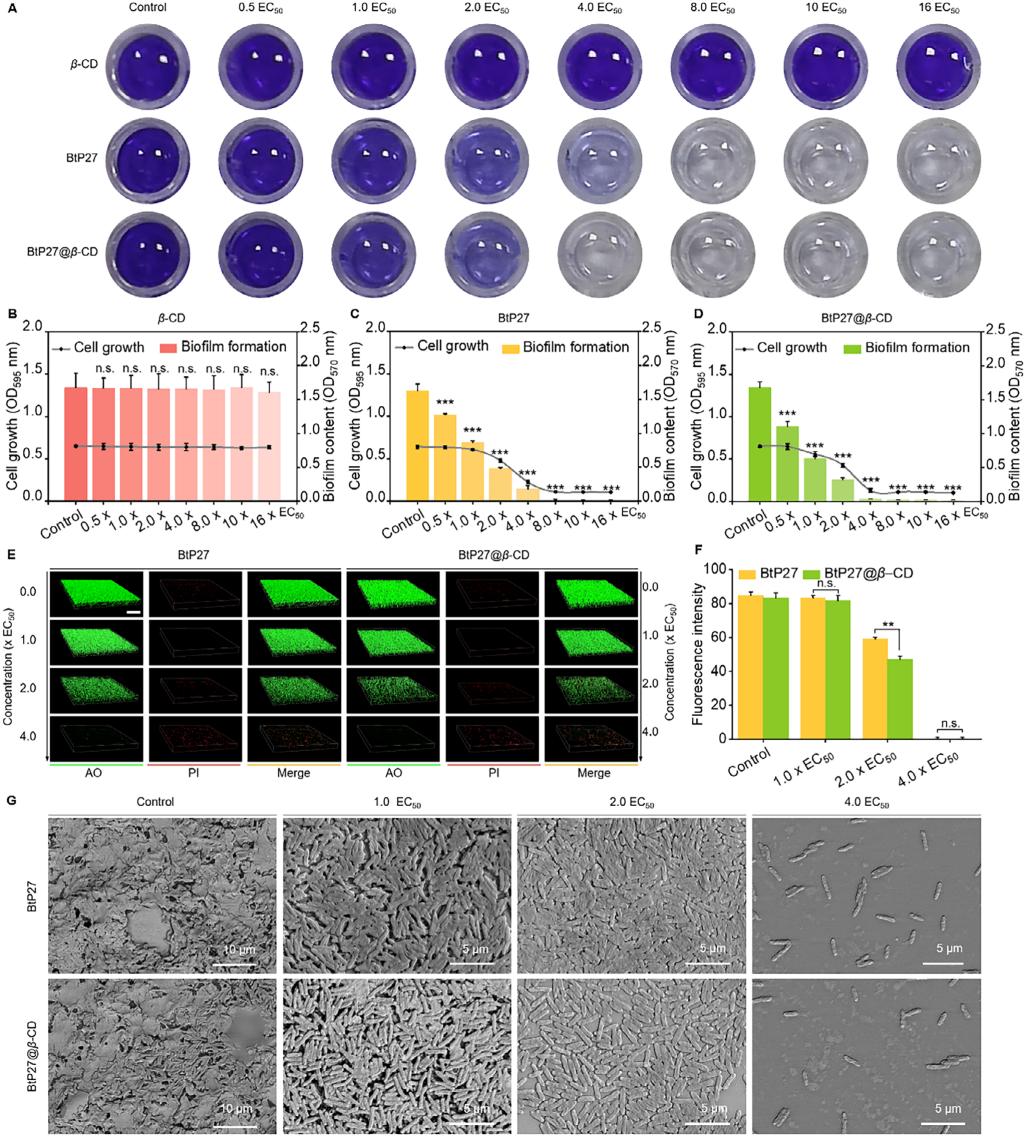
Inhibition of biofilm formation by BtP27@*β*‐CD. A) Crystal violet staining assays of *β*‐CD, BtP27, and BtP27@*β*‐CD against *Xoo*‐biofilm formation at various concentrations over 48 h. B‐D) Optical density measurements at 570 nm and 595 nm to quantify the remaining biofilm and bacterial growth after incubation with different doses of *β*‐CD (B), BtP27 (C), and BtP27@*β*‐CD (D) for 48 h (n = 8). E) CLSM 3D images of *Xoo* stained with AO and PI after exposure to different doses of BtP27 or BtP27@*β*‐CD for 48 h, scale bar: 50 *µm*. F) Statistical analysis of green fluorescence intensity indicating *Xoo* growth and biofilm inhibition from the above CLSM 3D images using ImageJ (n = 3). G) SEM imagines showing the inhibitory effects of BtP27 and BtP27@*β*‐CD on *Xoo* growth and biofilm formation at various concentrations. Statistically significant differences between the means were analyzed with one‐way ANOVA, followed by the least significant difference (LSD) post‐hoc test in (B‐D, F) (For all studies, n ≥ 3; *p < 0.05, **p < 0.01, ***p < 0.001; n.s. = no significance).

Following the confirmation of the potent in vitro and in vivo antibacterial potency and safety of BtP27 and BtP27@*β*‐CD, we proceeded to investigate their potential to disrupt biofilm formation and inhibit T3SS. *Xoo*, a prominent Gram‐negative pathogen known for causing biofilm‐associated infections, was selected as the primary subject for mechanistic studies. Initially, crystal violet staining assays were performed to evaluate the impact of BtP27, *β*‐CD, and BtP27@*β*‐CD on biofilm formation. The findings revealed that *β*‐CD alone exerted no measurable impact on *Xoo* biofilm formation, even at elevated concentrations of 13.92 µg mL^−1^ (16 × EC_50_) (**Figure** **5**A,B). In contrast, both BtP27 and BtP27@*β*‐CD inhibited biofilm formation in a dose‐dependent manner, with suppression observed as early as 0.44 µg mL^−1^ (0.5 × EC_50_), and inhibition levels progressively increased with concentration (Figure 5C,D). Remarkably, BtP27@*β*‐CD demonstrated near‐complete inhibition (97.80%) at 3.48 µg mL^−1^ (4.0 × EC_50_), while BtP27 almost halted biofilm formation (99.26% inhibition rate) at 6.96 µg mL^−1^ (8.0 × EC_50_). Intriguingly, BtP27@*β*‐CD minimally affected bacterial growth, yet effectively suppressed biofilm formation at low concentrations (< 1.74 µg mL^−1^) (Figures S10 and S11A, Supporting Information). Meanwhile, it not only thwarted biofilm formation but also annihilated embedded bacteria at elevated concentrations. This dual‐concentration dependency enables selective biofilm suppression without affecting bacterial viability at lower doses, transitioning to combined antibiofilm‐bactericidal activity at higher concentrations.

To further corroborate the inhibition of biofilm formation and the bactericidal efficacy against encapsulated bacteria, 3D imaging of cells stained with acridine orange (AO) or propidium iodide (PI) was performed using confocal laser scanning microscopy (CLSM) (Figure 5E). Staining with AO and PI revealed a gradual reduction in green fluorescence, accompanied by the emergence of red fluorescence, as concentrations rose from 0 to 3.48 µg mL^−1^ (4.0 × EC_50_). At 3.48 µg mL^−1^ (4.0 × EC_50_), green fluorescence was nearly eliminated, with relative intensities reduced to 5.50% and 5.48% for BtP27 and BtP27@*β*‐CD, respectively (Figure 5F). These results demonstrate that both BtP27 and BtP27@*β*‐CD effectively reduced viable *Xoo* cell counts and suppressed biofilm formation. SEM images of *Xoo* clusters treated with varying concentrations (0–3.48 µg mL^−1^) of the molecules further substantiated their inhibitory effects on biofilm formation and bacterial growth, as evidenced by the progressive reduction in biofilm presence and decline in bacterial population (Figure 5G).

### 2.8 Effective Eradication of Mature *Xoo* Biofilm by BtP27@*β*‐CD

**Figure 6:**
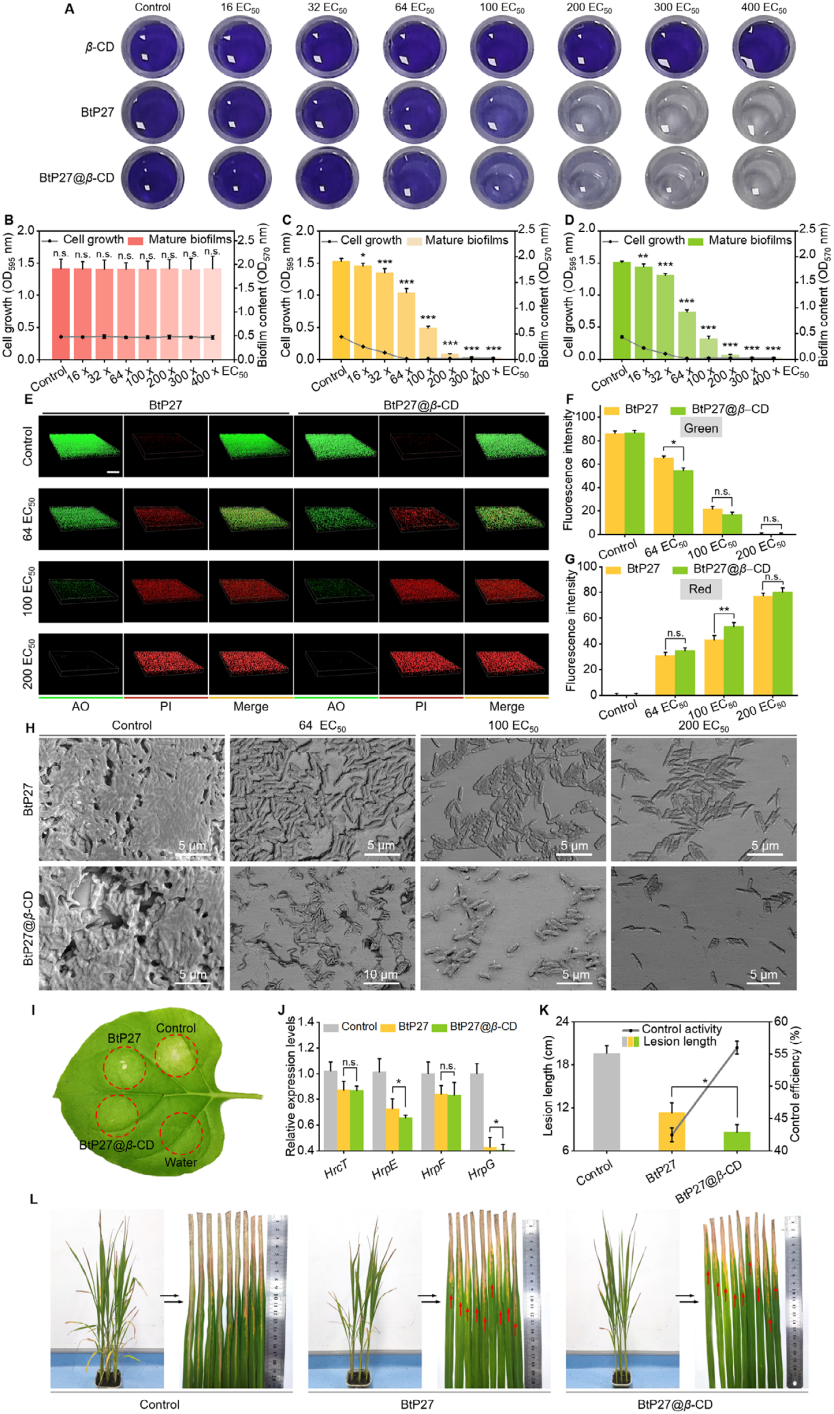
BtP27@*β*‐CD eradicates mature biofilms, downregulates the expression of T3SS‐related genes, and reduces pathogenicity. A) Crystal violet staining assays demonstrating the efficacy of *β*‐CD, BtP27, and·BtP27@*β*‐CD in eradicating pre‐established biofilms at specified concentrations. B‐D) Optical density measurements at 570 nm and 595 nm to quantify the remaining biofilm and bacterial growth after incubation with different doses of *β*‐CD (B), BtP27 (C), and BtP27@*β*‐CD (D) for 24 h (n = 8). E) CLSM 3D images of *Xoo* stained with AO and PI after exposure to different doses of BtP27 or BtP27@*β*‐CD for 24 h, scale bar: 50 *µm*. F) Statistical analysis of green fluorescence intensity from CLSM 3D images using image‐J software for *Xoo* live cell count and biofilm eradication (n = 3). G) Statistical analysis of red fluorescence intensity using image‐J software for *Xoo* dead cell count and biofilm eradication in CLSM 3D images (n = 3). H) SEM imagines showing the inhibitory effects of BtP27 and BtP27@*β*‐CD on *Xoo* survival and their eradicative impact on pre‐established biofilm at various concentrations. I) Photographs of hypersensitive response in the non‐host plant *nicotiana benthamiana* after exposure to water, BtP27, and BtP27@*β*‐CD. J) The expression levels of T3SS‐related genes in *Xoo* following treatment with BtP27 and BtP27@*β*‐CD, analyzed by qRT‐PCR (n = 3). *gyrB* was used as the reference gene. K) Statistical analysis of leaf lesion lengths and control efficiencies from the pathogenicity experiment (n = 9). L) Representative pathogenic rice leaves treated with BtP27 and BtP27@*β*‐CD via leaf‐clipping inoculation method. Statistically significant differences between the means were analyzed with one‐way ANOVA, followed by the least significant difference (LSD) post‐hoc test in (B‐D, F, G, J, K) (For all studies, n ≥ 3; *p < 0.05, **p < 0.01, ***p < 0.001; n. s. = no significance).

Despite extensive research on the prevention of biofilm formation, the eradication of mature biofilms remains a formidable challenge.^[^ ^52^, ^53^ ^]^ Encouraged by the promising initial findings, we proceeded to evaluate the efficacy of BtP27 and BtP27@*β*‐CD in eradicating pre‐established *Xoo* biofilms. *Xoo* was cultured for 48 h to allow biofilm maturation, after which they were co‐cultured with above molecules for an additional 24 h, followed by quantification of the biofilms using crystal violet staining (**Figure** **6**A–D). Unlike *β*‐CD, which exhibited no effect on bacterial growth and biofilm elimination even at concentrations up to 348.00 µg mL^−1^ (400 × EC_50_), both the supramolecular and guest molecules commenced biofilm eradication at a concentration of 13.92 µg mL^−1^ (16 × EC_50_), with eradication rates of 4.77% and 4.56%, respectively. The eradication effect intensified in a dose‐dependent manner with increasing concentrations (ranging from 13.92 to 348.00 µg mL^−1^), with near‐complete eradication achieved at a concentration of 174.00 µg mL^−1^ (200 × EC_50_, Figure S11B, Supporting Information). Notably, both entities exhibited bactericidal effects within mature biofilms at concentrations as low as 13.92 µg mL^−1^ (16 × EC_50_) (Figure 6C,D).

The eradication of mature biofilms and the bactericidal activity against bacteria embedded within the biofilm were further corroborated by AO and PI staining, where escalating concentrations resulted in a reduction in green fluorescence and an elevation in red fluorescence, signifying a dose‐dependent eradication of mature biofilms and annihilation of the biofilm‐embedded cells (Figure 6E–G). At an elevated concentration of 174.00 µg mL^−1^ (200 × EC_50_), the relative green fluorescence intensities for BtP27 and BtP27@*β*‐CD declined to 4.80% and 3.15%, respectively, whereas the relative red fluorescence intensities surged to 77.44% and 80.43%, respectively. These results indicate that at this concentration, a substantial fraction of the mature biofilm was eradicated, and a large proportion of bacteria embedded within the biofilm were effectively eliminated. SEM images provided a visual illustration of the reduction in mature biofilm density and bacterial population upon treatment with BtP27 and BtP27@*β*‐CD, along with a disturbance in bacterial survival (Figure 6H). These findings underscore the potential of these molecules not only to inhibit biofilm formation but also to effectively eradicate mature biofilms and annihilate the bacteria encased within them.

### 2.9 Potential Mechanisms Underlying Biofilm Disruption and Bactericidal Action

*Xoo* produces a distinct extracellular polysaccharide (EPS) regulated by the *gum* gene cluster, essential for biofilm formation and *Xoo* chemotaxis.^[^ ^33^, ^34^ ^]^ To delve into the mechanism through which BtP27 and BtP27@*β*‐CD target biofilms and induce disruptive effects, we investigated EPS production using the standard gravimetric method. As shown in Figure S12 (Supporting Information), compared to the control, BtP27@*β*‐CD reduced EPS yields to 45.32% and 24.99% at 0.87 µg mL^−1^ (1 × EC_50_) and 1.74 µg mL^−1^ (2 × EC_50_), respectively, versus 60.00% and 39.98% for BtP27. These results indicate that BtP27@*β*‐CD more effectively inhibits EPS production. Subsequently, we performed qRT‐PCR to evaluate the expression levels of *gum* gene cluster in *Xoo* following treatment with these molecules. At 0.87 µg mL^−1^ (1.0×EC_50_), BtP27@*β*‐CD downregulated *gum B*, *gum D*, *gum I*, and *gum J* by 58.01%, 65.06%, 54.93%, and 87.74%, respectively, surpassing the individual guest molecule across all subtypes (Figure S13, Supporting Information). Notably, neither the individual guest molecule nor the supramolecular material affected *Xoo* cell growth at 1.74 µg mL^−1^ (2 × EC_50_). Thus, both entities effectively inhibit EPS production by suppressing *gum* gene cluster expression while preserv statistical analysising cell viability.

Once supramolecular nanoparticles penetrate the biofilm barrier, they must efficiently eliminate the embedded bacteria to achieve bacteric6dal activity. Extensive studies have reported that exogenous chemicals, particularly those containing benzothiazole and *α*‐aminophosphate structures, induce reactive oxygen species (ROS) production in pathogens.^[^ ^54^ ^]^ ROS are known to disrupt bacterial structural integrity and irreversibly damage DNA via oxygen radical attacks, ultimately leading to pathogen death.^[^ ^55^, ^56^ ^]^ Consequently, we examined ROS generation in *Xoo* upon treatment with BtP27 and BtP27@*β*‐CD using fluorescence analysis. The two molecules differentially induce ROS production in *Xoo*, where the supramolecular material elicits a greater ROS response than the guest molecule alone, as evidenced by increased fluorescence intensity and peak emission (Figure S14, Supporting Information), which is fundamentally consistent with the observed differences in their in vitro activities. Thus, ROS generation likely constitutes the primary bactericidal mechanism of both entities,^[^ ^57^, ^58^ ^]^ with the underlying cause of ROS accumulation potentially linked to their ability to inhibit catalase (CAT) and superoxide dismutase (SOD) enzymatic activities (Figure S15, Supporting Information).

### 2.10 BtP27@*β*‐CD‐Mediated Attenuation of Pathogenicity via T3SS Targeting

In a validation experiment for T3SS intervention, treatment of *Xoo* with BtP27 and BtP27@*β*‐CD at a concentration of 0.87 µg mL^−1^ (1 × EC_50_), which did not affect bacterial growth, attenuated the hypersensitive response in the non‐host plant *nicotiana benthamiana* (Figure 6I). This observation highlights the potent virulence inhibition exerted by both the guest molecule and the supramolecular material, suggesting a potential link to T3SS. Further qRT‐PCR analysis revealed that BtP27 and BtP27@*β*‐CD downregulated key T3SS‐related genes associated with the hypersensitive response, specifically *HrcT*, *HrpE*, *HrpF*, and *HrpG*, with expression reductions ranging from 12.57% to 59.42% (Figure 6J; Tables S11 and S12, Supporting Information). These results demonstrate that both the functional molecule and supramolecular material mitigate pathogenicity by targeting T3SS. Subsequent pathogenicity assays confirmed the inhibitory effects of the two molecules on pathogenicity under physiological conditions (Figure 6K,L). Treatment with BtP27 and BtP27@*β*‐CD alleviated disease symptoms on rice leaves, with the average lesion length shortened by 10.98 and 8.33 cm, respectively, in comparison to the control. The inactivation efficiencies of 56.00% and 42.47%, respectively, indicate a mitigation in pathogenicity.

### 2.11 Universality of the Supramolecular System for Diverse Substrates and Diseases

**Figure 7:**
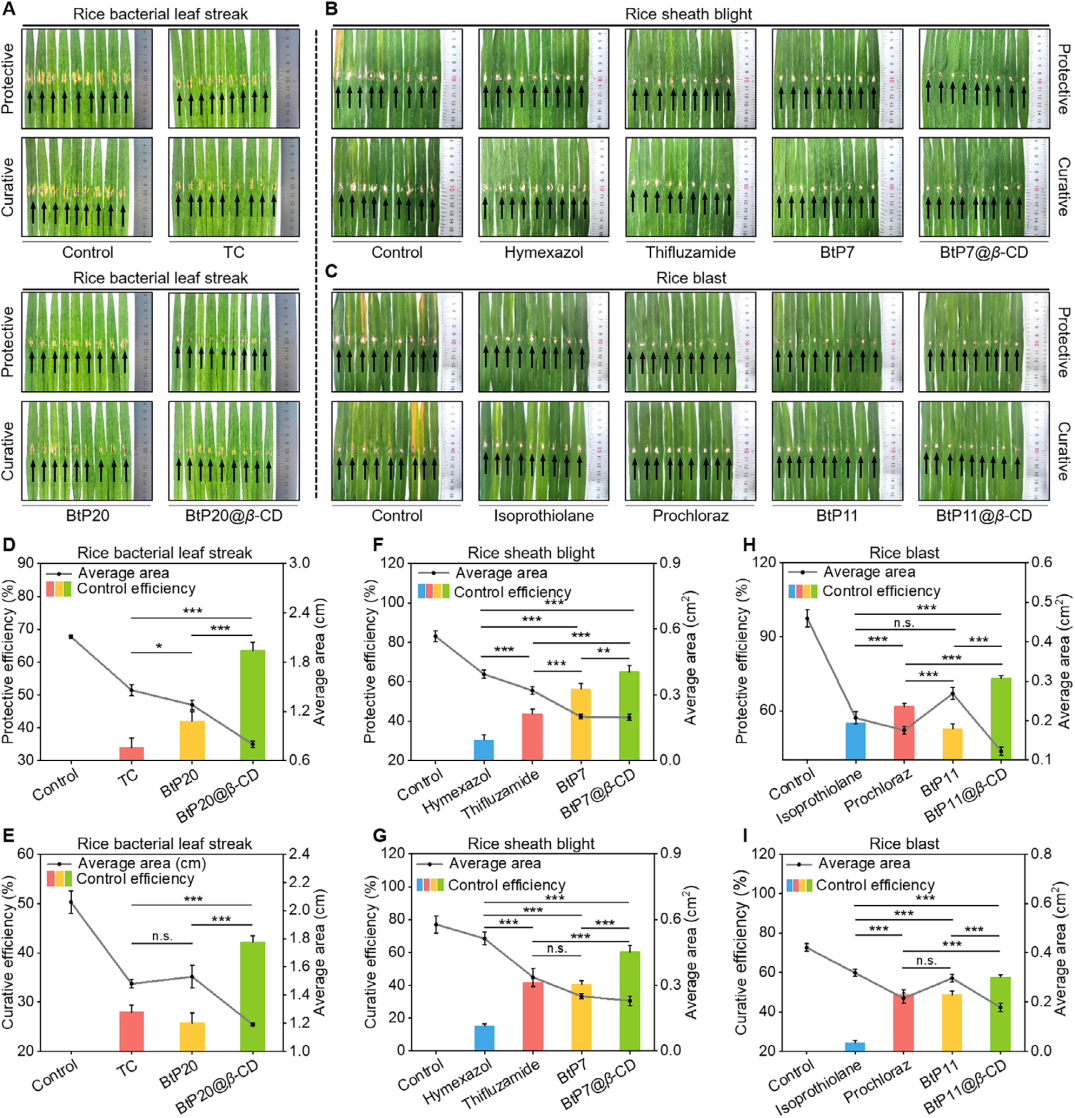
In vivo antimicrobial potency of BtP7, BtP11 and BtP20, alongside their respective derived supramolecular materials. A) Photographic documentation of control efficacies of TC, BtP20, and BtP20@*β*‐CD against *Xoc*‐induced rice bacterial leaf streak at 200 µg mL^−1^. B) Photographic documentation of control efficacies of hymexazol, thifluzamide, BtP7, and BtP7@*β*‐CD against *Rs*‐induced rice sheath blight at 200 µg mL^−1^. C) Photographic documentation of control efficacies of isoprothiolane, prochloraz, BtP11, and BtP11@*β*‐CD against *Mo*‐induced rice blast at 200 µg mL^−1^. D,E) Protective (D) and curative (E) efficiencies as determined from (A). F,G) Protective (F) and curative (G) efficiencies as determined from (B). H,I) Protective (H) and curative (I) efficiencies as determined from (C). Statistically significant differences between the means were analyzed with one‐way ANOVA, followed by the least significant difference (LSD) post‐hoc test in (D‐I) (For all studies, n = 9/group; *p < 0.05, **p < 0.01, ***p < 0.001; n.s. = no significance).

Given the impressive therapeutic effects of BtP27 and BtP27@*β*‐CD on bacterial leaf blight (BLB) and their unique mode of action in interfering with both biofilm and T3SS, we expanded our investigation to include their analogs and corresponding supramolecular materials for antibacterial potency against a range of rice diseases. These included *Xoc* (*Xanthomonas oryzae* pv. *oryzicola*)‐induced bacterial leaf streak (BLS), fungal *Rs* (*Rhizoctonia solani*)‐induced rice sheath blight, and *Mo* (*Magnaporthe oryzae*)‐induced fungal rice blast, aiming to broaden the substrate scope and antibacterial applicability of these supramolecular materials.^[^ ^59^ ^]^ Prior to in vivo experiments, we assessed the antibacterial potency of these potential guest molecules (BtP1‐30) against *Xoc*, *Rs*, and *Mo*. Among them, compounds BtP20, BtP7, and BtP11 displayed the highest efficacy against their respective (Tables S13 and S14, Supporting Information), making them promising candidates for in vivo efficacy studies of their derived supramolecular materials. Following the preparation method of BtP27@*β*‐CD, we fabricated supramolecular materials from these selected molecules and confirmed their successful formation using HRMS (Figures S17–S19, Supporting Information). This indicates a certain versatility in the substrate scope for this plant protection supramolecular system. For BLS caused by *Xoc*, the control group exhibited severe disease symptoms, whereas treatment with BtP20@*β*‐CD at a concentration of 200 µg mL^−1^ reduced the lesion area on rice leaves (**Figure** **7**A,D,E; Figure S23, Supporting Information), achieving protective efficacy of 63.53% and curative efficacy of 42.18%. These results exceeded those of the guest molecule alone (protective activity: 42.14%, curative activity: 25.79%) and the commercial fungicide TC (protective activity: 34.01%, curative activity: 27.95%) (Table S15, Supporting Information).

For rice sheath blight (Figure 7B,F,G; Figure S24, Supporting Information) and blast (Figure 7C,H,I; Figure S25, Supporting Information), both caused by fungal infections, the supramolecular materials also effectively alleviated disease symptoms. The protective and curative efficacies of BtP7@*β*‐CD against rice sheath blight were 65.09% and 60.41%, respectively, which were superior to those of the functional molecule BtP7 (protective efficiency: 56.45%, curative efficiency:40.58%) and the commercial antifungal agents hymexazole (protective efficiency: 30.45%, curative efficiency:15.25%) and thifluzamide (protective efficiency: 43.65%, curative efficiency:41.77%) (Table S16, Supporting Information). In addition, the protective and therapeutic effects of BtP11@*β*‐CD on rice blast disease were 73.30% and 57.67%, respectively, outperforming those of the functional molecule BtP11 (protective efficiency: 52.62%, curative efficiency:48.69%) and the commercial antifungal agents isoprothiolane (protective efficiency: 54.95%, curative efficiency: 24.40%) and prochloraz (protective efficiency: 61.77%, curative efficiency: 48.81%) (Table S17, Supporting Information). These findings demonstrate the broad‐spectrum efficacy of these supramolecular materials against not only bacterial diseases but also fungal infections, highlighting their potential in comprehensive plant protection strategies.

## 3 Conclusion

Herein, we present an innovative multifunctional material (BtP27@*β*‐CD) fabricated via supramolecular engineering, emanating from the self‐assembly of the dufulin‐derived guest molecule BtP27 and the host molecule *β*‐CD, orchestrated by hydrophilic and hydrophobic forces. The resultant multifunctional displays a sunflower‐shaped material morphology, characterized by low surface tension and small contact angle, which facilitates its optimal integration with the microstructures on superhydrophobic rice leaves, thereby enhancing droplet deposition and retention. The superior foliar affinity exhibited by BtP27@*β*‐CD, unattainable by conventional antimicrobials, effectively translates its notable in vitro activity into high in vivo efficacy against bacterial leaf blight, outperforming both individual BtP27 and the registered TC. Furthermore, BtP27@*β*‐CD inherits the superior uptake and translocation characteristics of dufulin, alongside its capacity to activate salicylic acid‐mediated plant defense pathways, thereby ensuring its protective efficacy throughout the entire leaf. Importantly, it delivers its efficacy in vivo without compromising safety, as demonstrated by the absence of obvious toxicity in both target and non‐target organisms. Mechanistic investigations elucidate that the integration of isopropanolamine confers BtP27@*β*‐CD with robust biofilm disruption capabilities, evidenced by its inhibition of biofilm formation and eradication of established biofilms in a dose‐dependent fashion. This finding represents a paradigm shift in the action mechanism of dufulin analogues, shifting from antiviral to antimicrobial effectiveness by specifically targeting biofilms. Further mechanistic insights have shown that the BtP27@*β*‐CD also impedes T3SS functionality, as demonstrated by qRT‐PCR analysis indicating the downregulation of pivotal T3SS‐associated genes, such as *HrcT*, *HrpE*, *HrpF*, and *HrpG*. This represents the inaugural report of a dual‐targeted inhibitor that addresses both biofilms and T3SS through a supramolecular strategy.

Furthermore, additional dufulin derivatives, specifically BtP7, BtP11, and BtP20, have also demonstrated the capacity to self‐assemble with *β*‐CD in aqueous environments, forming supramolecular materials as confirmed by high‐resolution mass spectrometry (HRMS). These materials exhibit superior in vivo efficacy against a spectrum of diseases, including fungal infections like rice sheath blight and blast, alongside bacterial maladies such as rice leaf streak. Notably, their protective efficacy fluctuates between 63.53% and 73.30%, whereas their therapeutic efficacy varies from 42.18% to 60.41%, consistently outperforming their respective guest molecules and commercial counterparts. These findings indicate that this class of plant‐protecting supramolecular systems exhibits a degree of substrate and disease universality, imparting valuable insights for the development of other multifunctional materials in plant disease control and potential broader applications.

Crucially, the guest molecules within these supramolecular complexes are autonomously engineered, allowing for modulation over their structures, properties, and mechanisms of action through structural modification. This ensures efficient and streamlined synthesis and endows the materials with advantageous properties and specific antibacterial mechanisms. Meanwhile, the preparation of these guest molecules and their subsequent assembly into supramolecular materials eschew the excessive use of additives and intricate processing procedures, thereby mitigating the risks that traditional additives present to already vulnerable ecosystems, food safety, and human health. In summary, this class of supramolecular systems for plant disease control holds considerable potential, presenting multiple advantages in terms of tailorable properties, cost‐effectiveness, eco‐friendliness, and environmental sustainability.

## 4 Experimental Section

### Uptake and Translocation Characteristics

BtP27@*β*‐CD, BtP27, and *β*‐CD were formulated at a concentration of 200 µg mL^−1^. These formulations were subsequently adhered to filter papers (1 cm × 1 cm) and affixed centrally on the rice leaves. After 24 h, the leaves were inoculated with *Xoo* (OD_595_ = 0.1) via a leaf‐cutting method, 2 cm away from the application area, ensuring the leaves were not completely severed. All treated plants were then incubated in an artificial climate chamber maintained at 28 °C and 90% relative humidity for 10 days. Post incubation, the lesion lengths were measured, and the prevention efficacy was evaluated. The control efficiency (*I*) of *Xoo* was calculated using the following equation:

(1) $$\mathrm{Control}\;\mathrm{efficiency}\;I\;\left(\%\right)=\left(C-T\right)/C\times 100$$

where *C* represents the lesion length of the control group, and *T* denotes the lesion length of the treatment group.

### Evaluation of Biofilm Formation Inhibition

*Xoo* cells, initially cultivated in NB liquid medium with an OD_595_ of 0.6, were diluted to an OD_595_ of 0.1. Subsequently, 200 µL of the bacterial suspension was aliquoted into a 96‐well plate, followed by the addition of varying concentrations of BtP27, BtP27@*β*‐CD, and *β*‐CD through a serial half‐dilution technique. The plate was incubated at 28 °C for 48 h. Post‐incubation, the suspended *Xoo* cells were meticulously removed and rinsed twice with sterile water. The plate was then air‐dried at 25 °C for 4 h. Each well received 200 µL of a 0.1% crystal violet solution, allowing for a 30‐minute staining period. Excess crystal violet was discarded, and the wells were rinsed thrice with sterile water. The plate was subsequently dried in an oven at 40 °C for 1 h, after which the crystal violet was solubilized with a 95% ethanol solution. The biofilm content was quantified by measuring OD_570_ nm using a microplate reader.

### Eradication Assessment of Mature Biofilm

A 200 µL suspension of *Xoo* bacteria at an OD_595_ of 0.1 was introduced into 96‐well plates and incubated for 48 h to facilitate mature biofilm development. Following this incubation, the medium was aspirated, and the planktonic bacteria were carefully rinsed twice with PBS buffer (pH 7.4, 10 mM). Subsequently, 200 µL of medium containing various concentrations of BtP27@*β*‐CD, BtP27, and *β*‐CD was added to the wells, with the plates incubated at 28 °C for an additional 24 h. Their effects in eradicating mature biofilms were subsequently assessed through crystal violet staining.

### Hypersensitivity Assay

*Xoo* cells were inoculated into the medium and incubated overnight. The bacterial solution was subsequently adjusted to an OD_595_ of 0.1 using sterile distilled water. Sterile centrifuge tubes containing 1.0 mL of *Xoo* cells were then supplemented with BtP27@*β*‐CD, BtP27, and a control solution containing 0.4% DMSO, all at a dose of 1.0 EC_50_. These tubes were incubated in a shaker at 28 °C for 4 h. Thereafter, 200 µL of the bacterial solution was aspirated with a 1.0 mL needleless syringe and injected into *N. benthamiana* via the osmotic pressure method, with water serving as a blank control. The samples were then incubated at 28 °C for 48 h, after which hypersensitive response (HR) symptoms were observed and documented.

## Conflict of Interest

The authors declare no conflict of interest.

## Author Contributions

X.M. and K.L. contributed equally to this work. P.W. designed the study. X.M., K.L., J.Y, and J.L. performed the experiments. X.M. and K.L. analyzed the data. P.W. checked up the data. X.M. wrote the original draft. K.L., F.D., G.H., and P.W. reviewed and edited the manuscript. The authors read and approved the final manuscript.

## Supporting information



Supporting Information

Supplemental Video 1

Supplemental Video 2

Supporting Information

Supporting Information

## Data Availability

The data that support the findings of this study are available in the supplementary material of this article.
