# Supplementary material for: From De Novo Conceived Small Molecules to Multifunctional Supramolecular Nanoparticles: Dual Biofilm and T3SS Intervention, Enhanced Foliar Affinity, and Effective Rice Disease Control
Source: Adv Sci (Weinh). 2025 Mar 27;12(20):2410878. doi: 10.1002/advs.202410878 (PMC12120793; doi:10.1002/advs.202410878)
Supplement: Supplementary file 1 — Supporting Information [file ADVS-12-2410878-s003.docx]

Supporting Information

**From *De Novo* Conceived Small Molecules to Multifunctional Supramolecular Nanoparticles: Dual Biofilm and T3SS Intervention, Enhanced Foliar Affinity, and Effective Rice Disease Control**

*Xianfu Mu^1†^, Kongjun Liu**^1,2†*^, Jinghan Yang^1^, Juan Liu^1^, Fengpei Du^3^, Gefei Hao^1^and Peiyi Wang^1*^*

X. Mu, K. Liu, J. Yang, J. Liu, Prof. F. Du, Prof. G. Hao, Prof. P. Wang

^1^State Key Laboratory of Green Pesticide, Key Laboratory of Green Pesticide and Agricultural Bioengineering, Ministry of Education, Center for Research and Development of Fine Chemicals of Guizhou University, Guiyang, 550025, China.

^2^Key Laboratory of Basic Pharmacology of Ministry of Education and Joint International Research Laboratory of Ethnomedicine of Ministry of Education, Zunyi Medical University, Zunyi, 563006, China.

^3^Department of Applied Chemistry, College of Science, China Agricultural University, Beijing, 100193, China.

^*^Corresponding author E-mail: pywang888@126.com; pywang@gzu.edu.cn (P.-Y. Wang); jkrmanai@163.com (K.-J. Liu).

^†^The two authors contribute equally to this work.

**Contents**

[1. Chemicals and instrument 3](#_Toc174120668)

[2. Experimental section 3](#_Toc174120669)

[3. Supplementary Figures and Tables 20](#_Toc174120670)

[4. Characterization of target compounds 57](#_Toc174120671)

[5. ^1^H NMR, ^13^C NMR, ^31^P NMR, ^19^F NMR, and HRMS Spectra of BtP1- BtP30. 73](#_Toc174120672)

[6. References 139](#_Toc174120673)

# 1. Chemicals and instrument

Commercially sourced solvents and reagents were employed without further purification unless specified otherwise. The ^1^H NMR, ^13^C NMR, ^31^P NMR, and ^19^F NMR spectra were recorded using CDCl_3_ or D_2_O as the solvent, with TMS as the internal standard, on either a JEOL-ECX500 spectrometer or a Bruker Biospin-AG-400 instrument. High-resolution mass spectrometry was performed on a UitiMate 3000 (Thermo Scientific) system. Ultraviolet-visible (UV-vis) spectra were measured using a UV–2335 spectrophotometer (Uniko (Shanghai, China) Instrument Co., Ltd). Contact angle measurements were conducted with a JC-2-2000D1 apparatus (Shanghai Zhongchen Digital Technic Apparatus Co., Ltd., Shanghai, China). Scanning electron microscopy (SEM) images were acquired on a FEI Nova microscope. The Zeta potential was assessed using a DelsaNanoC analyzer (Beckman Coulter, Inc., USA). *In vitro* antibacterial activity was quantified by monitoring the OD value at 595 nm with Cytation™ 5 multimode readers (BioTek Instruments, Inc., USA). The primers were obtained from Beijing Qingke Biotechnology Co., Ltd. and Wuhan GenScript Biotech Co., Ltd. The superoxide dismutase (SOD) and catalase (CAT) kits were purchased from Beijing Solarbio Science & Technology Co., Ltd. The reactive oxygen species detection kit was purchased from Beyotime Biotechnology. The RNA extraction kit and qPCR SYBR Green Master Mix were purchased from Yeasen Biotechnology (Shanghai) Co., Ltd. The reverse transcription kit, FastKing gDNA Dispelling RT SuperMix, was purchased from Tiangen Biotech (Beijing) Co., Ltd. RNAiso Plus is a product of Takara. High-speed impact experiments were captured using the i-SPEED 220 camera. Three-dimensional imaging was performed with a Nikon A1R Confocal Microscope System (Nikon Instruments Inc., Melville, NY, USA).

# 2. Experimental section

**2.1 Synthesis of target compounds BtP1-BtP30**

**Preparation of intermediate 1**. A 15 mL pressure-resistant vial was charged with 4-methylbenzothiazole (3.04 mmol), *p*-hydroxybenzaldehyde (3.04 mmol), and diethyl phosphite (3.65 mmol), followed by stirring at 80 °C for 14 hours. Upon completion of the reaction, as monitored by TLC, the mixture was extracted with dichloromethane (30 mL). The solvent was then removed under reduced pressure, and the resulting crude product was recrystallized, yielding a white solid with a 65% yield.

**Preparation of intermediate 2.** Intermediate 1 (0.25 mmol), KOH (0.30 mmol), and epoxybromopropane (0.50 mmol) were introduced into a 25 mL round-bottom flask and stirred at room temperature for 7 hours. Following the reaction, the mixture was extracted with ethyl acetate (30 mL), the organic phase was evaporated to dryness, and the residue was purified via column chromatography using a CH_2_Cl_2_:CH_3_OH (30:1, v/v) eluent system, affording a light yellow solid with a yield of 78%.

**Preparation of target compounds (BtP1-BtP30).** Intermediate 2 (0.67 mmol), corresponding amine (1.0 mmol), and K_2_CO_3_ (0.67 mmol) were dissolved in 3 mL of isopropanol and transferred to a 15 mL reaction vial. The mixture was stirred at 50 °C for 6 hours with TLC monitoring the reaction progress. Upon completion, the reaction was quenched by the addition of water, and the mixture was extracted with ethyl acetate (50 mL). The organic phase was separated, dried over anhydrous Na_2_SO_4_, and concentrated under reduced pressure. The residue was purified by column chromatography using CH_2_Cl_2_: CH_3_OH (30:1, v/v) as the eluent, yielding the target compounds. All structures were confirmed by NMR and HRMS spectra.

**2.2 *In vitro* bioassay**

*Xanthomonas oryzae* pv*. oryzae (Xoo), Xanthomonas oryzae* pv*. oryzicola (Xoc), Rhizoctonia solani (Rs), and Magnaporthe oryzae (Mo)* were employed for both *in vivo* and *in vitro* experimental assessments. BtP27@*β*-CD and BtP27 were initially dissolved in DMSO to a concentration of 50 *μ*g *μ*L^-1^ for subsequent investigates. Unless otherwise specified, all experimental solutions were derived by diluting this stock solution with water. Furthermore, for both *in vivo* and *in vitro* activity studies, 0.1% Tween-20 was incorporated as a solubilizing agent in addition to the stock solution dilution.

**Antibacterial potency.** The *in vitro* turbidimetric method was employed to assess the antibacterial efficacy of small molecules BtP1-BtP30 against the phytopathogenic strains *Xoo* and *Xoc*. These molecules were individually introduced into 4.0 mL of NB liquid medium, which comprised 1.5 g of beef extract, 2.5 g of peptone, 0.5 g of yeast powder, 5.0 g of glucose, and 500 mL of distilled water, adjusted to a pH of 7.2. Starting at a concentration of 50 *μ*g mL^-1^, the compounds were serially diluted by a factor of two. Subsequently, 40 μL of bacterial suspension (OD_595_ = 0.6) were added to each test tube. The inoculated tubes were incubated at 28 ± 1 °C with continuous shaking at 180 rpm for 24 to 48 hours until the control group bacteria reached the logarithmic growth phase. Following incubation, 200 μL samples were transferred to 96-well plates, and the OD_595_ was measured using an enzyme-linked immunosorbent assay reader. Dimethyl sulfoxide served as the blank control, while thiodiazole-copper (TC) was utilized as the positive control. All test tubes were monitored at OD_595 (turbidity-corrected values)_ = OD_bacterial wilt_ − OD_no bacterial wilt_, and the inhibition rate *I* was determined using the equation:

$$\text{I}\text{ (\%)=}\frac{C-T}{C}\text{×100}$$

where *C* represents the corrected turbidity values of bacterial growth in untreated NB (blank control) and *T* denotes the corrected turbidity values of bacterial growth in treated NB. Drug concentration data were log-transformed (log(x)), and inhibition rate data were converted to odds (y). The toxicity regression equation (y = ax+b) and correlation coefficient (R^2^) were derived using Excel calculations to determine the half-maximal effective concentration (EC_50_).

**Antifungal potency.** The *in vitro* hyphal growth rate method was employed to evaluate the antifungal activity of functional molecules BtP1-BtP30 against phytopathogenic strains *Rs* and *Mo*. A quantity of 0.6 mg of each functional molecule was dissolved in 0.6 mL of DMSO, followed by the addition of 5.4 mL of Tween 20 aqueous solution, resulting in a total volume of 6 mL. This solution was then incorporated into the melted PDA medium, consisting of 20 g of glucose, 200 g of potato extract, 20 g of agar, and 1000 mL of distilled water. The mixture was thoroughly homogenized, poured into petri dishes, and allowed to cool and solidify. In a sterile environment, a 5 mm perforator was sterilized and used to create inoculum cakes from fungal colonies, which were subsequently placed at the center of the PDA medium. The plates were incubated at 25-28 °C for approximately 5 days until the colonies in the blank control reached a diameter of approximately 6 cm. Colony diameters were measured twice using the cross method and averaged to determine the size. Initially, a screening concentration of 100 *µ*g mL^-1^ was employed, and the mycelial growth inhibition rate was calculated using the following formula:

$$I\left( \% \right)=\frac{C-T}{C-0.5}\times100$$

where 0.5 (cm) is the diameter of the inoculum cake, *C* represents the diameter of the control colony, and *T* is the diameter of the colony in the treatment group. Hymexazol, thifluzamide, isoprothiolane, and prochloraz were used as control agents in the assay. Each component was tested in triplicate to ensure accuracy and reproducibility of the results.

**2.3 3D-QSAR model**

Given the potent antimicrobial activity demonstrated by functional molecules BtP1-BtP30, 3D-QSAR studies employing comparative molecular field analysis (CoMFA) and comparative molecular similarity index analysis (CoMSIA) modeling were conducted to elucidate conformational relationships and inform future structural design. ^[1]^ Initially, Schrödinger's LigPrep was utilized to prepare the 3D structures of the functional molecules BtP1-BtP30. The force field employed was OPLS4, maintaining the ionization state unchanged, and generating all possible isomers. Ligand Alignment facilitated the conformational superposition, ConfGen was used for ligand conformational search, and the generated conformations were sequentially superimposed with the reference ligand. The best conformations matching the reference ligand were obtained, with the superposition of the largest common backbone performed using the Largest Common Bemis-Murcko scaffold method. For the 3D-QSAR field-based modeling, 75% of the functional molecules BtP1-BtP30 were randomly allocated to the training set and 25% to the test set. The *Xoo*-resistant EC_50_ values served as the modeling data to establish the CoMSIA and CoMFA field models.

**2.4 Characterization of BtP27@*β*-CD**

**2.4.1 Characterization of morphology**

BtP27@*β*-CD and BtP27 were prepared at a concentration of 200 *µ*g mL^-1^. A 20 *μ*L aliquot was pipetted onto conductive glass, allowed to air-dry naturally, and then coated with a thin layer of gold for scanning electron microscopy (SEM) microstructure observation. Separately, BtP27@*β*-CD and BtP27were also prepared at a concentration of 200 *µ*g mL^-1^, and 20 *μ*L droplets were deposited onto a copper mesh. These droplets were air-dried and subsequently stained with a 1% phosphotungstic acid solution for 30 seconds. The excess solution was blotted with filter paper, the samples were air-dried, and then visualized by transmission electron microscopy (TEM).

**2.4.2 Critical Micelle Concentration (CMC) Assay**

A stock solution was prepared at concentrations of 0.5 and 1 *µ*g mL^-1^. Aliquots of this solution were serially diluted in 1.5 mL of water to generate a range of concentrations: 6.25, 3.125, 1.56, 0.78, 0.3906, 0.1953, 0.0976, 0.04882, and 0 *µ*g mL^-1^. Each diluted sample was subsequently exposed to infrared light, and images were captured for documentation. Surface tension measurements were subsequently performed using the drop method. The resulting data were fitted to a curve, allowing for the identification of the inflection point, which corresponds to the critical micelle concentration (CMC).

**2.4.3 Particle size and Zeta potential determination**

The BtP27 and BtP27@*β*-CD were dissolved to a concentration of 200 *µ*g mL^-1^. Particle size and Zeta potential of these solutions were measured using a dynamic light scattering (DLS) instrument.

**2.4.4 Phase solubility analysis**

Introduce 1 mL of water and precisely 0.2 mg of BtP27 into each of six 10 mL volumetric flasks. Subsequently, add *β*-CD solutions with concentrations of 0, 10, 20, 30, 40, and 50 *μ*M to the respective flasks, ensuring the final volume remains constant. Incubate the flasks in a shaker set to 25 °C and 180 rpm for 72 hours to achieve equilibrium dissolution. Filter the resulting solutions through a 0.22 *μ*m microporous membrane, perform appropriate dilutions, and measure the absorbance using a UV-Vis spectrophotometer. Construct the solubility curve by plotting the concentration of dissolved BtP27 against the *β*-CD concentration.

**2.4.5 UV-vis titration experiment**

The UV-vis spectrum was obtained using a 3.0 mL colorimetric dish containing a 40 *μ*M aqueous solution of BtP27. A 50 mM aqueous solution of *β*-CD was prepared and incrementally added to the colorimetric dish for the UV-visible spectrum analysis. The binding constant (*K*_a_) was determined using the Benesi-Hildebrand (B-H) equation:

$$\frac{\text{1}}{\text{Δ}\text{A}}\text{=}\frac{\text{1}}{\text{α}\text{×}\text{K}\text{a}}\text{×}\frac{\text{1}}{\text{c}\text{(}\text{β}\text{-CD)}}\text{+}\frac{\text{1}}{\text{α}}$$

where *ΔA* represents the difference in absorbance values before and after inclusion, *c* denotes the total concentration of *β*-CD, and *α* is a constant. To obtain Ka, plot 1/*ΔA* against 1/*c*(*β*-CD) and calculate the slope and intercept of the resulting linear plot.

**2.4.6 Job's plot experiment**

Several solutions of BtP27 and *β*-CD were prepared, each with a total concentration of 80 μM in a 3.0 mL volume, varying in molar ratios. In this process, BtP27 was present in varying molar fractions (x-axis). The difference in UV absorbance (ΔA) was measured for each concentration, both with and without BtP27. Job's curve was then generated by plotting ΔA(y-axis) against the molar ratio N*β*_-CD_:N_[BtP27+_*β*_-CD]_.

**2.4.7 NMR Spectra**

The assembly behavior and driving forces of BtP27 and *β*-CD in D_2_O were investigated using ^1^H NMR spectroscopy. For this analysis, solutions containing 4.0 mM of BtP27, *β*-CD, and their inclusion complexes were prepared in varying molar ratios (1:0, 1:0.3, 1:0.6, 1:1, and 1:2) and characterized by ^1^H NMR to assess the interactions and encapsulation position.

**2.4.8 Molecular docking of *β*-CD and BtP27**

BtP27 and *β*-CD were imported into AutoDock, where they were processed and optimized after adding hydrogen atoms. ^[2]^ The appropriate grid size was set, and the parameters were saved as a gpf file. Subsequently, the command code was entered to initiate the docking simulation.

**2.4.9 Molecular dynamics simulations of BtP27@*β*-CD**

Molecular dynamics (MD) simulations were performed using the GROMACS 2023.3 software package, employing the GAFF force field parameters.

**Structure Preparation**. The source of BtP27 was synthesized by our group, while *β*-CD was purchased from Energy Chemical. The supramolecular binary building block BtP27@*β*-CD was simulated using AutoDock (2.6). The topology files are constructed using the ACPYPE software^[3]^. The atomic charges were computed using the Restrained Electrostatics Potential (RESP) charge, calculated at the B3LYP/6-311G** level in all structures by ORCA software ^[4]^ and Mutifwn software^[5]^. This initial structure, consisting of BtP27, *β*-CD, and Tip3p water, was generated by the package software in a cube box of 10 nm × 10 nm × 10 nm^[6]^.

**EM.** This initial structure were then energetically minimized using the steepest descent and conjugate gradient techniques until the minimization converged with the maximum force of less than 10 kJ/mol.

**NVT.** To obtain an equilibrated structure, the systems were heated for 100 ps at 300 K in an NVT ensemble using periodic boundary conditions. A 2 fs step size was used, with all covalent bonds to hydrogen atoms held rigid using the LINCS approach. The temperature was maintained at 300 K using the V-rescale^[7]^. The electrostatic interactions were cut off at 1.4 nm using the Particle-Mesh-Ewald (PME) method^[8]^, and van der Waals interactions were also cut off at 1.4 nm.

**NPT.** A short equilibration of 100 ps followed, with a production run using a 2 fs step size in an NPT ensemble with the same protocols. The pressure was coupled with the Parrinello-Rahman barostat at 1.0 atm^[9]^.

**MD.** Finally, under the conditions of 300K and 1 atm, the system was simulated for 100ns.

**Analysis.** Molecular dynamics simulation trajectories, including hydrogen bonding and root-mean-square deviation (RMSD), were analyzed using functions such as gmx_hbond and gmx_rmsd from the GROMACS package. The system and the trajectories were visualized by VMD (version 1.9.3).

**2.5 Foliar affinity evaluation of BtPp27@*β*-CD**

**2.5.1 Determination of surface tension, contact angle, and droplet pull-up height**

BtP27@*β*-CD, BtP27, and *β*-CD were prepared at a concentration of 200 *μ*g mL^-1^. Pristine rice leaves were affixed onto slides for subsequent analysis. The surface tension of the various sample droplets was quantified utilizing the pendant drop method via a JC-2000D1 contact angle meter, with each sample subjected to at least three replicates. Following this, contact angle measurements were undertaken to evaluate droplet spread on the rice leaf surfaces using goniometric method, ensuring a minimum of three replicates per sample. A calibrated flat-head needle with an 0.8 mm diameter was employed to elevate the component droplets from the rice leaves, with the heights recorded and photographed. Each sample was measured at least three times to ensure statistical robustness.

**2.5.2 Liquid holding capacities assay**

Discs, each 1 cm in diameter, were excised from rice leaves using a perforator and subsequently immersed in solutions of BtP27@*β*-CD, BtP27, *β*-CD, and water, each at a concentration of 200 *μ*g mL^-1^. After 30 seconds, the discs were lifted vertically with tweezers until residual droplets were removed, and their masses were determined using an analytical balance. Water served as the control. This procedure was repeated three times for each sample. The leaf hydration capacity (LHC) was then calculated using the following equation: LHC = (M_1_-M_0_)/S, where M_0_ represents the initial mass of the leaf disc, M_1_ denotes the mass of the leaf disc post-wetting, and S is the area of the leaf disc.

**2.5.3 Droplet impact assay**

The dynamic impact of droplets on rice leaves was meticulously recorded using an i-SPEED 220 high-speed camera (iX Cameras) operating at 2000 frames per second. Droplets containing BtP27@*β*-CD, BtP27, *β*-CD, and water were dispensed via a microsyringe with an inner diameter of 0.25 mm, each at a concentration of 200 *μ*g mL^-1^. Video analysis, conducted using the i-SPEED Suite software, provided detailed parameters. For the splash and bounce experiments, droplets were released from heights of 30 cm and 10 cm, respectively.

**2.5.4 Spray observation**

A solution of 10 mL of BtP27@*β*-CD, BtP27, and *β*-CD, each at a concentration of 200 *μ*g mL^-1^, was prepared, and water was used as a control. This solution was then sprayed evenly on rice leaves. Subsequently, photos were taken to observe the dispersion of the droplets.

**2.5.5 Dispersion and deposition of BtP27@*β*-CD and BtP27 on rice leaf**

The surface morphology of rice leaves following application of BtP27@*β*-CD and BtP27 was characterized using scanning electron microscopy (SEM). In brief, 20 *μ*L droplets of 200 *μ*g mL^-1^ BtP27@*β*-CD and BtP27 were sprayed onto rice leaves mounted on a carrier stage. After drying at ambient temperature, all samples underwent SEM analysis to elucidate their surface characteristics.

**2.6 *In vivo* activity and safety evaluation**

**2.6.1** ***In vivo* efficacy against *Xoo*-induced rice bacterial leaf blight**

To ascertain the *in vivo* protective efficacy of BtP27@*β*-CD against rice bacterial leaf blight induced by *Xoo* infection, the following methodology was employed: Initially, the rice leaves were uniformly sprayed with solutions of BtP27@*β*-CD, BtP27, and TC (SC, 20%) at a concentration of 200 *μ*g mL^-1^. After 24 hours, the leaf tips were excised to a length of 1-2 cm using sterilized scissors. Subsequently, the wounds were immersed in a suspension of *Xoo* cells with an OD_595_ of 0.6. All treated plants were incubated in an artificial climate chamber maintained at 28°C and 90% relative humidity for 14 days. The defense efficacy was assessed using a graded standard count method, and the disease index (C or T) was calculated according to the specified formula. To evaluate the *in vivo* curative efficacy of BtP27@*β*-CD against rice bacterial leaf blight, the following procedure was conducted: Initially, the leaf tips were excised to a length of 1-2 cm using sterilized scissors. The wounds were then immersed in a suspension of *Xoo* cells with an OD_595_ of 0.6. After 24 hours, the leaves were uniformly sprayed with solutions of BtP27@*β*-CD, BtP27, and TC (SC, 20%) at a concentration of 200 *μ*g mL^-1^. The treated rice plants were then incubated in an artificial climate chamber set to 28°C and 90% relative humidity for 14 days. The defense efficacy was determined using a graded standard counting method, and the disease index (*C* or *T*) was calculated using the following formula:

$Disease index (C\mathrm{or}T) = \sum(the number of leaves at each Grade \times the corresponding Grade) / (the total number of leaves \times the superlative Grade).$

The control efficiency (*I*) was calculated using the equation:

$$Control efficiency I \left( \% \right)=\left( C - T \right)/C \times100$$

where *C* represents the disease index of the negative control, and *T* denotes the treatment group.

**2.6.2 Phytotoxicity assay**

Fifty milliliters of BtP27@*β*-CD and BtP27 at concentrations of 200 and 500 *μ*g mL^-1^, respectively, were uniformly sprayed onto rice leaves, with an equal volume of DMSO serving as a blank control. After a 7-day incubation period in the greenhouse, the symptoms of rice leaf intoxication were documented and photographed.

**2.6.3 Toxicity assay on zebrafish**

During the experiment, acute toxicity tests were performed on zebrafish using the hydrostatic bioassay method in the absence of feeding. BtP27@*β*-CD or BtP27 was co-cultured with 10 live fish in 1.0 L aqueous solutions at drug concentrations of 20, 15, 10, 5, and 0 *μ*g mL^-1^, with each concentration tested in triplicate. The survival rate of the zebrafish was recorded after 96 hours, and the LC_50_ was determined through a linear fitting equation.

**2.6.4 Toxicity assay on earthworm**

The acute toxicity test on earthworms was conducted using the filter paper exposure method in accordance with relevant OECD standards. Initially, cut filter paper was placed in a disposable transparent plastic bowl measuring 10 cm in length and 6 cm in depth. Subsequently, 2 mL of a 15.0 *μ*g mL^-1^ BtP27@*β*-CD solution or a BtP27 solution was slowly dripped onto the filter paper. Ten earthworms were then introduced into each plastic bowl. To maintain humidity and sealing, the bowls were covered with sealing film, with a small hole made for ventilation. The bowls were kept at (20 ± 1) °C, 75% humidity, and protected from light. A separate control group with deionized water was included. The survival of the earthworms was recorded after 48 hours of exposure. Earthworms that did not respond to mechanical stimuli were considered dead, those exhibiting symptoms such as significant exudation of yellow body fluid were classified as poisoned, and those with no obvious symptoms were deemed normal.

**2.6.5 *In vivo* efficacy against *Xoc*-induced rice bacterial leaf streak**

Initially, the leaves were uniformly sprayed with a solution containing BtP20@*β*-CD, BtP20, and TC (SC, 20%) at a concentration of 200 *μ*g mL^-1^, with an equal volume of DMSO serving as the control. After 24 hours, the leaves were inoculated with *Xoc* (OD_595_ = 0.6) by injecting 100 μL of the bacterial solution into the rice leaf pulp cells using a quantitative syringe. The inoculated rice plants were then incubated in an artificial climate chamber maintained at 28°C and 90% relative humidity for 14 days. The lesion lengths were measured, and the protective efficacy was calculated using the specified formula. To assess curative efficacy, *Xoc* (OD_595_ = 0.6) was first injected into the rice leaf pulp cells using a dosing syringe, with 100 μL of the bacterial solution per injection. After 24 hours, the leaves were uniformly sprayed with a solution containing BtP20@*β*-CD, BtP20, and TC (SC, 20%) at a concentration of 200 *μ*g mL^-1^, with an equal volume of DMSO used as a control. The treated rice plants were then incubated in an artificial climate chamber set to 28 °C and 90% relative humidity for 14 days. The lesion lengths were measured, and the treatment effectiveness was calculated using the formula:

$$Control efficiency I \left( \% \right)=\left( C - T \right)/C\times100$$

In this equation, C represents the lesion length in the control group, and T represents the lesion length in the treatment group.

**2.6.6 *In vivo* efficacy against *Rs*-induced rice sheath blight**

Initially, rice leaves were uniformly sprayed with solutions of BtP7@*β*-CD, BtP7, hymexazol, and thifluzamide at a concentration of 200 *μ*g mL^-1^, with an equal volume of DMSO serving as the control. After 24 hours, 5-mm diameter fungal discs were excised from pre-activated rice sheath blight cultures using a perforator and inoculated onto the leaf surfaces with sterile needles. The treated plants were then incubated under controlled conditions of 28°C and 90% relative humidity for 14 days. Lesion areas were subsequently measured, and the protective efficacy was quantified. To assess the curative efficacy, 5-mm fungal discs were prepared and inoculated on the rice leaves as described. After 24 hours, the leaves were treated with solutions of BtP7@*β*-CD, BtP7, hymexazol, and thifluzamide at 200 *μ*g mL^-1^, while DMSO was used as the control. Following a 14-day incubation period at 28 °C and 90% relative humidity, lesion areas were measured to calculate efficacy. The control efficiency against rice sheath blight was calculated using the following equation:

$$Control efficiency I \left( \% \right)=\left( C - T \right)/C \times100$$

where C represents the lesion area in the control group, and T represents the lesion area in the treatment group.

**2.6.7 *In vivo* efficacy against *Mo*-induced rice blast**

Initially, rice leaves were uniformly treated with solutions containing BtP11@*β*-CD, BtP11, isoprothiolane, and prochloraz, each at a concentration of 200 *μ*g mL^-1^, with an equivalent volume of DMSO serving as the control. After 24 hours, 5-mm diameter fungal discs were prepared using a perforator and inoculated onto the leaf surfaces with a sterile needle. The treated rice plants were subsequently incubated in an artificial climate chamber set to 28°C and 90% relative humidity for 14 days. The lesion areas were measured, and the protective efficacy was quantified using the formula provided. To evaluate the curative efficacy, 5-mm diameter fungal discs were prepared and inoculated onto rice leaves as described. After 24 hours, the leaves were treated with solutions of BtP11@*β*-CD, BtP11, isoprothiolane, and prochloraz at 200 *μ*g mL^-1^, with DMSO as the control. The plants were then incubated for 14 days in an artificial climate chamber at 28 °C and 90% relative humidity. Control efficiency against rice blast was determined using the following equation:

$$Control efficiency I \left( \% \right)=\left( C - T \right)/C \times100$$

In this equation, *C* represents the lesion area in the control group, and *T* represents the lesion area in the treatment group.

**2.7 Investigations into the mechanism of action**

**2.7.1 Growth curve determination**

The *Xoo* bacterial suspension was standardized to an optical density of 0.1 at 595 nm (OD_595_). Subsequently, 20 mL of this suspension was distributed into a series of conical flasks, each containing varying concentrations of BtP27@*β*-CD and BtP27. The concentrations employed were 0, 1.0 EC_50_, 2.0 EC_50_, 4.0 EC_50_, and 8.0 EC_50_, with triplicate assays for each concentration. The flasks were then incubated in a shaker set to 28°C with agitation at 180 rpm. OD_595_ values were recorded at 3-hour intervals over a 30-hour period.

**2.7.2** **Confocal laser scanning microscopy (****CLSM) 3D imaging**

**Inhibition of *Xoo* Biofilm Formation.** A bacterial suspension of *Xoo*, adjusted to an OD_595_ of 0.1, was incubated with varying concentrations of BtP27@*β*-CD, BtP27, and *β*-CD, including 0, 1.0 EC_50_, 2.0 EC_50_, and 4.0 EC_50_. The incubation lasted 48 hours at 28°C, with each concentration assessed in triplicate. Post-incubation, the medium was discarded, and the wells were washed thrice with PBS to eliminate any planktonic cells. The biofilms were then stained using a combination of acridine orange (0.1% AO) and propidium iodide (0.01% PI) for 10 minutes each. Fluorescent images were captured using confocal laser scanning microscopy (CLSM) with excitation wavelengths of 488 nm and 535 nm.

**Eradication of *Xoo* Mature Biofilm.** *Xoo* bacteria, standardized to an OD_595_ of 0.1, were incubated for 48 hours to allow the formation of mature biofilms. Following incubation, the culture medium was removed, and the biofilm-coated wells were gently washed twice with PBS (pH 7.4, 10 mM). Subsequently, BtP27@*β*-CD, BtP27, and *β*-CD were added at concentrations of 0, 64 EC_50_, 100 EC_50_, and 200 EC_50_, with each concentration tested in triplicate. The plates were then incubated at 28°C for an additional 24 hours. After this period, the wells were washed thrice with PBS, stained with a mixture of 0.1% AO and 0.01% PI for 10 minutes each. Fluorescent images were then visualized using CLSM in 3D mode with excitation wavelengths of 488 nm and 535 nm.

**2.7.3 Morphologic observation of biofilm inhibition**

Inoculate *Xoo* bacteria overnight and adjust the culture medium to an OD_595_ of 0.1. Transfer 1 mL of the bacterial solution into a sterile centrifuge tube containing slides. Add BtP27@*β*-CD, BtP27, and an equal volume of DMSO to the tube at concentrations of 0, 1.0 EC_50_, 2.0 EC_50_, and 4.0 EC_50_, respectively. Incubate the mixture for 48 hours at 28 °C. Carefully aspirate the bacterial solution from the centrifuge tube, then gently add 1 mL of PBS (pH 7.4, 10 mM) to wash the sample once. Subsequently, add 1 mL of 2.5% glutaraldehyde and fix the sample overnight. After removing the glutaraldehyde, sequentially replace it with 1 mL of 30%, 50%, 70%, 90%, and 100% ethanol for 10 minutes each. Freeze-dry the samples for 3 hours. Attach a conductive adhesive to the sample stage, cut a small piece of slide with a glass cutter, and affix it to the conductive adhesive. Coat the sample with gold for 45 seconds, then observe and photograph the sample for biofilm inhibition using SEM.

**2.7.4 Morphologic observation of biofilm eradication**

Inoculate *Xoo* bacteria and culture overnight, adjusting the OD_595_ of the bacterial suspension to 0.1. Dispense 1 mL of this suspension into a sterile centrifuge tube containing a sterilized slide, and incubate at 28 °C for 48 hours. Subsequently, gently aspirate the bacterial suspension, wash the slide twice with PBS (pH 7.4, 10 mM), and add DMSO containing BtP27@*β*-CD, BtP27, and an equivalent volume at concentrations of 0, 64 EC_50_, 100 EC_50_, and 200 EC_50_, respectively. Incubate for an additional 24 hours. Wash once with 1 mL of PBS (pH 7.4, 10 mM), then fix overnight with 1 mL of 2.5% glutaraldehyde. Post-fixation, sequentially replace the glutaraldehyde with 1 mL each of 30%, 50%, 70%, 90%, and 100% ethanol for 10 minutes per step, and finally freeze-dry for 3 hours. Affix the sample to a conductive adhesive on the sample stage, cut a small piece from the glass slide using a glass knife, and mount it on the adhesive. Coat with gold for 45 seconds, then observe and photograph the biofilm eradication using SEM.

**2.7.5 Pathogenicity assay**

The pathogenicity assay was conducted by inoculating premixed *Xoo* cells (OD_595_ = 0.1) with BtP27@*β*-CD, BtP27, and *β*-CD at a concentration of 1.0 EC_50_, alongside an equivalent DMSO solution, onto adult rice plants using a leaf-cutting method. After incubating the rice plants in a greenhouse for 14 days, the lesion lengths were measured and documented photographically.

**2.7.6 *OsNPR1* gene expression assay**

A 200 *µ*g mL^-1^ solution of BtP27 and BtP27@*β*-CD was prepared and uniformly applied to rice leaves via spraying. Water served as the control group. After 24 hours, rice leaves were harvested for further analysis. Total RNA was extracted using the classical Trizol method (TaKaRa, Beijing, China), followed by reverse transcription with the FastKing gDNA Dispelling RT SuperMix (Tiangen Biotechnology, Beijing, China). Finally, quantitative real-time polymerase chain reaction (qRT-PCR) was conducted according to the manufacturer's instructions provided in the Hieff q-PCR SYBR Green Master Mix (No ROX) kit (Yeasen Biotechnology, Shanghai, China).

**2.7.7 Determination of extracellular polysaccharide (EPS)**

*Xoo* was inoculated onto the culture medium and incubated until an OD_595_ of 0.6 was reached. The culture was then centrifuged and resuspended to an OD_595_ of 0.1. Different concentrations of *β*-CD, BtP27, and BtP27@*β*-CD were added, followed by incubation for 48 hours, while the control group received no treatment. After incubation, the bacterial suspension was centrifuged, and the supernatant was collected for analysis. To precipitate the extracellular polysaccharides (EPSs), 100 mL of absolute ethanol was added to the supernatant, and the mixture was left overnight. Once precipitation was complete, photographs were taken, and the sample was filtered, dried, and weighed. The quantification of secreted EPS secreted was subsequently determined.

**2.7.8 Biofilm-related gene expression assay**

*Xoo* was cultured overnight, then centrifuged and resuspended to an OD_595_ of 0.1. BtP27 and BtP27@*β*-CD at a concentration of 0.87 *µ*g mL^-1^ were added, followed by incubation for 12 hours; the control group remained untreated. The bacterial suspension was then centrifuged to collect the bacteria. Total RNA was extracted using the Molpure Bacterial RNA Kit according to the manufacturer’s protocol (Yesen Biotechnology, Shanghai, China). Reverse transcription was performed using the FastKing gDNA Dispelling RT SuperMix (Tiangen Biotechnology, Beijing,China). Finally, quantitative real-time polymerase chain reaction (qRT-PCR) was conducted using the Hieff qPCR SYBR Green Master Mix (No ROX) (Yesen Biotechnology, Shanghai, China), following the provided protocol.

**2.7.9 T3SS-related Gene Expression Assay**

*Xoo* cells were co-cultured with BtP27@*β*-CD and BtP27 at a concentration of 1.0 EC_50_, along with an equivalent volume of DMSO as a blank control. The *Xoo* suspensions were subsequently collected and subjected to immediate total RNA extraction using the TransZol Up kit (TaKaRa, Beijing, China). This was followed by reverse transcription using the cDNA kit (TaKaRa, Dalian, China). The SYBR Premix Ex TaqII (TaKaRa) was employed to quantify the levels of cDNA corresponding to virulence factors, utilizing the LightCycler96 real-time fluorescent quantitative PCR detection system (Roche, Switzerland). The relative transcription levels of the genes were analyzed using the 2-ΔΔCt method, with the *gyrB* gene serving as the internal control. The relevant primer sequences for qRT-PCR are detailed in Table S1.

**2.7.10 Reactive oxygen species (ROS) detection and analysis**

Following the protocol detailed in the ROS kit instructions (Beyotime Biotechnology, Shanghai, China), *Xoo* was inoculated onto the culture medium and incubated until an OD_595_ of 0.6 was reached. The culture was then centrifuged, resuspended in 1 mL of sterile water, and washed. Subsequently, 1 *µ*L of DCFH-DA dye was added to 1 mL of the resuspended culture, followed by incubation at 37°C for 20 minutes with gentle inversion every 3–5 minutes to ensure uniform mixing. The cells were then washed three times with water to remove excess dye. Different concentrations of *β*-CD, BtP27, and BtP27@*β*-CD were added, and the mixture was incubated for 30 minutes. Finally, ROS levels were quantified using a fluorescence spectrometer with excitation and emission wavelengths set at 488 nm and 525 nm, respectively.

**2.7.11 Enzyme activity assay**

Following the instructions provided in the assay kit (Solarbio, Beijing, China), *Xoo* was inoculated onto the culture medium and cultured until an OD_595_ of 0.6 was reached. The culture was centrifuged, resuspended to an OD_595_ of 0.1, and treated with varying concentrations of *β*-CD, BtP27, and BtP27@*β*-CD for 12 hours; the control group received no treatment. The bacterial suspension was centrifuged to collect the precipitate, discarding the supernatant. A precise 0.1 g of the precipitate was weighed and resuspended in 1 mL of extraction buffer, followed by ultrasonic disruption. The mixture was centrifuged at 8000 g for 10 minutes at 4°C, and the supernatant was collected and stored on ice for subsequent catalase (CAT) and superoxide dismutase (SOD) activity assays. For the CAT assay, the protocol described in the CAT assay kit was followed. The microplate reader was preheated for 30 minutes, and the wavelength was set to 240 nm. Distilled water was used for baseline calibration. The working solution was incubated at 25 °C for 10 minutes, and 10 *µ*L of sample was mixed with 190 *µ*L of the working solution. The initial absorbance at 240 nm (A_1_) and the absorbance after 1 minute (A_2_) were recorded. The change in absorbance (ΔA = A_1_ - A_2_) was used to calculate CAT activity using the formula: CAT (U/g) = 764.5 × ΔA / 0.1. For the SOD assay, the reaction mixture was prepared according to the SOD assay kit instructions and mixed thoroughly. The reaction was carried out at 37°C for 30 minutes, and the absorbance at 560 nm was measured. The change in absorbance were calculated as follows: ΔA_measured_ = A_measured_ - A_control_ and ΔA_blank_ = A_blank_ – A_blank control_. SOD activity was then determined using the formulas: Inhibition percentage = (ΔA_blank_ - ΔA_measured_) / ΔA_blank_ × 100 and SOD (U/g) = 10 × inhibition percentage / (1 - inhibition percentage) / 0.1.

**2.7.12 Statistical analysis**

Statistical analyses were performed using IBM SPSS Statistics 26 and Origin 2021 for Windows. Unless otherwise specified, all experiments were conducted independently with a minimum of three replicates with the similar results. Data are presented as mean ± standard deviation (SD), and the sample size corresponding to each data point is specified within the respective figure legend. Statistically significant differences between the means were analyzed with one-way ANOVA, followed by the least significant difference (LSD) post-hoc test. (For all studies, n ≥ 3; **p* < 0.05, ***p* < 0.01, ****p* < 0.001; n.s. = no signiﬁcance). It should be noted that all statistical tests conducted in this study were two-sided.

# 3. Supplementary Figures and Tables

**Table S1.** Results of CoMFA model.

| # Factors | SD | R^2^ | R^2^ CV | R^2^ Scramble | Stability | F | *P* | RMSE | Q^2^ | Pearson-r |
| --- | --- | --- | --- | --- | --- | --- | --- | --- | --- | --- |
| 1 | 3.9793 | 0.204 | -0.1034 | 0.4372 | 0.857 | 5.4 | 0.0305 | 27.69 | -0.2758 | -0.42 |
| 2 | 3.2538 | 0.4931 | -0.3734 | 0.626 | 0.418 | 9.7 | 0.00112 | 27.59 | -0.2668 | 0.0932 |
| 3 | 2.1798 | 0.7839 | -1.0104 | 0.8145 | -0.696 | 23 | 1.55E-06 | 27.81 | -0.2875 | 0.11 |
| 4 | 1.6752 | 0.8791 | -1.0344 | 0.9069 | -0.914 | 32.7 | 4.93E-08 | 27.98 | -0.3026 | 0.1032 |
| 5 | 1.1944 | 0.9419 | -1.1225 | 0.9526 | -1 | 55.2 | 6.57E-10 | 27.67 | -0.2743 | 0.2319 |

**Table S2.** Scores of CoMFA model.

| # Factors | Force Field Steric | Force Field Electrostatic |
| --- | --- | --- |
| 1 | 0.406 | 0.594 |
| 2 | 0.621 | 0.379 |
| 3 | 0.697 | 0.303 |
| 4 | 0.707 | 0.293 |
| 5 | 0.69 | 0.31 |

**Table S3.** Results of CoMSIA model**.**

| # Factors | SD | R^2^ | R^2^ CV | R^2^ Scramble | Stability | F | *P* | RMSE | Q^2^ | Pearson-r |
| --- | --- | --- | --- | --- | --- | --- | --- | --- | --- | --- |
| 1 | 11.662 | 0.4472 | -0.4896 | 0.4664 | 0.4 | 17 | 0.000486 | 12.54 | -6.116 | 0.1109 |
| 2 | 8.5205 | 0.7189 | -0.7408 | 0.7329 | -0.13 | 25.6 | 3.08E-06 | 14.05 | -7.9277 | 0.0524 |
| 3 | 6.4533 | 0.8468 | -0.7034 | 0.8642 | -0.33 | 35 | 6.09E-08 | 14.54 | -8.5683 | 0.0759 |
| 4 | 5.2349 | 0.9045 | -0.7634 | 0.9242 | -0.519 | 42.6 | 6.03E-09 | 15.24 | -9.5028 | 0.0119 |
| 5 | 3.9024 | 0.9499 | -0.7792 | 0.9555 | -0.635 | 64.4 | 1.90E-10 | 13.36 | -7.069 | -0.1322 |

**Table S4.** Scores of CoMSIA model**.**

| # Factors | Steric Field | Electrostatic Field | Hydrophobic Field | H-bond Acceptor Field | H-bond Donor Field |
| --- | --- | --- | --- | --- | --- |
| 1 | 0.537 | 0.081 | 0.148 | 0.115 | 0.119 |
| 2 | 0.518 | 0.079 | 0.158 | 0.127 | 0.118 |
| 3 | 0.488 | 0.081 | 0.175 | 0.137 | 0.118 |
| 4 | 0.451 | 0.087 | 0.193 | 0.147 | 0.122 |
| 5 | 0.462 | 0.085 | 0.216 | 0.137 | 0.101 |

**Table S5.** Explanation of Statistical Parameters for 3D-QSAR.

| **Column** | **Description** |
| --- | --- |
| #Factors | The number of factors in a partial least squares regression (PLS) model. |
| SD | Standard deviation of the regression. This is the RMS error of the fitted activity values distributed over n-m-1 degrees of freedom (n ligands, m PLS factors). |
| R^2^ | R Squared is also known as the coefficient of determination, also known as the goodness of fit, reflecting the degree to which the independent variable x explains the changes in the dependent variable y. The closer to 1, the better the model fits. The closer it is to 1, the better the model fits. |
| R^2^ CV | The R value (coefficient of determination) of the regression. For example, a value of 0.80 means that the model accounts for 80% of the variance of the observed activity data. r is always between 0 and 1. |
| R^2^ Scramble | The R-mean of a series of models constructed using perturbed activity. A measure of how well the molecular field can fit random data. A low value means that the model cannot fit random data, but a high value just means that the set of variables is fairly complete and can fit anything. |
| Stability | Stability of model predictions to changes in the composition of the training set. The maximum value is 1. High values indicate that the model is insensitive to omissions in the training set. Stability values below the R-value indicate overfitting. |
| F | Ratio of model variance to observed activity variance. The model variance is distributed over m degrees of freedom, and the activity variance is distributed over n-m-1 degrees of freedom (n ligands, mPLS factors). Large values of F indicate that the regression is more statistically significant. |
| *P* | The level of significance when treating F as a ratio of the chi-square distribution. A smaller value indicates a higher level of confidence. *P*-value of 0.05 means that F is significant at the 95% level. |
| RMSE | Root Mean Square Error in Test Set Predictions. |
| Q^2^ | Predicts the Q-value of the activity. Directly analogous to R-squared, but based on test set predictions. Q can take a negative value if the variance of the error is greater than the observed variance |
| Pearson-r | Pearson-r values for the correlation between predicted and observed activities for the test set. |

**Table S6.** Chemical shift and chemical shift change (Δ*δ*) of BtP27 with different molar ratios of *β*-CD addition.

| Molar ratio | Chemical shift (ppm) | | Δ*δ* (ppm) | |
| --- | --- | --- | --- | --- |
|  | H-3 | H-37 | H-3 | H-37 |
| BtP27:*β*-CD = 1:0 | 5.427 | 2.213 | - | - |
| BtP27:*β*-CD = 1:0.3 | 5.376 | 2.249 | -0.051 | 0.036 |
| BtP27:*β*-CD = 1:0.6 | 5.339 | 2.265 | -0.088 | 0.052 |
| BtP27:*β*-CD = 1:1 | 5.286 | 2.287 | -0.141 | 0.074 |
| BtP27:*β*-CD = 1:1.2 | 5.274 | 2.296 | -0.153 | 0.083 |

**Table S7.** *In vivo* efficacy against *Xoo*-induced rice bacterial leaf blight.

| Treatment | Curative effect | | | Protective effect | | |
| --- | --- | --- | --- | --- | --- | --- |
|  | Morbidity  (%) | Disease index (%) | Control efficiency (%) *^b^* | Morbidity  (%) | Disease index (%) | Control  efficiency (%) *^b^* |
| BtP27@*β*-CD | 100 | 46.66 ± 3.24 | 51.16 ± 2.35 | 100 | 36.00 ± 1.16 | 62.67 ± 3.08 |
| BtP27 | 100 | 56.44 ± 3.87 | 40.93 ± 2.87 | 100 | 47.55 ± 2.18 | 50.69 ± 2.84 |
| TC | 100 | 59.11 ± 4.14 | 38.13 ± 2.20 | 100 | 60.00 ± 3.06 | 37.78 ± 2.44 |
| CK *^a^* | 100 | 95.55 ± 2.11 |  | 100 | 96.44 ± 2.35 |  |

*^a^* Negative control. *^b^* Statistical analysis was conducted using ANOVA under equal variances assumed (*P* > 0.05) and equal variances not assumed (*P* < 0.05).

**Table S8.** Protective efficiencies after upward and downward conduction.

| Treatment | Upward conduction | | | Downward conduction | | |
| --- | --- | --- | --- | --- | --- | --- |
|  | Morbidity  (%) | Average area (cm) | Control efficiency (%) *^b^* | Morbidity  (%) | Average area (cm) | Control efficiency (%) *^b^* |
| BtP27@*β*-CD | 100 | 0.81 ± 0.10 | 88.90 ± 1.86 | 100 | 2.31 ± 0.21 | 62.47 ± 5.36 |
| BtP27 | 100 | 2.11 ± 0.10 | 72.72 ± 2.21 | 100 | 4.30 ± 0.23 | 33.57 ± 3.88 |
| *β*-CD | 100 | 7.31 ± 0.11 | 5.68 ± 2.20 | 100 | 6.15 ± 0.21 | 4.83 ± 4.02 |
| CK *^a^* | 100 | 7.76 ± 0.16 |  | 100 | 6.48 ± 0.25 |  |

*^a^* Negative control. *^b^* Statistical analysis was conducted using ANOVA under equal variances assumed (*P* > 0.05) and equal variances not assumed (*P* < 0.05).

**Table S9.** Results of acute toxicity toward zebrafish treated for 96 h.

| Concentration (*μ*g mL^-1^) | Mortality | | | | | | | |
| --- | --- | --- | --- | --- | --- | --- | --- | --- |
|  | 24 h | | 48 h | | 72 h | | 96 h | |
|  | BtP27@*β*-CD | BtP27 | BtP27@*β*-CD | BtP27 | BtP27@*β*-CD | BtP27 | BtP27@*β*-CD | BtP27 |
| 20 | 30 | 30 | 30 | 30 | 30 | 30 | 30 | 30 |
| 15 | 20 | 24 | 22 | 24 | 22 | 26 | 24 | 27 |
| 10 | 0 | 0 | 0 | 0 | 0 | 0 | 0 | 0 |
| 5 | 0 | 0 | 0 | 0 | 0 | 0 | 0 | 0 |
| 0 | 0 | 0 | 0 | 0 | 0 | 0 | 0 | 0 |

**Table S10.** LC_50_ values of of acute toxicity toward zebrafish treated for 96 h.

| Compounds | Toxic regression equation | R^2^ | LC_50_ (*μ*g mL^-1^) |
| --- | --- | --- | --- |
| BtP27 | y = 24.9510x - 23.4920 | 0.9903 | 13.86 ± 0.12 |
| BtP27@*β*-CD | y = 24.7870x - 23.4480 | 0.9990 | 14.05 ± 0.21 |

##

**Table S11.** Primer sequences of representative genes for qRT-PCR.

| Gene | Forward primer | Reverse primer |
| --- | --- | --- |
| *HrpG* | 5′- CGCAATGTCTCGGTGTTCTC -3′ | 5′- CTGGCGTCGAAGACCAGTAA -3′ |
| *HrpF* | 5′- AGATCGTTTCGACGCTGAAT-3′ | 5′- CTGCGGATGTGAATCAGAGA -3′ |
| *HrpE* | 5′- ACGAGGCTCAGAAGTCCA -3′ | 5′- GAGCTGCTTAGCGTTGTC -3′ |
| *HrcT* | 5′- GAGGCGTTCATTGGTCTG -3′ | 5′- AACAGCAGCACGCCCAGT -3′ |
| *GumB* | 5′- GCCATATTTCGTTGCCGCTT -3′ | 5′- GGAACACGATGACATTGCCG -3′ |
| *GumD* | 5′- GTTGCCTGTTGAGCGAACTG-3′ | 5′- TGGTTCAAAAAGCCACGCAG-3′ |
| *GumI* | 5′- CGAATACTTGCTGCGCCATC -3′ | 5′- CCGTGCAGAATCGTATCGGT -3′ |
| *GumJ* | 5′- GCTATGCCAGTCGCCAATGTG -3′ | 5′- CAACAGGAACGCACTGAAGC -3′ |
| *OsNPR1* | 5′- GAGCCCTTGACTCTGACGAT-3′ | 5′- CCTCGCAGCAATGTGAAGAA -3′ |
| *OsActin* | 5′- TGCTGCTCACTGAAGGACAGAC-3′ | 5′- ATCTGCAATGCGAGATCCAA -3′ |
| *gyrB* | 5′- CGAGCTGTATCTGAAGGACG -3′ | 5′- CTGGTGAACAGCAGCAGTAGT-3′ |

**Table S12.** Transcriptional expression levels of related genes.

| Genes | Transcriptional levels | | |
| --- | --- | --- | --- |
|  | Control | BtP27 | BtP27@*β*-CD |
| *HrpE* | 1.0133 ± 0.1040 | 0.7277 ± 0.0760 | 0.6575 ± 0.0209 |
| *HrpF* | 1.0027 ± 0.0890 | 0.8442 ± 0.0630 | 0.8334 ± 0.0980 |
| *HrpG* | 1.0019 ± 0.0770 | 0.4300 ± 0.0740 | 0.4058 ± 0.0440 |
| *HrcT* | 1.0214 ± 0.0700 | 0.8742 ± 0.0670 | 0.8709 ± 0.0320 |
| *GumB* | 1.0034 ± 0.0150 | 0.4849 ± 0.0317 | 0.4199 ± 0.0260 |
| *GumD* | 1.0115 ± 0.0180 | 0.3969± 0.0241 | 0.3494 ± 0.0328 |
| *GumI* | 1.0012 ± 0.0120 | 0.5404 ± 0.0409 | 0.4507 ± 0.0417 |
| *GumJ* | 1.0058 ± 0.0260 | 0.3130 ± 0.0325 | 0.1226 ± 0.0217 |
| *OsNPR1* | 1.0062 ± 0.0100 | 1.7604 ± 0.1201 | 2.1401 ± 0.1410 |

**Table S13.** EC_50_ values of BtP1-BtP30 against against *Xoc ^a^.*

| Compd. | EC_50_ (*µ*g mL^-1^) | Regression equation | R^2^ |
| --- | --- | --- | --- |
| BtP1 | 22.18 ± 1.53 | y = 1.9450x + 2.3820 | 0.9412 |
| BtP2 | 3.81 ± 0.28 | y = 1.7084x + 4.0063 | 0.9894 |
| BtP3 | 4.93 ± 0.46 | y = 2.5757x + 3.2135 | 0.9077 |
| BtP4 | 5.64 ± 0.38 | y = 2.8243x + 2.8766 | 0.9992 |
| BtP5 | 31.85 ± 1.93 | y = 2.3436x + 1.4770 | 0.9827 |
| BtP6 | 23.85 ± 2.54 | y = 1.8256x + 2.4851 | 0.9698 |
| BtP7 | 1.97 ± 0.18 | y = 1.2825x + 4.6209 | 0.9662 |
| BtP8 | 7.24 ± 0.28 | y = 1.6183x + 3.6081 | 0.9624 |
| BtP9 | 7.05 ± 0.17 | y = 1.9096x + 3.3797 | 0.9989 |
| BtP10 | 4.67 ± 0.24 | y = 5.9211x + 1.0336 | 0.9927 |
| BtP11 | 6.42 ± 0.17 | y = 3.4520x + 2.2115 | 0.9854 |
| BtP12 | 4.43 ± 0.021 | y = 3.6996x + 2.4960 | 0.9069 |
| BtP13 | 18.79 ± 1.35 | y = 2.7620x + 1.4809 | 0.9021 |
| BtP14 | 7.17 ± 0.54 | y = 2.2539x + 2.6943 | 0.9146 |
| BtP15 | 10.06 ± 1.42 | y = 1.9812x + 3.0136 | 0.9565 |
| BtP16 | 4.27 ± 0.58 | y = 1.2685x + 4.1992 | 0.9850 |
| BtP17 | 4.71 ± 0.41 | y = 2.3199x + 3.4378 | 0.9985 |
| BtP18 | 4.85 ± 0.40 | y = 1.9640x + 3.6526 | 0.9493 |
| BtP19 | 4.72 ± 0.68 | y = 2.5563x + 3.2759 | 0.9976 |
| BtP20 | 0.95 ± 0.16 | y = 0.6712x + 5.0121 | 0.9938 |
| BtP21 | 3.76 ± 0.11 | y = 3.7676x + 2.8289 | 0.9969 |
| BtP22 | 4.59 ± 0.15 | y = 3.1159x + 2.9371 | 0.9290 |
| BtP23 | 1.32 ± 0.72 | y = 0.9452x + 4.8833 | 0.9950 |
| BtP24 | 9.54 ± 0.37 | y = 3.5451x + 1.5265 | 0.9334 |
| BtP25 | 0.98 ± 0.21 | y = 0.8182x + 5.0061 | 0.9816 |
| BtP26 | 1.64 ± 0.48 | y = 1.0098x + 4.7815 | 0.9295 |
| BtP27 | 3.12 ± 0.17 | y = 1.4880x + 4.2631 | 0.9807 |
| BtP28 | 1.26 ± 0.34 | y = 1.5273x + 4.8457 | 0.9072 |
| BtP29 | 3.51 ± 0.17 | y = 4.0412x + 2.7945 | 0.9365 |
| BtP30 | 3.50 ± 0.18 | y = 1.9104x + 3.9591 | 0.9291 |
| *β*-CD | >100 |  |  |
| Dufulin *^b^* | >100 |  |  |
| BtP20@*β*-CD | 0.86 ± 0.07 | y = 2.4655x + 5.1572 | 0.9704 |
| TC *^b^* | 84.13 ± 1.39 | y = 2.4465x + 0.2905 | 0.9831 |

*^a^* The statistical analysis was conducted by ANOVA method at the condition of equal variances assumed (*P* > 0.05) and equal variances not assumed (*P* < 0.05); *^b^* Commercially available agrochemicals.

**Table S14.** *In vitro* inhibitory effects of BtP1-BtP30 (100 *μ*g mL^-1^) against *Rs* and *Mo* ***^a^***.

| Compd. | *Rs* (%) | *Mo* (%) | Compd. | *Rs* (%) | *Mo* (%) |
| --- | --- | --- | --- | --- | --- |
| BtP1 | 18.89 ± 2.41 | 20.29 ± 1.89 | BtP16 | 32.06 ± 2.62 | 28.12 ± 1.39 |
| BtP2 | 27.34 ± 1.27 | 19.42 ± 2.62 | BtP17 | 52.58 ± 1.57 | 44.06 ± 0.52 |
| BtP3 | 22.63 ±2.89 | 19.13 ± 0.91 | BtP18 | 48.70 ±2.29 | 40.29 ± 1.39 |
| BtP4 | 28.73 ± 3.82 | 26.38 ±1.05 | BtP19 | 47.03 ± 2.73 | 35.07 ± 2.78 |
| BtP5 | 32.89 ± 2.20 | 21.45 ± 1.39 | BtP20 | 58.13 ± 1.89 | 46.67 ± 1.39 |
| BtP6 | 27.62 ±1.27 | 20.87 ± 0.91 | BtP21 | 31.50 ± 1.39 | 28.70 ± 1.57 |
| BtP7 | 97.50 ± 0.96 | 53.62 ± 1.39 | BtP22 | 58.96 ± 1.39 | 43.77 ± 1.39 |
| BtP8 | 36.49 ± 2.55 | 40.00 ± 0.91 | BtP23 | 55.91± 0.91 | 39.13 ± 2.41 |
| BtP9 | 74.49 ± 6.74 | 51.59 ± 1.39 | BtP24 | 61.54 ± 2.62 | 45.80 ± 1.14 |
| BtP10 | 69.77 ± 1.67 | 54.49 ± 0.52 | BtP25 | 67.00 ± 2.92 | 55.36 ± 2.50 |
| BtP11 | 78.37 ± 0.96 | 92.03 ± 1.05 | BtP26 | 68.94 ± 2.78 | 59.71 ± 1.05 |
| BtP12 | 79.76 ± 1.27 | 57.10 ± 0.52 | BtP27 | 64.23 ± 1.82 | 51.88 ± 1.05 |
| BtP13 | 32.89 ± 1.39 | 28.99 ± 2.12 | BtP28 | 65.61 ± 1.05 | 54.49 ± 2.10 |
| BtP14 | 17.08 ± 0.52 | 13.33 ± 2.92 | BtP29 | 65.61 ± 1.39 | 51.59 ± 1.39 |
| BtP15 | 29.28 ± 1.39 | 23.77 ± 2.10 | BtP30 | 64.23 ± 1.82 | 51.88 ± 1.05 |
| *β*-CD | 1.09 ± 0.11 | 0.76 ± 0.16 | BtP7@*β*-CD | 98.90 ± 1.01 | / |
| BtP11@*β*-CD | / | 98.52 ± 1.82 | Hymexazol *^b^* | 72.01 ± 2.17 | / |
| [Thifluzamide](https://www.chemsrc.com/en/cas/130000-40-7_402146.html) *^b^* | 98.86 ± 1.24 | / | Isoprothiolane *^b^* | / | 78.42± 1.09 |
| Prochloraz *^b^* | / | 96.66 ± 1.18 | Dufulin *^b^* | 1.25 ± 0.12 | 3.01 ± 0.26 |

*^a^* The statistical analysis was conducted by ANOVA method at the condition of equal variances assumed (*P* > 0.05) and equal variances not assumed (*P* < 0.05); *^b^* Commercially available agrochemicals.

**Table S15**. *In vivo* efficiency against *Xoc*-induced ed·rice bacterial leaf streak under the greenhouse condition.

| Treatment | Curative effect | | | Protective effect | | |
| --- | --- | --- | --- | --- | --- | --- |
|  | Morbidity (%) | Length (cm) | Control efficiency (%) *^b^* | Morbidity (%) | Length (cm) | Control efficiency (%) *^b^* |
| BtP20@*β*-CD | 100 | 1.19 ± 0.01 | 42.18 ± 1.30 | 100 | 0.80 ± 0.04 | 63.53 ± 2.44 |
| BtP20 | 100 | 1.53 ± 0.08 | 25.79 ± 2.08 | 100 | 1.28 ± 0.06 | 42.14 ± 2.89 |
| TC | 100 | 1.48 ± 0.03 | 27.95 ± 1.47 | 100 | 1.46 ± 0.07 | 34.01 ± 2.87 |
| CK *^a^* | 100 | 2.06 ± 0.08 |  | 100 | 2.11 ± 0.02 |  |

*^a^* Negative control. *^b^* Statistical analysis was conducted using ANOVA under equal variances assumed (*P* > 0.05) and equal variances not assumed (*P* < 0.05).

**Table S16.** *In vivo* efficiency against *Rs*-induced·rice sheath blight under the greenhouse condition.

| Treatment | Curative effect | | | Protective effect | | |
| --- | --- | --- | --- | --- | --- | --- |
|  | Morbidity (%) | Average area (cm^2^) | Control efficiency (%) *^b^* | Morbidity (%) | Average area (cm^2^) | Control efficiency (%) *^b^* |
| BtP7@*β*-CD | 100 | 0.22 ± 0.02 | 60.41 ± 3.89 | 100 | 0.19 ± 0.02 | 65.09 ± 3.13 |
| BtP7 | 100 | 0.25 ± 0.01 | 40.58 ± 2.01 | 100 | 0.20 ± 0.01 | 56.45 ± 2.62 |
| Hymexazl | 100 | 0.51 ± 0.03 | 15.25 ± 1.42 | 100 | 0.39 ± 0.02 | 30.45 ± 2.47 |
| Thifluzamide | 100 | 0.34 ± 0.04 | 41.77 ± 2.71 | 100 | 0.32 ± 0.02 | 43.65 ± 2.49 |
| CK *^a^* | 100 | 0.58 ± 0.04 |  | 100 | 0.57 ± 0.03 |  |

*^a^* Negative control. *^b^* Statistical analysis was conducted using ANOVA under equal variances assumed (*P* > 0.05) and equal variances not assumed (*P* < 0.05).

**Table S17.** *In vivo* efficiency against *Mo*-induced·rice blast under the greenhouse condition.

| Treatment | Curative effect | | | Protective effect | | |
| --- | --- | --- | --- | --- | --- | --- |
|  | Morbidity (%) | Average area (cm^2^) | Control efficiency (%) *^b^* | Morbidity (%) | Average area (cm^2^) | Control efficiency (%) *^b^* |
| BtP11@*β*-CD | 100 | 0.18 ± 0.02 | 57.67 ± 1.12 | 100 | 0.12 ± 0.01 | 73.30 ± 1.01 |
| BtP11 | 100 | 0.29 ± 0.01 | 48.69 ± 1.76 | 100 | 0.26 ± 0.02 | 52.62 ± 2.09 |
| Isoprothiolane | 100 | 0.32 ± 0.01 | 24.40 ± 1.24 | 100 | 0.21 ± 0.02 | 54.95 ± 1.96 |
| Prochloraz | 100 | 0.22 ± 0.02 | 48.81 ± 2.34 | 100 | 0.17 ± 0.01 | 61.77 ± 1.12 |
| CK *^a^* | 100 | 0.42 ± 0.02 |  | 100 | 0.45 ± 0.02 |  |

*^a^* Negative control. *^b^* Statistical analysis was conducted using ANOVA under equal variances assumed (*P* > 0.05) and equal variances not assumed (*P* < 0.05).

**Figure S1.** Representative molecules featuring isopropanolamine, benzothiazole, or α-aminophosphonate.


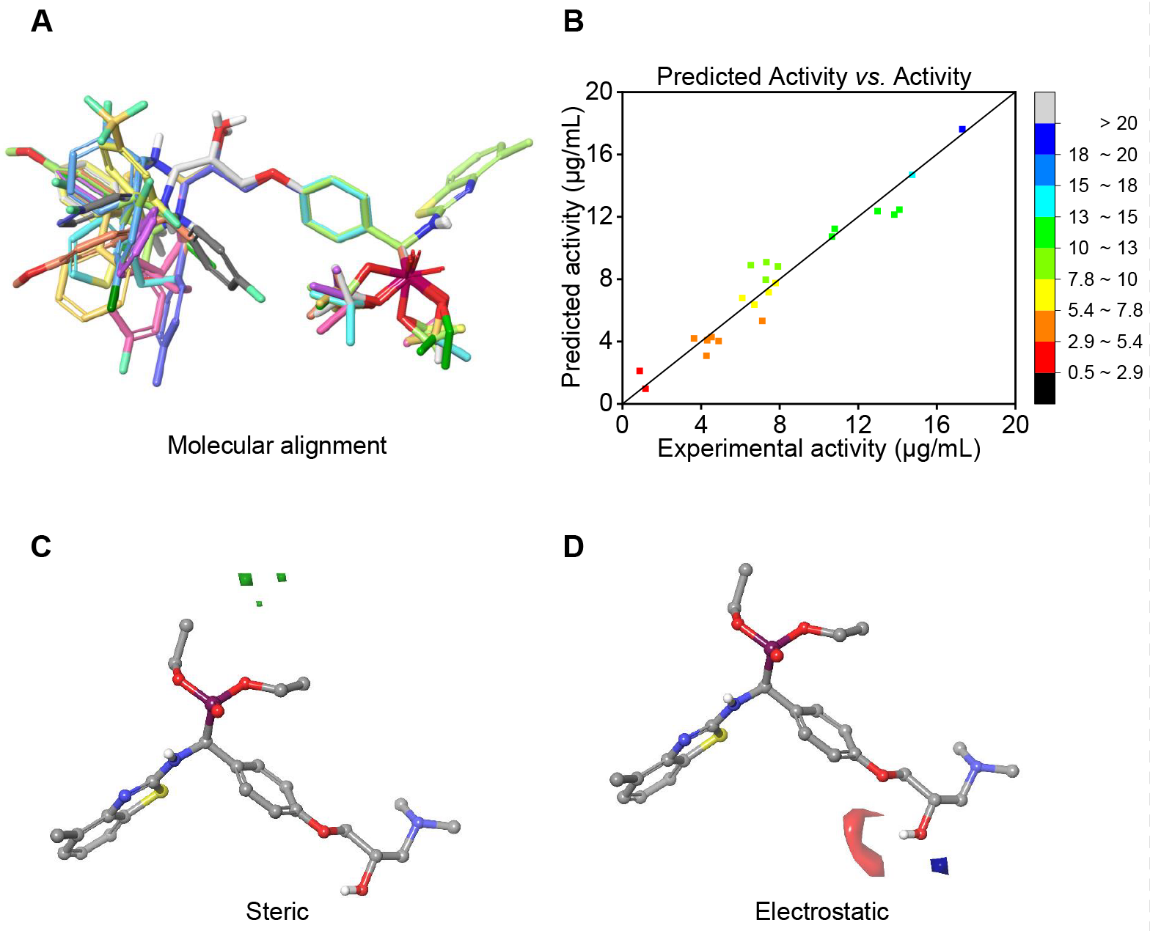


**Figure S2.** CoMFA contour maps with BtP27 inside the fields. (A) Molecular alignment of compounds BtP1-BtP30. (B) Plots of predicted versus experimental EC_50_ values against *Xoo* for CoMFA model. (C) Steric fields of CoMFA model, with green and yellow polyhedra indicating the regions where steric bulk would enhance and reduce the activity. (D) Electrostatic fields of CoMFA model, with blue and red polyhedra indicating the regions where positive and negative charges would enhance activity.


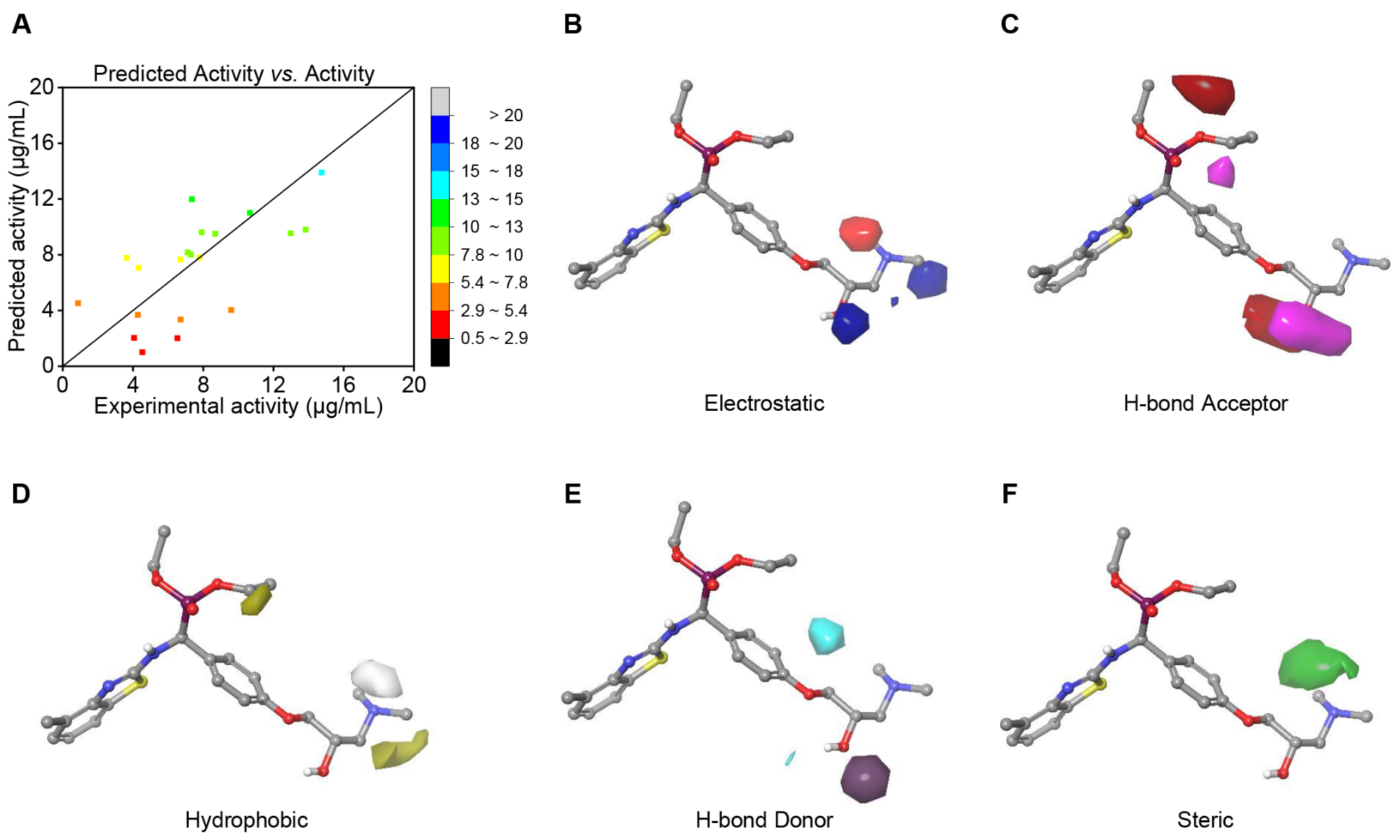


**Figure S3**. CoMSIA contour maps with BtP27 inside the fields. (A) Plots of predicted versus experimental EC_50_ values against *Xoo* for CoMSIA model. (B) Electrostatic fields of CoMSIA model, with blue and red polyhedra indicating the regions where positive and negative charges would enhance activity. (C) H-bond acceptor fields of CoMSIA model, with red contours indicating the regions where H-bond acceptor groups would enhance activity. (D) Hydrophobic fields of CoMSIA model, with yellow and white polyhedra indicating the regions where hydrophobicity and hydrophilicity would enhance activity. (E) H-bond donor fields of CoMSIA model, with purple contours indicating the regions where H-bond donor groups would enhance activity. (F) Steric fields of CoMSIA model, with green and yellow polyhedra indicating the regions where steric bulk would enhance and reduce the activity.


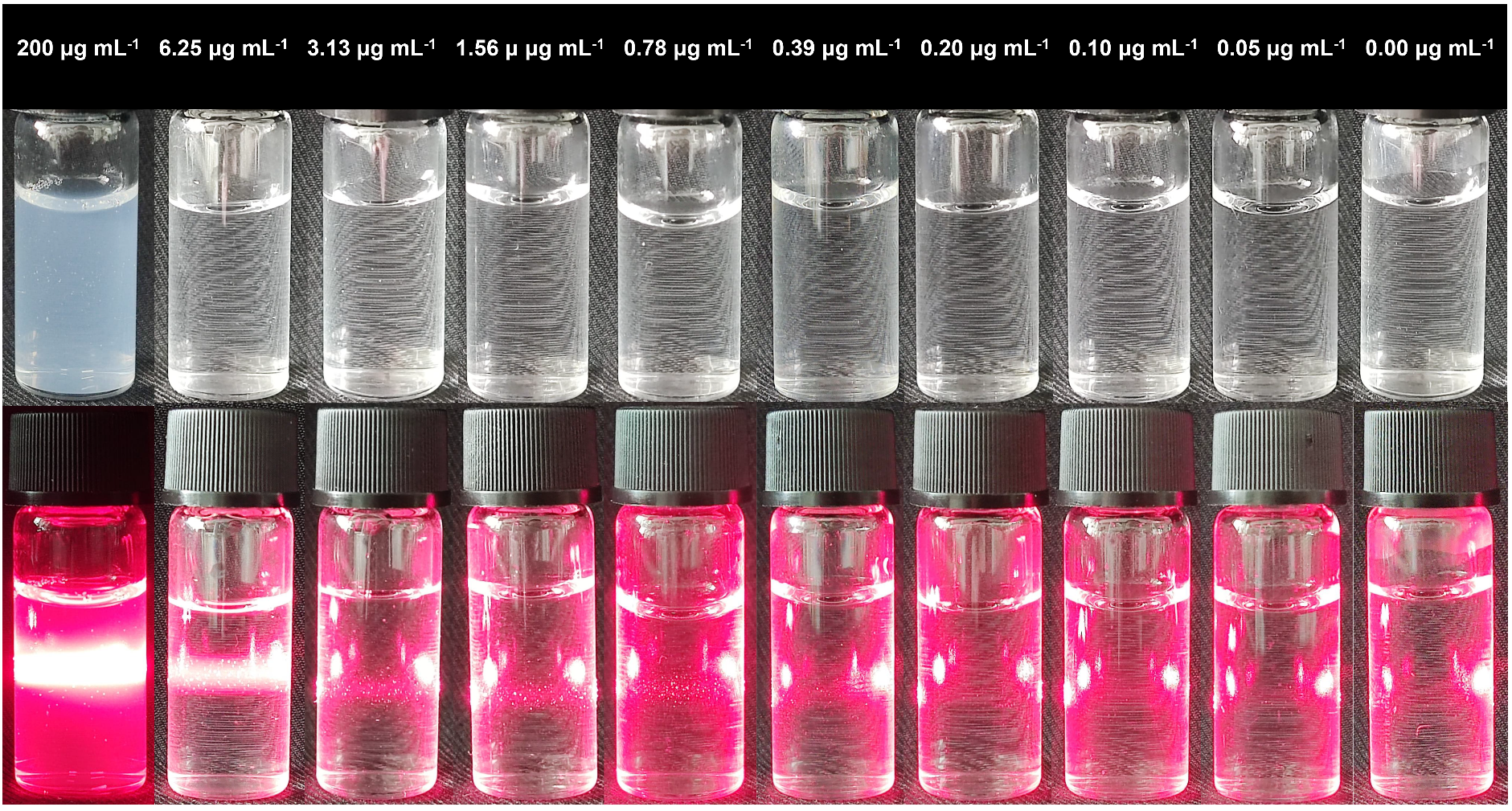


**Figure S4.** Photographic series of BtP27@*β*-CD aqueous solutions at varying concentrations and their Tyndall effects


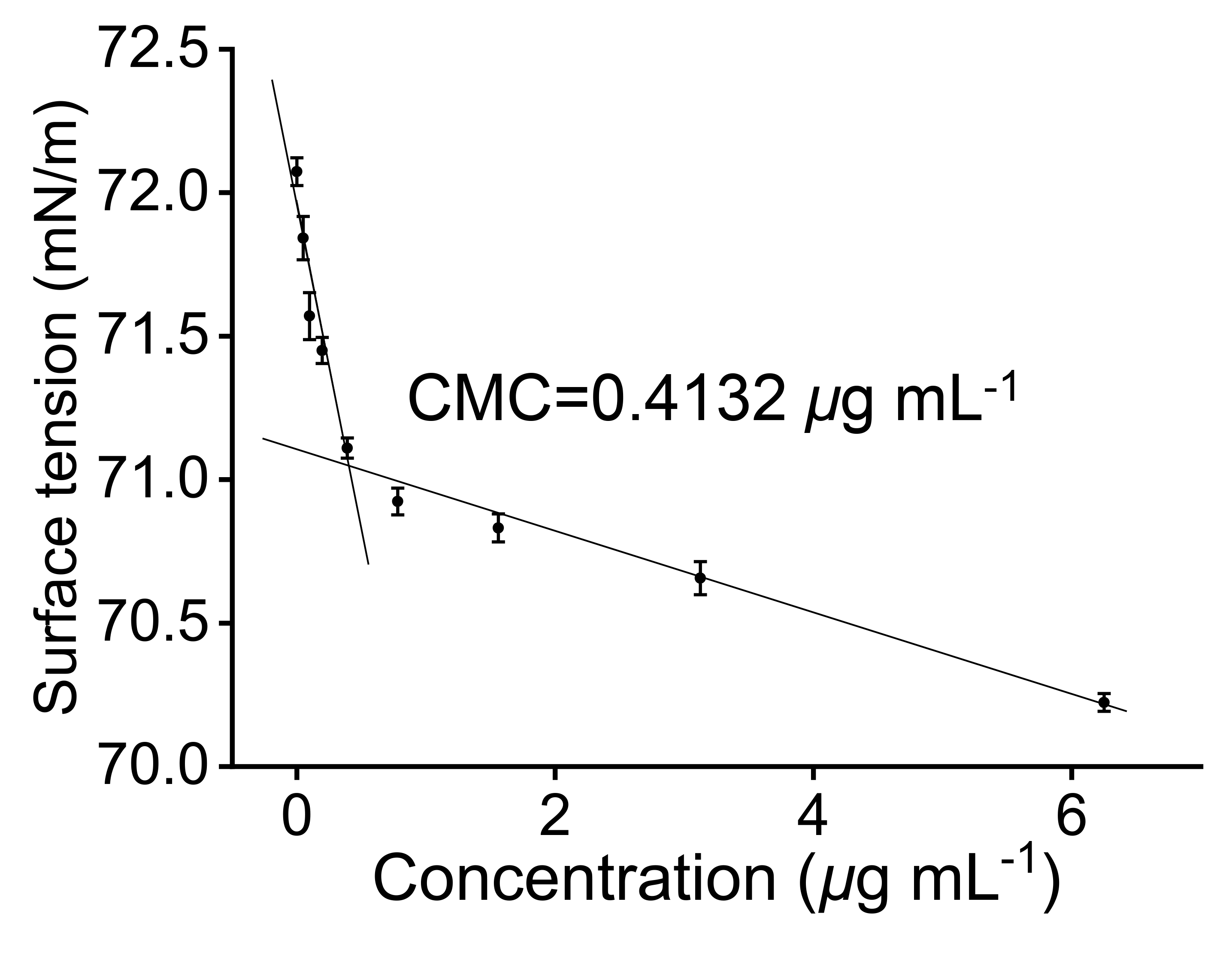


**Figure S5.** Changes in the surface tension of BtP27@*β*-CD at different concentrations. The critical micelle concentration (CMC) of BtP27@*β*-CD was determined by fitting the data.


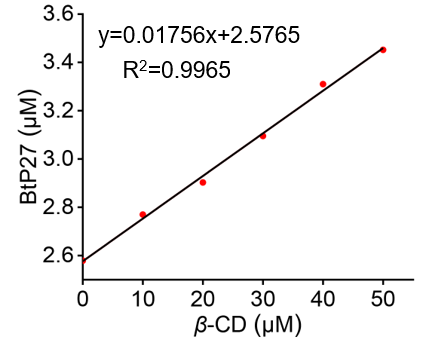


**Figure S6.** Phase solubility diagram of BtP27 and *β*-CD at 25 ℃.


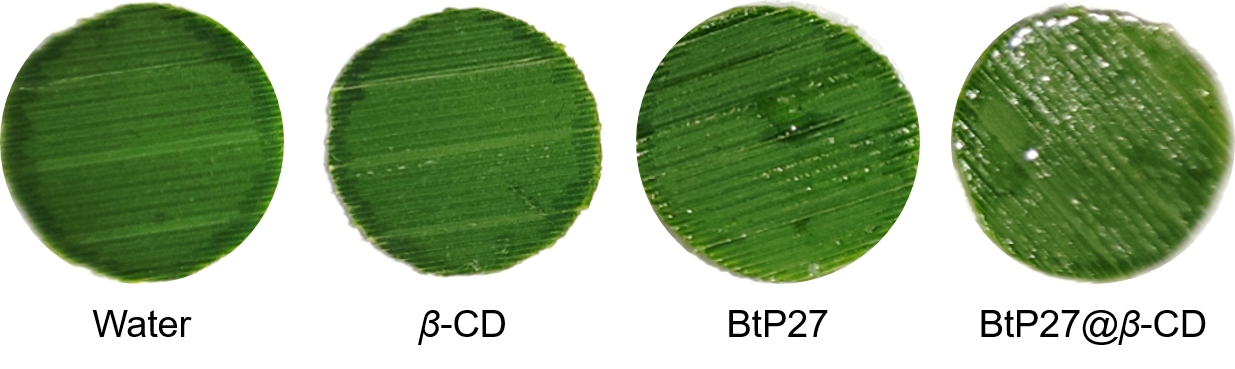


**Figure S7.** Images of rice leaf disks post-soaking in water, *β*-CD, BtP27, and BtP27@*β*-CD solutions

**Figure S8.** The maximum rebound heights of *β*-CD, BtP27, and BtP27@*β*-CD droplets (200 *μ*g mL⁻¹) dropped from a height of 10 cm above rice leaves were statistically analyzed, with water serving as the blank control. Statistically significant differences between the means were analyzed with one-way ANOVA, followed by the least significant difference (LSD) post-hoc test (n = 3; **p* < 0.05, ***p* < 0.01, ****p* < 0.001; n.s. = no signiﬁcance).


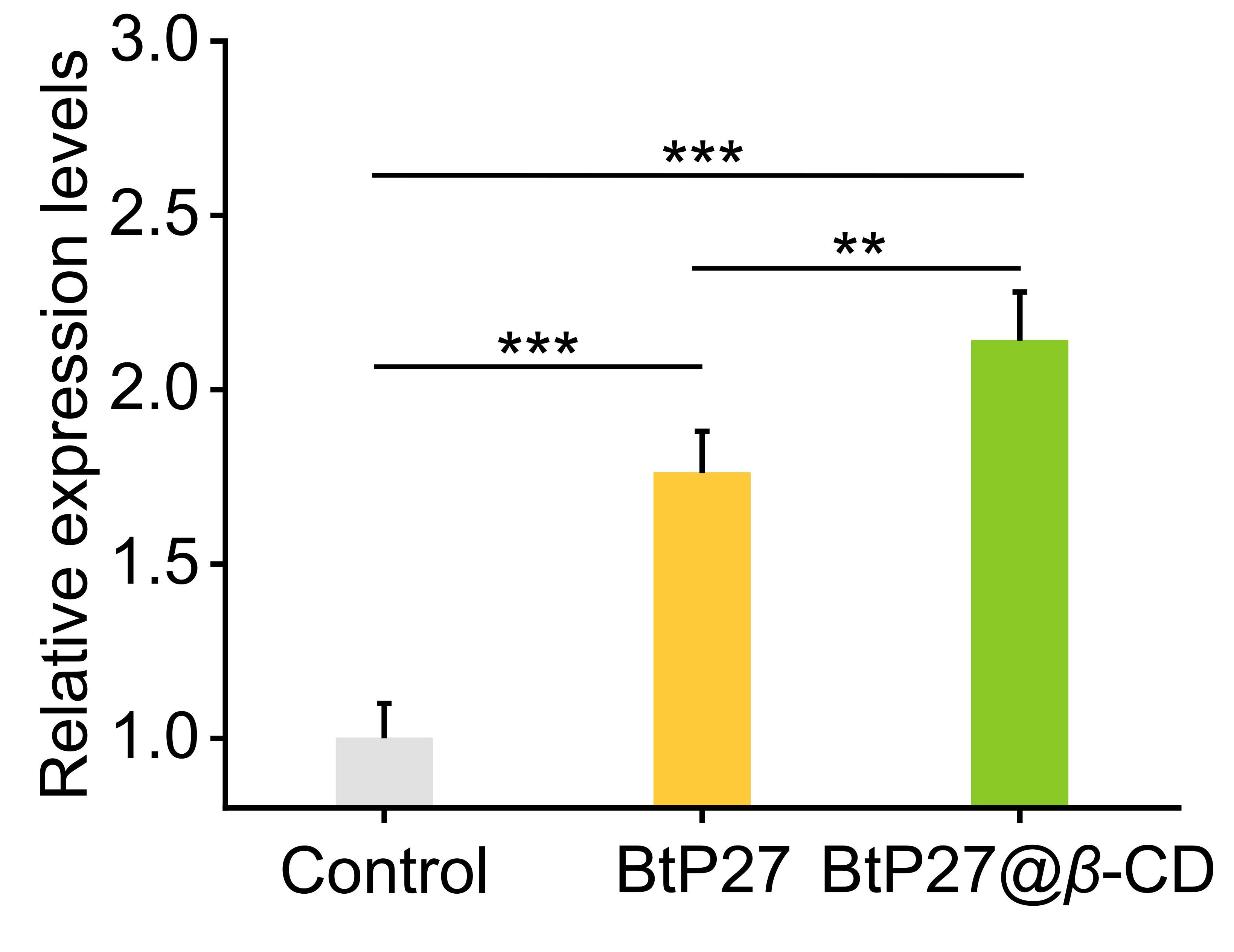


**Figure S9.** The expression levels of the *OsNPR1* gene in rice leaves, sprayed with BtP27@*β*-CD and BtP27 at a concentration of 200 *µ*g mL⁻¹, were measured after 24 h. OsActin was used as the reference gene. Statistically significant differences between the means were analyzed with one-way ANOVA, followed by the least significant difference (LSD) post-hoc test (n = 3; **p* < 0.05, ***p* < 0.01, ****p* < 0.001; n. s. = no signiﬁcance).


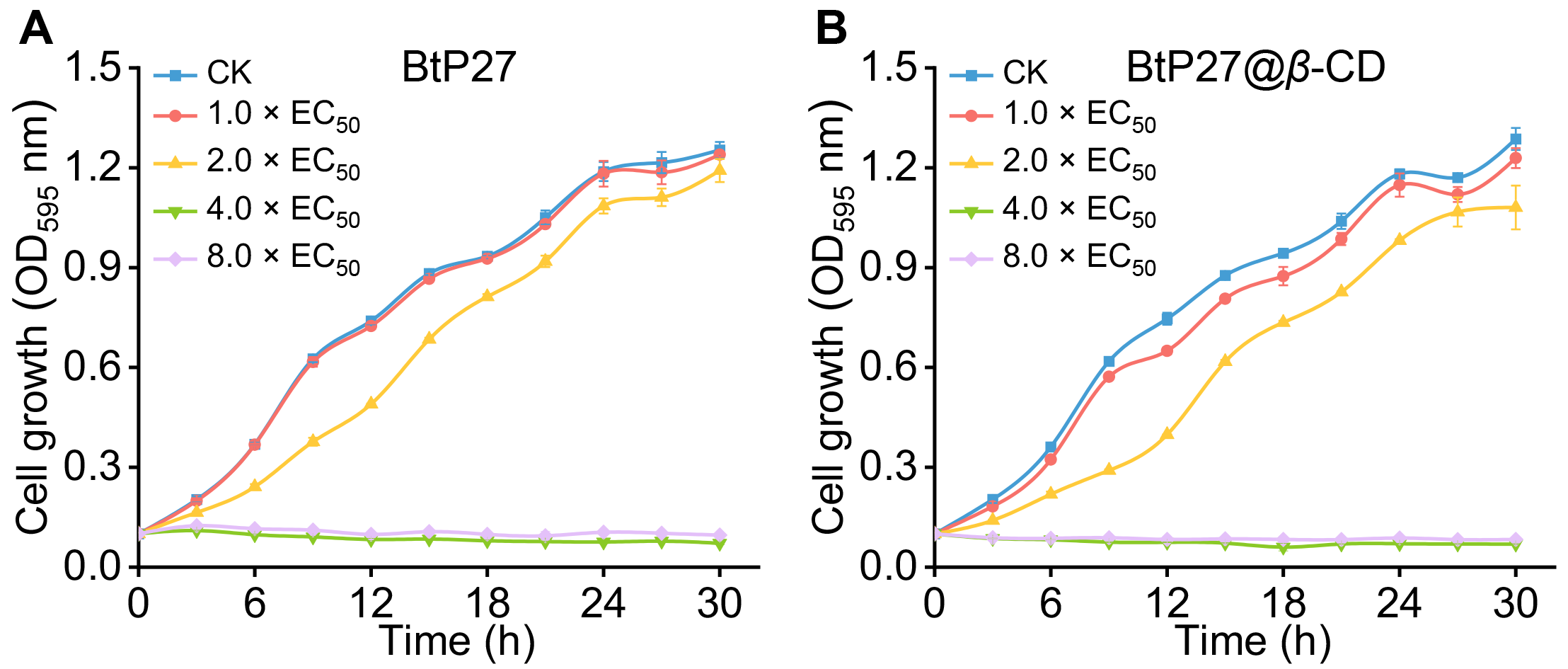


**Figure S10.** Growth curves of *Xoo* incubated with BtP27 (A) and BtP27@*β*-CD (B) at various concentrations (0-8.0 EC_50_) over 30 hours.


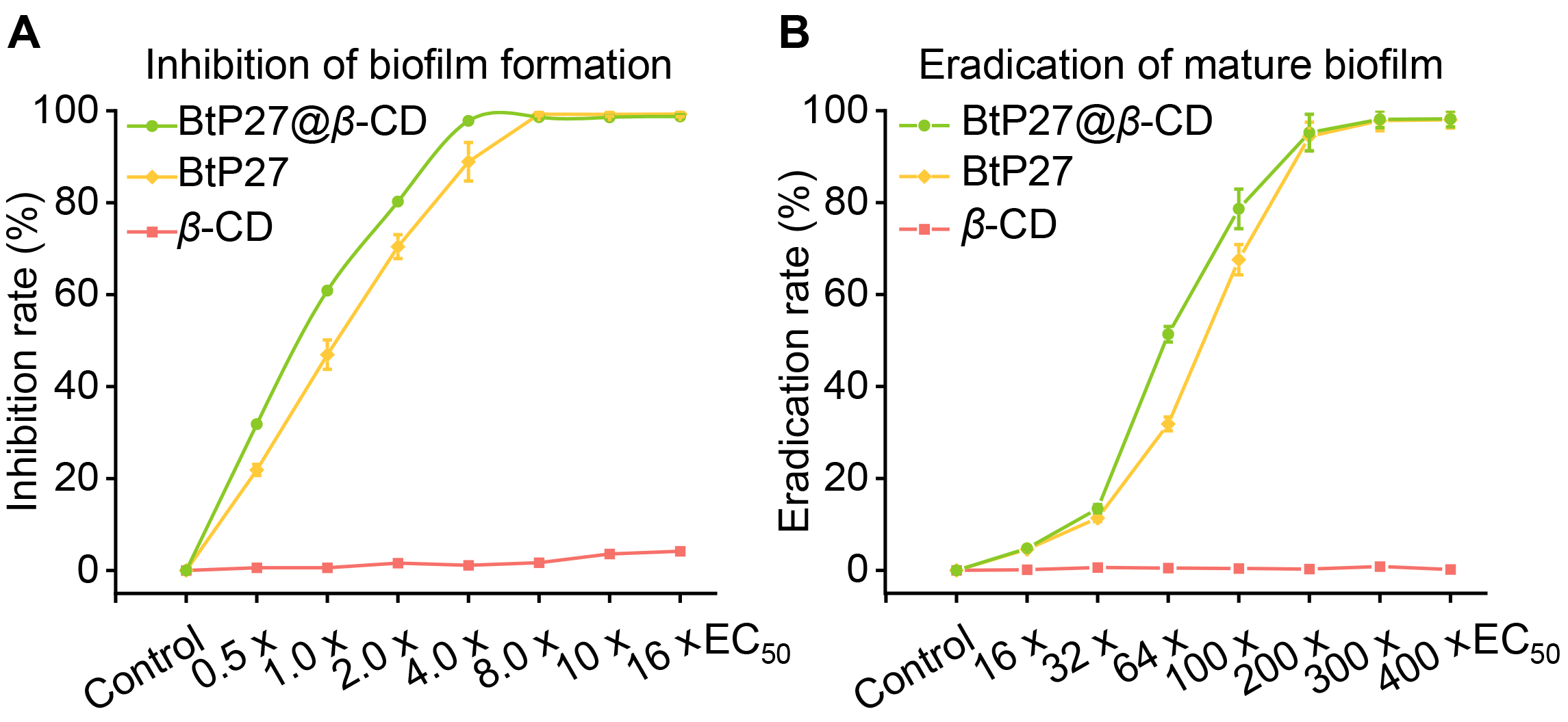


**Figure S11.** (A) Inhibition rates of biofilm formation in *Xoo* after 48-hour incubation with various concentrations of *β*-CD, BtP27, and BtP27@*β*-CD. (B) Eradication rates of mature biofilm in *Xoo* following incubation with different concentrations of *β*-CD, BtP27, and BtP27@*β*-CD.

**Figure S12.** Quantification of extracellular polysaccharide (EPS) secretion by *Xoo* after 48-hour treatment with varying concentrations of BtP27@*β*-CD, BtP27, and *β*-CD. Statistically significant differences between the means were analyzed with one-way ANOVA, followed by the least significant difference (LSD) post-hoc test (n = 3; **p* < 0.05, ***p* < 0.01, ****p* < 0.001; n.s. = no signiﬁcance).


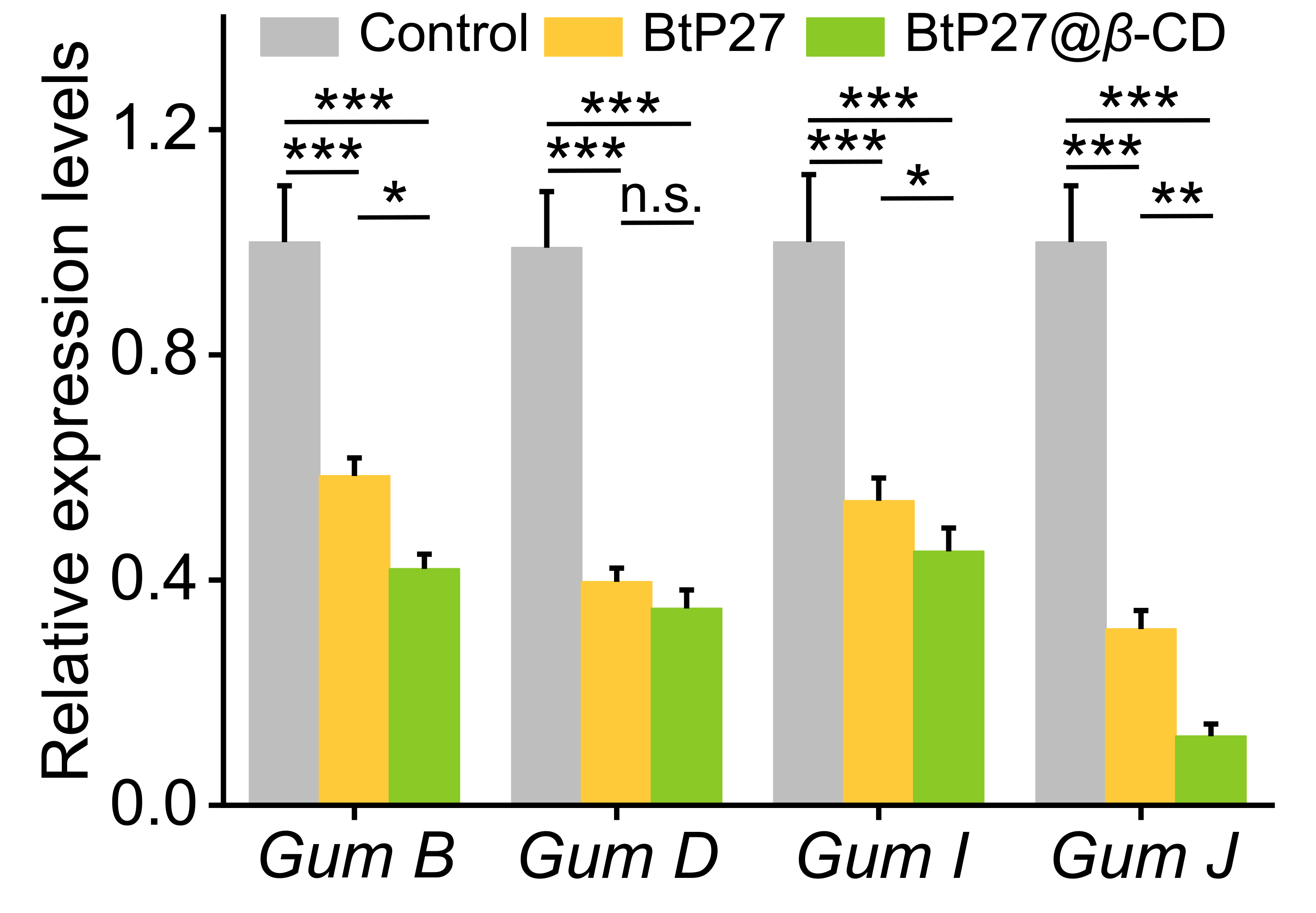


**Figure S13.** The expression levels of biofilm-related genes in *Xoo* treated with BtP27@*β*-CD and BtP27 at a concentration of 0.87 *µ*g mL⁻¹ (1×EC₅₀) was detected after 12 h. *gyrB* was used as the reference gene. Statistically significant differences between the means were analyzed with one-way ANOVA, followed by the least significant difference (LSD) post-hoc test (n = 3; **p* < 0.05, ***p* < 0.01, ****p* < 0.001; n.s. = no signiﬁcance).


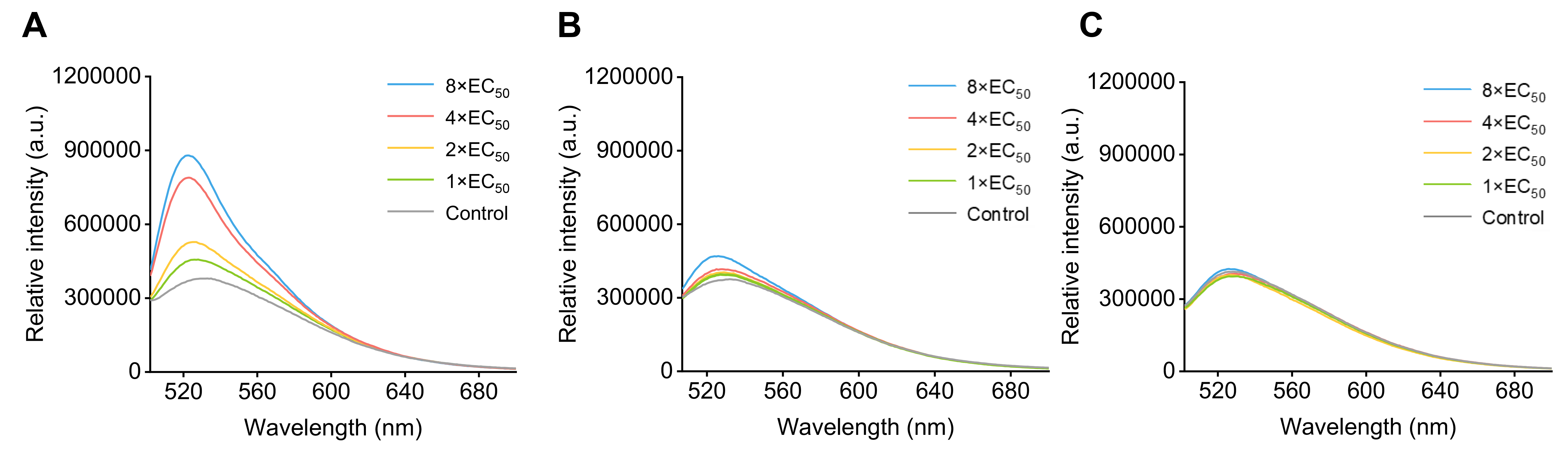


**Figure S14.** Reactive oxygen species (ROS) fluorescence intensity in *Xoo* treated with specified concentrations of BtP27@*β*-CD (A), BtP27 (B), and *β*-CD (C) was measured using an ROS detection kit. The excitation wavelength was set to 488 nm.


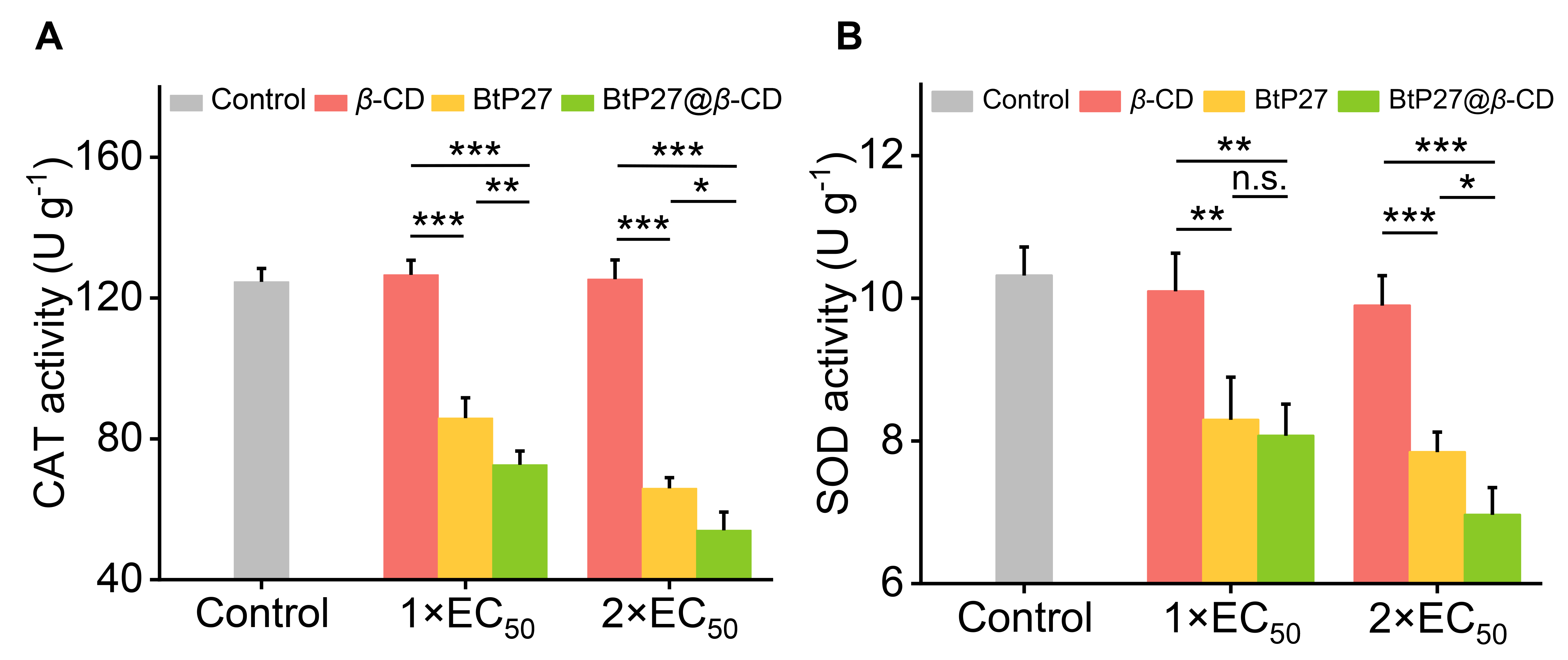


**Figure S15.** Statistical analysis of the activity of catalase (CAT) (A) and superoxide dismutase (SOD) (B) in *Xoo* treated with different concentrations of *β*-CD, BtP27, and BtP27@*β*-CD. Statistically comparisons were analyzed with one-way ANOVA, followed by the least significant difference (LSD) post-hoc test (n = 3; **p* < 0.05, ***p* < 0.01, ****p* < 0.001; n.s. = no signiﬁcance).

**Figure S16.** HRMS spectrum of BtP27@*β*-CD.

**Figure S17.** HRMS spectrum of BtP7@*β*-CD.

**Figure S18.** HRMS spectrum of BtP11@*β*-CD.

**Figure S19.** HRMS spectrum of BtP20@*β*-CD.


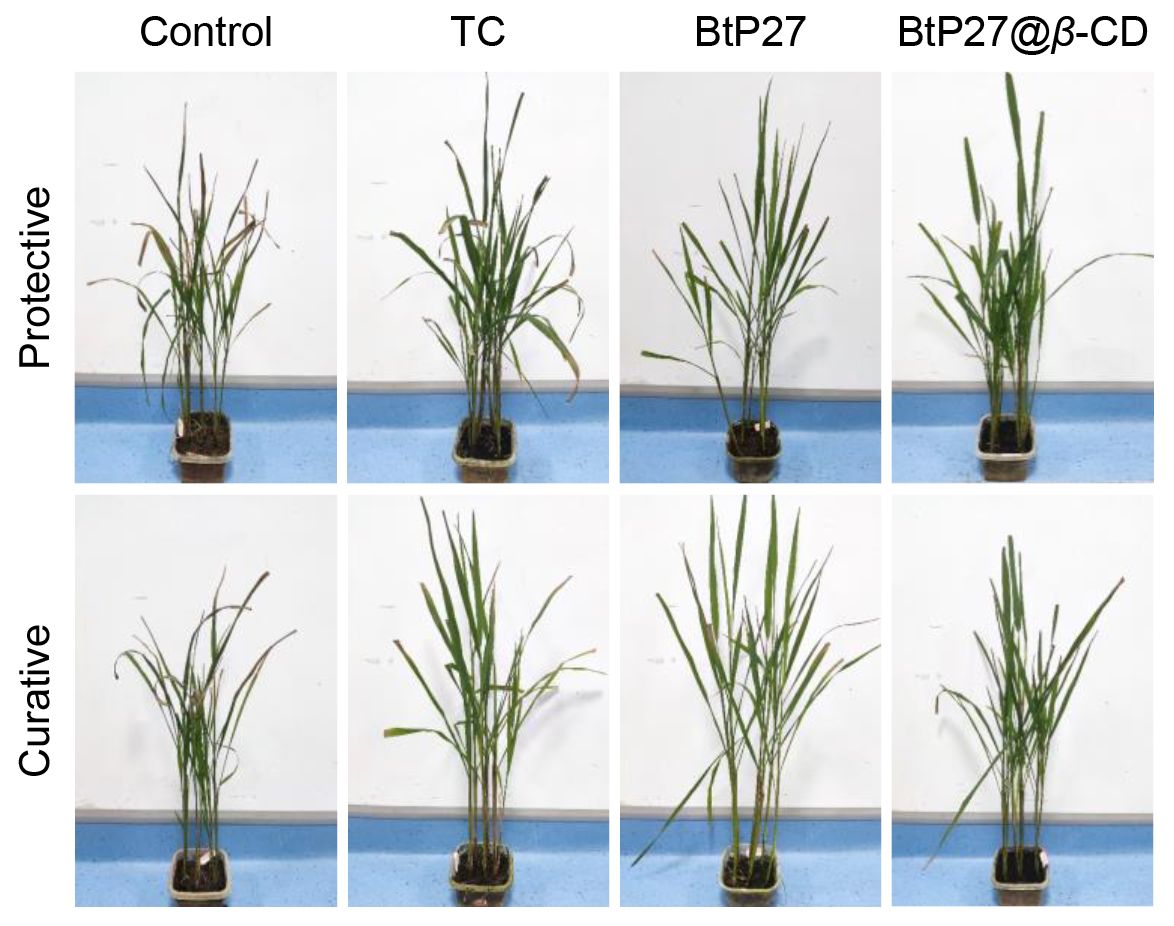


**Figure S20.** Photographs of control efficacies of TC, BtP27, and BtP27@*β*-CD against *Xoo*-induced rice bacterial leaf blight at 200 *μ*g mL^-1^, with Water as the control.


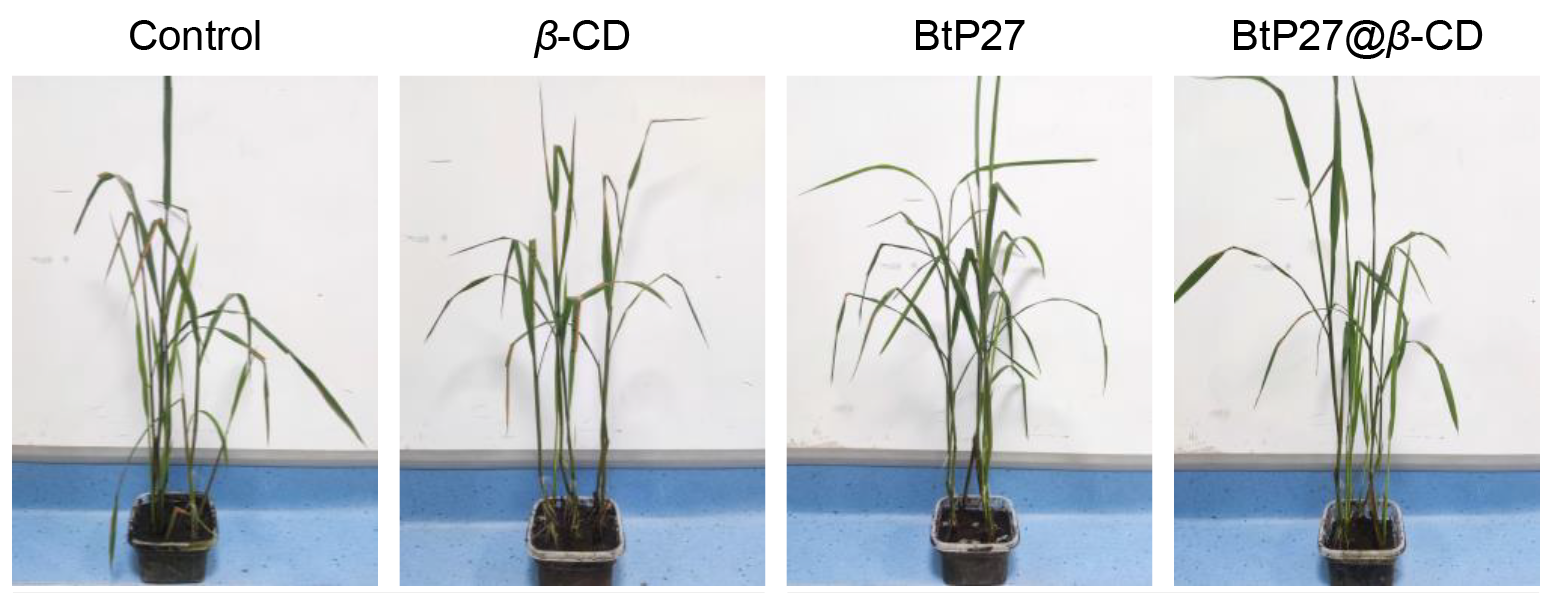


**Figure S21.** Photographs of protective efficacies of *β*-CD, BtP27, and BtP27@*β*-CD (200 *μ*g mL^-1^) against *Xoo*-induced rice bacterial leaf blight post-uptake and translocation, with water as the control.


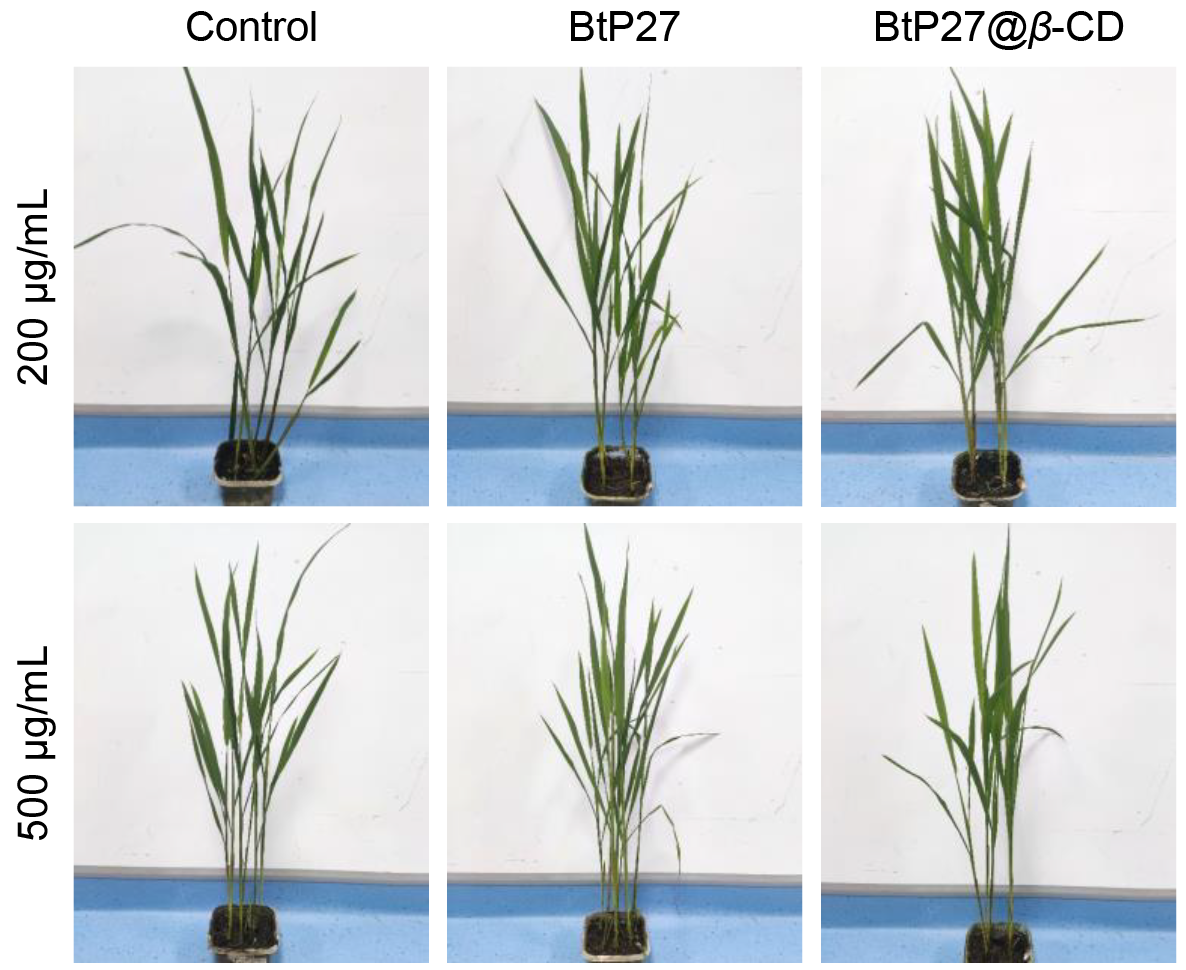


**Figure S22.** Photographs of phytotoxicity tests on rice leaves treated with BtP27 and BtP27@*β*-CD at 200 and 500 *μ*g mL^-1^, 7 days post-spraying, with Water as the control


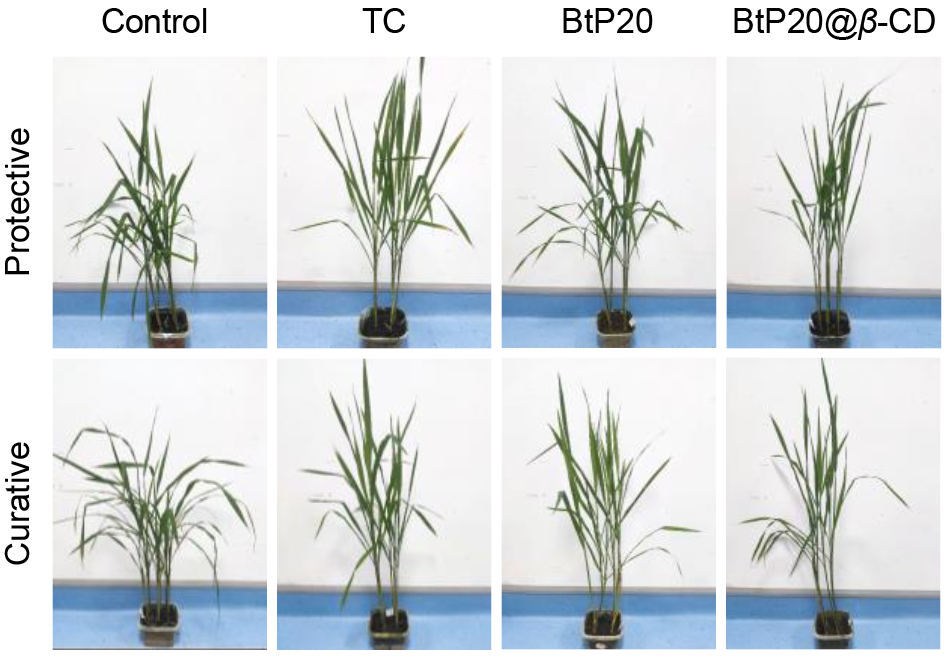


**Figure S23.** Photographs of control efficacies of TC, BtP20, and BtP20@*β*-CD against *Xoc*-induced rice bacterial leaf streak at 200 *μ*g mL^-1^, with Water as the control.


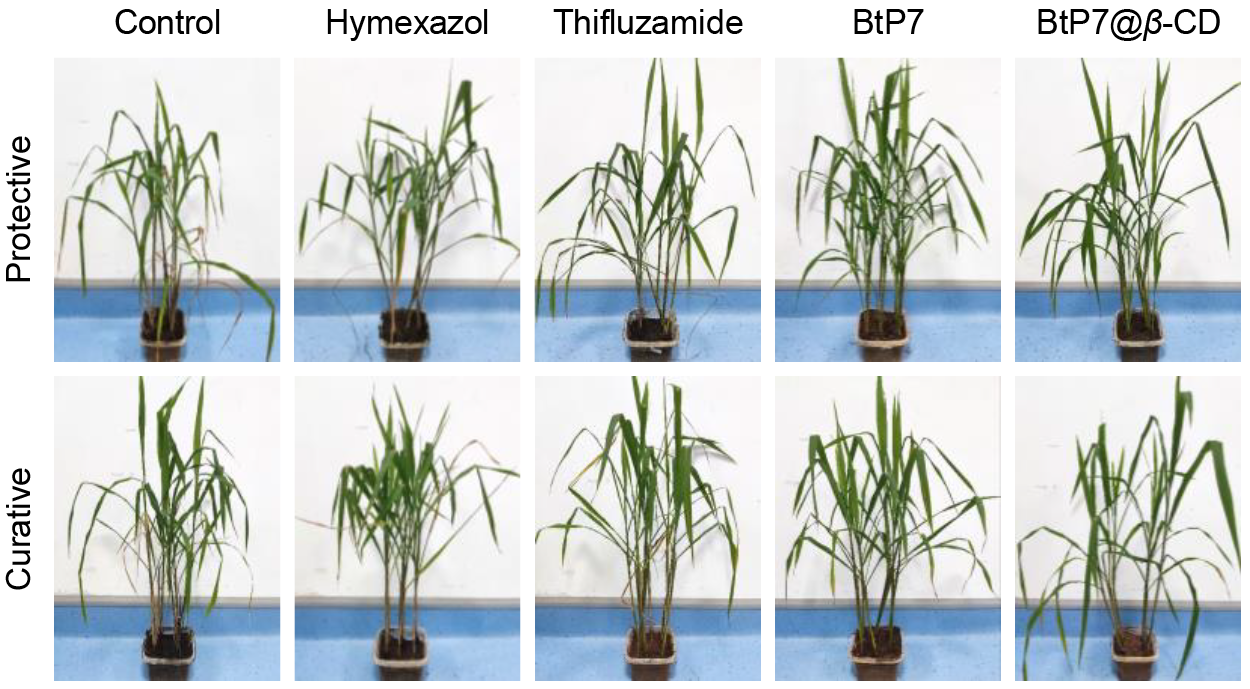


**Figure S24.** Photographs of control efficacies of hymexazol, thifluzamide, BtP7, and BtP7@*β*-CD against *Rs*-induced rice sheath blight at 200 *μ*g mL^-1^, with Water as the control.


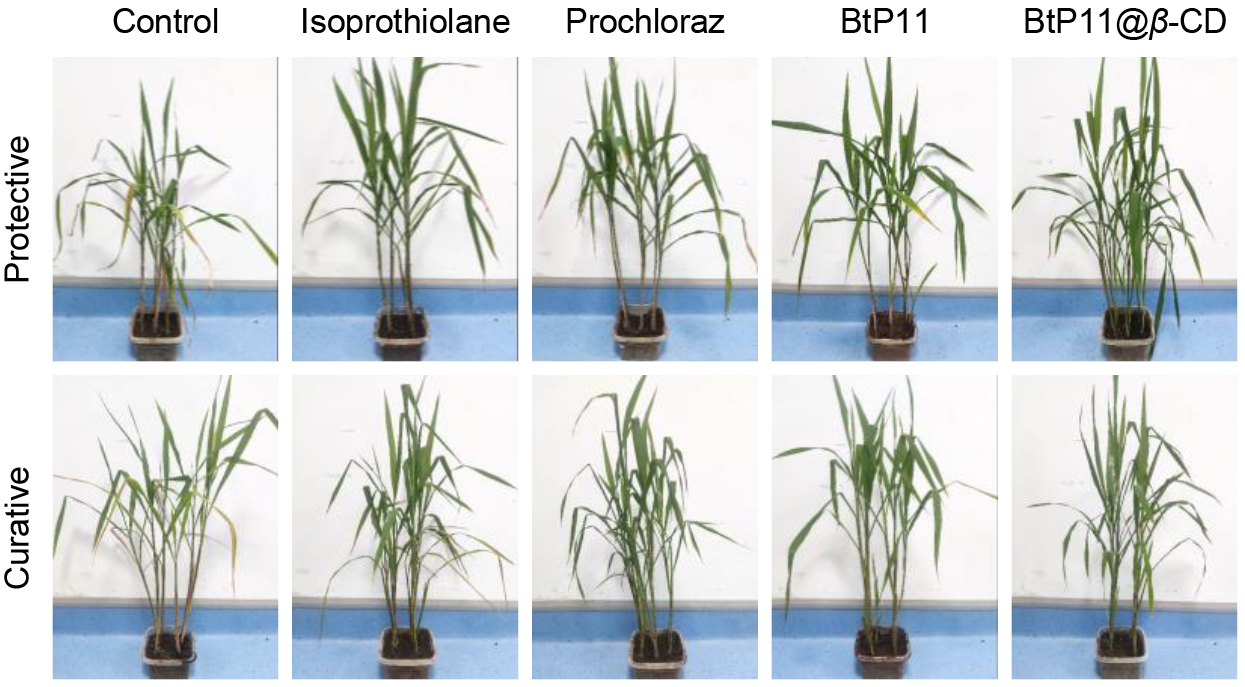


**Figure S25.** Photographs of control efficacies of isoprothiolane, prochloraz, BtP11, and BtP11@*β*-CD against *Mo*-induced rice blast at 200 *μ*g mL^-1^, with Water as the control.

# 4. Characterization of target compounds

**BtP1,** *diethyl ((4-(3-(dimethylamino)-2-hydroxypropoxy)phenyl)((4-methylbenzo[d]thiazol-2-yl)amino)methyl)phosphonate*. A colorless oil, yield 20.3%; ^1^H NMR (400 MHz, CDCl_3_) δ 7.46 (d, *J* = 7.0 Hz, 2H, Benzene-H), 7.31 (d, *J* = 7.6 Hz, 1H, Benzothiazole-H), 7.01 (d, *J* = 7.2 Hz, 1H, Benzothiazole-H), 6.95 – 6.86 (m, 1H, Benzothiazole-H), 6.82 (d, *J* = 7.6 Hz, 2H, Benzene-H), 5.50 (d, *J* = 21.8 Hz, 1H, PCH), 4.31 – 4.06 (m, 3H, CHOH, OCH_2_CH_3_), 4.00 – 3.83 (m, 3H, OCH_2_CH_3,_ OCH_2_CH), 3.77 – 3.60 (m, 1H, OCH_2_CH), 2.79 (d, *J* = 69.7 Hz, 2H, NCH_2_), 2.53 (s, 6H, 2NCH_3_), 2.50 (s, 3H, Benzothiazole-CH_3_), 1.22 (t, *J* = 7.0 Hz, 3H, OCH_2_CH_3_), 1.08 (t, *J* = 7.0 Hz, 3H, OCH_2_CH_3_); ^13^C NMR (101 MHz, CDCl_3_) δ 164.94, 158.32, 150.94, 130.82, 129.67, 128.92, 127.68, 126.49, 121.60, 118.18, 114.61, 69.84, 65.06, 63.64 (d, ^2^*J_C-P_* = 7.0 Hz), 60.86, 54.97 (d, ^1^*J_C-P_* = 156.7 Hz), 44.68, 18.36, 16.38 (dd, ^3^*J_C-P_* = 18.7, 5.8 Hz); ^31^P NMR (162 MHz, CDCl_3_) δ 21.03; HRMS (ESI) [M+H]^+^ calcd for C_24_H_35_O_5_N_3_PS:508.2030, found: 508.2021.

**BtP2,** *diethyl ((4-(3-(diethylamino)-2-hydroxypropoxy)phenyl)((4-methylbenzo[d]thiazol-2-yl)amino)methyl)phosphonate*. A colorless oil, yield 50.5%; ^1^H NMR (500 MHz, CDCl_3_) δ 7.48 (d, *J* = 6.8 Hz, 2H, Benzene-H), 7.35 (d, *J* = 7.7 Hz, 1H, Benzothiazole-H), 7.06 (d, *J* = 7.4 Hz, 1H, Benzothiazole-H), 6.95 (t, *J* = 7.6 Hz, 1H, Benzothiazole-H), 6.87 (d, *J* = 8.7 Hz, 2H, Benzene-H), 5.37 (d, *J* = 22.0 Hz, PCH), 4.24 – 4.11 (m, 3H, CHOH, OCH_2_CH_3_), 4.06 – 3.95 (m, 2H, OCH_2_CH_3_), 3.94 – 3.87 (m, 1H, OCH_2_CH), 3.81 – 3.72 (m, 1H, OCH_2_CH), 3.01 – 2.69 (m, 6H, 3NCH_2_), 2.52 (s, 3H, Benzothiazole-CH_3_), 1.28 (t, *J* = 7.1 Hz, 3H, OCH_2_CH_3_), 1.18 (t, *J* = 7.2 Hz, 6H, 2NCH_2_CH_3_), 1.14 (t, *J* = 7.1 Hz, 3H, OCH_2_CH_3_); ^13^C NMR (126 MHz, CDCl_3_) δ 165.19, 158.65, 151.22, 131.03, 129.86, 129.37, 127.87, 126.78, 121.93, 118.42, 114.78, 70.09, 65.51, 63.78 (dd, ^2^*J_C-P_* = 20.0, 6.9 Hz), 56.73, 55.67 (d, ^1^*J_C-P_* = 155.8 Hz), 48.00, 18.57, 16.63 (dd, ^3^*J_C-P_* = 22.8, 5.7 Hz), 10.82; ^31^P NMR (202 MHz, CDCl_3_) δ 21.61; HRMS (ESI) [M+H]^+^ calcd for C_26_H_39_O_5_N_3_PS:536.2343, found: 536.2333.

**BtP3,** *diethyl ((4-(2-hydroxy-3-(pyrrolidin-1-yl)propoxy)phenyl)((4-methylbenzo[d]thiazol-2-yl)amino)methyl)phosphonate*. A yellow oil, yield 78.9%; ^1^H NMR (400 MHz, CDCl_3_) δ 7.49 (d, *J* = 7.9 Hz, 2H, Benzene-H), 7.31 (d, *J* = 7.7 Hz, 1H, Benzothiazole-H), 7.01 (d, *J* = 7.3 Hz, 1H, Benzothiazole-H), 6.90 (t, *J* = 7.6 Hz, 1H, Benzothiazole-H), 6.84 (d, *J* = 8.5 Hz, 2H, Benzene-H), 5.57 (d, *J* = 21.3 Hz, 1H, PCH), 4.42 – 4.08 (m, 3H, CHOH, OCH_2_CH_3_), 4.02 – 3.86 (m, 3H, OCH_2_CH_3,_ OCH_2_CH), 3.83 – 3.70 (m, 1H, OCH_2_CH), 3.25 – 2.89 (m, 6H, 3NCH_2_), 2.49 (s, 3H, Benzothiazole-CH_3_), 1.82 (s, 4H, pyrrolidine-H), 1.24 (t, *J* = 7.1 Hz, 3H, OCH_2_CH_3_), 1.12 (t, *J* = 7.1 Hz, 3H, OCH_2_CH_3_); ^13^C NMR (101 MHz, CDCl_3_) δ 165.09, 158.32, 151.08, 130.98, 129.91, 128.99, 128.15, 126.59, 121.69, 118.26, 114.67, 69.92, 65.89, 63.75 (dd, ^2^*J_C-P_* = 7.0, 4.1 Hz), 58.45, 54.94 (d, ^1^*J_C-P_* = 156.3 Hz), 54.84, 23.18, 18.48, 16.56 (dd, ^3^*J_C-P_* = 17.6, 5.7 Hz); ^31^P NMR (162 MHz, CDCl_3_) δ 21.21; HRMS (ESI) [M+H]^+^ calcd for C_26_H_37_O_5_N_3_PS:543.2186, found: 543.2177.

**BtP4,** *diethyl ((4-(2-hydroxy-3-(piperidin-1-yl)propoxy)phenyl)((4-methylbenzo[d]thiazol-2-yl)amino)methyl)phosphonate*. A yellow oil, yield 49.7%;^1^H NMR (400 MHz, CDCl_3_) δ 7.43 (d, *J* = 6.8 Hz, 2H, Benzene-H), 7.28 (d, *J* = 7.7 Hz, 1H, Benzothiazole-H), 6.99 (d, *J* = 7.3 Hz, 1H, Benzothiazole-H), 6.87 (t, *J* = 7.6 Hz, 1H, Benzothiazole-H), 6.80 (d, *J* = 8.6 Hz, 2H, Benzene-H), 5.36 (dd, *J* = 21.9, 4.0 Hz, 1H, PCH), 4.21 – 4.00 (m, 3H, CHOH, OCH_2_CH_3_), 3.96 – 3.83 (m, 3H, OCH_2_CH_3_, OCH_2_CH), 3.77 – 3.62 (m, 1H, OCH_2_CH), 2.59 (s, 2H, NCH_2_), 2.53 – 2.44 (m, 5H, Benzothiazole-CH3, NCH_2_), 2.39 (s, 2H, NCH_2_), 1.61 – 1.51 (m, 4H, Piperidine-H), 1.46 – 1.31 (m, 2H, Piperidine-H), 1.20 (t, *J* = 7.1 Hz, 3H, OCH_2_CH_3_), 1.07 (t, *J* = 7.1 Hz, 3H, OCH_2_CH_3_); ^13^C NMR (101 MHz, CDCl_3_) δ 165.01, 158.65, 151.04, 130.86, 129.62, 129.06, 127.57, 126.48, 121.60, 118.16, 114.58, 70.29, 65.16, 63.55 (dd, ^2^*J_C-P_* = 19.8, 7.0 Hz), 61.22, 55.34 (d, ^1^*J_C-P_* = 155.6 Hz), 54.75, 25.61, 23.89, 18.35, 16.42 (dd, ^3^*J_C-P_* = 18.4, 5.7 Hz); ^31^P NMR (162 MHz, CDCl_3_) δ 21.181; HRMS (ESI) [M+H]^+^ calcd for C_27_H_39_O_5_N_3_PS:548.2343, found: 548.2330.

**BtP5,** *diethyl ((4-(2-hydroxy-3-morpholinopropoxy)phenyl)((4-methylbenzo[d]thiazol-2-yl)amino)methyl)phosphonate*. A colorless oil, yield 31.4%;^1^H NMR (400 MHz, CDCl_3_) δ 7.80 (s, 1H, NH), 7.57 (d, *J* = 6.9 Hz, 2H, Benzene-H), 7.37 (d, *J* = 7.7 Hz, 1H, Benzothiazole-H), 7.08 (d, *J* = 7.3 Hz, 1H, Benzothiazole-H), 6.96 (t, *J* = 7.6 Hz, 1H, Benzothiazole-H), 6.90 (d, *J* = 8.6 Hz, 2H, Benzene-H), 5.56 (d, *J* = 21.9 Hz, 1H, PCH), 4.31 – 4.09 (m, 3H, CHOH, OCH_2_CH_3_), 4.07 – 3.89 (m, 3H, OCH_2_CH_3_, OCH_2_CH), 3.86 – 3.76 (m, 1H, OCH_2_CH), 3.75 – 3.66 (m, 4H, Morpholine-H), 2.65 (dd, *J* = 11.0, 4.7 Hz, 2H, Morpholine-H), 2.60 – 2.51 (m, 5H, Benzothiazole-CH_3_, NCH_2_), 2.48 (dd, *J* = 7.7, 5.0 Hz, 2H, Morpholine-H), 1.30 (t, *J* = 7.1 Hz, 3H, OCH_2_CH_3_), 1.17 (t, *J* = 7.1 Hz, 3H, OCH_2_CH_3_); ^13^C NMR (101 MHz, CDCl_3_) δ 164.91, 158.56, 151.05, 130.88, 129.81, 128.96, 127.82, 126.45, 121.53, 118.13, 114.53, 70.22, 66.96, 65.49, 63.57 (dd, ^2^*J_C-P_* = 17.1, 7.0 Hz), 61.07, 55.12 (d, ^1^*J_C-P_* = 156.0 Hz), 53.79, 18.39, 16.44 (dd, ^3^*J_C-P_* = 18.5, 5.7 Hz); ^31^P NMR (162 MHz, CDCl_3_) δ 21.26; HRMS (ESI) [M+H]^+^ calcd for C_26_H_37_O_6_N_3_PS: 550.2135, found: 550.2122.

**BtP6,** *diethyl ((4-(2-hydroxy-3-thiomorpholinopropoxy)phenyl)((4-methylbenzo[d]thiazol-2-yl)amino)methyl)phosphonate*. A yellow oil, yield 63.1%; ^1^H NMR (400 MHz, CDCl_3_) 7.56 (d, *J* = 7.3 Hz, 2H, Benzene-H), 7.37 (d, *J* = 7.8 Hz, 1H, Benzothiazole-H), 7.08 (d, *J* = 7.3 Hz, 1H, Benzothiazole-H), 6.96 (t, *J* = 7.6 Hz, 1H, Benzothiazole-H), 6.90 (d, *J* = 8.5 Hz, 2H, Benzene-H), 5.53 (d, *J* = 21.9 Hz, 1H, PCH), 4.32 – 4.16 (m, 2H, OCH_2_CH_3_), 4.09 – 3.99 (m, 2H, OCH_2_CH_3_), 3.94 (d, *J* = 4.8 Hz, 2H, OCH_2_CH), 3.87 – 3.73 (m, 1H, OCH_2_CH), 2.96 – 2.83 (m, 2H, NCH_2_), 2.69 (dd, *J* = 16.8, 10.2 Hz, 6H, Thiomorpholine-H), 2.60 – 2.45 (m, 5H, Benzothiazole-H, Thiomorpholine-H), 1.30 (t, *J* = 7.0 Hz, 3H, OCH_2_CH_3_), 1.17 (t, *J* = 7.0 Hz, 3H, OCH_2_CH_3_); ^13^C NMR (101 MHz, CDCl_3_) δ 165.01, 158.57, 151.05, 130.89, 129.79, 128.99, 127.79, 126.46, 121.55, 118.14, 114.55, 70.16, 65.46, 63.57 (dd, ^2^*J_C-P_* = 17.7, 7.0 Hz), 61.20, 55.32, 55.17 (d, ^1^*J_C-P_* = 156.0 Hz), 27.99, 18.38, 16.44 (dd, ^3^*J_C-P_* = 18.5, 5.7 Hz); ^31^P NMR (162 MHz, CDCl_3_) δ 21.34; HRMS (ESI) [M+H]^+^ calcd for C_9_H_18_ON_5_S: 244.1227, found: 244.1219.

**BtP7,** *diethyl ((4-(3-(benzyl(methyl)amino)-2-hydroxypropoxy)phenyl)((4-methylbenzo[d]thiazol-2-yl)amino)methyl)phosphonate*. A yellow oil, yield 23.3%; ^1^H NMR (400 MHz, CDCl_3_) δ 7.43 (d, *J* = 6.9 Hz, 2H, Benzene-H), 7.27 (d, *J* = 7.6 Hz, 1H, Benzothiazole-H), 7.23 – 7.14 (m, 5H, Benzylamine-H), 6.98 (d, *J* = 7.3 Hz, 1H, Benzothiazole-H), 6.86 (t, *J* = 7.6 Hz, 1H, Benzothiazole-H), 6.78 (d, *J* = 8.6 Hz, 1H, Benzene-H), 5.37 (d, *J* = 22.0 Hz, 1H, PCH), 4.20 – 3.89 (m, 4H, 2OCH_2_CH_3_), 3.84 (d, *J* = 5.0 Hz, 2H, OCH_2_CH), 3.75 – 3.62 (m, 1H, CHOH), 3.50 (dd, *J* = 59.3, 13.0 Hz, 2H, , Benzylamine-CH_2_), 2.55 (dd, *J* = 12.2, 9.7 Hz, 1H, NHCH_2_CH), 2.45 (s, 3H, Benzothiazole-CH_3_), 2.42 (dd, *J* = 12.4, 4.1 Hz, 1H, NHCH_2_CH), 2.18 (s, 3H, NCH_3_), 1.19 (t, *J* = 7.1 Hz, 3H, OCH_2_CH_3_), 1.06 (t, *J* = 7.1 Hz, 3H, OCH_2_CH_3_); ^13^C NMR (101 MHz, CDCl_3_) δ 163.97, 157.59, 149.97, 137.09, 129.79, 128.56, 128.01, 127.97, 127.33, 126.46, 126.27, 125.41, 120.51, 117.08, 113.48, 69.21, 65.03, 62.49 (dd, ^2^*J_C-P_* = 21.8, 7.0 Hz), 61.46, 58.45, 54.25 (d, ^1^*J_C-P_* = 155.7 Hz), 41.11, 17.30, 15.35 (dd, ^3^*J_C-P_* = 18.4, 5.7 Hz); ^31^P NMR (162 MHz, CDCl_3_) δ 21.17; HRMS (ESI) [M+H]^+^ calcd for C_30_H_39_O_5_N_3_PS: 584.2343, found: 548.2333.

**BtP8,** *diethyl ((4-(3-((2-fluorobenzyl)(methyl)amino)-2-hydroxypropoxy)phenyl)((4-methylbenzo[d]thiazol-2-yl)amino)methyl)phosphonate*. A yellow oil, yield 61.8%; ^1^H NMR (500 MHz, CDCl_3_) δ 7.46 (s, 2H, Benzene-H), 7.36 – 7.29 (m, 2H, Benzothiazole-H, Benzylamine-H), 7.24 (s, 1H, Benzylamine-H), 7.03 (d, *J* = 21.8 Hz, 3H, Benzylamine-H, Benzothiazole-H), 6.94 (s, 1H, Benzothiazole-H), 6.86 (d, *J* = 3.5 Hz, 2H, Benzene-H), 5.33 (d, *J* = 21.9 Hz, 1H, PCH), 4.15 (dd, *J* = 22.7, 11.9 Hz, 3H, CHOH, OCH_2_CH_3_), 3.96 (dd, *J* = 16.3, 14.2 Hz, 3H, OCH_2_CH_3,_ OCH_2_CH), 3.74 (d, *J* = 12.0 Hz, 2H, Benzylamine-CH_2_), 3.62 (d, *J* = 12.6 Hz, 1H, OCH_2_CH), 2.61 (dd, *J* = 41.5, 16.4 Hz, 2H, NCH_2_), 2.52 (s, 3H, Benzothiazole-CH_3_), 2.29 (s, 3H, NCH_3_), 1.27 (t, *J* = 18.5 Hz, 3H, OCH_2_CH_3_), 1.17 – 1.06 (m, 3H, OCH_2_CH_3_); ^13^C NMR (126 MHz, CDCl_3_) δ 165.28, 161.66 (d, ^1^*J_C-F_* = 246.5 Hz), 158.88, 151.17, 131.79, 130.96, 129.76, 129.42, 129.27, 127.58, 126.73, 124.74 (d, ^3^*J_C-F_* = 14.3 Hz), 124.20 (d, ^4^*J_C-F_* = 3.2 Hz), 121.84, 118.37, 115.67 (d, ^2^*J_C-F_* = 22.1 Hz), 114.79, 70.39, 66.29, 63.76 (dd, ^2^*J_C-P_* = 23.7, 7.0 Hz), 59.69, 55.65 (d, ^1^*J_C-P_* = 155.5 Hz), 55.32, 42.23, 18.55, 16.58 (dd, ^3^*J_C-P_* = 22.7, 5.7 Hz); ^31^P NMR (202 MHz, CDCl_3_) δ 21.75; ^19^F NMR (376 MHz, CDCl_3_) δ -117.68; HRMS (ESI) [M+H]^+^ calcd for C_30_H_38_O_5_N_3_PSF: 602.2248, found: 602.2236.

**BtP9,** *diethyl ((4-(3-((3-fluorobenzyl)(methyl)amino)-2-hydroxypropoxy)phenyl)((4-methylbenzo[d]thiazol-2-yl)amino)methyl)phosphonate*. A yellow oil, yield 20.1%; ^1^H NMR NMR (500 MHz, CDCl_3_) δ 7.48 (s, 2H, Benzene-H), 7.34 (d, *J* = 4.1 Hz, 1H, Benzothiazole-H), 7.25 (s, 1H, Benzylamine-H), 7.05 (s, 2H, Benzothiazole-H, Benzylamine-H), 7.01 (d, *J* = 9.2 Hz, 1H, Benzylamine-H), 6.94 (s, 2H, Benzothiazole-H, Benzylamine-H), 6.86 (d, *J* = 5.5 Hz, 2H, Benzene-H), 5.34 (d, *J* = 21.9 Hz, 1H, PCH), 4.23 – 4.07 (m, 3H, CHOH, OCH_2_CH_3_), 4.03 – 3.73 (m, 4H, OCH_2_CH_3,_ OCH_2_CH), 3.59 (dd, *J* = 62.5, 13.1 Hz, 2H, Benzylamine-CH_2_), 2.62 (dd, *J* = 31.5, 21.2 Hz, 2H, NCH_2_), 2.52 (s, 3H, Benzothiazole-CH_3_), 2.27 (s, 3H, NCH_3_), 1.27 (d, *J* = 6.0 Hz, 3H, OCH_2_CH_3_), 1.13 (d, *J* = 5.7 Hz, 3H, OCH_2_CH_3_); ^13^C NMR (126 MHz, CDCl_3_) δ 165.29, 163.12 (d, ^1^*J_C-F_* = 245.9 Hz), 158.86, 151.18, 140.94, 130.97, 130.11 (d, ^3^*J_C-F_ J* = 8.2 Hz), 129.83, 129.31, 127.67, 126.76, 124.80, 121.89, 118.40, 116.00 (d, ^6^*J_C-F_* = 21.2 Hz), 114.80, 114.51 (d, ^2^*J_C-F_* = 21.1 Hz), 70.40, 66.39, 63.78 (dd, ^2^*J_C-P_* = 23.8, 7.0 Hz), 62.24, 59.85, 55.71 (d, ^1^*J_C-P_* = 155.5 Hz), 42.41, 18.57 16.61 (dd, ^3^*J_C-P_* = 22.9, 5.7 Hz); ^31^P NMR (202 MHz, CDCl_3_) δ 21.72; ^19^F NMR (471 MHz, CDCl_3_) δ -113.17; HRMS (ESI) [M+H]^+^ calcd for C_30_H_38_O_5_N_3_PSF: 602.2248, found: 602.2233.

**BtP10,** *diethyl ((4-(3-((4-fluorobenzyl)(methyl)amino)-2-hydroxypropoxy)phenyl)((4-methylbenzo[d]thiazol-2-yl)amino)methyl)phosphonate*. A yellow oil, yield 71.2%; ^1^H NMR (400 MHz, CDCl_3_) δ 7.43 (d, *J* = 6.8 Hz, 2H, Benzene-H), 7.27 (d, *J* = 7.7 Hz, 1H, Benzothiazole-H), 7.16 (dd, *J* = 8.5, 5.5 Hz, 2H, Benzylamine-H), 6.98 (d, *J* = 7.4 Hz, 1H, Benzothiazole-H), 6.94 – 6.84 (m, 3H, Benzylamine-H, Benzothiazole-H), 6.79 (d, *J* = 8.6 Hz, 2H, Benzene-H), 5.35 (d, *J* = 22.0 Hz, 1H, PCH), 4.20 – 3.88 (m, 4H, 2OCH_2_CH_3_), 3.84 (d, *J* = 4.8 Hz, 2H, OCH_2_CH), 3.75 – 3.63 (m, 1H, CHOH), 3.46 (dd, *J* = 55.7, 13.1 Hz, 2H, Benzylamine-CH_2_), 2.53 (dd, *J* = 12.2, 9.7 Hz, 1H, NCH_2_), 2.45 (s, 3H, Benzothiazole-CH_3_), 2.41 (dd, *J* = 12.4, 4.1 Hz, 1H, NCH_2_), 2.16 (s, 1H, NCH_3_), 1.20 (t, *J* = 7.1 Hz, 3H, OCH_2_CH_3_), 1.06 (t, *J* = 7.1 Hz, 3H, OCH_2_CH_3_); ^13^C NMR (101 MHz, CDCl_3_) δ 165.09 (s), 162.28 (d, ^1^*J_C-F_* = 245.3 Hz), 158.83, 151.23, 134.12, 131.04, 130.75 (d, ^3^*J_C-F_* = 7.9 Hz), 129.87, 129.27, 127.77, 126.69, 121.80, 118.35, 115.42 (d, ^2^*J_C-F_* = 21.3 Hz), 114.75, 70.44, 66.34, 63.74 (dd, ^2^*J_C-P_*= 19.7, 7.0 Hz), 61.96, 59.66, 55.57 (d, ^1^*J_C-P_* = 155.6 Hz), 42.26, 18.54, 16.60 (dd, ^3^*J_C-P_* = 18.4, 5.7 Hz); ^31^P NMR (162 MHz, CDCl_3_) δ 21.24; ^19^F NMR (376 MHz, CDCl_3_) δ -125.33; HRMS (ESI) [M+H]^+^ calcd for C_30_H_38_O_5_N_3_PSF: 602.2248, found: 602.2235.

**BtP11,** *diethyl ((4-(2-hydroxy-3-(methyl(4-(trifluoromethyl)benzyl)amino)propoxy)phenyl)((4-methylbenzo[d]thiazol-2-yl)amino)methyl)phosphonate*. A yellow oil, yield 49.2%; ^1^H NMR (400 MHz, CDCl_3_) δ 7.57 (d, *J* = 8.0 Hz, 2H, Benzene-H), 7.49 (d, *J* = 6.8 Hz, 2H, Benzylamine-H), 7.41 (d, *J* = 8.0 Hz, 2H, Benzylamine-H), 7.36 (d, *J* = 7.8 Hz, 1H, Benzothiazole-H), 7.07 (d, *J* = 7.3 Hz, 1H, Benzothiazole-H), 6.96 (t, *J* = 7.6 Hz, 1H, Benzothiazole-H), 6.87 (d, *J* = 8.6 Hz, 2H, Benzene-H), 5.35 (d, *J* = 22.1 Hz, 1H, PCH), 4.23 – 4.06 (m, 3H, CHOH, OCH_2_CH_3_), 4.02 – 3.90 (m, 3H, OCH_2_CH_3,_ OCH_2_CH), 3.83 – 3.73 (m, 1H, OCH_2_CH)), 3.63 (dd, *J* = 38.6, 16.7 Hz, 2H, Benzylamine-CH_2_), 2.65 (dd, *J* = 12.3, 9.5 Hz, 1H, NCH_2_), 2.56 (d, *J* = 4.1 Hz, 1H, OH), 2.53 (s, 1H, Benzothiazole-CH_3_), 2.43 (dd, *J* = 15.5, 10.7 Hz, 1H, NCH_2_), 2.27 (s, 3H, NCH_3_), 1.28 (t, *J* = 7.1 Hz, 3H, OCH_2_CH_3_), 1.14 (t, *J* = 7.1 Hz, 3H, OCH_2_CH_3_); ^13^C NMR (101 MHz, CDCl_3_) δ 165.23, 158.88, 151.23, 142.60, 131.01, 129.84, 129.84 (d, ^2^*J_C-F_* = 32.4 Hz), 129.42, 128.46, 127.71, 126.80, 125.61 (dd, ^2^*J_C-F_* = 8.1, 4.3 Hz), 124.40 (d, ^1^*J_C-F_* = 272.1 Hz), 121.95, 118.43, 114.81, 70.37, 66.46, 63.76 (dd, ^2^*J_C-P_* = 18.5, 6.9 Hz), 62.32, 59.95, 55.76 (d, ^1^*J_C-P_* = 155.1 Hz), 42.45, 18.57, 16.62 (dd, ^3^*J_C-P_* = 18.5, 5.8 Hz); ^31^P NMR (162 MHz, CDCl_3_) δ 21.10; ^19^F NMR (376 MHz, CDCl_3_) δ -62.40; HRMS (ESI) [M+H]^+^ calcd for C_31_H_38_O_5_N_3_PSF_3_: 652.2216, found: 652.2198.

**BtP12,** *diethyl ((4-(2-hydroxy-3-(methyl(4-methylbenzyl)amino)propoxy)phenyl)((4-methylbenzo[d]thiazol-2-yl)amino)methyl)phosphonate*. A yellow oil, yield 73.5%; ^1^H NMR (400 MHz, CDCl_3_) δ 7.43 (d, *J* = 6.8 Hz, 2H, Benzene-H), 7.26 (d, *J* = 7.5 Hz, 1H, Benzothiazole-H), 7.05 (dd, *J* = 22.5, 7.9 Hz, 4H, Benzylamine-H), 6.97 (d, *J* = 7.3 Hz, 1H, Benzothiazole-H), 6.86 (t, *J* = 7.6 Hz, 1H, Benzothiazole-H), 6.78 (d, *J* = 8.6 Hz, 2H, Benzene-H), 5.37 (d, *J* = 22.0 Hz, 1H, PCH), 4.19 – 3.87 (m, 4H, 2OCH_2_CH_3_), 3.83 (d, *J* = 4.9 Hz, 2H, OCH_2_CH), 3.75 – 3.61 (m, 1H, CHOH), 3.46 (dd, *J* = 60.7, 13.0 Hz, 2H, Benzylamine-CH_2_), 2.59 – 2.48 (m, 1H, NCH_2_), 2.45 (s, 3H, Benzothiazole-CH_3_), 2.40 (dd, *J* = 12.4, 4.0 Hz, 1H, NCH_2_), 2.23 (s, 1H, NCH_3_), 2.16 (s, 3H, Benzylamine-CH_3_), 1.19 (t, *J* = 7.1 Hz, 3H, OCH_2_CH_3_), 1.06 (t, *J* = 7.1 Hz, 3H, OCH_2_CH_3_); ^13^C NMR (101 MHz, CDCl_3_) δ 164.93, 158.72, 151.07, 136.95, 135.09, 130.89, 129.71, 129.65, 129.10, 129.07, 127.56, 126.50, 121.59, 118.17, 114.59, 70.37, 66.10, 63.58 (dd, ^2^*J_C-P_* = 21.5, 7.0 Hz), 62.24, 59.45, 55.36 (d, ^1^*J_C-P_* = 155.7 Hz), 42.16, 21.16, 18.38, 16.44 (dd, ^3^*J_C-P_* = 18.4, 5.7 Hz); ^31^P NMR (162 MHz, CDCl_3_) δ 21.32; HRMS (ESI) [M+H]^+^ calcd for C_31_H_41_O_5_N_3_PS: 598.2499, found: 598.2483.

**BtP13,** *diethyl ((4-(3-(cyclopropylamino)-2-hydroxypropoxy)phenyl)((4-methylbenzo[d]thiazol-2-yl)amino)methyl)phosphonate*. A yellow oil, yield 68.5%; ^1^H NMR (400 MHz, CDCl_3_) δ 7.42 (d, *J* = 6.8 Hz, 2H, Benzene-H), 7.27 (d, *J* = 7.8 Hz, 1H, Benzothiazole-H), 6.98 (d, *J* = 7.2 Hz, 1H, Benzothiazole-H), 6.87 (t, *J* = 7.6 Hz, 1H, Benzothiazole-H), 6.79 (d, *J* = 8.6 Hz, 2H, Benzene-H), 5.36 (d, *J* = 22.0 Hz, 1H, PCH), 4.20 – 3.89 (m, 4H, 2OCH_2_CH_3_), 3.83 (d, *J* = 5.2 Hz, 2H, OCH_2_CH), 3.76 – 3.62 (m, 1H, CHOH), 2.80 (ddd, *J* = 20.7, 12.3, 6.0 Hz, 2H, NHCH_2_), 2.45 (s, 3H, Benzothiazole-CH_3_), 2.16 – 2.04 (m, 1H, NHCHCH_2_), 1.19 (t, *J* = 7.1 Hz, 3H, OCH_2_CH_3_), 1.07 (t, *J* = 7.1 Hz, 3H, OCH_2_CH_3_), 0.33 (dd, *J* = 28.3, 7.0 Hz, 4H, CH_2_CH_2_); ^13^C NMR (101 MHz, CDCl_3_) δ 164.95, 158.60, 151.01, 130.81, 129.63, 129.05, 127.56, 126.50, 121.60, 118.16, 114.58, 70.52, 68.00, 63.58 (dd, ^2^*J_C-P_* = 14.1, 7.0 Hz), 55.30 (d, ^1^*J_C-P_* = 155.8 Hz), 51.87, 30.50, 18.37, 16.42 (dd, ^3^*J_C-P_* = 18.3, 5.7 Hz), 6.30; ^31^P NMR (162 MHz, CDCl_3_) δ 21.25; HRMS (ESI) [M+H]^+^ calcd for C_25_H_35_O_5_N_3_PS: 520.2030, found: 520.2019.

**BtP14,** *diethyl ((4-(2-hydroxy-3-(propylamino)propoxy)phenyl)((4-methylbenzo[d]thiazol-2-yl)amino)methyl)phosphonate*. A colorless oil, yield 33.7%; ^1^H NMR (400 MHz, CDCl_3_) δ 7.48 (d, *J* = 7.3 Hz, 2H, Benzene-H), 7.33 (d, *J* = 7.7 Hz, 1H, Benzothiazole-H), 7.04 (d, *J* = 7.3 Hz, 1H, Benzothiazole-H), 6.92 (t, *J* = 7.6 Hz, 1H, Benzothiazole-H), 6.80 (d, *J* = 8.6 Hz, 2H, Benzene-H), 5.60 (d, *J* = 23.3 Hz, 1H, PCH), 4.62 – 4.33 (m, 1H, CHOH), 4.31 – 4.12 (m, 2H, OCH_2_CH_3_), 4.05 – 3.89 (m, 3H, OCH_2_CH_3,_ OCH_2_CH), 3.84 – 3.71 (m, 1H, OCH_2_CH), 3.12 (t, *J* = 13.0 Hz, 2H, NHCH_2_CH), 2.97 – 2.82 (m, 1H, NHCH_2_CH_2_), 2.51 (s, 3H, Benzothiazole-CH_3_), 1.75 (dd, *J* = 15.7, 7.6 Hz, 2H, NHCH_2_CH_2_), 1.25 (t, *J* = 7.0 Hz, 3H, OCH_2_CH_3_), 1.14 (t, *J* = 7.0 Hz, 3H, OCH_2_CH_3_), 0.88 (t, *J* = 7.4 Hz, 3H, CH_2_CH_2_CH_3_); ^13^C NMR (101 MHz, CDCl_3_) δ 164.93, 158.07, 151.01, 130.89, 129.69, 128.94, 128.00, 126.42, 121.51, 118.10, 114.51, 69.74, 65.95, 63.62 (dd, ^2^*J_C-P_* = 16.0, 7.0 Hz), 54.79 (d, ^1^*J_C-P_* = 156.5 Hz), 50.97, 50.40, 19.95, 18.35, 16.42 (dd, ^3^*J_C-P_* = 18.4, 5.7 Hz), 11.19; ^31^P NMR (162 MHz, CDCl_3_) δ 21.19; HRMS (ESI) [M+H]^+^ calcd for C_25_H_37_O_5_N_3_PS: 522.2186, found: 522.2176.

**BtP15,** *diethyl ((4-(3-(butylamino)-2-hydroxypropoxy)phenyl)((4-methylbenzo[d]thiazol-2-yl)amino)methyl)phosphonate*. A colorless oil, yield 50.7%; ^1^H NMR (400 MHz, CDCl_3_) δ 7.42 (d, *J* = 7.0 Hz, 2H, Benzene-H), 7.26 (d, *J* = 7.7 Hz, 1H, Benzothiazole-H), 6.97 (d, *J* = 7.3 Hz, 1H, Benzothiazole-H), 6.85 (t, *J* = 7.6 Hz, 1H, Benzothiazole-H), 6.75 (d, *J* = 8.6 Hz, 2H, Benzene-H), 5.45 (d, *J* = 21.7 Hz, 1H, PCH), 4.21 – 4.02 (m, 3H, CHOH, OCH_2_CH_3_), 3.96 – 3.78 (m, 3H, OCH_2_CH_3,_ OCH_2_CH), 3.74 – 3.63 (m, 1H, OCH_2_CH), 2.89 – 2.70 (m, 2H, NCH_2_CH), 2.69 – 2.55 (m, 2H, NCH_2_CH_2_), 2.44 (s, 3H, Benzothiazole-CH_3_), 1.51 – 1.39 (m, 1H, NCH_2_CH_2_), 1.28 – 1.20 (m, 2H, CH_2_CH_2_CH_3_), 1.18 (t, *J* = 7.0 Hz, 3H, OCH_2_CH_3_), 1.06 (t, *J* = 7.1 Hz, 3H, OCH_2_CH_3_), 0.78 (t, *J* = 7.3 Hz, 3H, CH_2_CH_2_CH_3_); ^13^C NMR (101 MHz, CDCl_3_) δ 165.04, 158.44, 151.02, 130.85, 129.67, 128.96, 127.74, 126.45, 121.53, 118.12, 114.53, 70.33, 67.36, 63.58 (dd, ^2^*J_C-P_* = 7.1, 2.2 Hz), 55.06 (d, ^1^*J_C-P_* = 156.1 Hz), 51.69, 49.22, 30.80, 20.27, 18.36, 16.41 (dd, ^3^*J_C-P_* = 18.3, 5.7 Hz), 13.89; ^31^P NMR (162 MHz, CDCl_3_) δ 21.30; HRMS (ESI) [M+H]^+^ calcd for C_26_H_39_O_5_N_3_PS: 536.2343, found: 536.2328.

**BtP16,** *diethyl ((4-(2-hydroxy-3-((thiophen-2-ylmethyl)amino)propoxy)phenyl)((4-methylbenzo[d]thiazol-2-yl)amino)methyl)phosphonate*. A yellow oil, yield 73.4%; ^1^H NMR (400 MHz, CDCl_3_) δ 7.41 (d, *J* = 6.8 Hz, 2H, Benzene-H), 7.27 (d, *J* = 7.5 Hz, 1H, Benzothiazole-H), 7.13 – 7.09 (m, 1H, Thiophene-H), 6.99 (d, *J* = 7.2 Hz, 1H, Benzothiazole-H), 6.88 (d, *J* = 7.6 Hz, 1H, Benzothiazole-H), 6.86 – 6.82 (m, 2H, Thiophene-H), 6.77 (d, *J* = 8.6 Hz, 2H, Benzene-H), 5.33 (d, *J* = 22.1 Hz, 1H, PCH), 4.18 – 4.05 (m, 2H, OCH_2_CH_3_), 4.01 – 3.89 (m, 4H, OCH_2_CH_3,_ Thiophene-CH_2_), 3.85 (d, *J* = 5.2 Hz, 2H, OCH_2_CH), 3.77 – 3.63 (m, 1H, CHOH), 2.82 (dd, *J* = 12.2, 2.9 Hz, 1H, NHCH_2_), 2.71 (dd, *J* = 12.1, 7.8 Hz, 1H, NHCH_2_), 2.45 (s, 3H, Benzothiazole-CH_3_), 1.19 (t, *J* = 7.0 Hz, 3H, OCH_2_CH_3_), 1.06 (t, *J* = 7.1 Hz, 3H, OCH_2_CH_3_); ^13^C NMR (101 MHz, CDCl_3_) δ 165.06, 158.58, 151.03, 143.32, 130.81, 129.61, 129.09, 127.58, 126.76, 126.53, 125.32, 124.70, 121.64, 118.18, 114.61, 70.41, 68.37, 63.56 (dd, ^2^*J_C-P_* = 14.5, 7.0 Hz), 55.39 (d, ^1^*J_C-P_* = 155.7 Hz), 50.96, 48.20, 18.37, 16.42 (dd, ^3^*J_C-P_* = 18.2, 5.7 Hz); ^31^P NMR (162 MHz, CDCl_3_) δ 21.20; HRMS (ESI) [M+H]^+^ calcd for C_27_H_35_O_5_N_3_PS_2_: 576.1750, found: 567.1715.

**BtP17,** *diethyl ((4-(3-(benzylamino)-2-hydroxypropoxy)phenyl)((4-methylbenzo[d]thiazol-2-yl)amino)methyl)phosphonate*. A yellow oil, yield 47.4%; ^1^H NMR (400 MHz, CDCl_3_) δ 7.40 (d, *J* = 8.7 Hz, 2H, Benzene-H), 7.25 (d, *J* = 7.7 Hz, 1H, Benzothiazole-H), 7.22 – 7.08 (m, 5H, Benzylamine-H), 6.97 (d, *J* = 7.3 Hz, 1H, Benzothiazole-H), 6.85 (t, *J* = 7.6 Hz, 1H, Benzothiazole-H), 6.73 (d, *J* = 8.6 Hz, 2H, Benzene-H), 5.39 (d, *J* = 22.0 Hz, 1H, PCH), 4.17 – 3.95 (m, 3H, CHOH, OCH_2_CH_3_), 3.94 – 3.75 (m, 3H, OCH_2_CH_3,_ OCH_2_CH), 3.71 (d, *J* = 6.3 Hz, 2H, Benzylamine-CH_2_), 3.68 – 3.61 (m, 1H, OCH_2_CH), 2.71 (ddd, *J* = 20.4, 12.9, 6.9 Hz, 2H, NHCH_2_CH), 2.44 (s, 3H, Benzothiazole-CH_3_), 1.17 (t, *J* = 7.1 Hz, 3H, OCH_2_CH_3_), 1.04 (t, *J* = 7.1 Hz, 3H, OCH_2_CH_3_); ^13^C NMR (101 MHz, CDCl_3_) δ 165.11, 158.56, 151.06, 139.29, 130.84, 129.67, 129.03, 128.57, 128.33, 127.63, 127.31, 126.51, 121.59, 118.18), 114.58, 70.47, 68.20, 63.60 (dd, ^2^*J_C-P_* = 12.1, 7.0 Hz), 55.24 (d, ^1^*J_C-P_* = 155.9 Hz), 53.67, 51.30, 18.41, 16.44 (dd, ^3^*J_C-P_* = 18.0, 5.7 Hz); ^31^P NMR (162 MHz, CDCl_3_) δ 21.33; HRMS (ESI) [M+H]^+^ calcd for C_29_H_37_O_5_N_3_PS: 570.2186, found: 570.2172.

**BtP18,** *diethyl ((4-(3-((2-fluorobenzyl)amino)-2-hydroxypropoxy)phenyl)((4-methylbenzo[d]thiazol-2-yl)amino)methyl)phosphonate*. A yellow oil, yield 43.5%; ^1^H NMR (400 MHz, CDCl_3_) δ 7.50 (d, *J* = 6.8 Hz, 2H, Benzene-H), 7.35 (d, *J* = 7.6 Hz, 1H, Benzothiazole-H), 7.30 (t, *J* = 7.5 Hz, 1H, Benzylamine-H), 7.21 (dd, *J* = 15.1, 7.4 Hz, 1H, Benzylamine-H), 7.12 – 6.99 (m, 3H, Benzothiazole-H, Benzylamine-H), 6.95 (t, *J* = 7.6 Hz, 1H, Benzothiazole-H), 6.84 (d, *J* = 8.6 Hz, 2H, Benzene-H), 5.45 (d, *J* = 22.0 Hz, 1H, PCH), 4.28 – 3.95 (m, 4H, 2OCH_2_CH_3_), 3.92 (d, *J* = 5.2 Hz, 2H, OCH_2_CH), 3.87 (s, 2H, Benzylamine-CH_2_), 3.83 – 3.70 (m, 1H, CHOH), 2.80 (dd, *J* = 32.7, 8.8 Hz, 2H, NCH_2_), 2.54 (s, 3H, Benzothiazole-CH_3_), 1.27 (t, *J* = 7.1 Hz, 3H, OCH_2_CH_3_), 1.14 (t, *J* = 7.1 Hz, 3H, OCH_2_CH_3_); ^13^C NMR (101 MHz, CDCl_3_) δ 165.25, 161.38 (d, ^1^*J_C-F_* = 245.6 Hz), 158.74, 151.21, 130.99, 130.61 (d, ^2^*J_C-F_* = 4.7 Hz), 129.86, 129.21, 129.11 (d, ^3^*J_C-F_* = 8.2 Hz), 127.77, 126.81 (d, ^5^*J_C-F_* = 15.0 Hz), 126.67, 124.33, 121.76, 118.33, 115.54 (d, ^6^*J_C-F_* = 21.8 Hz), 114.74, 70.67, 68.47, 63.73 (dd, ^2^*J_C-P_* = 14.2, 7.0 Hz), 55.49 (d, ^1^*J_C-P_* = 155.8 Hz), 51.31, 47.33, 18.54, 16.58 (dd, ^3^*J_C-P_* = 18.2, 5.7 Hz); ^31^P NMR (162 MHz, CDCl_3_) δ 21.28; ^19^F NMR (376 MHz, CDCl_3_) δ -119.16; HRMS (ESI) [M+H]^+^ calcd for C_29_H_36_O_5_N_3_PSF: 588.2092, found: 588.2081.

**BtP19,** *diethyl ((4-(3-((3-fluorobenzyl)amino)-2-hydroxypropoxy)phenyl)((4-methylbenzo[d]thiazol-2-yl)amino)methyl)phosphonate*. A colorless oil, yield 19.4%; ^1^H NMR (500 MHz, CDCl_3_) δ 7.45 (s, 2H, Benzene-H), 7.34 (s, 1H, Benzothiazole-H), 7.25 (s, 2H, Benzylamine-H), 7.09 (dd, *J* = 23.3, 9.1 Hz, 3H, Benzothiazole-H, Benzylamine-H), 6.91 (d, *J* = 25.9 Hz, 2H, Benzene-H), 6.83 (s, 1H, Benzothiazole-H), 5.34 (d, *J* = 22.4 Hz, 1H, PCH), 4.37 (d, *J* = 3.7 Hz, CHOH), 4.14 (dd, *J* = 19.8, 5.8 Hz, 3H, OCH_2_CH_3_, OCH_2_CH), 3.93 (dd, *J* = 26.5, 10.8 Hz, 4H, OCH_2_CH_3,_ Benzylamine-CH_2_), 3.76 (d, *J* = 11.1 Hz, 1H, OCH_2_CH), 2.86 (dd, *J* = 27.0, 8.6 Hz, 3H, NCH_2_, OH), 2.51 (s, 3H, Benzothiazole-CH_3_), 1.25 (s, 3H, OCH_2_CH_3_), 1.13 (d, *J* = 5.3 Hz, 3H, OCH_2_CH_3_); ^13^C NMR (126 MHz, CDCl_3_) δ 165.17 (s), 163.19 (d, ^1^*J_C-F_* = 246.7 Hz), 158.70, 151.21, 131.00, 130.47 (d, ^3^*J_C-F_* = 8.2 Hz), 129.83, 129.42, 127.85, 126.86, 124.51, 122.01, 118.46, 115.79 (d, ^2^*J_C-F_* = 21.4 Hz), 115.08, 114.91, 114.43, 70.41, 67.89, 63.83 (t, ^2^*J_C-P_* = 6.5 Hz), 55.76 (d, ^1^*J_C-P_* = 155.3 Hz), 52.85, 50.98, 18.59, 16.64 (dd, ^3^*J_C-P_* = 22.7, 5.6 Hz); ^31^P NMR (162 MHz, CDCl_3_) δ 21.04; ^19^F NMR (376 MHz, CDCl_3_) δ -112.84; HRMS (ESI) [M+H]^+^ calcd for C_29_H_36_O_5_N_3_PSF: 588.2092, found: 588.2077.

**BtP20,** *diethyl ((4-(3-((4-fluorobenzyl)amino)-2-hydroxypropoxy)phenyl)((4-methylbenzo[d]thiazol-2-yl)amino)methyl)phosphonate*. A colorless oil, yield 31.7%; ^1^H NMR (400 MHz, CDCl_3_) δ 7.39 (d, *J* = 8.5 Hz, 2H, Benzene-H), 7.26 (d, *J* = 7.8 Hz, 1H, Benzothiazole-H), 7.16 (dd, *J* = 9.8, 4.1 Hz, 2H, Benzylamine-H), 6.97 (d, *J* = 7.3 Hz, 1H, Benzothiazole-H), 6.92 – 6.80 (m, 3H, Benzylamine-H, Benzothiazole-H), 6.74 (d, *J* = 8.6 Hz, 2H, Benzene-H), 5.37 (d, *J* = 22.0 Hz, 1H, PCH), 4.17 – 3.86 (m, 4H, 2OCH_2_CH_3_), 3.81 (d, *J* = 4.8 Hz, 2H, OCH_2_CH), 3.73 – 3.61 (m, 3H, Benzylamine-CH_2_, CHOH), 2.70 (dd, *J* = 38.9, 16.1 Hz, 2H, NCH_2_), 2.44 (s, 3H, Benzothiazole-CH_3_), 1.17 (t, *J* = 7.1 Hz, 3H, OCH_2_CH_3_), 1.05 (t, *J* = 7.1 Hz, 3H, OCH_2_CH_3_); ^13^C NMR (101 MHz, CDCl_3_) δ 165.10, 162.14 (d, ^1^*J_C-F_* = 244.9 Hz), 158.69, 151.17, 135.50 (d, ^4^*J_C-F_* = 3.1 Hz), 130.94, 130.01, 129.85 (d, ^3^*J_C-F_* = 6.0 Hz), 129.17, 127.79, 126.67, 121.76, 118.32, 115.43 (d, ^2^*J_C-F_* = 21.2 Hz), 114.70, 70.62, 68.47, 63.73 (dd, ^2^*J_C-P_* = 12.3, 7.0 Hz), 55.39 (d, ^1^*J_C-P_* = 156.0 Hz), 53.15, 51.44, 18.53, 16.56 (dd, ^3^*J_C-P_* = 18.0, 5.7 Hz); ^31^P NMR (162 MHz, CDCl_3_) δ 21.16; ^19^F NMR (376 MHz, CDCl_3_) δ -115.54; HRMS (ESI) [M+H]^+^ calcd for C_29_H_36_O_5_N_3_PSF: 588.2092, found: 588.2077.

**BtP21,** *diethyl ((4-(2-hydroxy-3-((4-(trifluoromethyl)benzyl)amino)propoxy)phenyl)((4-methylbenzo[d]thiazol-2-yl)amino)methyl)phosphonate*. A yellow oil, yield 80.8%; ^1^H NMR (400 MHz, CDCl_3_) δ 7.58 (d, *J* = 8.1 Hz, 2H, Benzene-H), 7.51 (d, *J* = 6.9 Hz, 2H, Benzylamine-H), 7.45 (d, *J* = 8.0 Hz, 2H, Benzylamine-H), 7.38 (d, *J* = 7.6 Hz, 1H, Benzothiazole-H), 7.09 (d, *J* = 7.3 Hz, 1H, Benzothiazole-H), 6.98 (t, *J* = 7.6 Hz, 1H, Benzothiazole-H), 6.87 (d, *J* = 8.6 Hz, 2H, Benzene-H), 5.44 (d, *J* = 22.1 Hz, 1H, PCH), 4.29 – 3.99 (m, 4H, 2OCH_2_CH_3_), 3.96 (d, *J* = 5.0 Hz, 2H, OCH_2_CH), 3.90 (s, 2H, Benzylamine-CH_2_), 3.86 – 3.74 (m, 1H, CHOH), 2.91 – 2.77 (m, 2H, NCH_2_), 2.56 (s, 3H, Benzothiazole-CH_3_), 1.30 (t, *J* = 7.1 Hz, 3H, OCH_2_CH_3_), 1.17 (t, *J* = 7.1 Hz, 3H, OCH_2_CH_3_); ^13^C NMR (101 MHz, CDCl_3_) δ 165.26, 158.71, 151.21, 144.08, 130.97, 129.90, 129.58 (q, ^2^*J_C-F_* = 32.3 Hz), 129.30, 128.57, 127.87, 126.76, 125.62 (q, ^3^*J_C-F_* = 3.7 Hz), 124.42 (d, ^1^*J_C-F_* = 272.0 Hz), 121.8, 118.38, 114.77 (s), 70.61, 68.67, 63.77 (dd, ^2^*J_C-P_* = 12.6, 7.1 Hz), 55.57 (d, ^1^*J_C-P_* = 155.7 Hz), 53.45, 51.52, 18.56, 16.59 (dd, ^3^*J_C-P_* = 18.3, 5.7 Hz); ^31^P NMR (162 MHz, CDCl_3_) δ 21.09; ^19^F NMR (376 MHz, CDCl_3_) δ -62.35; HRMS (ESI) [M+H]^+^ calcd for C_30_H_36_O_5_N_3_PSF_3_: 638.2060, found: 638.2045.

**BtP22,** *diethyl ((4-(2-hydroxy-3-((4-methoxybenzyl)amino)propoxy)phenyl)((4-methylbenzo[d]thiazol-2-yl)amino)methyl)phosphonate*. A yellow oil, yield 24.6%; ^1^H NMR (400 MHz, CDCl_3_) δ 7.41 (d, *J* = 8.7 Hz, 2H, Benzene-H), 7.26 (d, *J* = 7.7 Hz, 1H, Benzothiazole-H), 7.15 (d, *J* = 6.9 Hz, 2H, Benzylamine-H), 7.08 (d, *J* = 7.6 Hz, 3H, Benzylamine-H), 6.98 (d, *J* = 7.3 Hz, 1H, Benzothiazole-H), 6.86 (t, *J* = 7.6 Hz, 1H, Benzothiazole-H), 6.74 (d, *J* = 8.6 Hz, 2H, Benzene-H), 5.40 (d, *J* = 21.9 Hz, 1H, PCH), 4.19 – 4.02 (m, 4H, 2OCH_2_CH_3_) , 3.82 (d, *J* = 4.3 Hz, 2H, OCH_2_CH), 3.69 (ddd, *J* = 10.1, 8.4, 7.2 Hz, 1H, CHOH), 2.99 – 2.67 (m, 6H, CH_2_NHCH_2_CH_2_), 2.45 (s, 3H, Benzothiazole-CH_3_), 1.18 (t, *J* = 7.1 Hz, 3H, OCH_2_CH_3_), 1.06 (t, *J* = 7.1 Hz, 3H, OCH_2_CH_3_); ^13^C NMR (101 MHz, CDCl_3_) δ 165.06, 158.44, 151.02, 139.08, 130.83, 129.70, 129.03, 128.74, 128.61, 127.66, 126.51, 126.44, 121.61, 118.18, 114.57, 70.31, 67.64, 64.46, 55.20 (d, ^1^*J_C-P_* = 155.9 Hz), 51.61, 50.81, 35.50, 18.39, 16.43 (dd, ^3^*J_C-P_* = 18.1, 5.7 Hz); ^31^P NMR (162 MHz, CDCl_3_) δ 21.25; HRMS (ESI) [M+H]^+^ calcd for C_30_H_39_O_5_N_3_PS: 584.2343, found: 584.2332.

**BtP23,** *diethyl ((4-(2-hydroxy-3-((3-phenylpropyl)amino)propoxy)phenyl)((4-methylbenzo[d]thiazol-2-yl)amino)methyl)phosphonate*. A yellow oil, yield 32.0%; ^1^H NMR (400 MHz, CDCl_3_) δ 7.40 (d, *J* = 7.4 Hz, 2H, Benzene-H), 7.24 (d, *J* = 7.7 Hz, 1H, Benzothiazole-H), 7.10 – 6.98 (m, 5H, Benzylamine-H), 6.96 (d, *J* = 7.3 Hz, 1H, Benzothiazole-H), 6.84 (t, *J* = 7.6 Hz, 1H, Benzothiazole-H), 6.68 (d, *J* = 8.5 Hz, 2H, Benzene-H), 5.54 (d, *J* = 21.7 Hz, 1H, PCH), 4.34 (s, 1H, CHOH), 4.22 – 4.01 (m, 2H, OCH_2_CH_3_), 3.97 – 3.60 (m, 4H, OCH_2_CH_3,_ OCH_2_CH), 3.04 – 2.89 (m, 2H, CH_2_NHCH_2_), 2.85 – 2.72 (m, 2H, CH_2_NHCH_2_), 2.50 (t, *J* = 7.5 Hz, 2H, Benzylamine-CH_2_), 2.44 (s, 3H, Benzothiazole-CH_3_), 1.95 (t, *J* = 11.0 Hz, 2H, NHCH_2_CH_2_), 1.16 (t, *J* = 7.1 Hz, 3H, OCH_2_CH_3_), 1.05 (t, *J* = 7.0 Hz, 3H, OCH_2_CH_3_); ^13^C NMR (101 MHz, CDCl_3_) δ 164.84, 158.11, 151.04, 140.44, 130.93, 129.69, 128.94, 128.53, 128.34, 127.99, 126.44, 126.21, 121.52, 118.13, 114.53, 69.80, 66.20, 63.64 (dd, ^2^*J_C-P_* = 17.8, 7.1 Hz), 54.79 (d, ^1^*J_C-P_* = 156.3 Hz), 51.17, 48.46, 32.87, 28.34, 18.39, 16.44 (dd, ^3^*J_C-P_ J* = 18.2, 5.7 Hz); ^31^P NMR (162 MHz, CDCl_3_) δ 21.25; HRMS (ESI) [M+H]^+^ calcd for C_31_H_41_O_5_N_3_PS: 598.2499, found: 598.2488.

**BtP24,** *diethyl ((4-(3-((4-fluorophenethyl)amino)-2-hydroxypropoxy)phenyl)((4-methylbenzo[d]thiazol-2-yl)amino)methyl)phosphonate*. A brown oil, yield 82.3%; ^1^H NMR (400 MHz, CDCl_3_) δ 7.41 (d, *J* = 6.9 Hz, 2H, Benzene-H), 7.26 (d, *J* = 7.5 Hz, 1H, Benzothiazole-H), 7.05 – 6.95 (m, 3H, Benzothiazole-H, Benzylamine-H), 6.85 (dd, *J* = 16.7, 8.2 Hz, 3H, Benzothiazole-H, Benzylamine-H), 6.74 (d, *J* = 8.6 Hz, 2H, Benzene-H), 5.40 (d, *J* = 21.9 Hz, 1H, PCH), 4.18 – 4.00 (m, 3H, CHOH, OCH_2_CH_3_), 3.95 – 3.79 (m, 3H, OCH_2_CH_3,_ OCH_2_CH), 3.73 – 3.64 (m, 1H, OCH_2_CH), 2.97 – 2.60 (m, 6H, CH_2_NHCH_2_CH_2_), 2.44 (s, 3H, Benzothiazole-CH_3_), 1.18 (t, *J* = 7.1 Hz, 3H, OCH_2_CH_3_), 1.05 (t, *J* = 7.1 Hz, 3H, OCH_2_CH_3_); ^13^C NMR (101 MHz, CDCl_3_) δ 165.20, 161.69 (d, ^1^*J_C-F_* = 244.2 Hz), 158.63, 151.18, 135.00, 130.98, 130.28 (d, ^3^*J_C-F_* = 7.8 Hz), 129.89, 129.19, 127.89, 126.68, 121.77, 118.33, 115.50 (d, ^2^*J_C-F_* = 21.2 Hz), 114.72, 70.50, 67.90, 63.75 (dd, ^2^*J_C-P_* = 7.0, 4.3 Hz), 55.34 (d, ^1^*J_C-P_* = 155.9 Hz), 51.80, 51.04, 34.95, 18.53, 16.57 (dd, ^3^*J_C-P_* = 18.2, 5.7 Hz); ^31^P NMR (162 MHz, CDCl_3_) δ 21.10; ^19^F NMR (376 MHz, CDCl_3_) δ -116.71.; HRMS (ESI) [M+H]^+^ calcd for C_30_H_38_O_5_N_3_PSF: 602.2248, found: 602.2238.

**BtP25,** *diethyl ((4-(2-hydroxy-3-(((R)-1-phenylethyl)amino)propoxy)phenyl)((4-methylbenzo[d]thiazol-2-yl)amino)methyl)phosphonate*. A brown oil, yield 72.8%;^1^H NMR (500 MHz, CDCl_3_) δ 7.45 (d, *J* = 6.9 Hz, 2H, Benzene-H), 7.34 (d, *J* = 7.8 Hz, 1H, Benzothiazole-H), 7.32 – 7.26 (m, 4H, Benzylamine-H), 7.24 – 7.19 (m, 1H, Benzylamine-H), 7.06 (d, *J* = 7.2 Hz, 1H, Benzothiazole-H), 6.95 (t, *J* = 7.6 Hz, 1H, Benzothiazole-H), 6.83 (d, *J* = 8.7 Hz, 2H, Benzene-H), 5.32 (d, *J* = 22.1 Hz, 1H, CPH), 4.23 – 4.08 (m, 2H, OCH_2_CH_3_), 4.01 – 3.82 (m, OCH_2_CH_3,_OCH_2_CH), 3.81 – 3.70 (m, CHOH, NHCHCH_3_), 2.65 (d, *J* = 5.8 Hz, NHCH_2_), 2.52(s, 3H, Benzothiazole-CH_3_), 1.37 (d, *J* = 6.6 Hz, 3H, NHCHCH_3_), 1.26 (t, *J* = 7.1 Hz, 3H, OCH_2_CH_3_), 1.13 (t, *J* = 7.1 Hz, 3H, OCH_2_CH_3_); ^13^C NMR (126 MHz, CDCl_3_) δ 165.06, 158.65, 151.06, 145.07, 130.84, 129.64, 129.23, 128.66, 127.48, 127.24, 126.66, 126.62, 121.79, 118.28, 114.68, 70.50, 68.84, 63.61 (dd, ^2^*J_C-P_* = 20.2, 7.0 Hz), 58.78, 55.60 (d, ^1^*J_C-P_* = 155.0 Hz), 49.92, 24.30, 18.43, 16.47 (dd, ^3^*J_C-P_* = 22.8, 5.7 Hz); ^31^P NMR (202 MHz, CDCl_3_) δ 21.61; HRMS (ESI) [M+H]^+^ calcd for C_30_H_39_O_5_N_3_PS: 584.2343, found: 584.2328.

**BtP26,** *diethyl ((4-(2-hydroxy-3-(((S)-1-phenylethyl)amino)propoxy)phenyl)((4-methylbenzo[d]thiazol-2-yl)amino)methyl)phosphonate*. A brown oil, yield 85.2%;^1^H NMR (400 MHz, CDCl_3_) δ 7.54 (d, *J* = 6.9 Hz, 2H, Benzene-H), 7.41 – 7.29 (m, 5H, Benzothiazole-H, Benzylamine-H), 7.28 – 7.22 (m, 1H, Benzylamine-H), 7.10 (dd, *J* = 7.4, 0.7 Hz, 1H, Benzothiazole-H), 6.98 (t, *J* = 7.6 Hz, 1H, Benzothiazole-H), 6.85 (d, *J* = 8.2 Hz, 2H, Benzene-H), 5.53 (d, *J* = 21.9 Hz, 1H, PCH), 4.34 – 3.98 (m, 4H, 2OCH_2_CH_3_), 3.97 – 3.71 (m, 4H, OCH_2_CH, CHOH, NHCH), 2.68 (d, *J* = 6.0 Hz, 2H, NHCH_2_), 2.58 (s, 3H, Benzothiazole-CH_3_), 1.41 (d, *J* = 6.6 Hz, 3H, NHCH_3_), 1.31 (t, *J* = 7.1 Hz, 3H, OCH_2_CH_3_), 1.18 (t, *J* = 7.1 Hz, 3H, OCH_2_CH_3_); ^13^C NMR (101 MHz, CDCl_3_) δ 165.19, 158.68, 151.16, 145.18, 130.97, 129.79, 128.70, 127.75, 127.26, 126.76, 126.61, 125.86, 121.69, 118.28, 114.66, 70.67, 68.91, 63.70 (dd, ^2^*J_C-P_* = 13.3, 7.0 Hz), 58.83, 55.33 (d, ^1^*J_C-P_* = 156.0 Hz), 50.14, 24.57, 18.52, 16.55 (dd, ^3^*J_C-P_* = 18.1, 5.7 Hz); ^31^P NMR (162 MHz, CDCl_3_) δ 21.35; HRMS (ESI) [M+H]^+^ calcd for C_30_H_39_O_5_N_3_PS: 584.2343, found: 584.2330.

**BtP27,** *diethyl ((4-(3-(((R)-1-(4-fluorophenyl)ethyl)amino)-2-hydroxypropoxy)phenyl)((4-methylbenzo[d]thiazol-2-yl)amino)methyl)phosphonate*. A yellow oil, yield 71.7%;^1^H NMR (400 MHz, CDCl_3_) δ 7.38 (d, *J* = 6.8 Hz, 2H, Benzene-H), 7.30 – 7.20 (m, 3H, Benzothiazole-H, Benzylamine-H), 6.99 (d, *J* = 7.3 Hz, 1H, Benzothiazole-H), 6.96 – 6.85 (m, 4H, Benzothiazole-H, Benzylamine-H), 6.73 (d, *J* = 8.5 Hz, 2H, Benzene-H), 5.30 (d, *J* = 20.9 Hz, 1H, PCH), 4.16 – 3.88 (m, 4H, 2OCH_2_CH_3_), 3.87 – 3.64 (m, 4H, OCH_2_CH, CHOH, NHCH), 2.78 – 2.56 (m, 2H, NHCH_2_), 2.45 (s, 3H, Benzothiazole-CH_3_), 1.36 (d, *J* = 6.6 Hz, 3H, NCH_3_), 1.24 – 1.14 (m, 3H, OCH_2_CH_3_), 1.07 (t, *J* = 7.1 Hz, 3H, OCH_2_CH_3_); ^13^C NMR (101 MHz, CDCl_3_) δ 165.26, 162.37 (d, ^1^*J_C-F_* = 245.2 Hz), 158.66, 151.18, 139.03, 130.98, 129.82, 129.35, 128.68 (t, ^3^*J_C-F_* = 8.3 Hz), 127.78, 126.80, 121.94, 118.43, 115.79 (d, ^2^*J_C-F_* = 21.2 Hz), 114.81, 70.49, 68.38, 63.81 (dd, ^2^*J_C-P_* = 6.8, 5.3 Hz), 58.42, 55.61 (d, ^1^*J_C-P_* = 155.4 Hz), 49.85, 23.71, 18.58, 16.62 (dd, ^3^*J_C-P_* = 18.2, 5.7 Hz); ^31^P NMR (162 MHz, CDCl_3_) δ 21.05; ^19^F NMR (376 MHz, CDCl_3_) δ -114.89; HRMS (ESI) [M+H]^+^ calcd for C_30_H_38_O_5_N_3_PSF: 602.2248, found: 602.2239.

**BtP28,** *diethyl ((4-(3-(((S)-1-(4-fluorophenyl)ethyl)amino)-2-hydroxypropoxy)phenyl)((4-methylbenzo[d]thiazol-2-yl)amino)methyl)phosphonate*. A yellow oil, yield 60.3%; ^1^H NMR (400 MHz, CDCl_3_) δ 7.45 (d, *J* = 7.6 Hz, 2H, Benzene-H), 7.38 – 7.29 (m, 3H, Benzothiazole-H, Benzylamine-H), 7.06 (d, *J* = 7.3 Hz, 1H, Benzothiazole-H), 7.03 – 6.90 (m, 4H, Benzothiazole-H, Benzylamine-H), 6.79 (d, *J* = 8.4 Hz, 2H, Benzene-H), 5.41 (d, *J* = 21.9 Hz, 1H, PCH), 4.28 – 4.00 (m, 4H, 2OCH_2_CH_3_), 3.98 – 3.75 (m, 4H, OCH_2_CH, CHOH, NHCH), 2.91 – 2.58 (m, 2H, NHCH_2_), 2.52 (s, 3H, Benzothiazole-CH_3_), 1.46 (d, *J* = 6.3 Hz, 3H, NCH_3_), 1.29 – 1.23 (m, 2H, OCH_2_CH_3_), 1.13 (t, *J* = 7.1 Hz, 3H, OCH_2_CH_3_); ^13^C NMR (101 MHz, CDCl_3_) δ 165.26, 162.46 (d, ^1^*J_C-F_* = 243.8 Hz), 158.57, 151.16, 130.98, 129.77, 129.30, 128.86 , 127.79, 126.78, 121.91, 118.41, 115.88 (d, ^2^*J_C-F_* = 22.3 Hz), 114.78, 70.40, 68.05, 63.82 (d, ^2^*J_C-P_* = 6.9 Hz), 58.21, 55.52 (d, ^2^*J_C-P_* = 155.8 Hz), 49.60, 29.95, 18.57, 16.61 (dd, ^3^*J_C-P_* = 18.3, 5.7 Hz); ^31^P NMR (162 MHz, CDCl_3_) δ 21.08; ^19^F NMR (376 MHz, CDCl3) δ -114.49. HRMS (ESI) [M+H]^+^ calcd for C_30_H_38_O_5_N_3_PSF: 602.2248, found: 602.2235.

**BtP29,** *diethyl ((4-(2-hydroxy-3-(((R)-1-(4-methoxyphenyl)ethyl)amino)propoxy)phenyl)((4-methylbenzo[d]thiazol-2-yl)amino)methyl)phosphonate*. A yellow oil, yield 24.6%;^1^H NMR (400 MHz, CDCl_3_) δ 7.46 (d, *J* = 6.9 Hz, 2H, Benzene-H), 7.33 (d, *J* = 7.8 Hz, 1H, Benzothiazole-H), 7.20 (dd, *J* = 16.1, 7.5 Hz, 2H, Benzylamine-H), 7.04 (d, *J* = 7.4 Hz, 1H, Benzothiazole-H), 6.93 (t, *J* = 7.6 Hz, 1H, Benzothiazole-H), 6.86 – 6.79 (m, 4H, Benzene-H, Benzylamine-H), 5.41 (d, *J* = 22.0 Hz, 1H, PCH), 4.22 – 3.91 (m, 4H, 2OCH_2_CH_3_), 3.87 – 3.81 (m, 1H, CHOH), 3.78 – 3.66 (m, 6H, OCH_3_, OCH_2_CH, NHCH). 2.61 (d, *J* = 5.9 Hz, 2H, NHCH_2_), 2.51 (s, 3H, Benzothiazole-CH_3_), 1.35 (d, *J* = 6.6 Hz, 3H, NCH_3_), 1.25 (t, *J* = 7.1 Hz, 3H, OCH_2_CH_3_), 1.12 (t, *J* = 7.1 Hz, 3H, OCH_2_CH_3_); ^13^C NMR (101 MHz, CDCl_3_) δ 165.07, 158.74, 151.18, 139.67, 137.18, 130.97, 129.75, 129.23, 127.84, 126.98, 126.69, 121.80, 118.34, 114.75, 114.02, 70.74, 68.86, 63.71 (dd, ^2^*J_C-P_* = 12.7, 7.0 Hz), 58.17, 56.91 (d, ^1^*J_C-P_* = 127.1 Hz), 55.46, 50.85, 25.67, 24.34, 18.52, 16.57 (dd, ^3^*J_C-P_* = 18.1, 5.7 Hz); ^31^P NMR (162 MHz, CDCl_3_) δ 21.23; HRMS (ESI) [M+H]^+^ calcd for C_30_H_38_O_5_N_3_PSF: 614.2448, found: 614.2435.

**BtP30,** *diethyl ((4-(2-hydroxy-3-(((S)-1-(4-methoxyphenyl)ethyl)amino)propoxy)phenyl)((4-methylbenzo[d]thiazol-2-yl)amino)methyl)phosphonate*. A yellow oil, yield 68.8%; ^1^H NMR (400 MHz, CDCl_3_) δ 7.46 (d, *J* = 6.9 Hz, 2H, Benzene-H), 7.33 (d, *J* = 7.8 Hz, 1H, Benzothiazole-H), 7.20 (dd, *J* = 16.1, 7.5 Hz, 2H, Benzylamine-H), 7.04 (d, *J* = 7.4 Hz, 1H, Benzothiazole-H), 6.93 (t, *J* = 7.6 Hz, 1H, Benzothiazole-H), 6.86 – 6.79 (m, 4H, Benzene-H, Benzylamine-H), 5.41 (d, *J* = 22.0 Hz, 1H, PCH), 4.22 – 3.91 (m, 4H, 2OCH_2_CH_3_), 3.87 – 3.81 (m, 1H, CHOH), 3.78 – 3.66 (m, 6H, OCH_3_, OCH_2_CH, NHCH). 2.61 (d, *J* = 5.9 Hz, 2H, NHCH_2_), 2.51 (s, 3H, Benzothiazole-CH_3_), 1.35 (d, *J* = 6.6 Hz, 3H, NCH_3_), 1.25 (t, *J* = 7.1 Hz, 3H, OCH_2_CH_3_), 1.12 (t, *J* = 7.1 Hz, 3H, OCH_2_CH_3_); ^13^C NMR (101 MHz, CDCl_3_) δ 165.01, 158.56, 151.01, 139.09, 136.54, 130.82, 129.57, 129.08, 127.70, 126.90, 126.53, 121.65, 118.18, 114.59, 113.87, 70.48, 68.55, 63.55 (dd, ^2^*J_C-P_* = 11.8, 7.0 Hz), 58.07, 56.78 (d, ^1^*J_C-P_* = 131.3 Hz), 55.26, 50.72, 25.29, 23.97, 18.36, 16.41 (dd, ^3^*J_C-P_* = 18.0, 5.7 Hz); ^31^P NMR (162 MHz, CDCl_3_) δ 21.04; HRMS (ESI) [M+H]^+^ calcd for C_30_H_38_O_5_N_3_PSF: 614.2448, found: 614.2437.

# 5. ^1^H NMR, ^13^C NMR, ^31^P NMR, ^19^F NMR, and HRMS Spectra of BtP1-30.


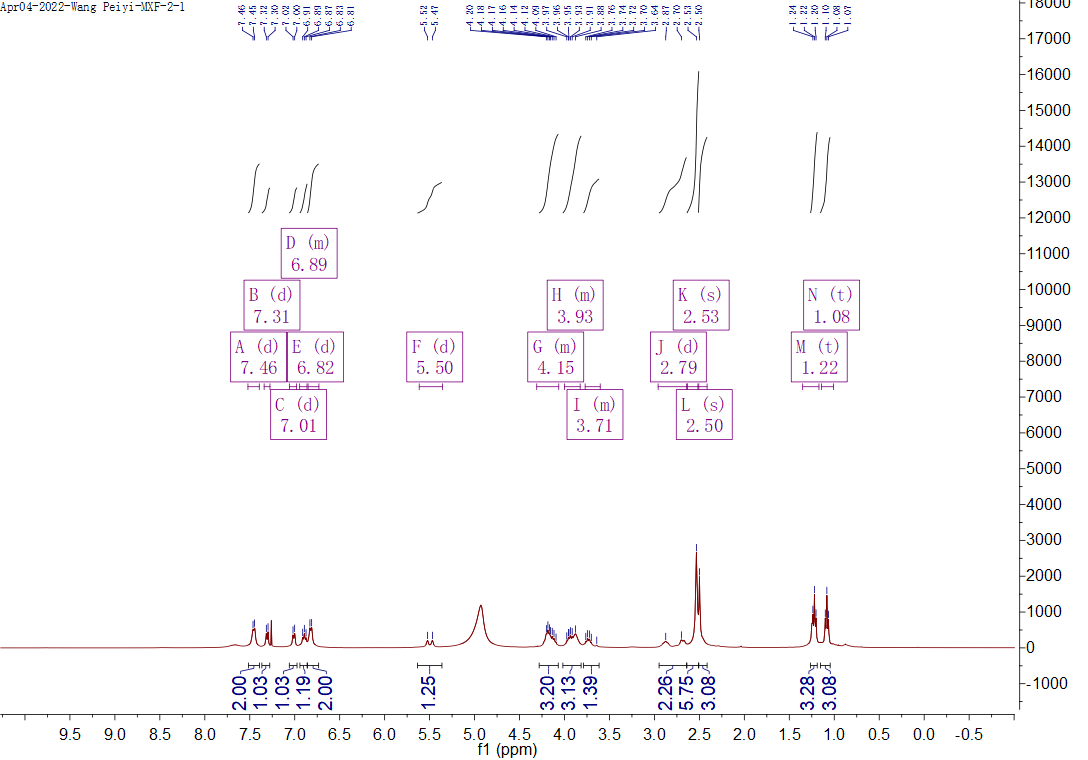
**Figure S26.** ^1^H NMR Spectrum (CDCl_3_, 400 MHz) of **BtP1**

**_
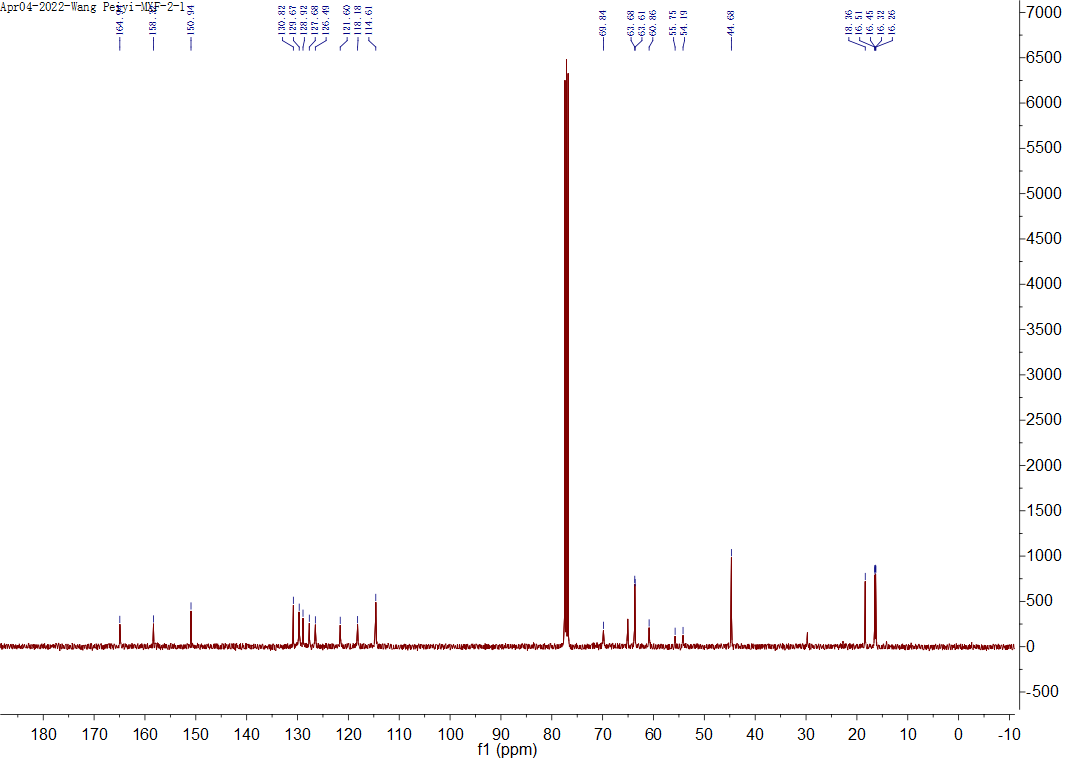
_Figure S27.** ^13^C NMR Spectrum (CDCl_3_, 101 MHz) of **BtP1**

**
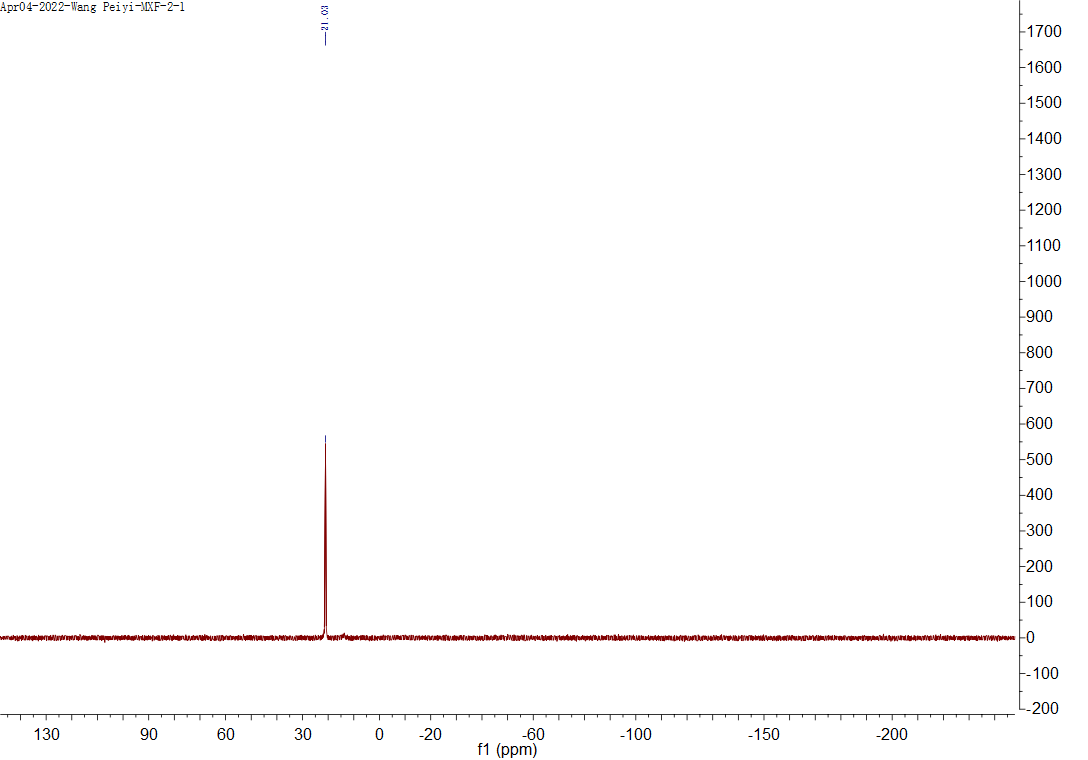
Figure S28.** ^31^P NMR Spectrum (CDCl_3_, 162 MHz) of **BtP1**

**Figure S29.** HRMS Spectrum of Target Compound **BtP1**


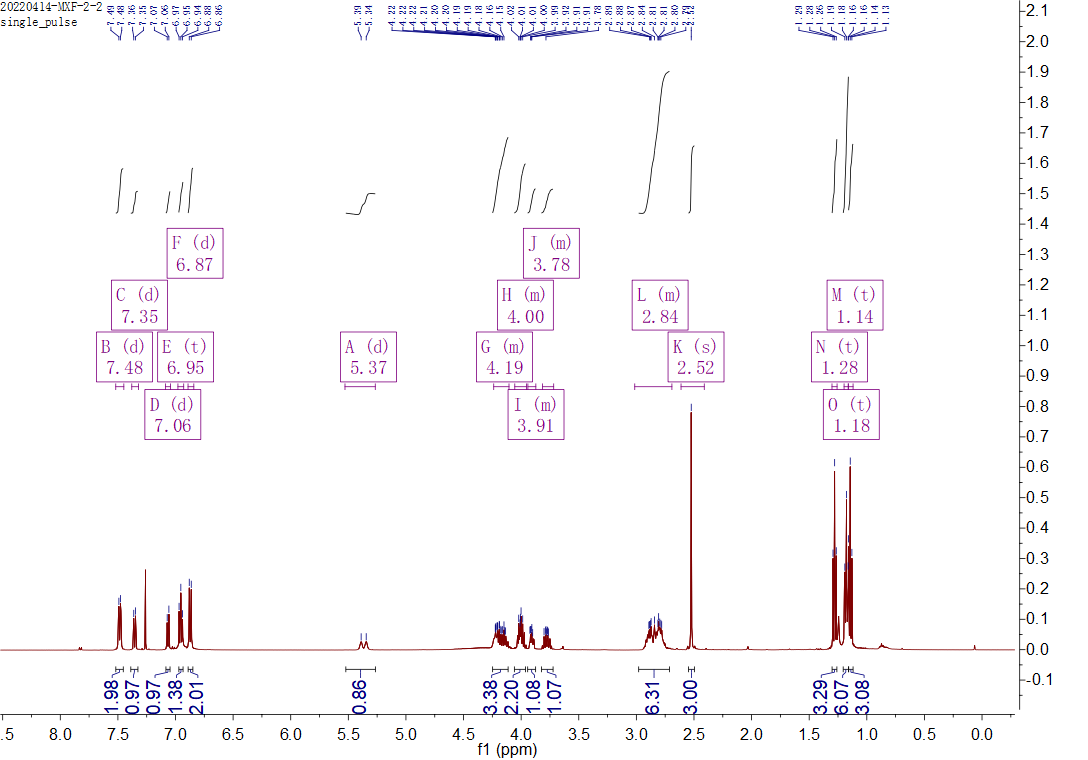
**Figure S30.** ^1^H NMR Spectrum (CDCl_3_, 500 MHz) of **BtP2**

**_
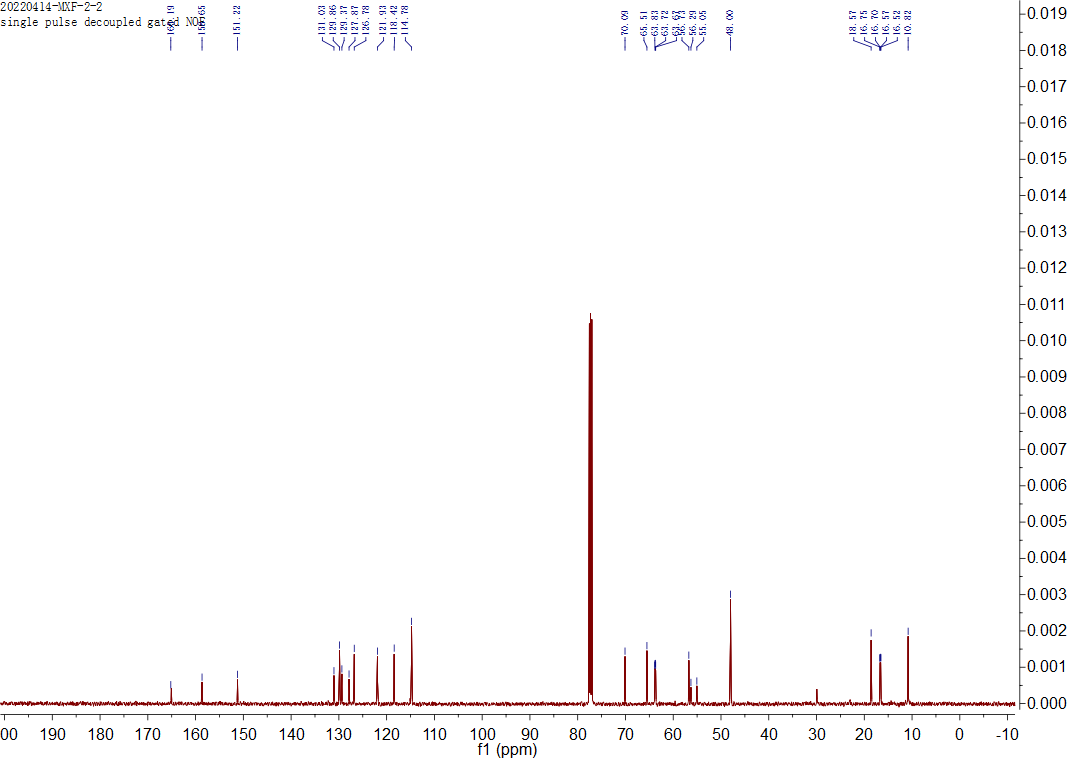
_Figure S31.** ^13^C NMR Spectrum (CDCl_3_, 126 MHz) of **BtP2**


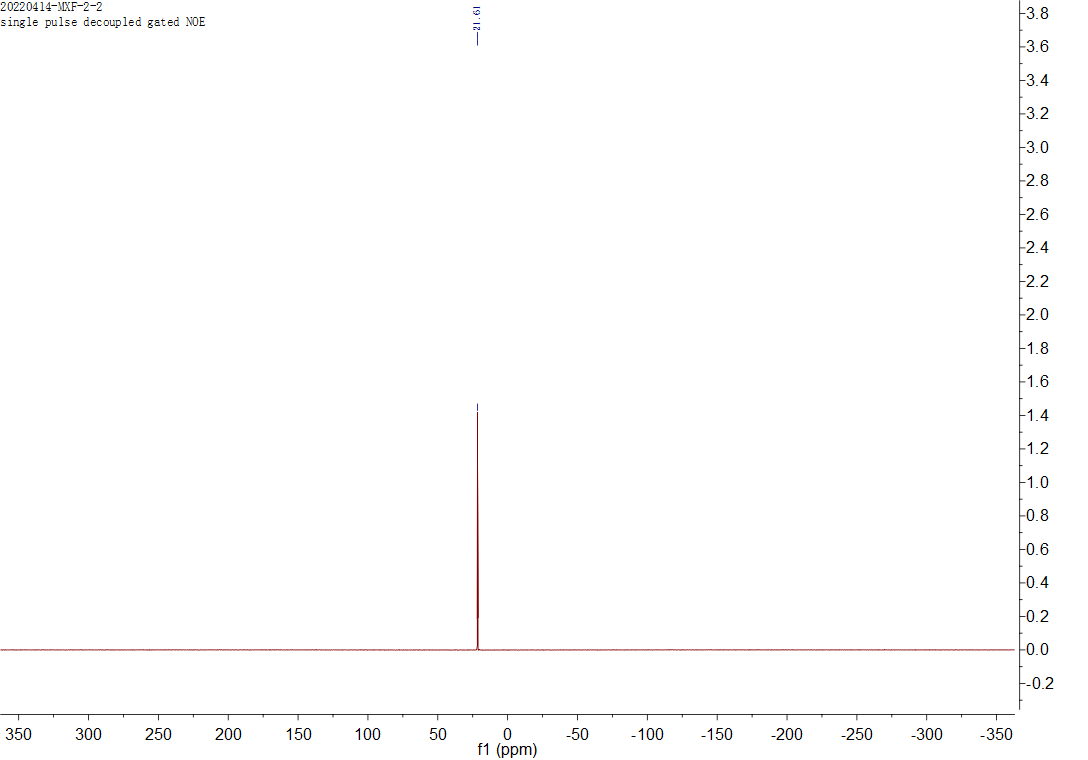


**Figure S32.** ^31^P NMR Spectrum (CDCl_3_, 202 MHz) of **BtP2**

**Figure S33.** HRMS Spectrum of Target Compound **BtP2**


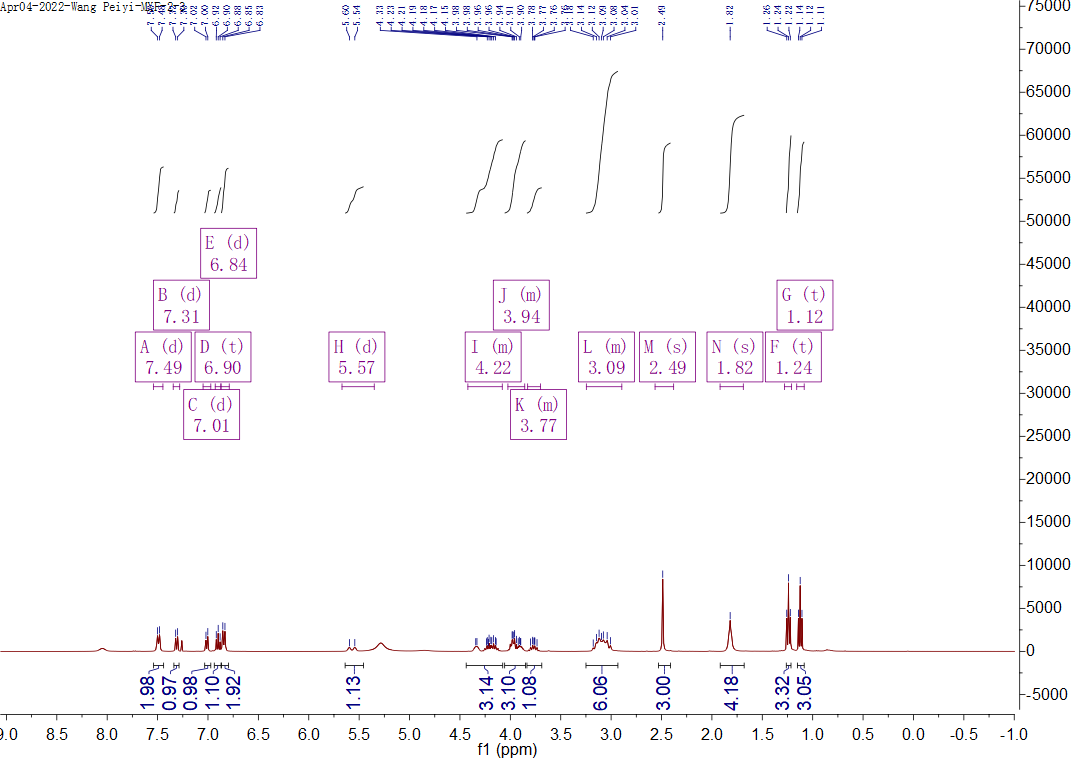
**Figure S34.** ^1^H NMR Spectrum (CDCl_3_, 400 MHz) of **BtP3**

**_
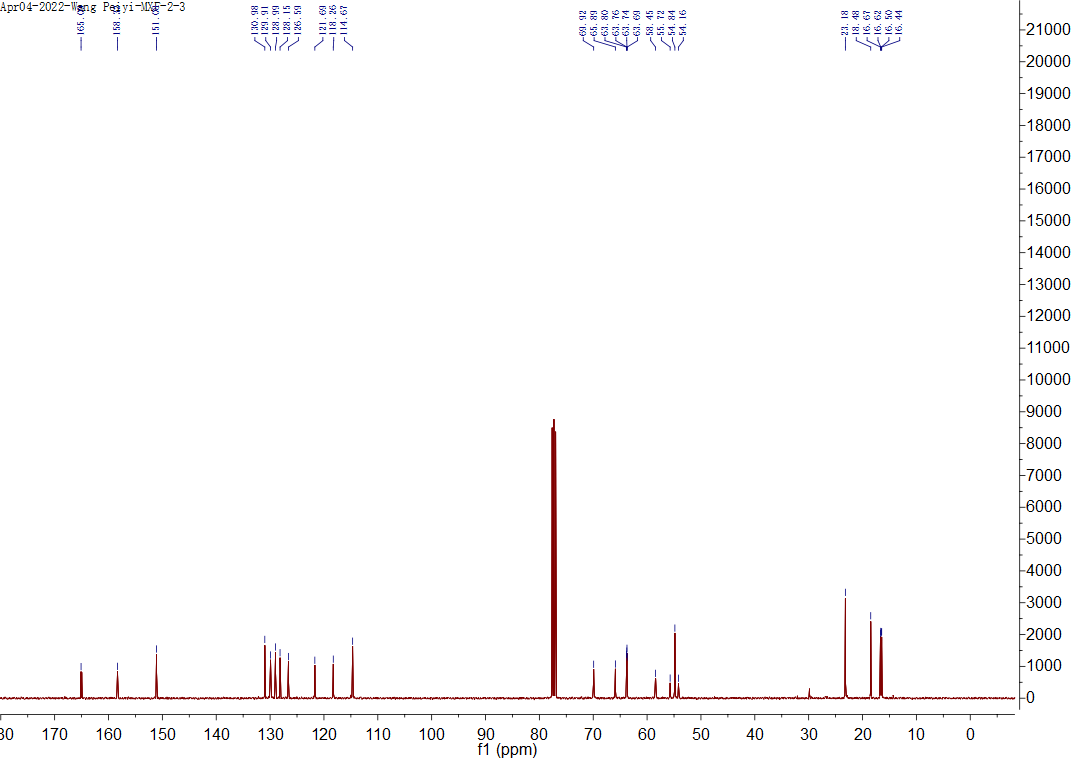
_Figure S35.** ^13^C NMR Spectrum (CDCl_3_, 101 MHz) of **BtP3**


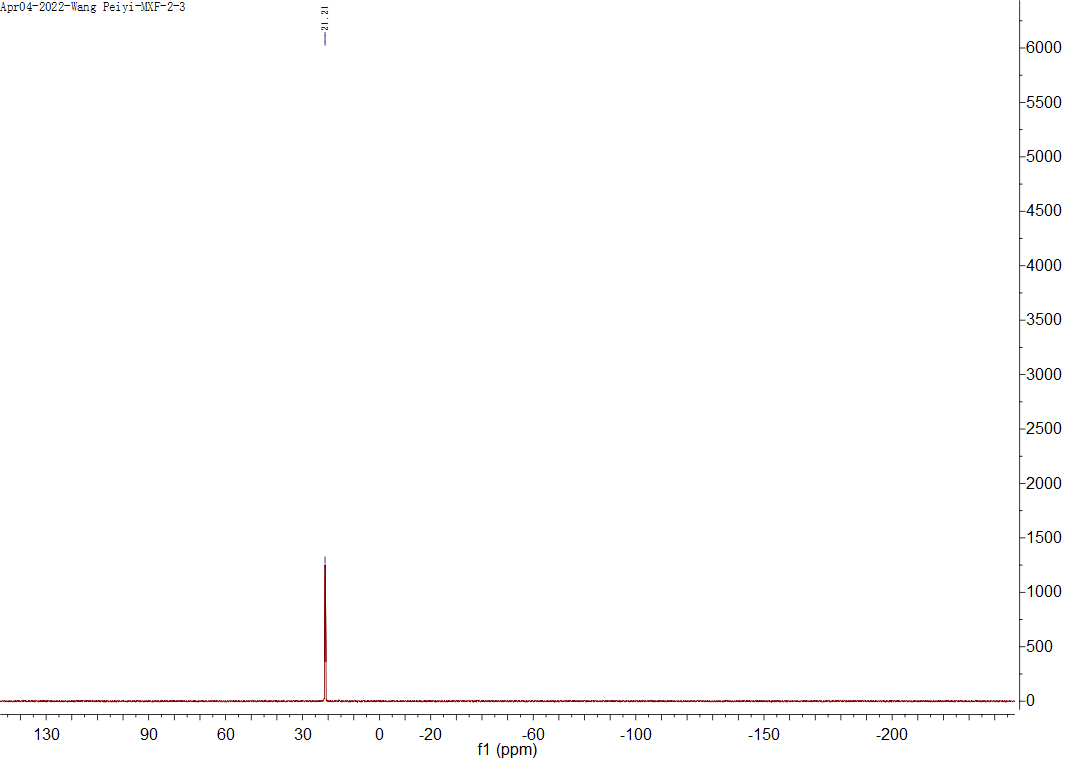


**Figure S36.** ^31^P NMR Spectrum (CDCl_3_, 162 MHz) of **BtP3**

**Figure S37.** HRMS Spectrum of Target Compound **BtP3**

**_
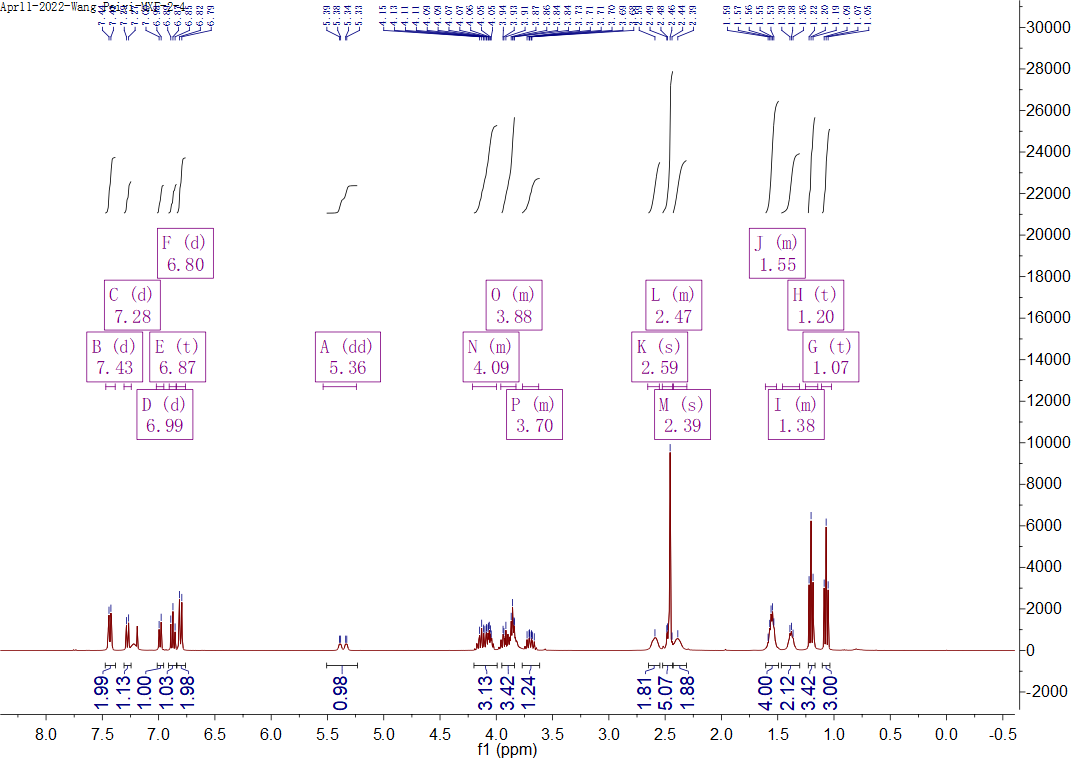
_Figure S38.** ^1^H NMR Spectrum (CDCl_3_, 400 MHz) of **BtP4**

**_
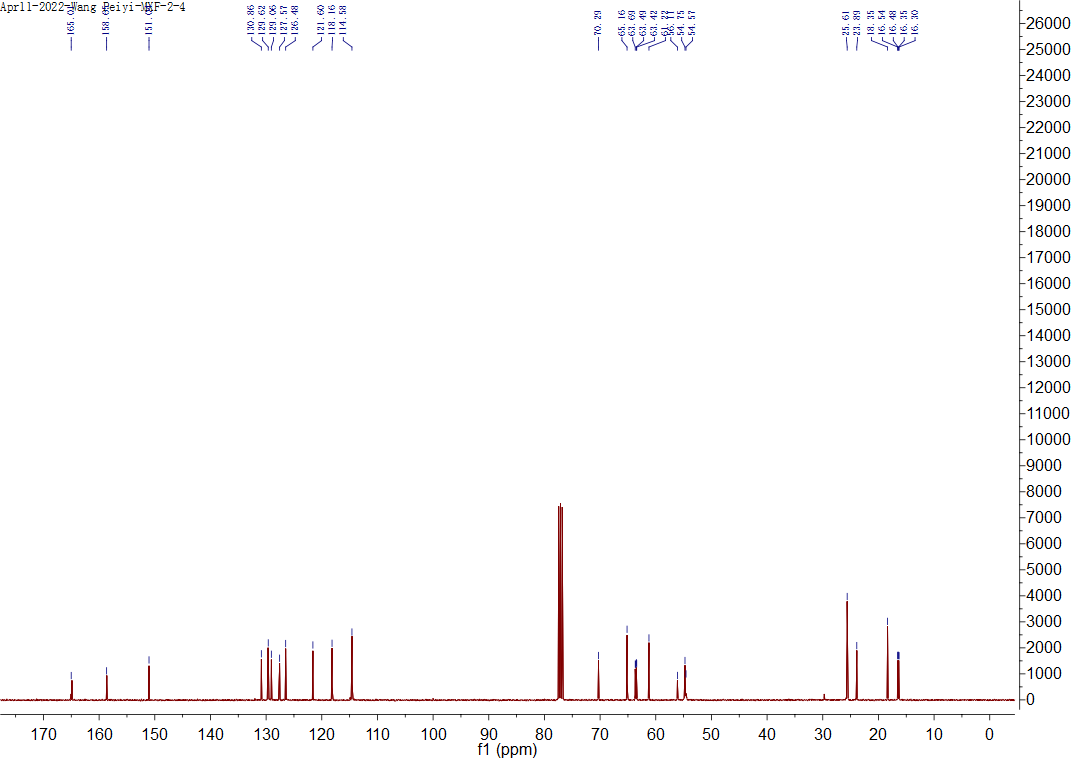
_Figure S39.** ^13^C NMR Spectrum (CDCl_3_, 101 MHz) of **BtP4**


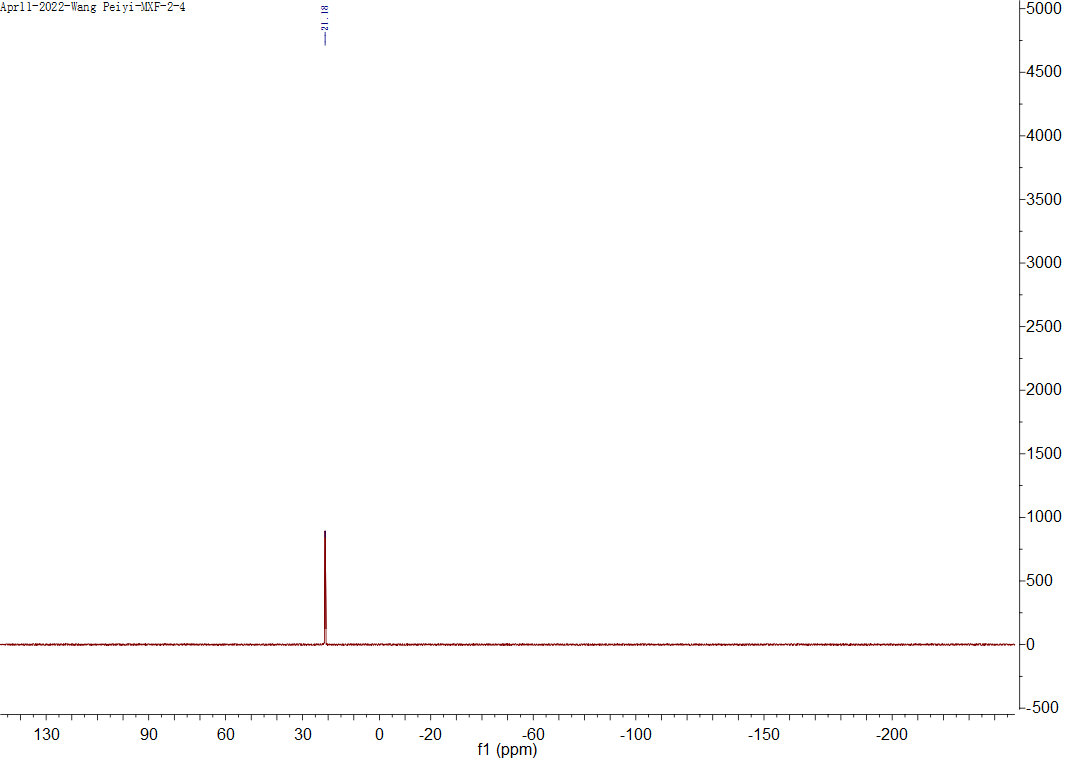


**Figure S40.** ^31^P NMR Spectrum (CDCl_3_, 162 MHz) of **BtP4**

**Figure S41.** HRMS Spectrum of Target Compound **BtP4**


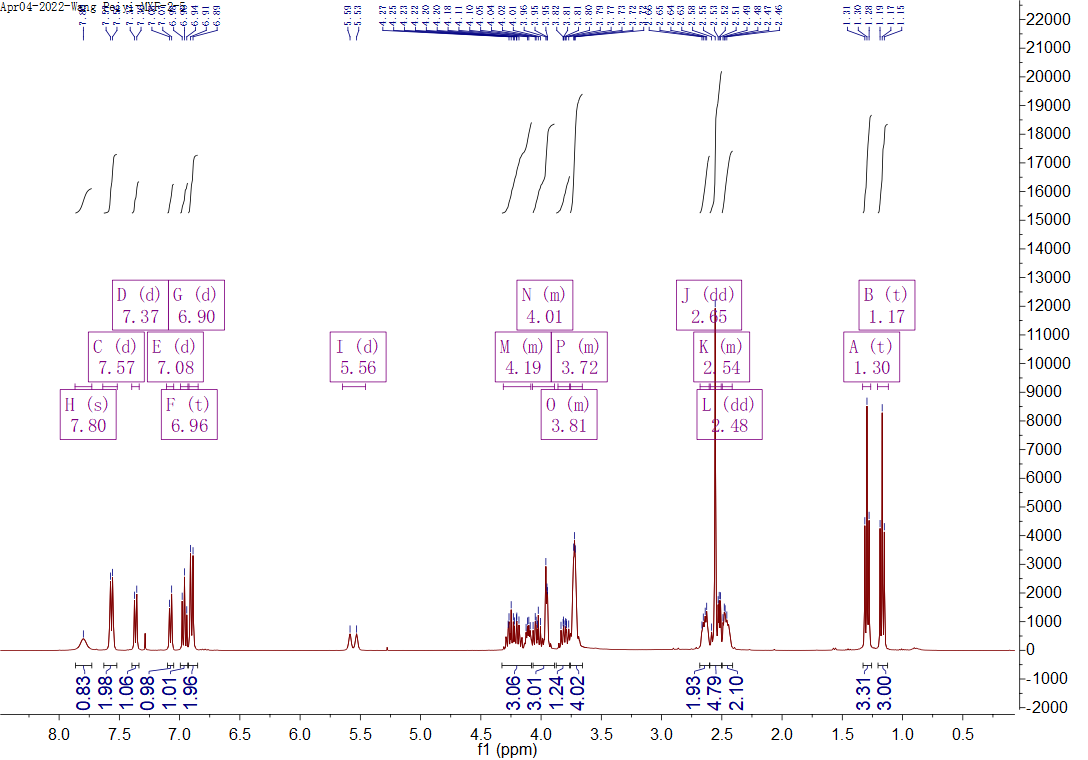
**Figure S42.** ^1^H NMR Spectrum (CDCl_3_, 400 MHz) of **BtP5**

**_
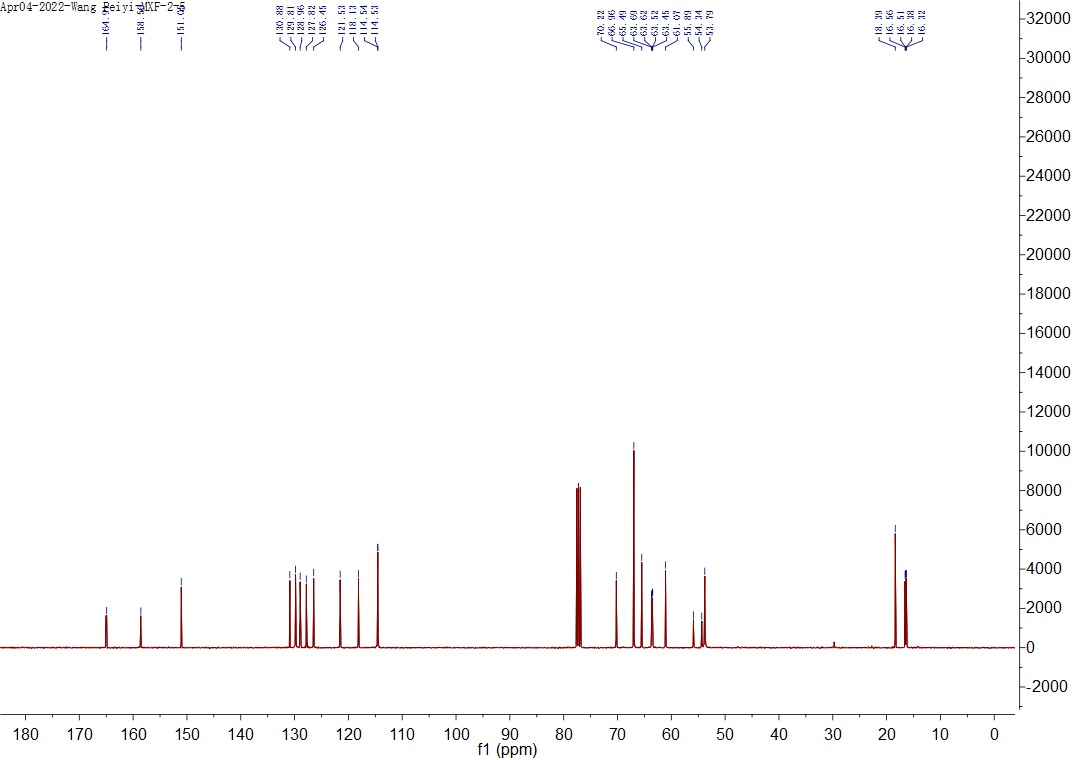
_Figure S43.** ^13^C NMR Spectrum (CDCl_3_, 101 MHz) of **BtP5**


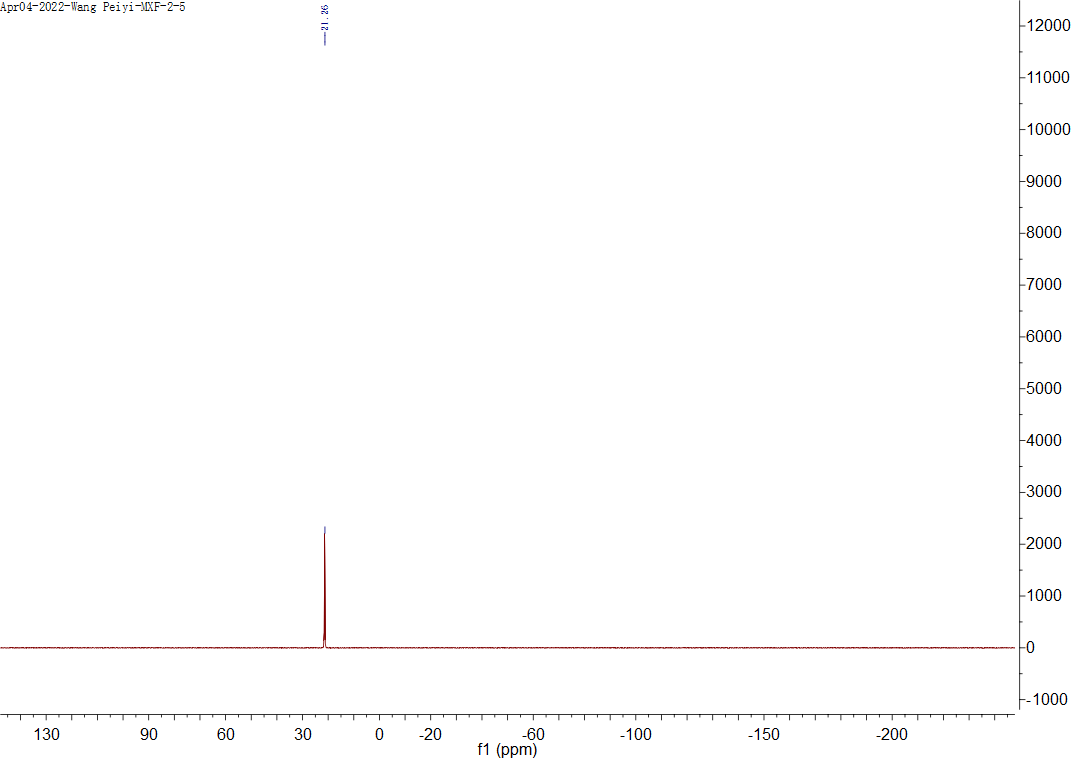


**Figure S44.** ^31^P NMR Spectrum (CDCl_3_, 162 MHz) of **BtP5**

**Figure S45.** HRMS Spectrum of Target Compound **BtP5**


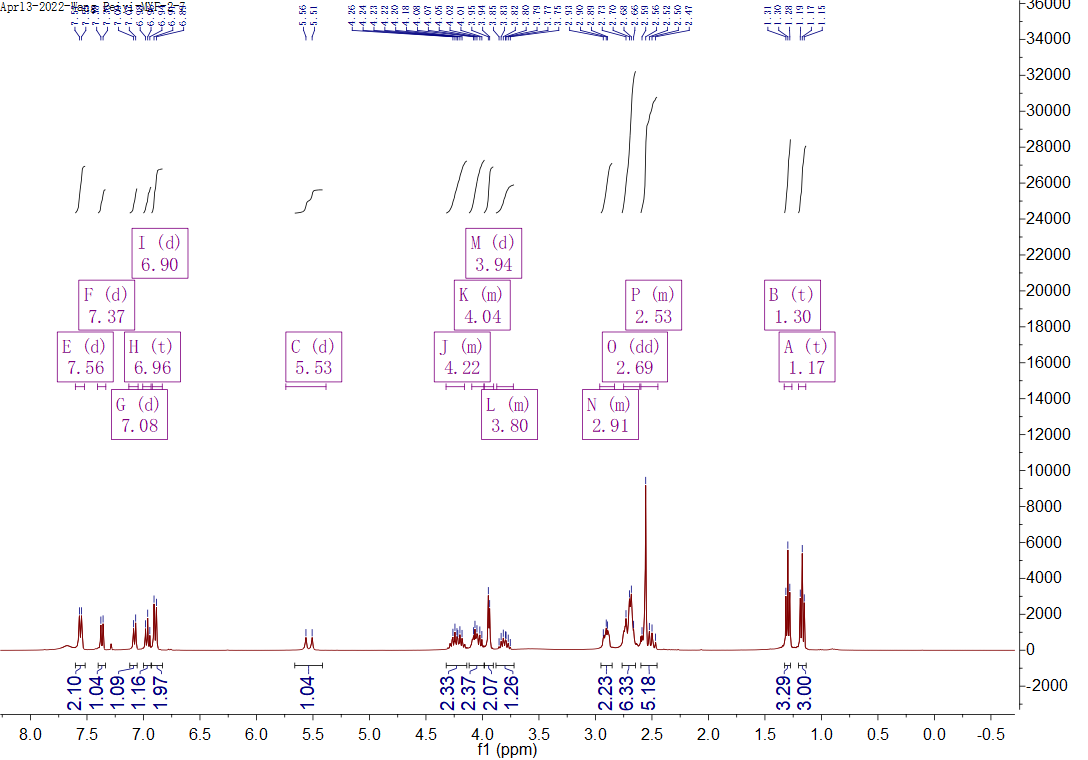
**Figure S46.** ^1^H NMR Spectrum (CDCl_3_, 400 MHz) of **BtP6**

**_
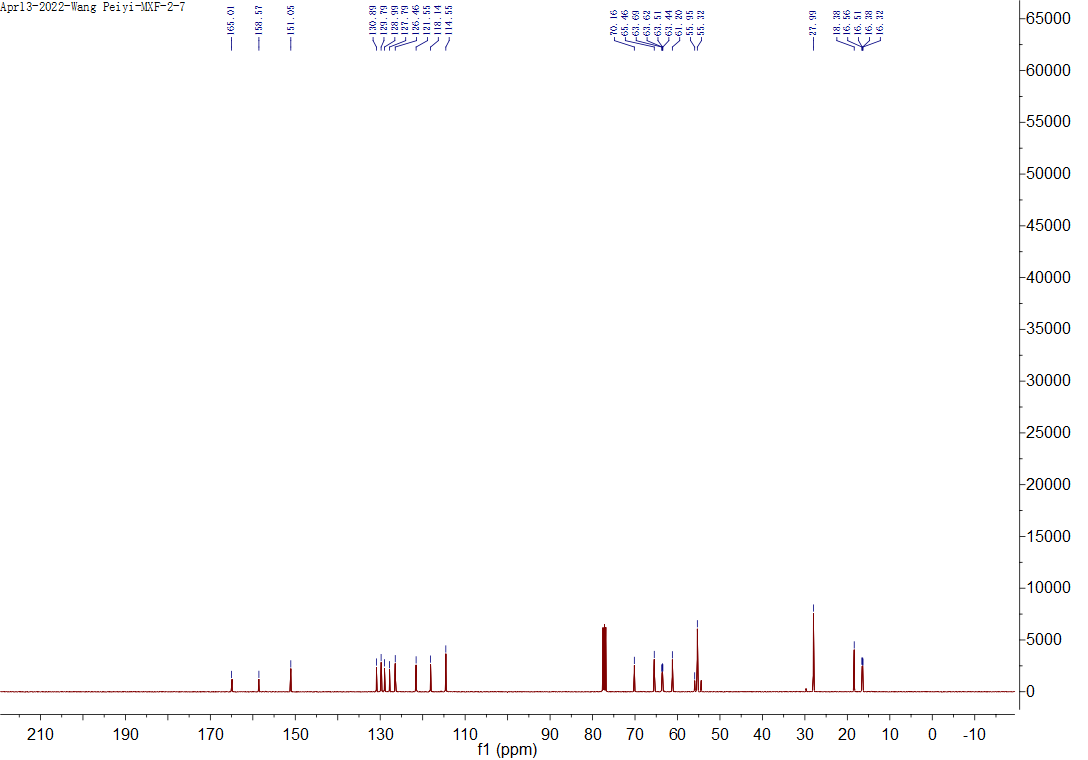
_Figure S47.** ^13^C NMR Spectrum (CDCl_3_, 101 MHz) of **BtP6**


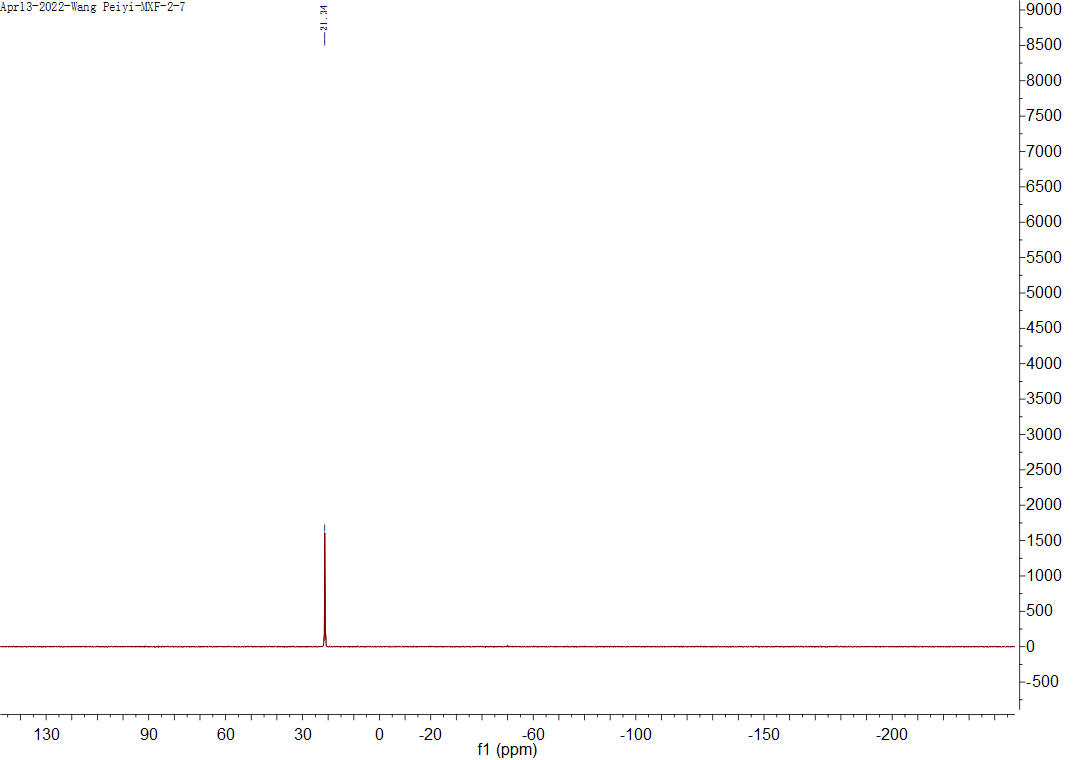


**Figure S48.** ^31^P NMR Spectrum (CDCl_3_, 162 MHz) of **BtP6**

**Figure S49.** HRMS Spectrum of Target Compound **BtP6**


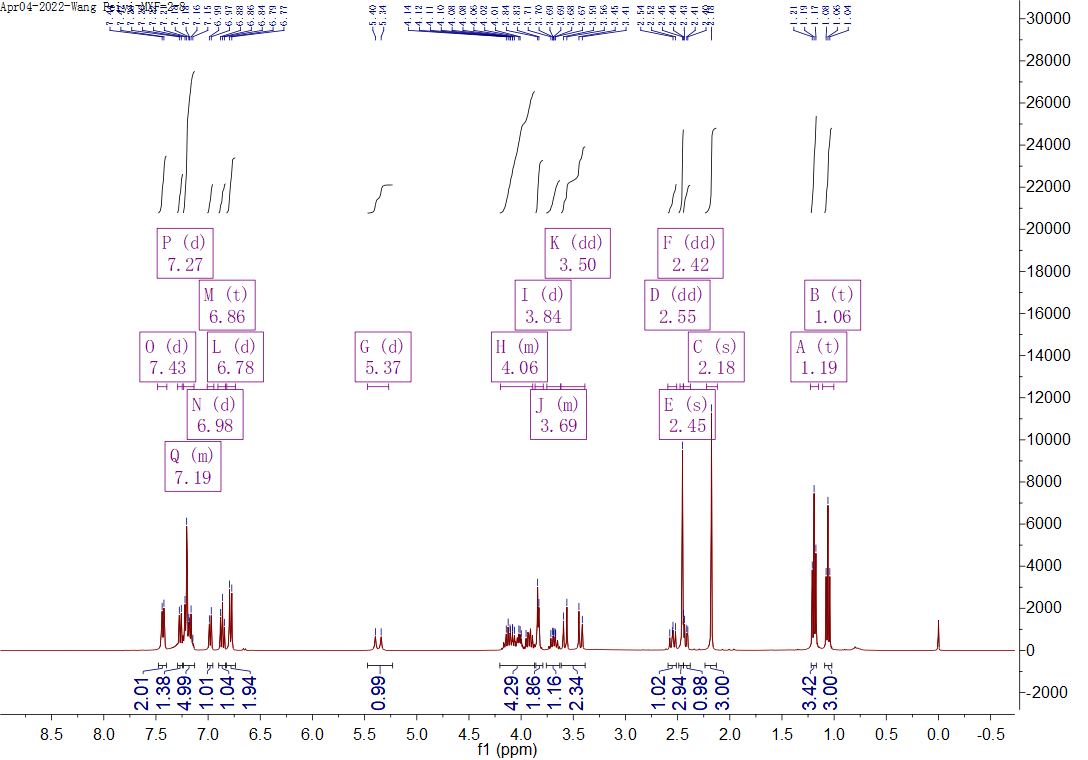
**Figure S50.** ^1^H NMR Spectrum (CDCl_3_, 400 MHz) of **BtP7**

**_
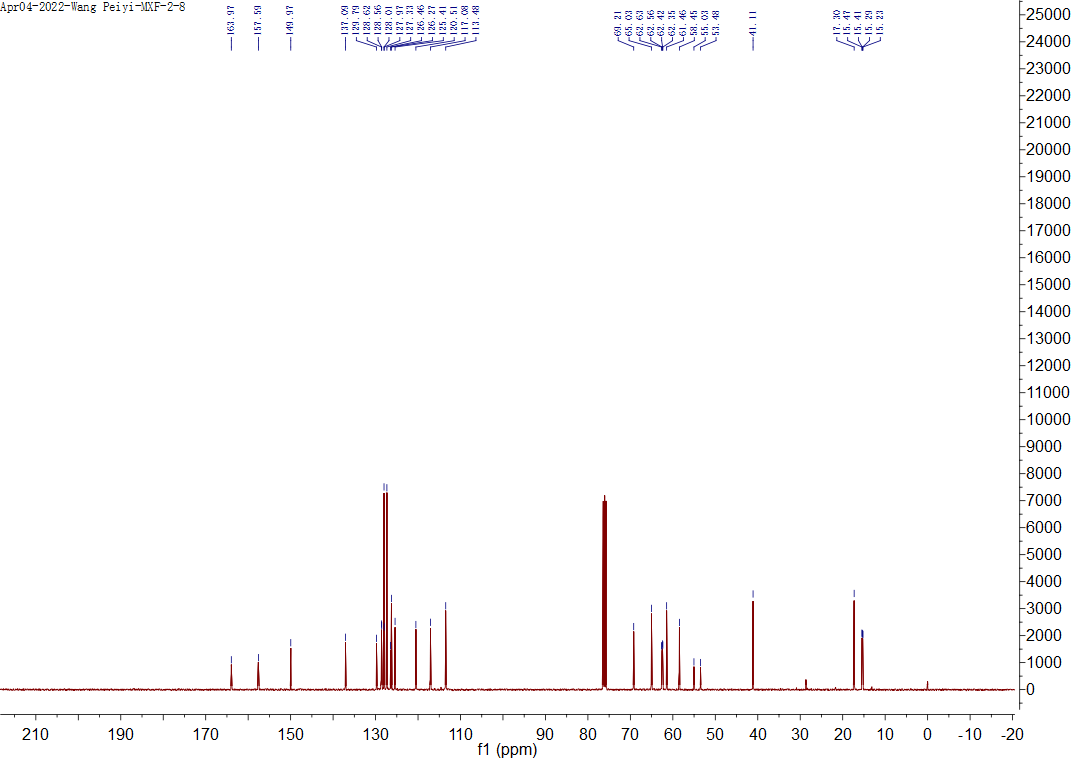
_Figure S51.** ^13^C NMR Spectrum (CDCl_3_, 101 MHz) of **BtP7**


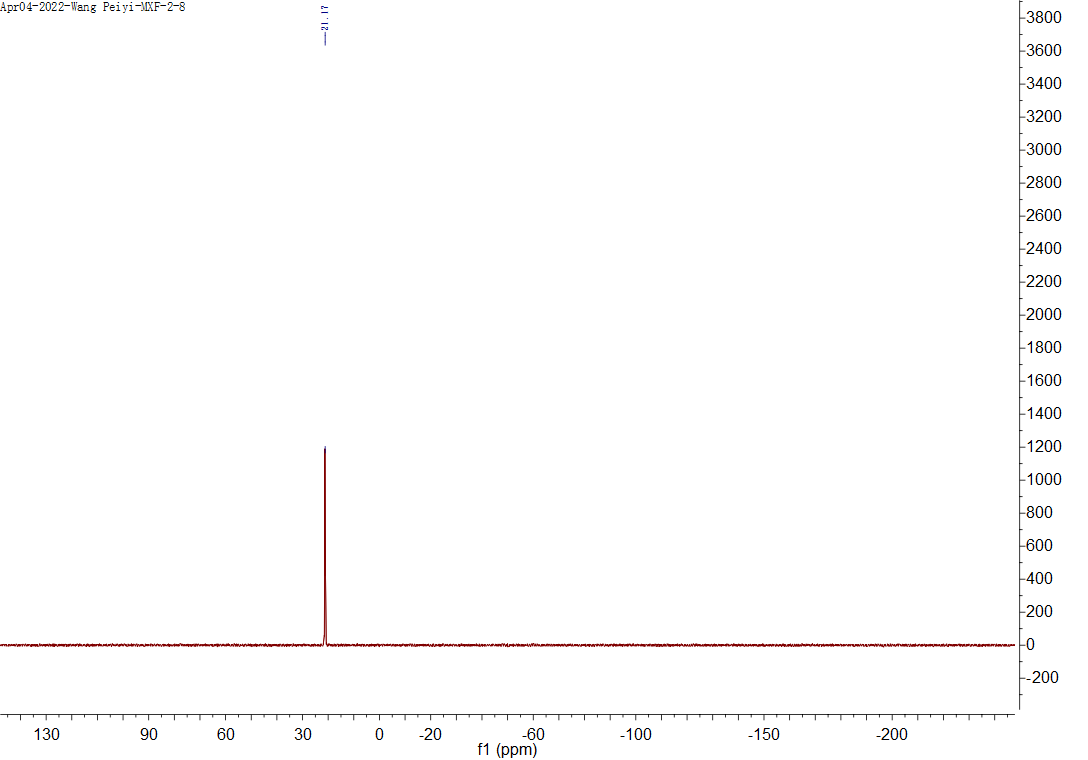


**Figure S52.** ^31^P NMR Spectrum (CDCl_3_, 162 MHz) of **BtP7**

**Figure S53.** HRMS Spectrum of Target Compound **BtP7**


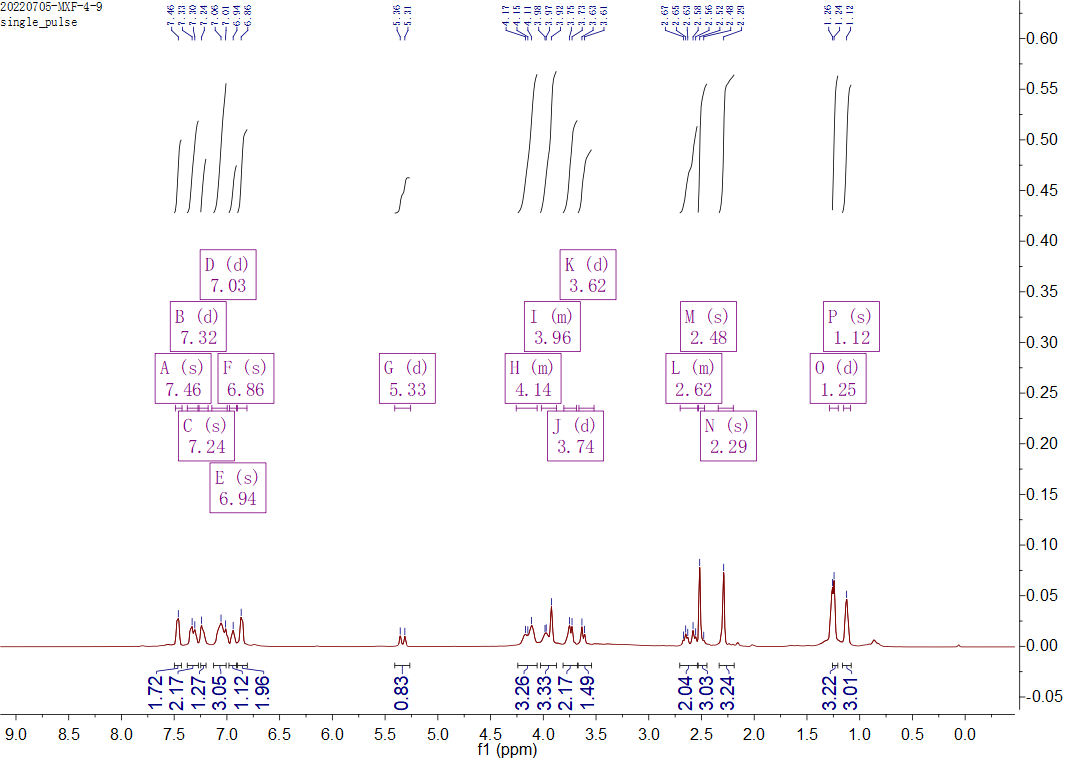
**Figure S54.** ^1^H NMR Spectrum (CDCl_3_, 500 MHz) of **BtP8**

**_
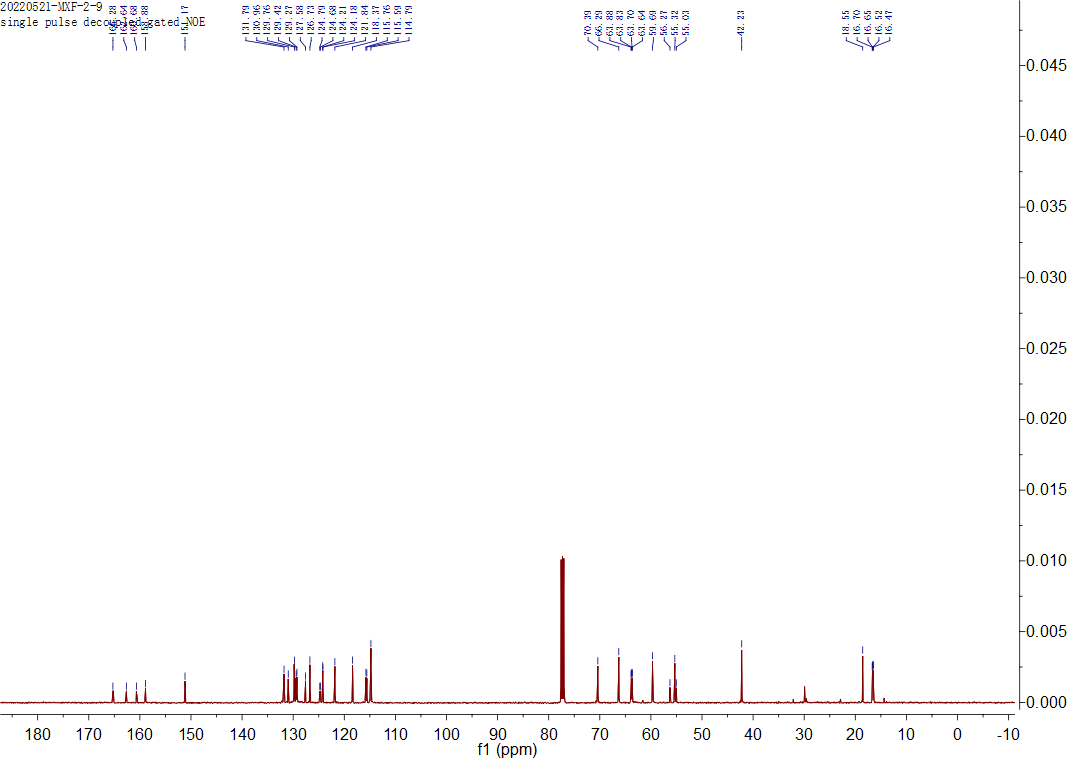
_Figure S55.** ^13^C NMR Spectrum (CDCl_3_, 126 MHz) of **BtP8**


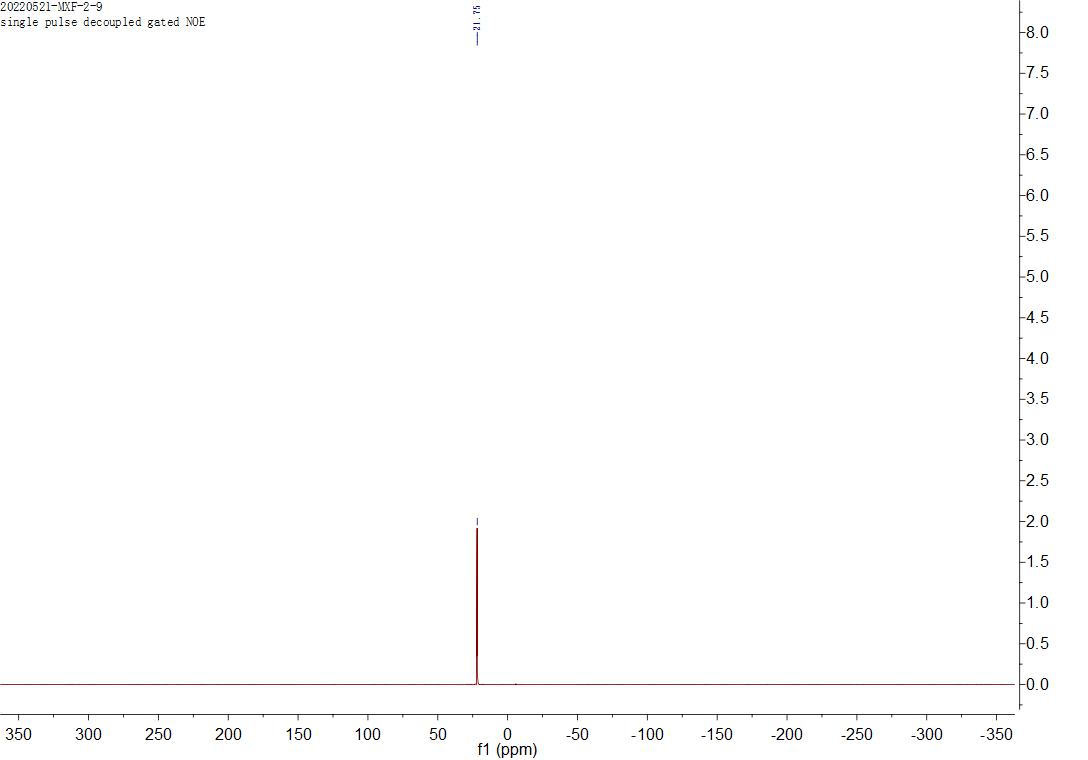


**Figure S56.** ^31^P NMR Spectrum (CDCl_3_, 202 MHz) of **BtP8**

**
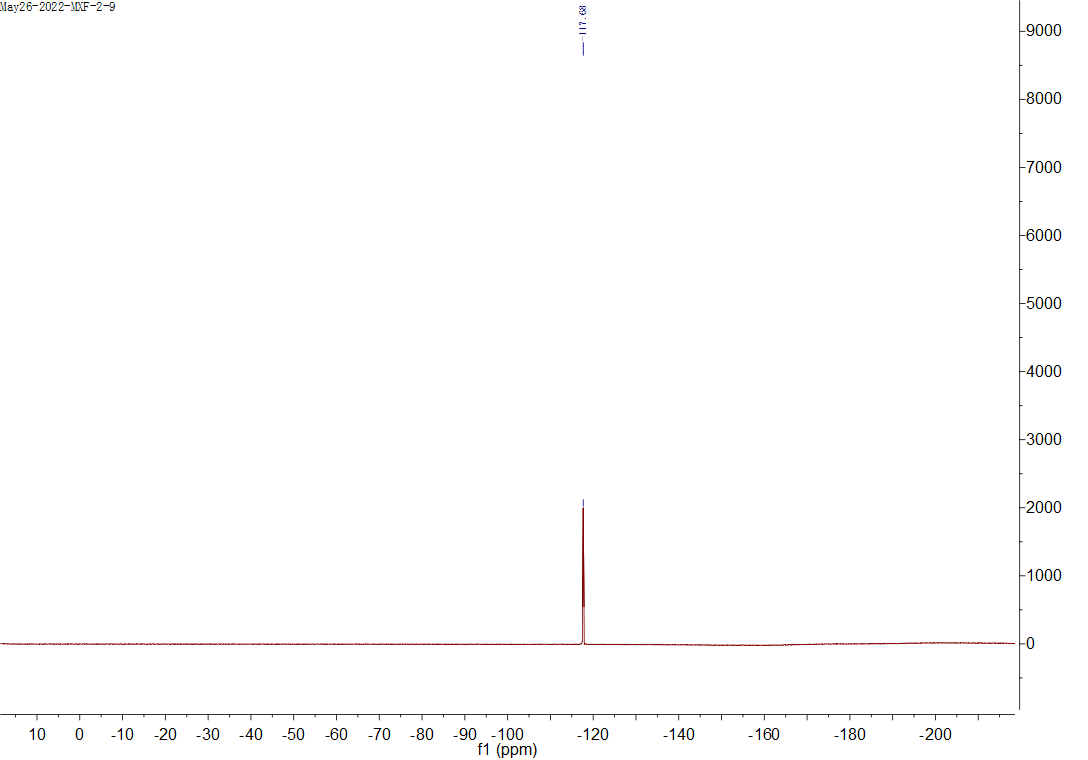
**

**Figure S57.** ^19^F NMR Spectrum (CDCl_3_, 376 MHz) of **BtP8**

**Figure S58.** HRMS Spectrum of Target Compound **BtP8**


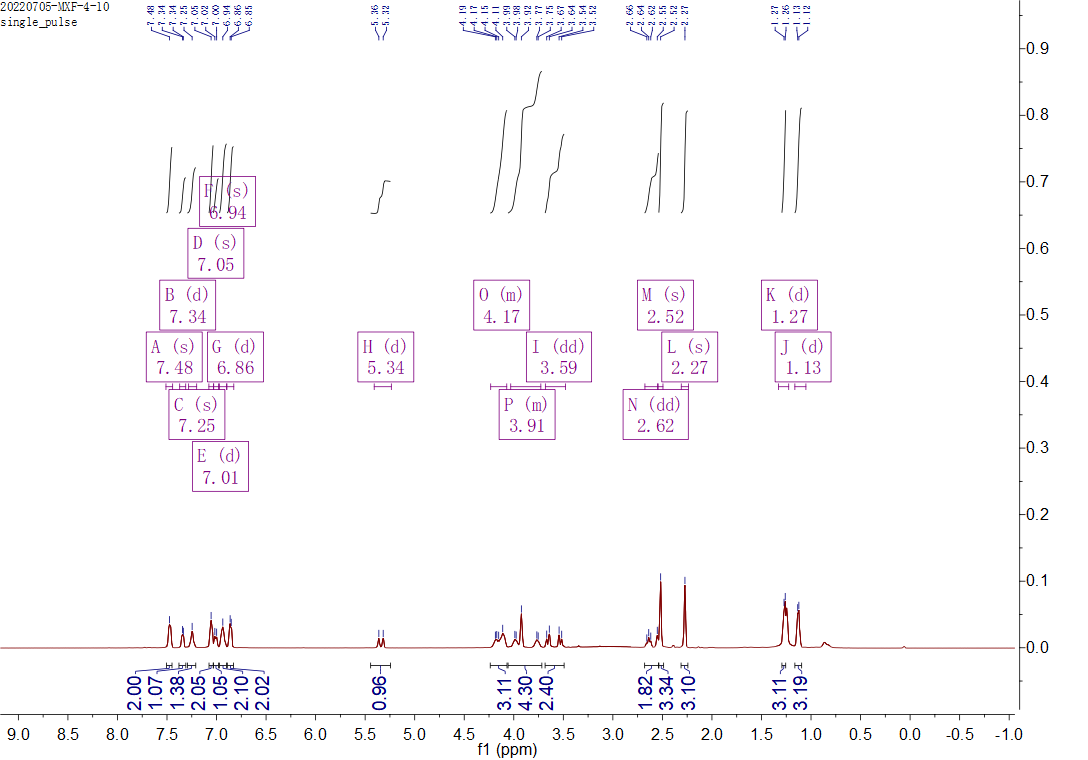
**Figure S59.** ^1^H NMR Spectrum (CDCl_3_, 500 MHz) of **BtP9**

**_
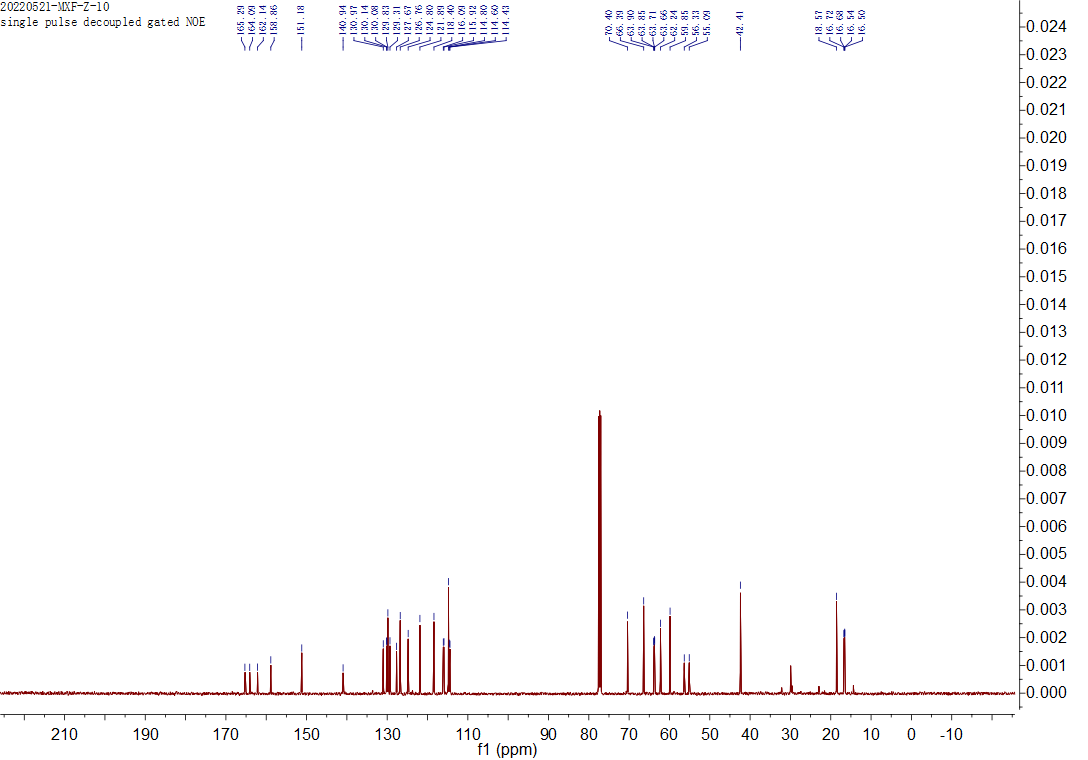
_Figure S60.** ^13^C NMR Spectrum (CDCl_3_, 126 MHz) of **BtP9**


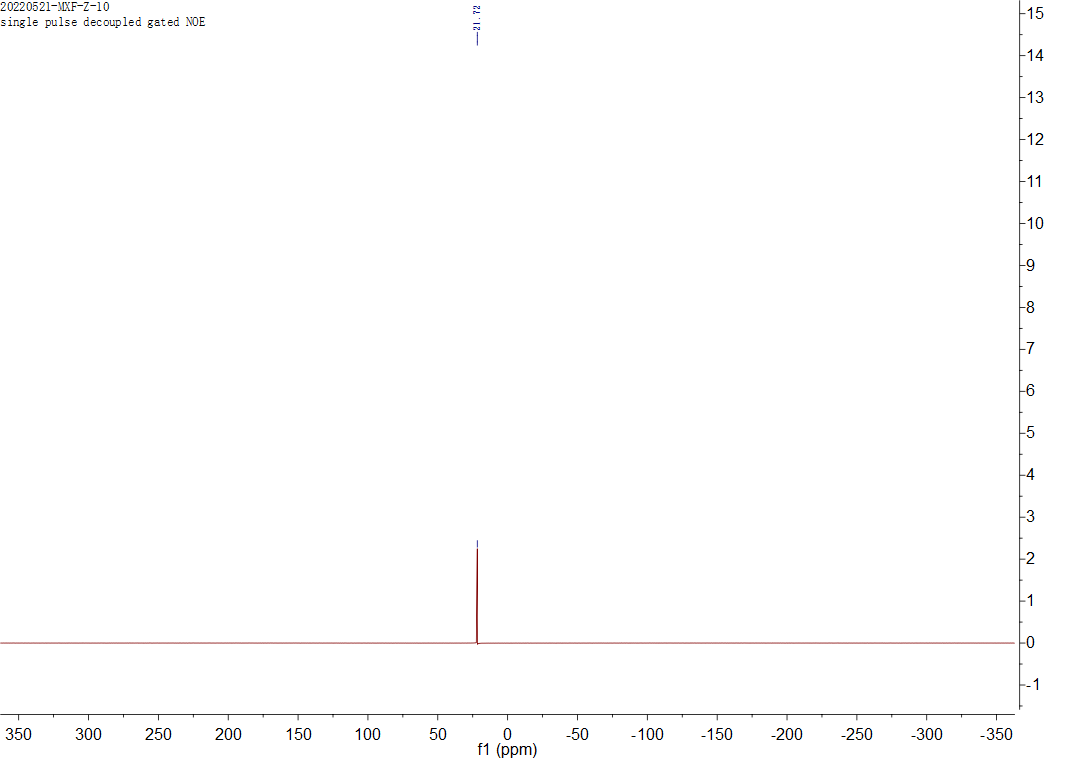


**Figure S61.** ^31^P NMR Spectrum (CDCl_3_, 202 MHz) of **BtP9**

**
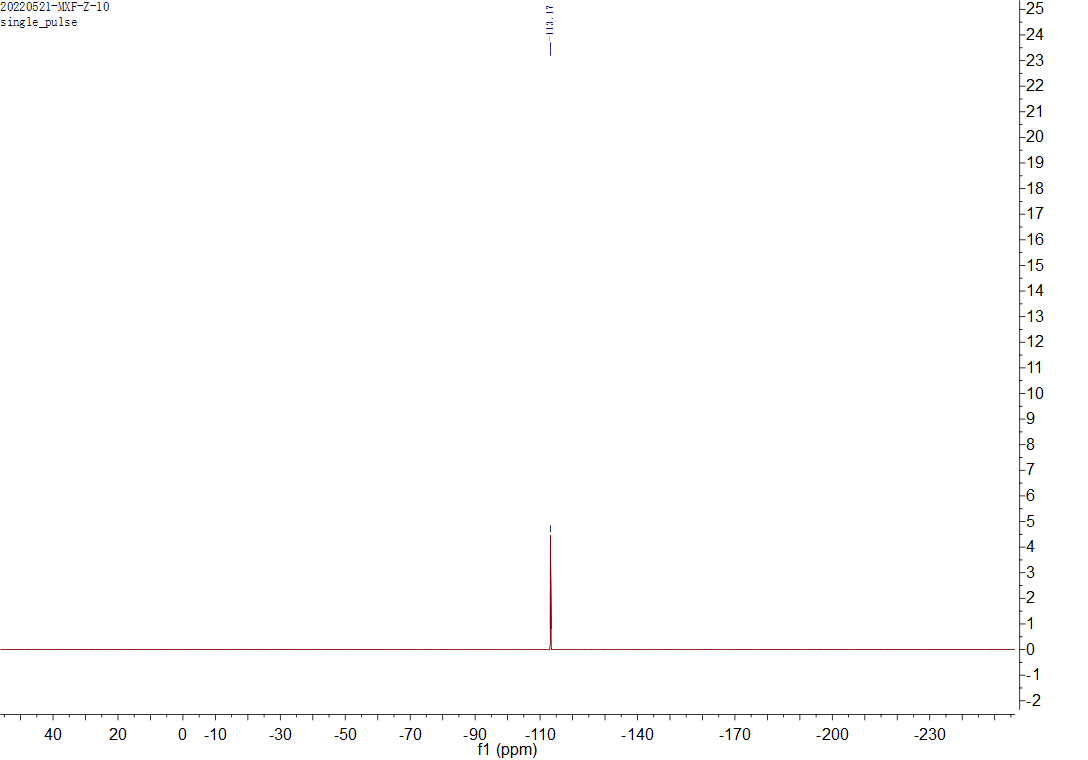
**

**Figure S62.** ^19^F NMR Spectrum (CDCl_3_, 471 MHz) of **BtP9**

**Figure S63.** HRMS Spectrum of Target Compound **BtP9**


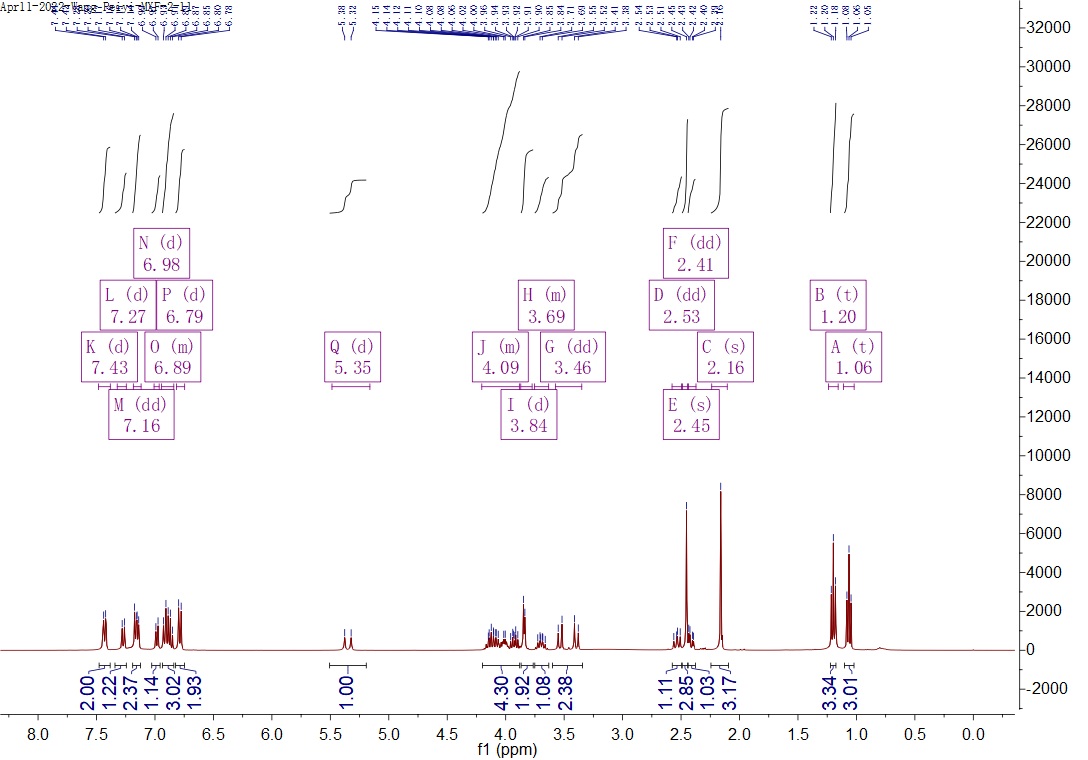
**Figure S64.** ^1^H NMR Spectrum (CDCl_3_, 400 MHz) of **BtP10**

**_
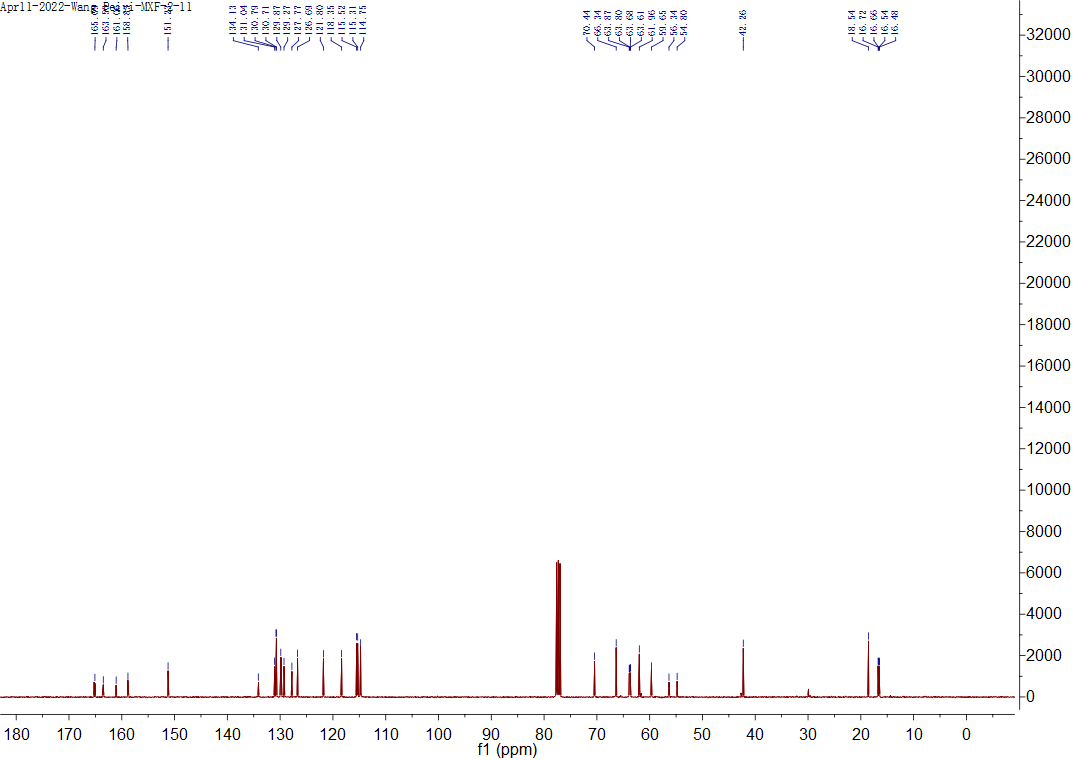
_Figure S65.** ^13^C NMR Spectrum (CDCl_3_, 101 MHz) of **BtP10**


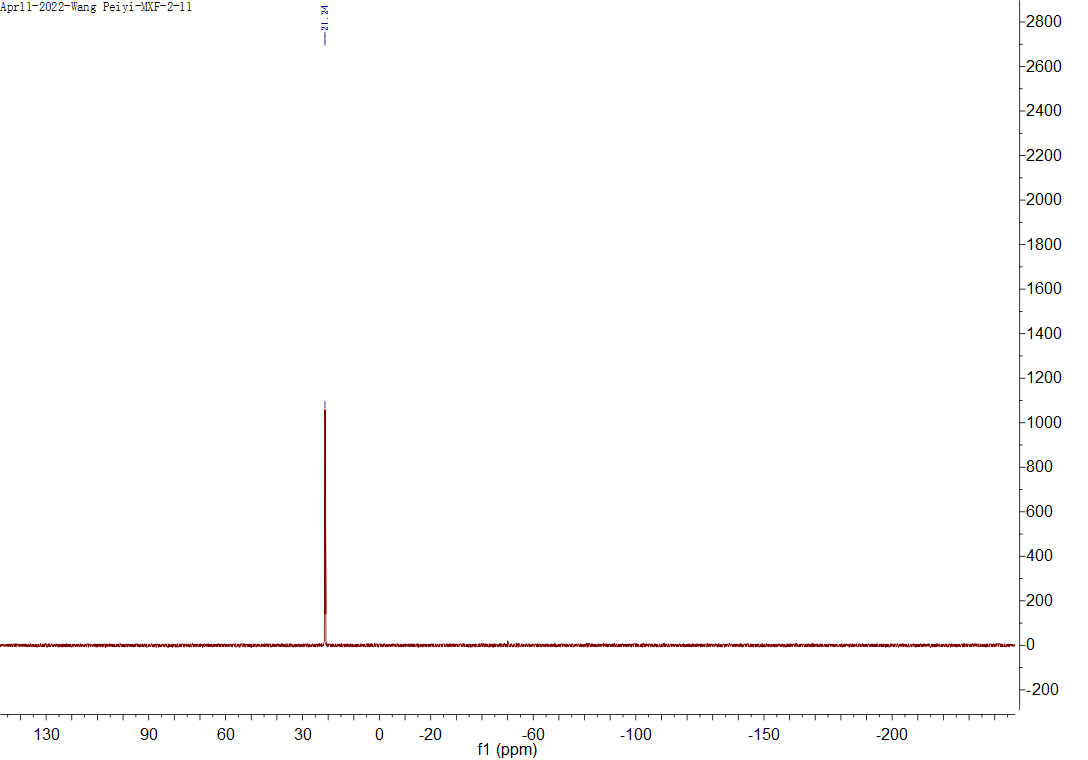


**Figure S66.** ^31^P NMR Spectrum (CDCl_3_, 162 MHz) of **BtP10**

**
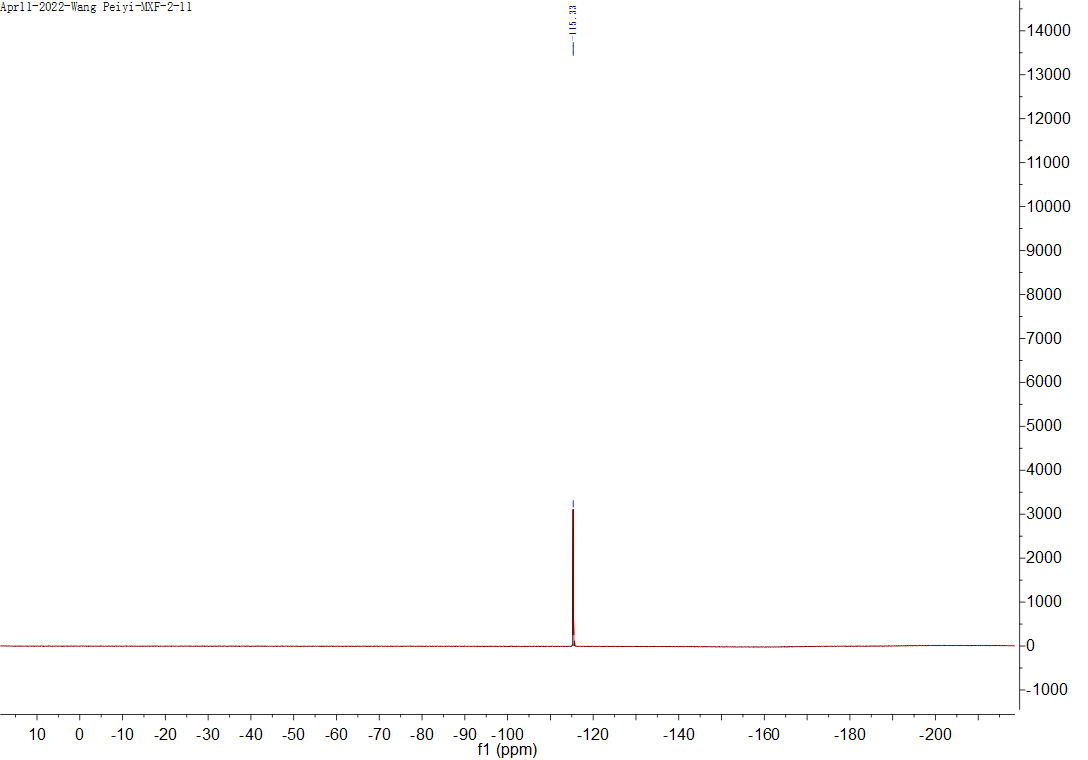
**

**Figure S67.** ^19^F NMR Spectrum (CDCl_3_, 376 MHz) of **BtP10**

**Figure S68.** HRMS Spectrum of Target Compound **BtP10**


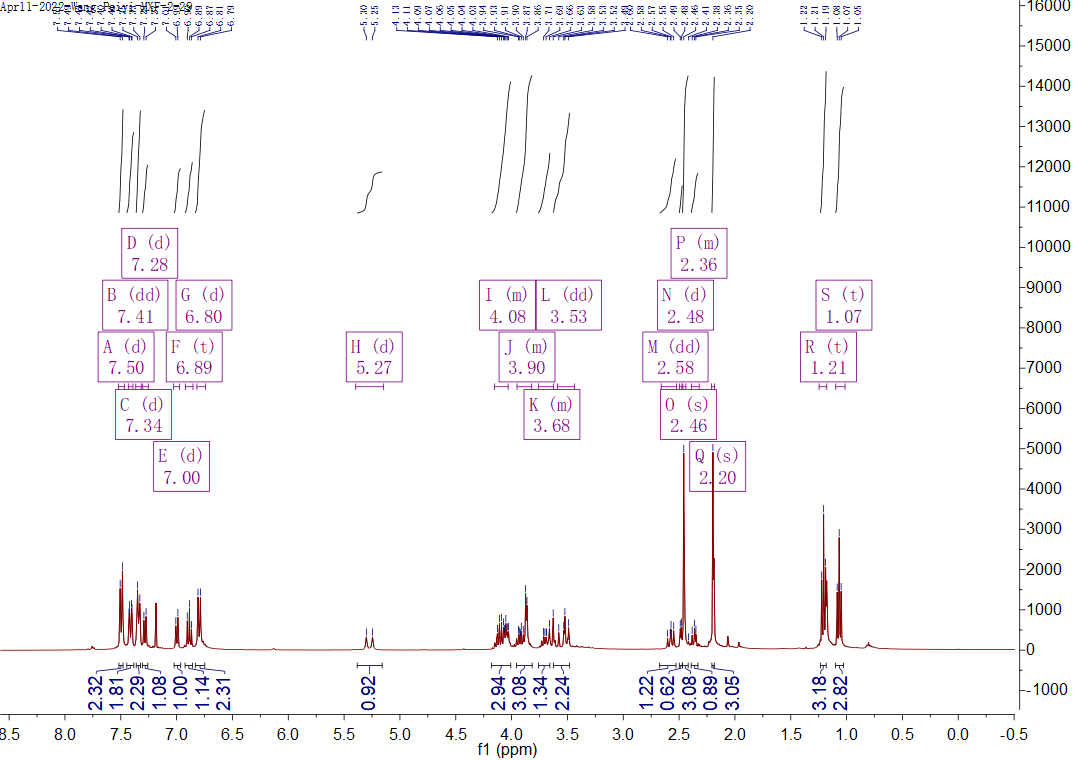
**Figure S69.** ^1^H NMR Spectrum (CDCl_3_, 400 MHz) of **BtP11**

**_
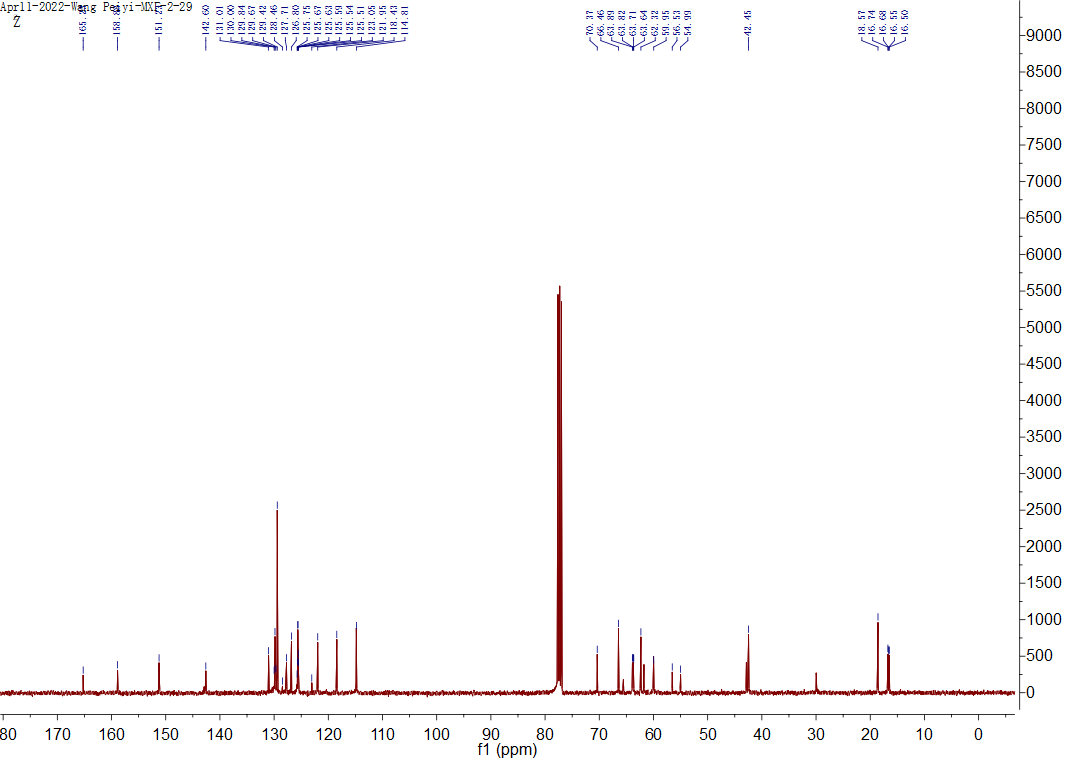
_Figure S70.** ^13^C NMR Spectrum (CDCl_3_, 101 MHz) of **BtP11**


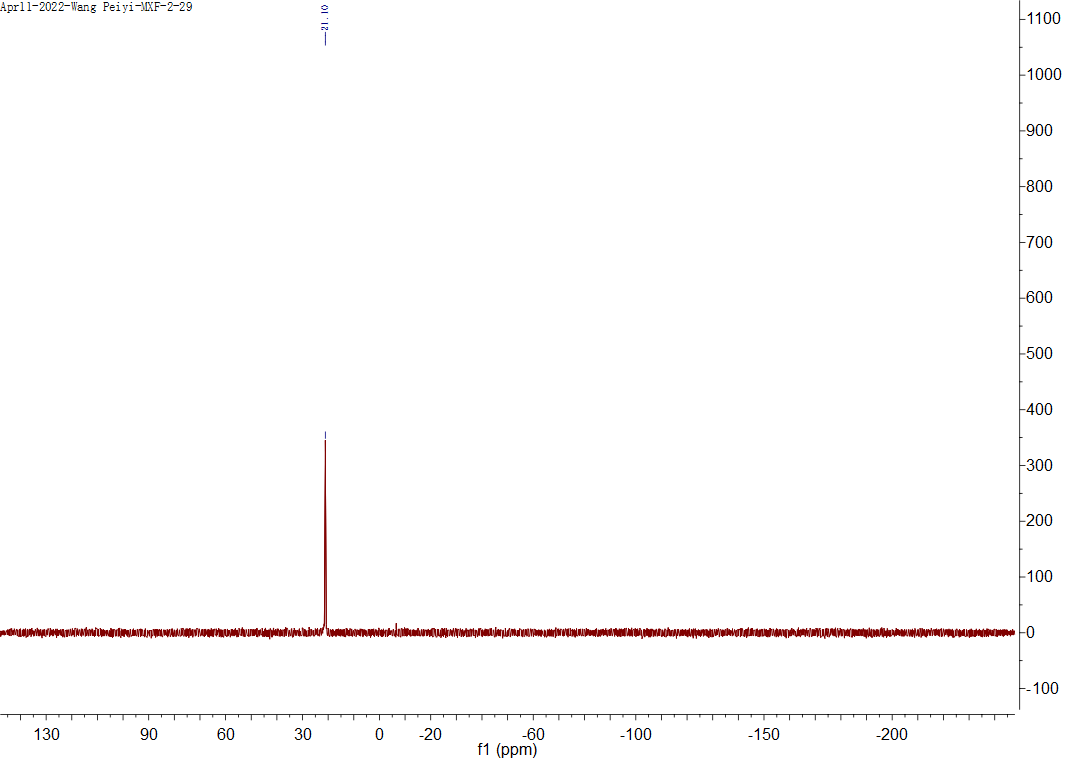


**Figure S71.** ^31^P NMR Spectrum (CDCl_3_, 162 MHz) of **BtP11**

**
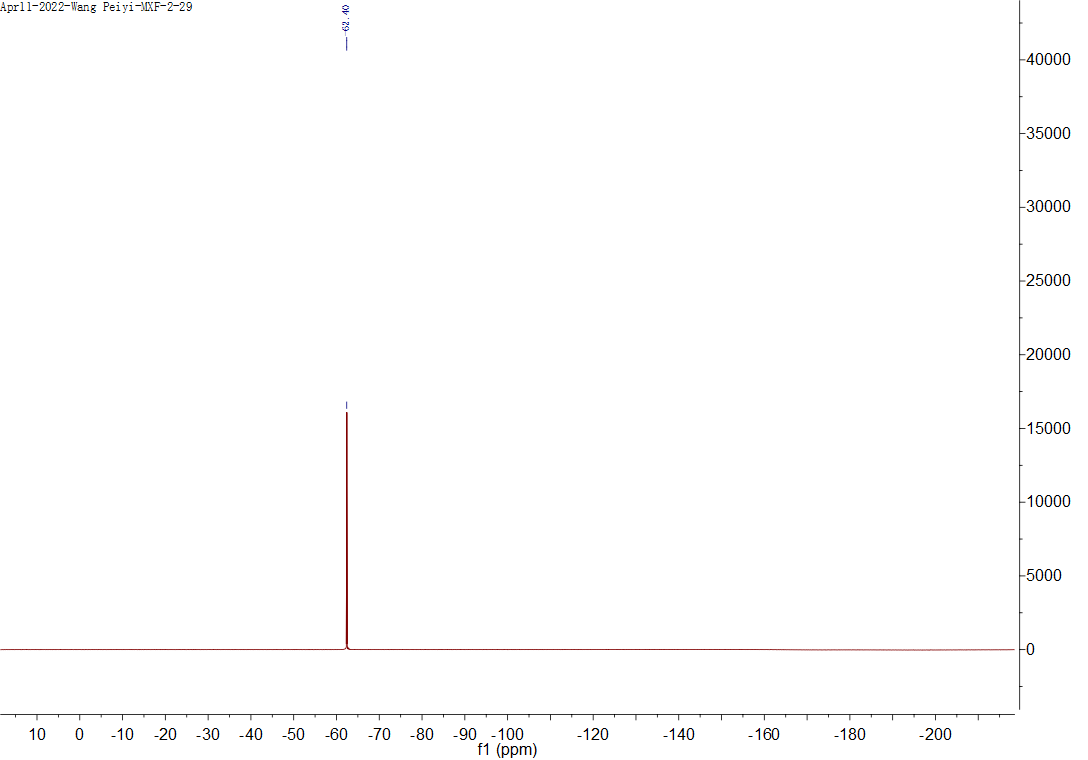
**

**Figure S72.** ^19^F NMR Spectrum (CDCl_3_, 376 MHz) of **BtP11**

**Figure S73.** HRMS Spectrum of Target Compound **BtP11**

**_
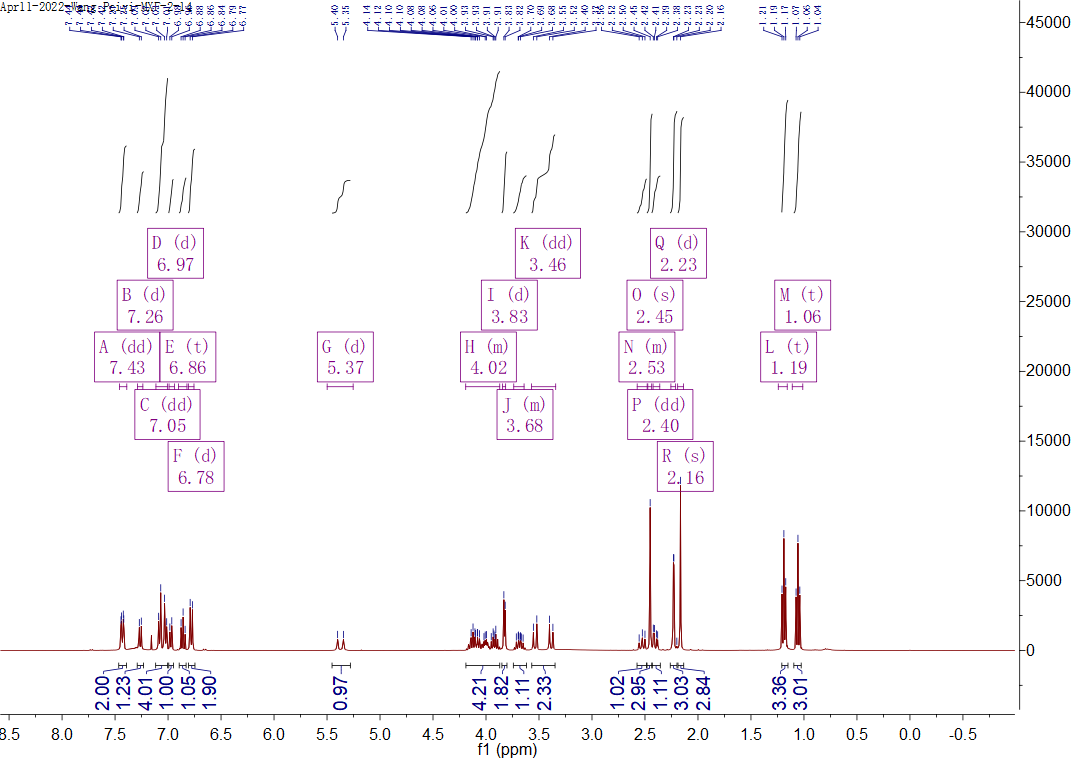
_**

**Figure S74.** ^1^H NMR Spectrum (CDCl_3_, 400 MHz) of **BtP12**

**_
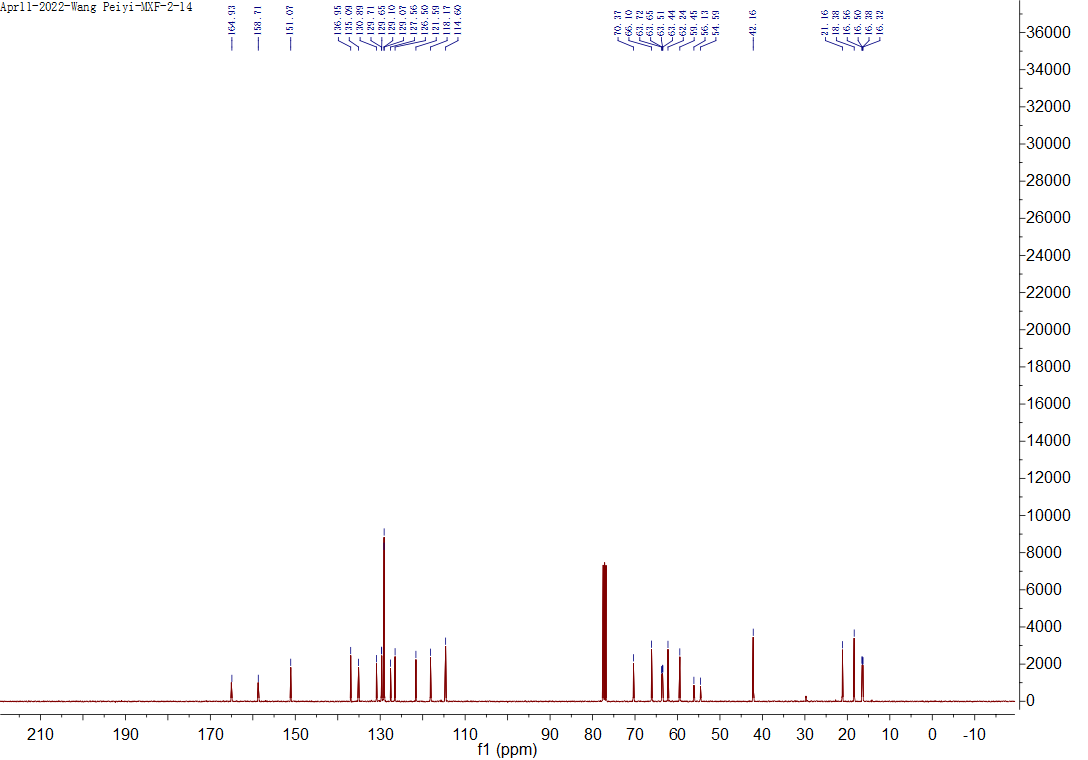
_Figure S75.** ^13^C NMR Spectrum (CDCl_3_, 101 MHz) of **BtP12**


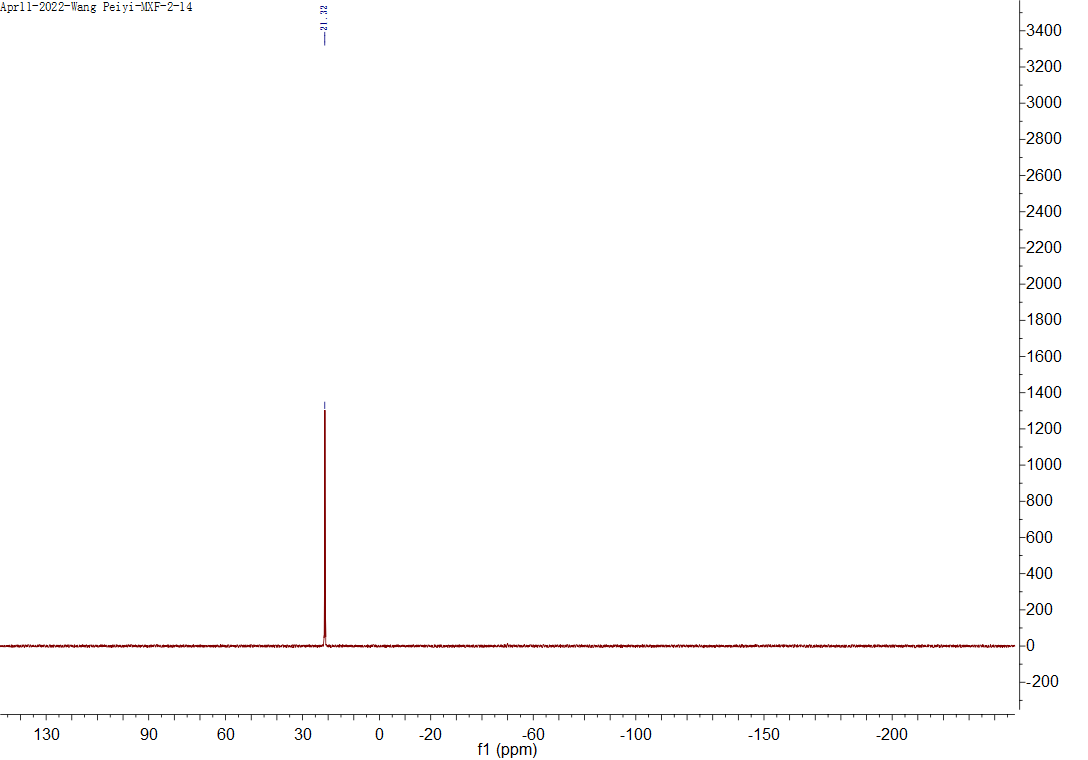


**Figure S76.** ^31^P NMR Spectrum (CDCl_3_, 162 MHz) of **BtP12**

**Figure S77.** HRMS Spectrum of Target Compound **BtP12**


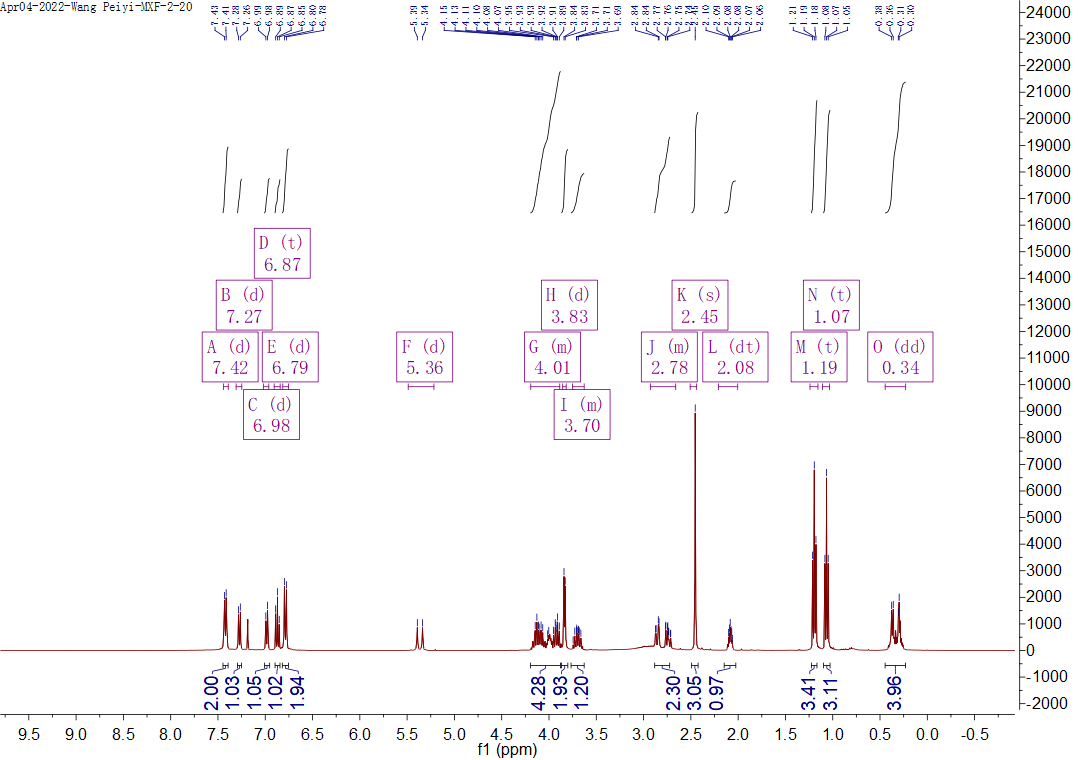
**Figure S78.** ^1^H NMR Spectrum (CDCl_3_, 400 MHz) of **BtP13**

**_
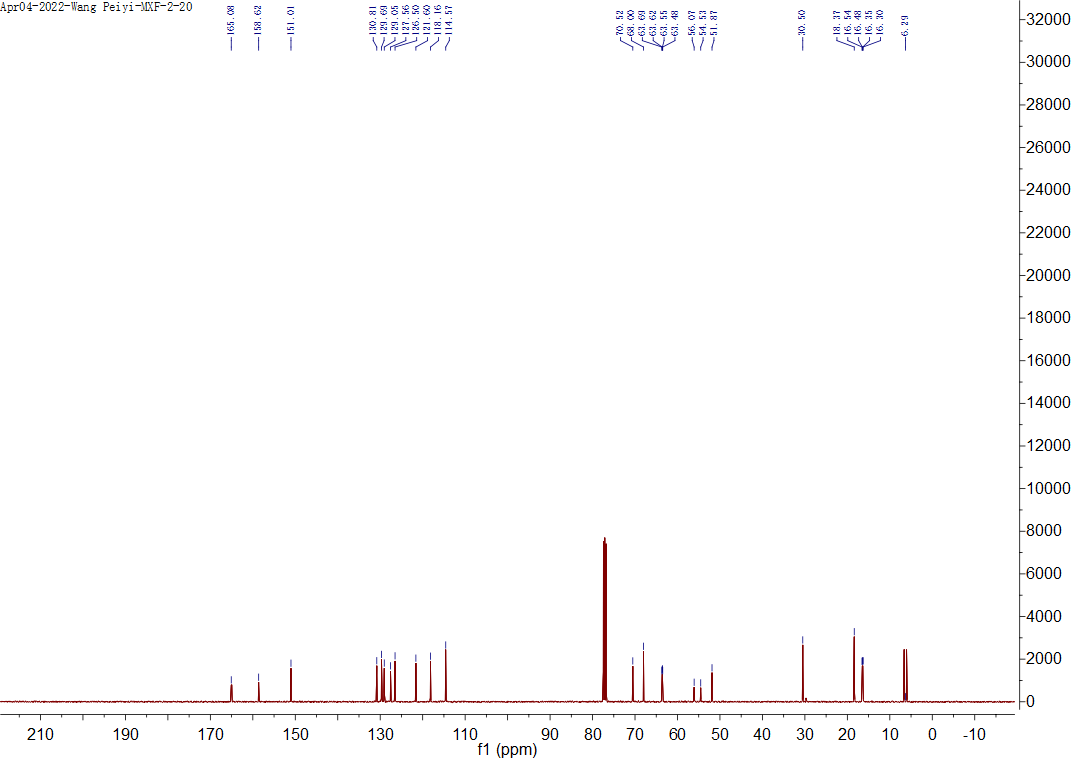
_Figure S79.** ^13^C NMR Spectrum (CDCl_3_, 101 MHz) of **BtP13**


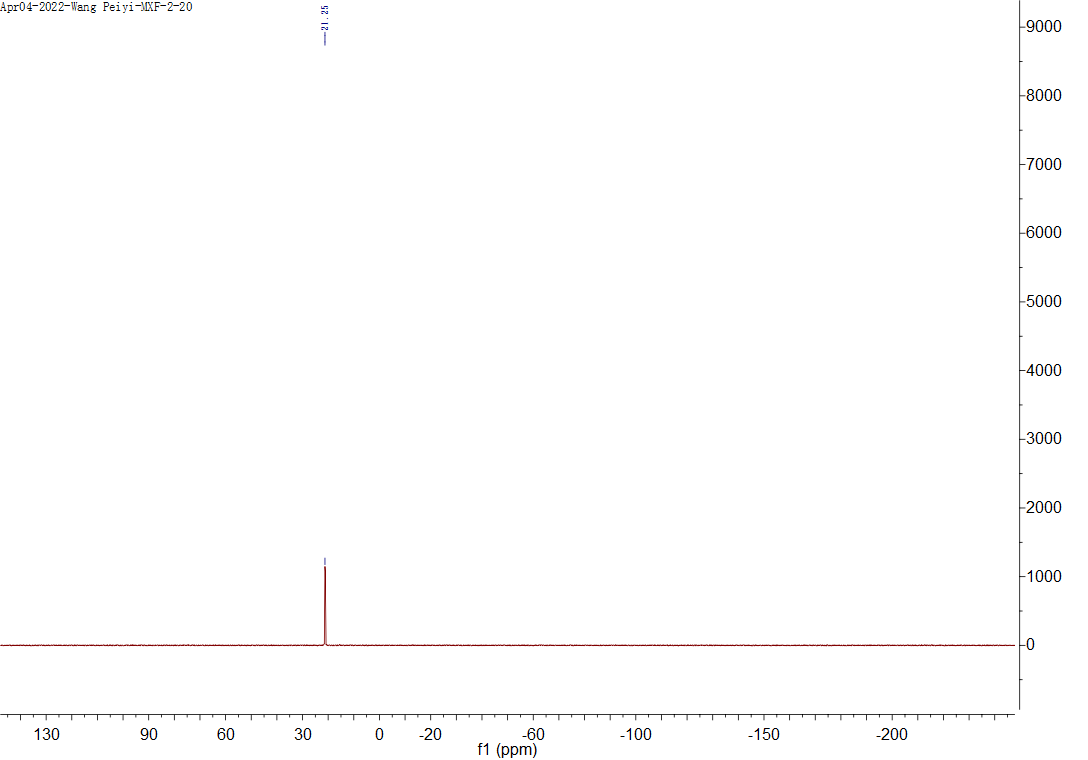


**Figure S80.** ^31^P NMR Spectrum (CDCl_3_, 162 MHz) of **BtP13**

**Figure S81.** HRMS Spectrum of Target Compound **BtP13**


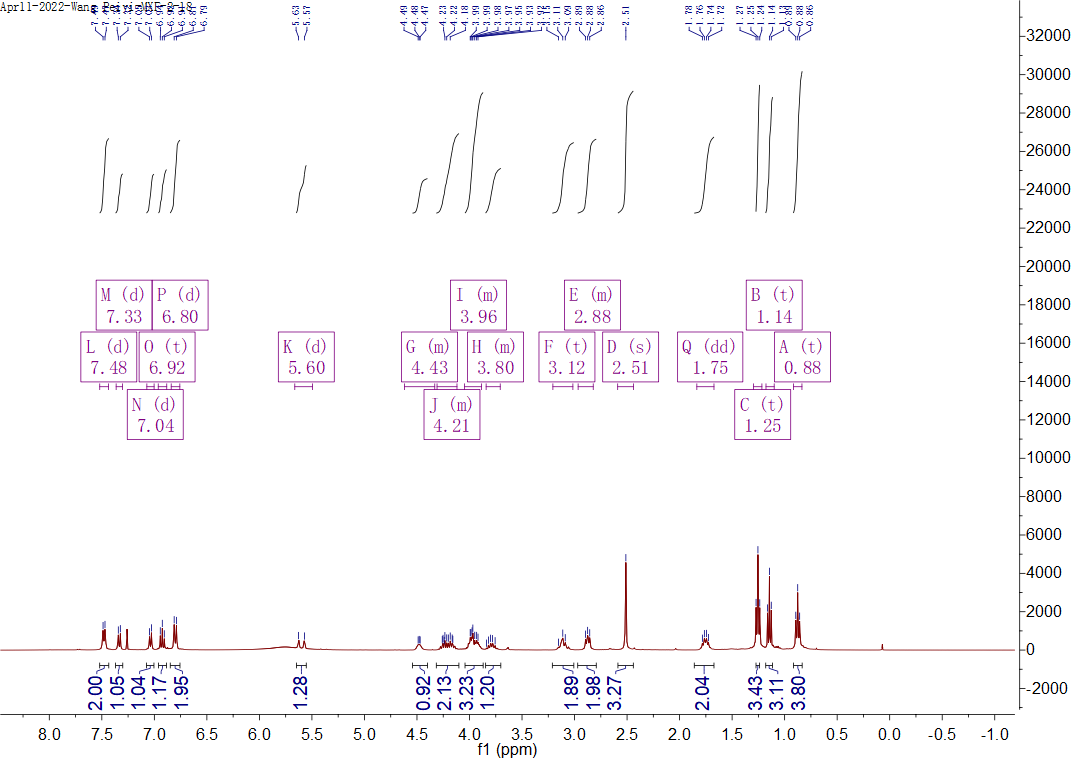
**Figure S82.** ^1^H NMR Spectrum (CDCl_3_, 400 MHz) of **BtP14**

**_
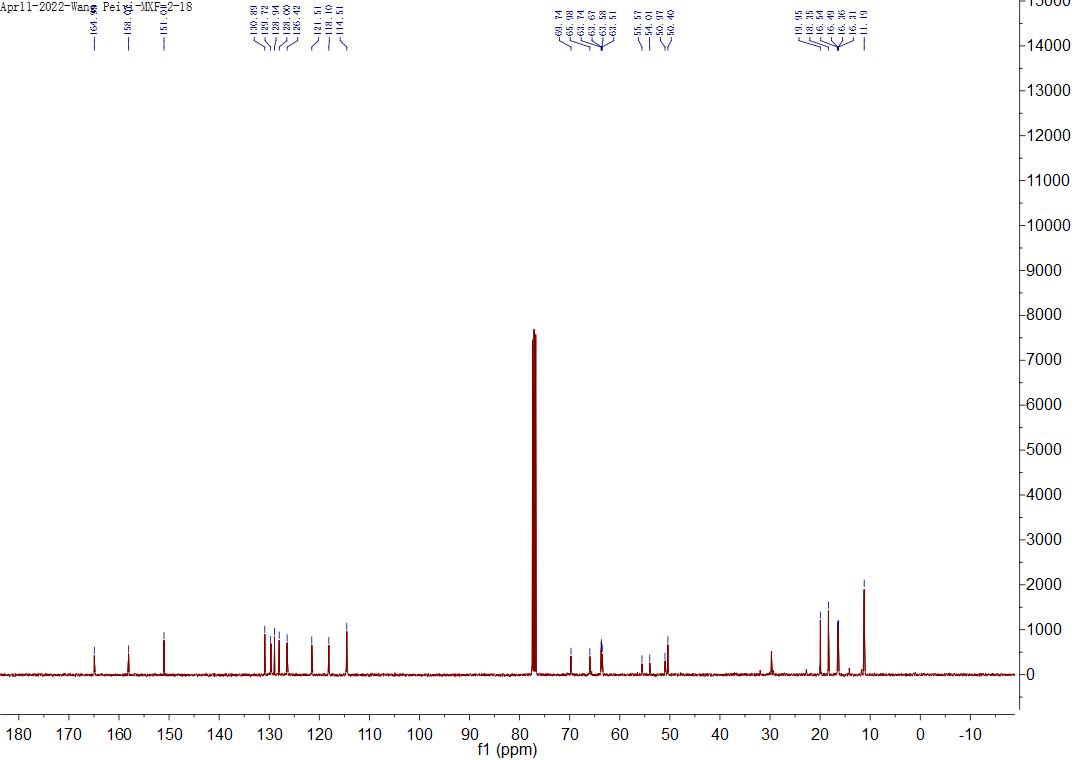
_Figure S83.** ^13^C NMR Spectrum (CDCl_3_, 101 MHz) of **BtP14**


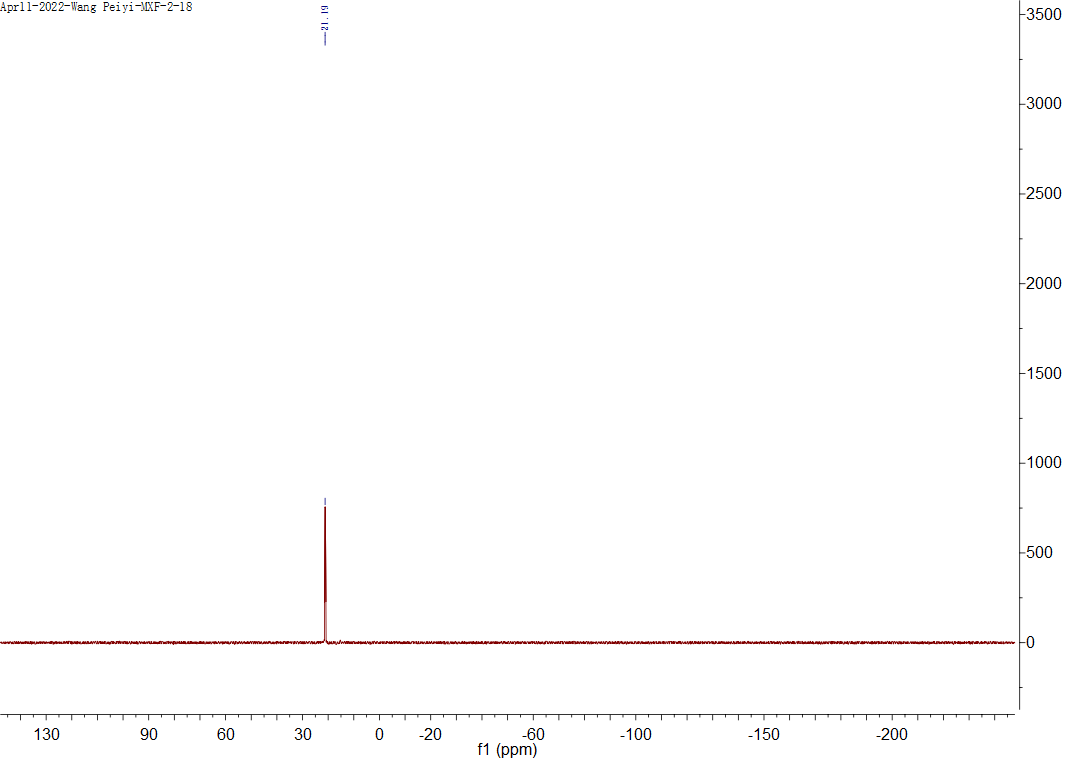


**Figure S84.** ^31^P NMR Spectrum (CDCl_3_, 162 MHz) of **BtP14**

**Figure S85.** HRMS Spectrum of Target Compound **BtP14**

**_
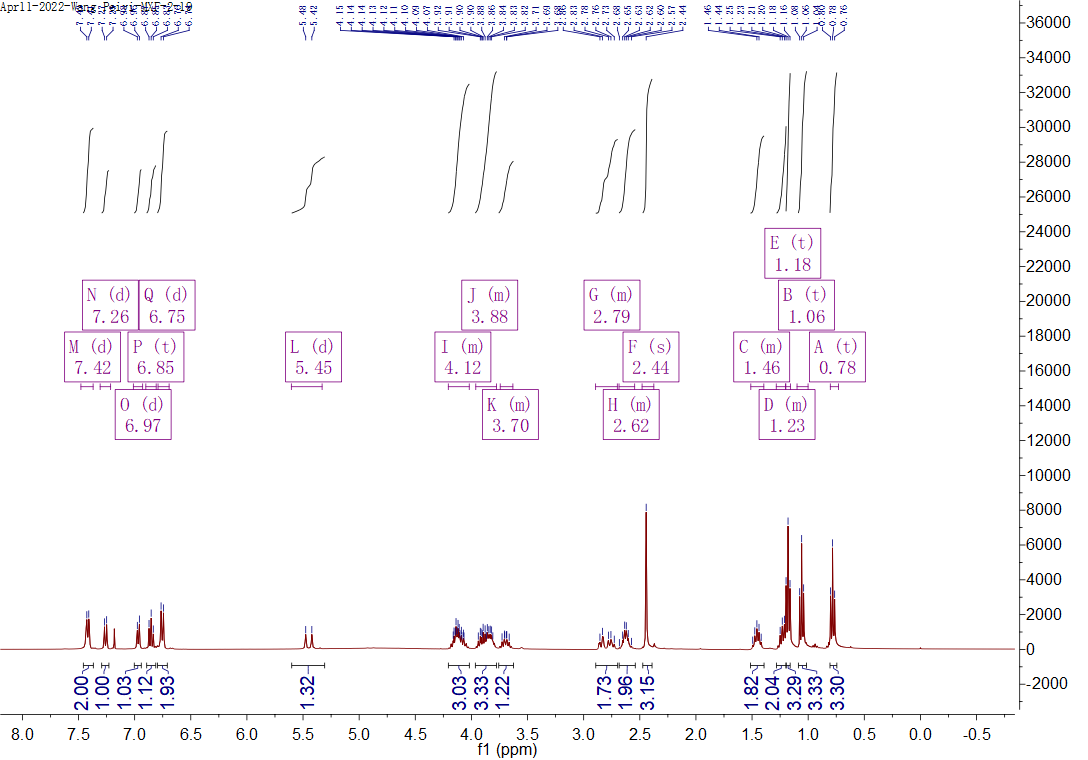
_Figure S86.** ^1^H NMR Spectrum (CDCl_3_, 400 MHz) of **BtP15**

**_
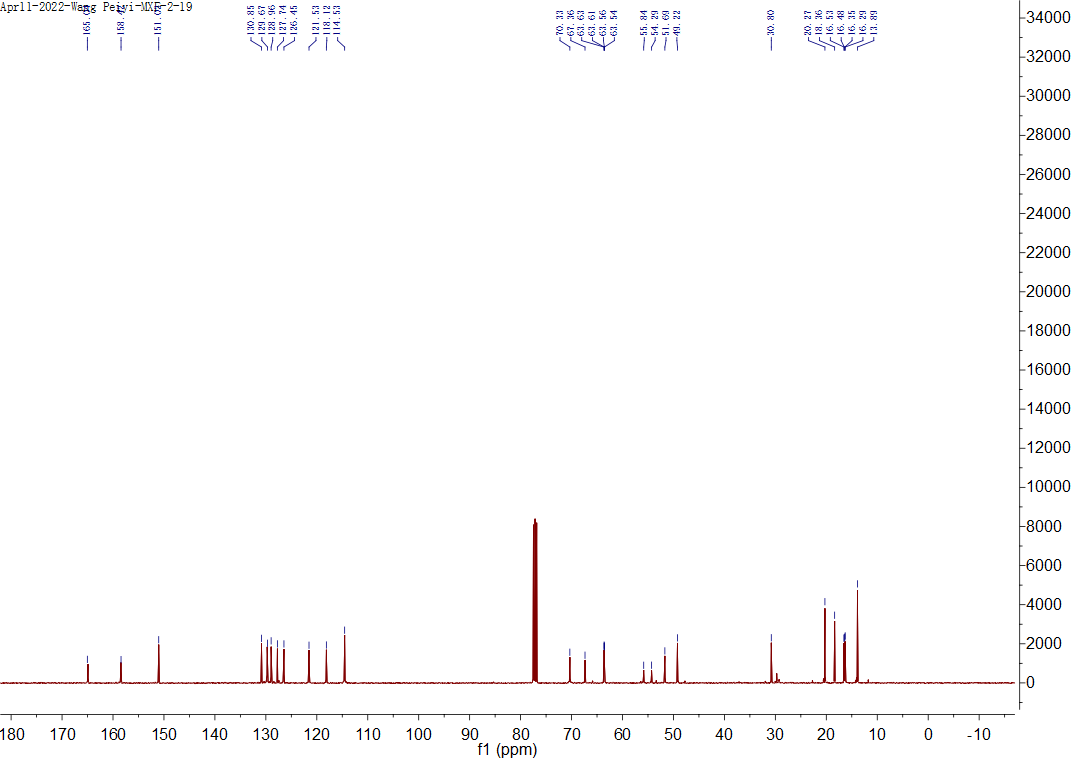
_Figure S87.** ^13^C NMR Spectrum (CDCl_3_, 101 MHz) of **BtP15**


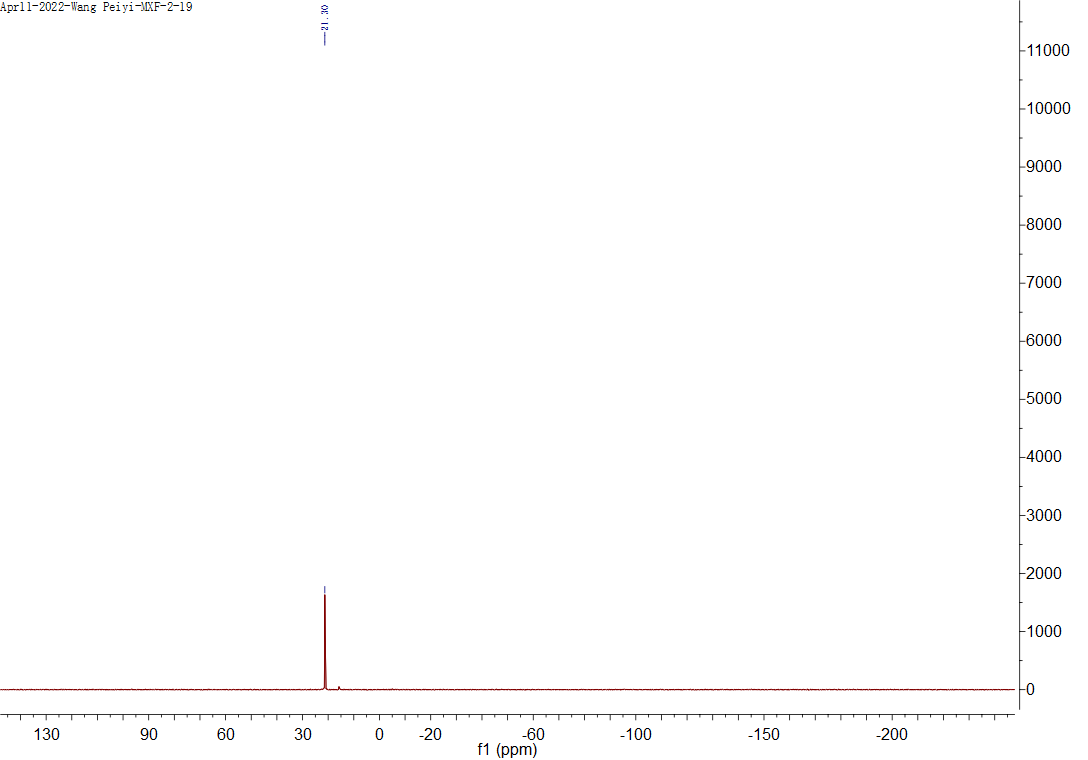


**Figure S88.** ^31^P NMR Spectrum (CDCl_3_, 162 MHz) of **BtP15**

**Figure S89.** HRMS Spectrum of Target Compound **BtP15**

**_
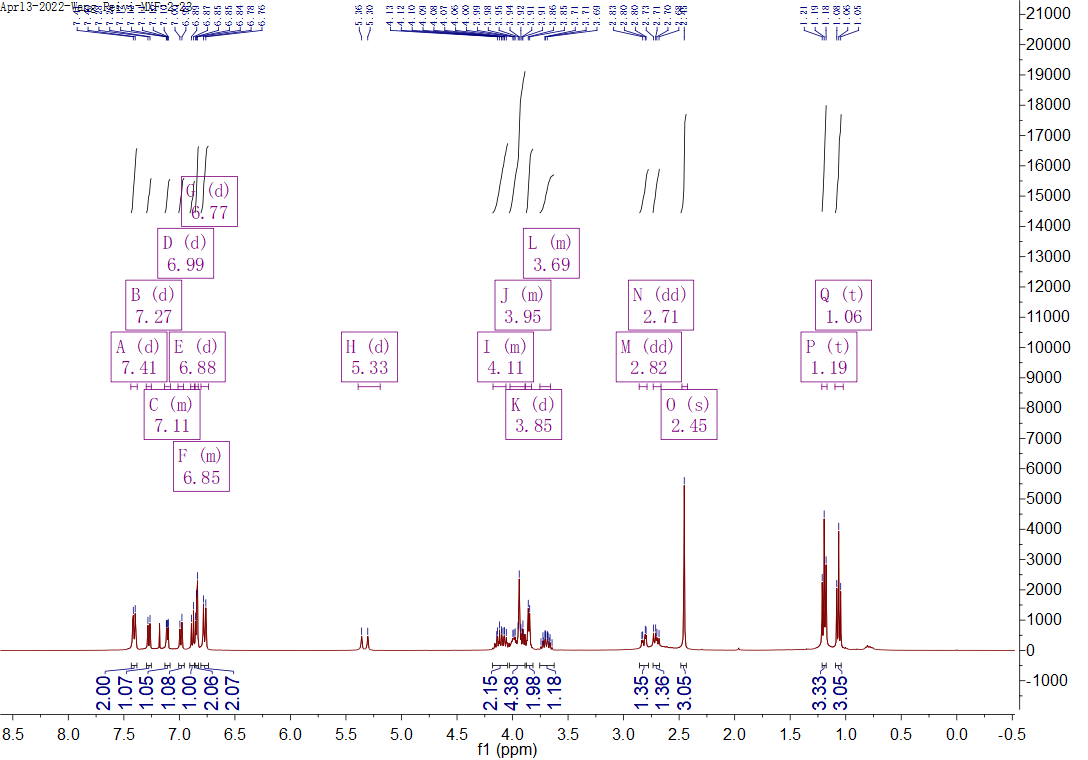
_Figure S90.** ^1^H NMR Spectrum (CDCl_3_, 400 MHz) of **BtP16**

**_
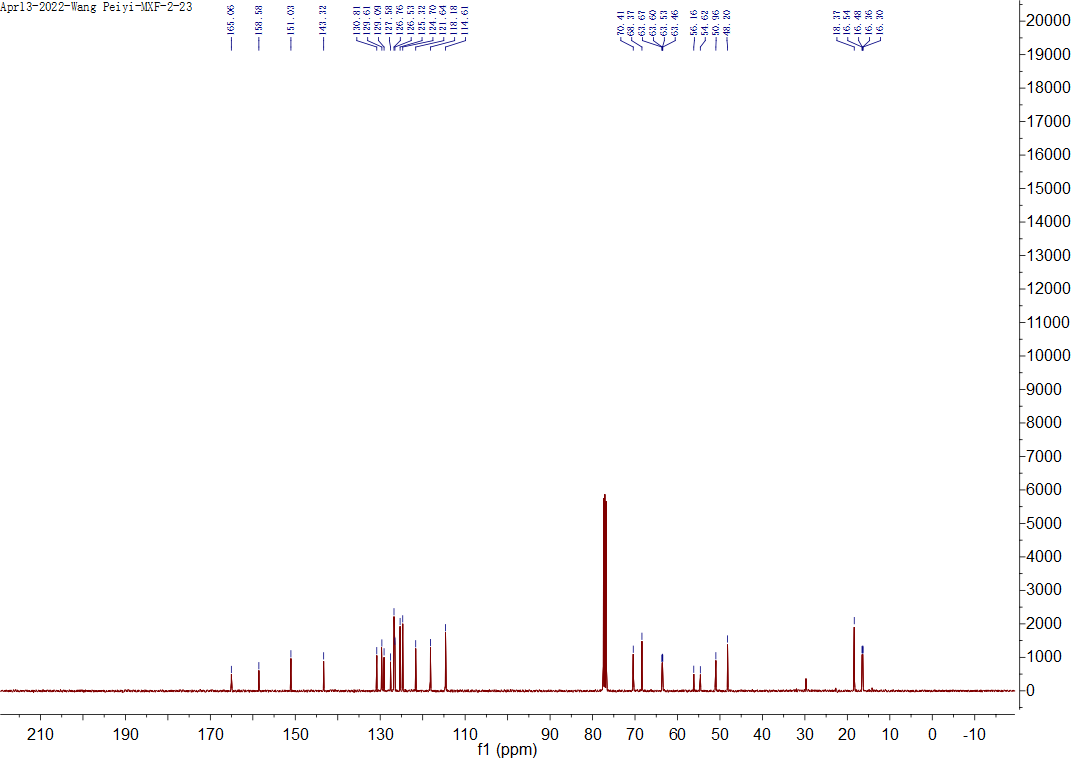
_Figure S91.** ^13^C NMR Spectrum (CDCl_3_, 101 MHz) of **BtP16**


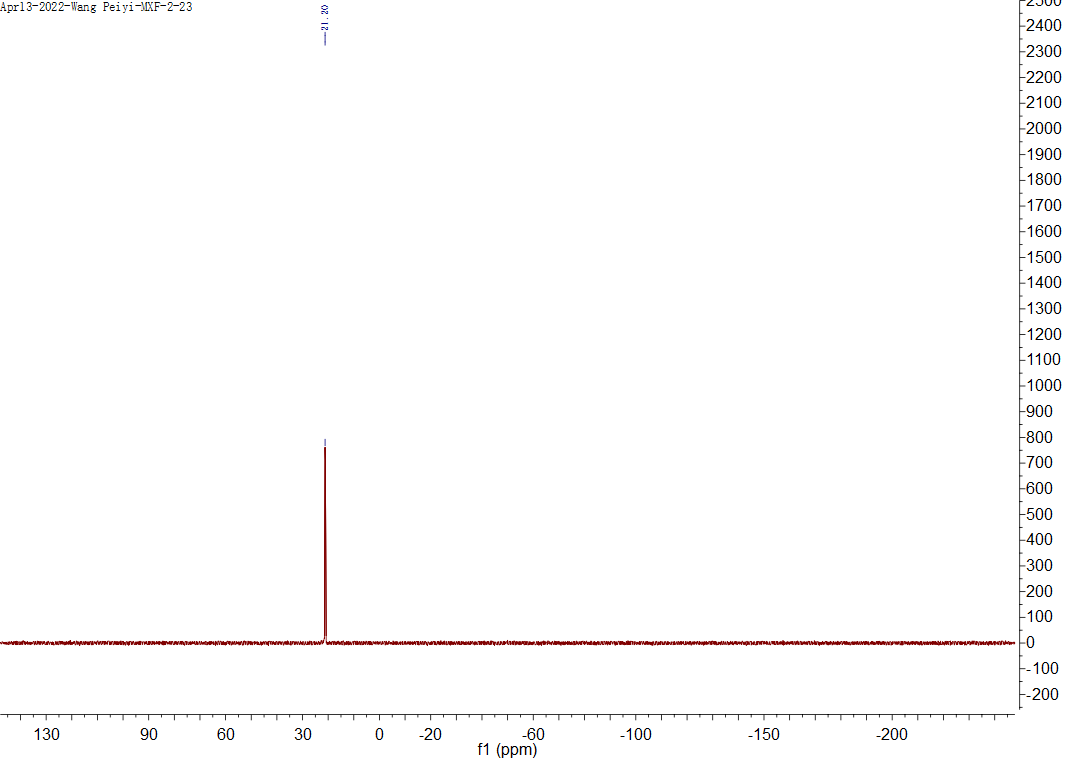


**Figure S92.** ^31^P NMR Spectrum (CDCl_3_, 162 MHz) of **BtP16**

**Figure S93.** HRMS Spectrum of Target Compound **BtP16**


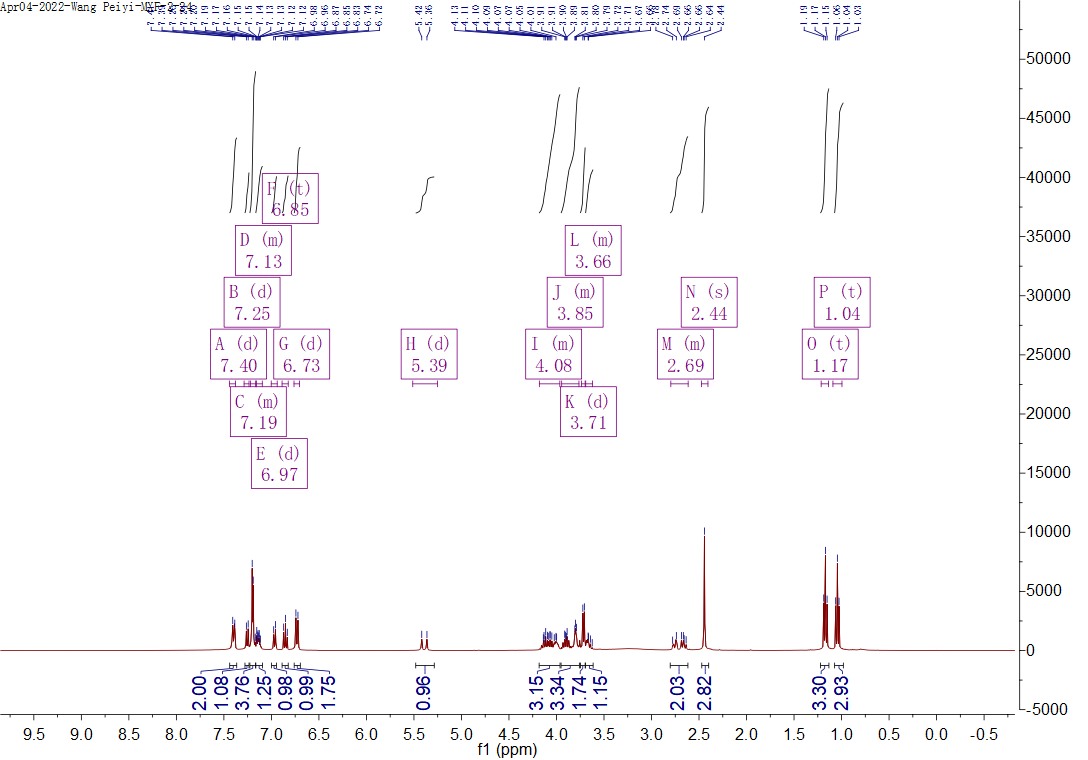
**Figure S94.** ^1^H NMR Spectrum (CDCl_3_, 400 MHz) of **BtP17**

**_
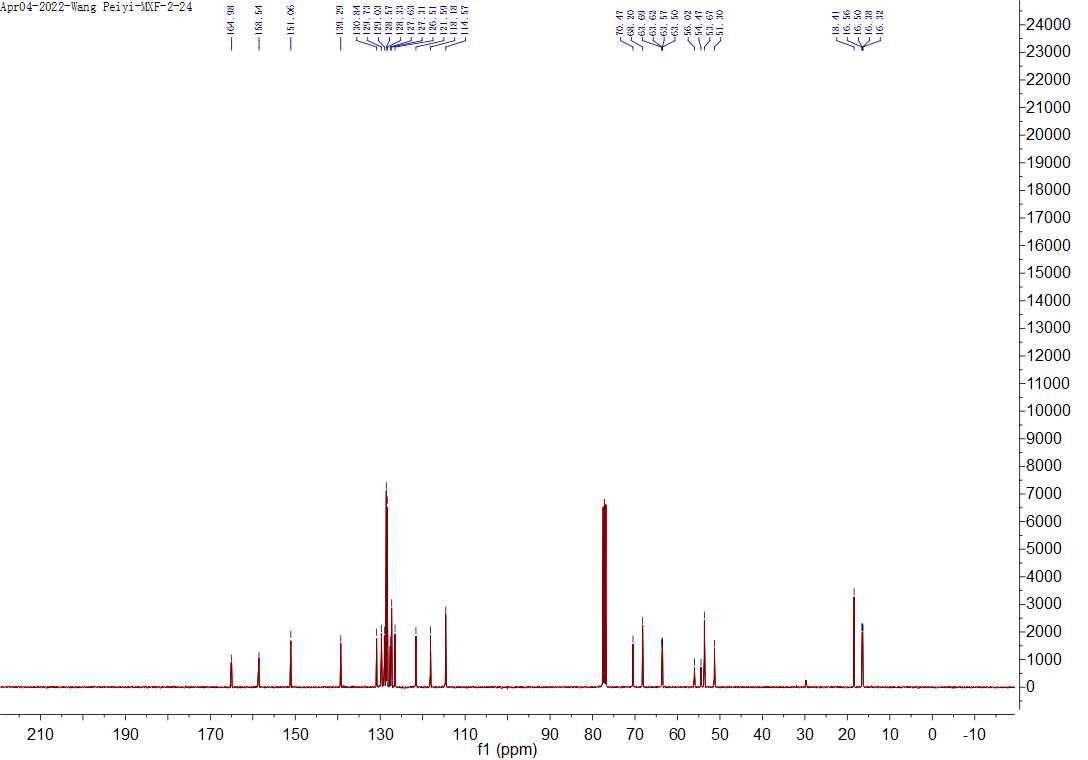
_Figure S95.** ^13^C NMR Spectrum (CDCl_3_, 101 MHz) of **BtP17**


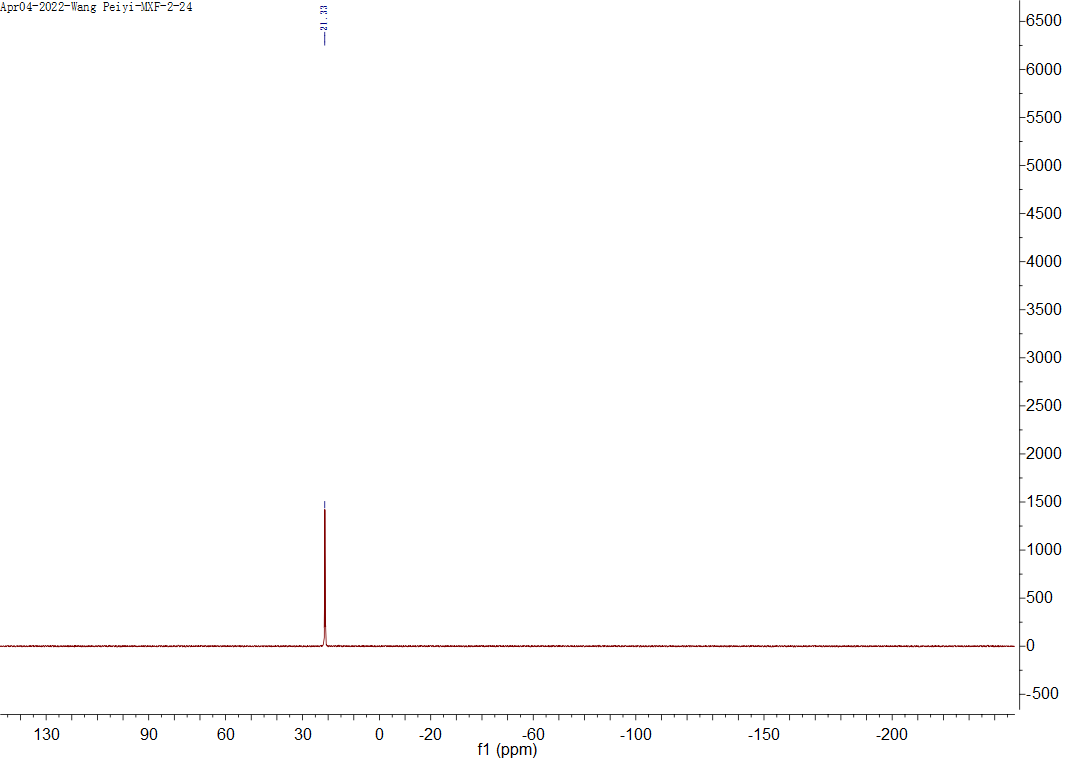


**Figure S96.** ^31^P NMR Spectrum (CDCl_3_, 162 MHz) of **BtP17**

**Figure S97.** HRMS Spectrum of Target Compound **BtP17**


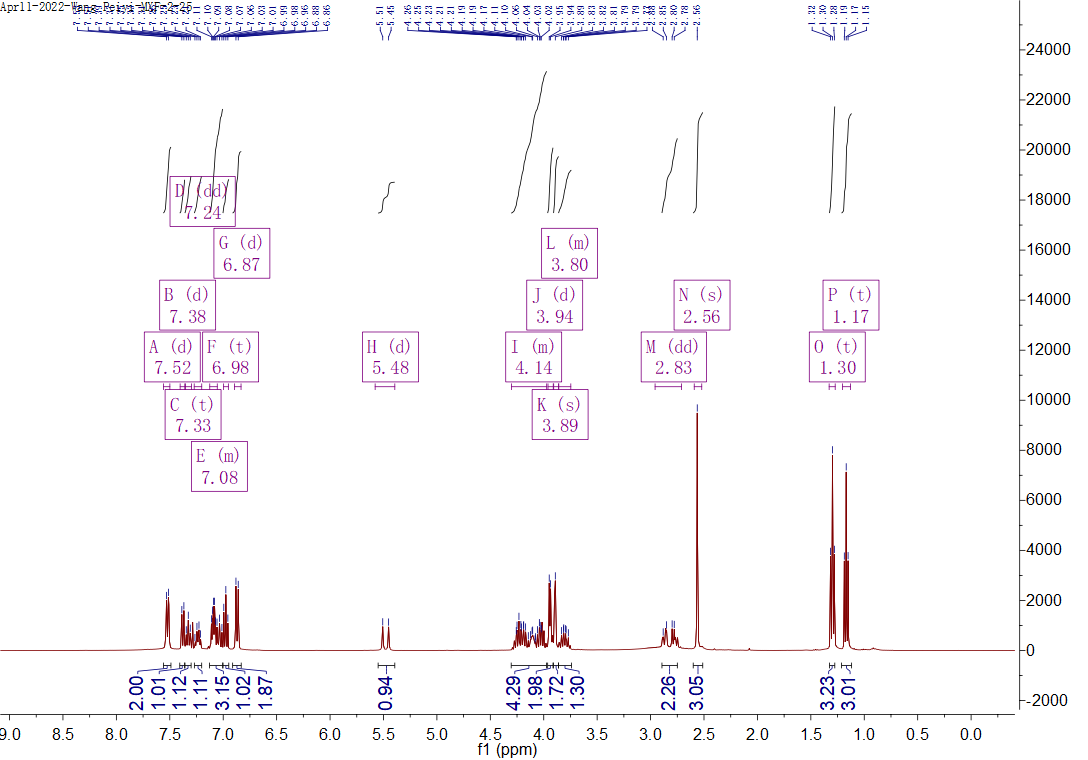
**Figure S98.** ^1^H NMR Spectrum (CDCl_3_, 400 MHz) of **BtP18**

**_
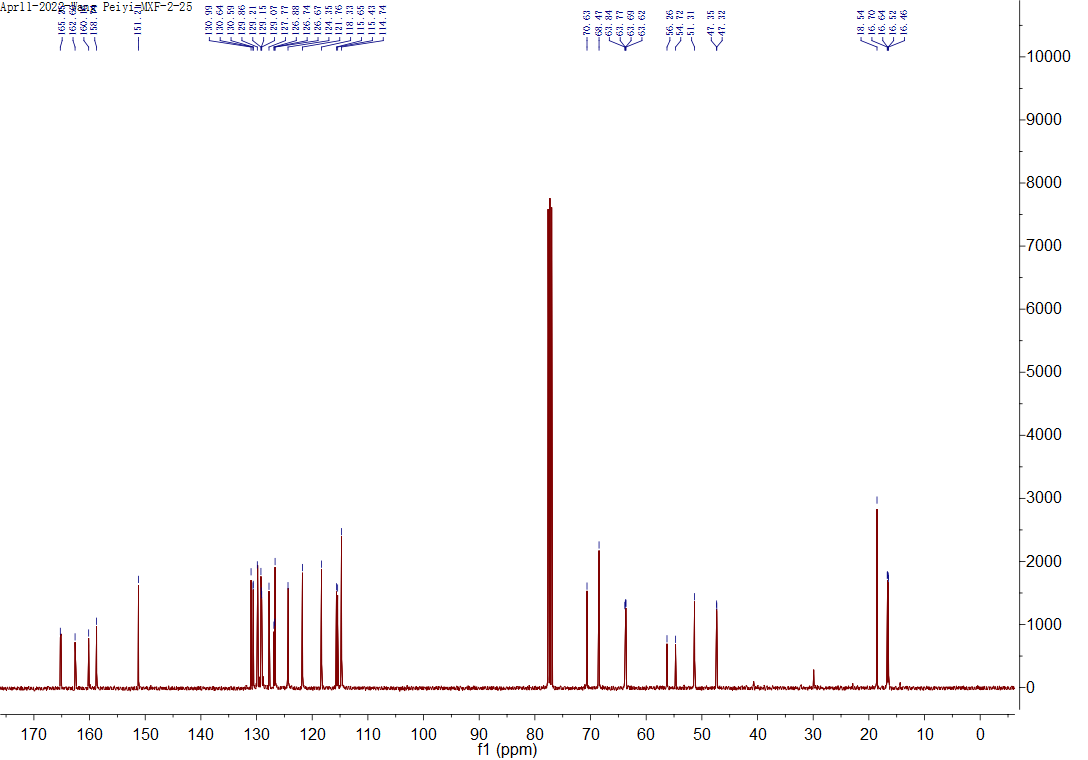
_Figure S99.** ^13^C NMR Spectrum (CDCl_3_, 101 MHz) of **BtP18**


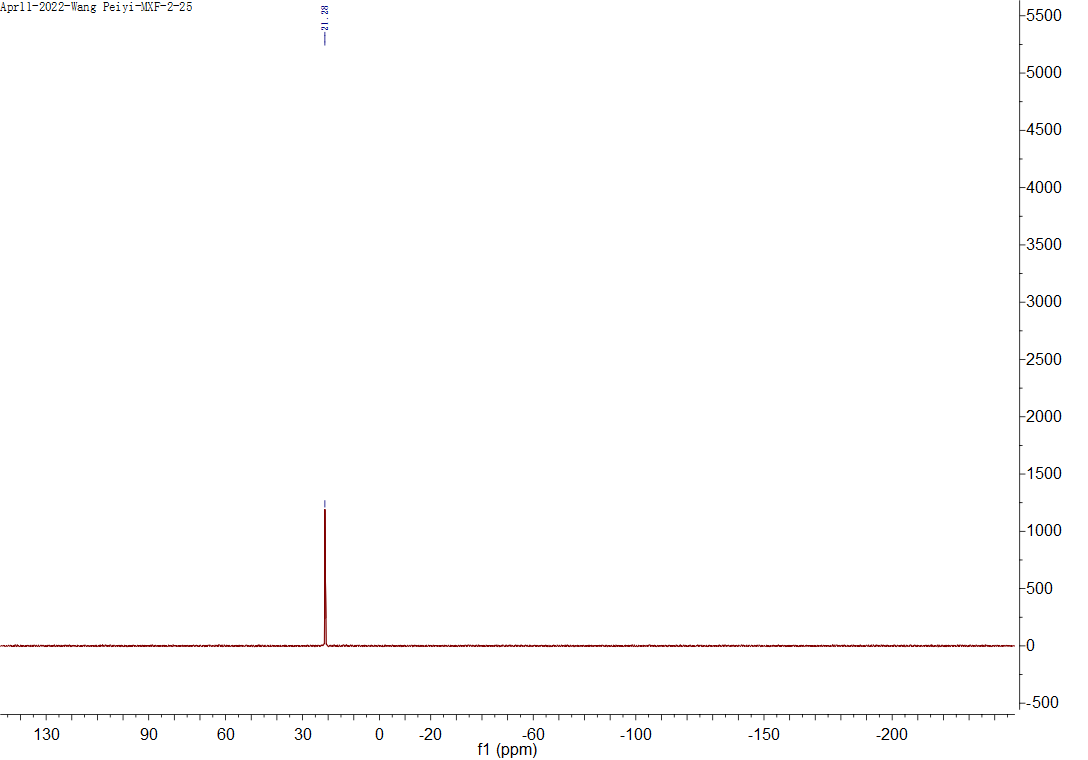


**Figure S100.** ^31^P NMR Spectrum (CDCl_3_, 162 MHz) of **BtP18**

**
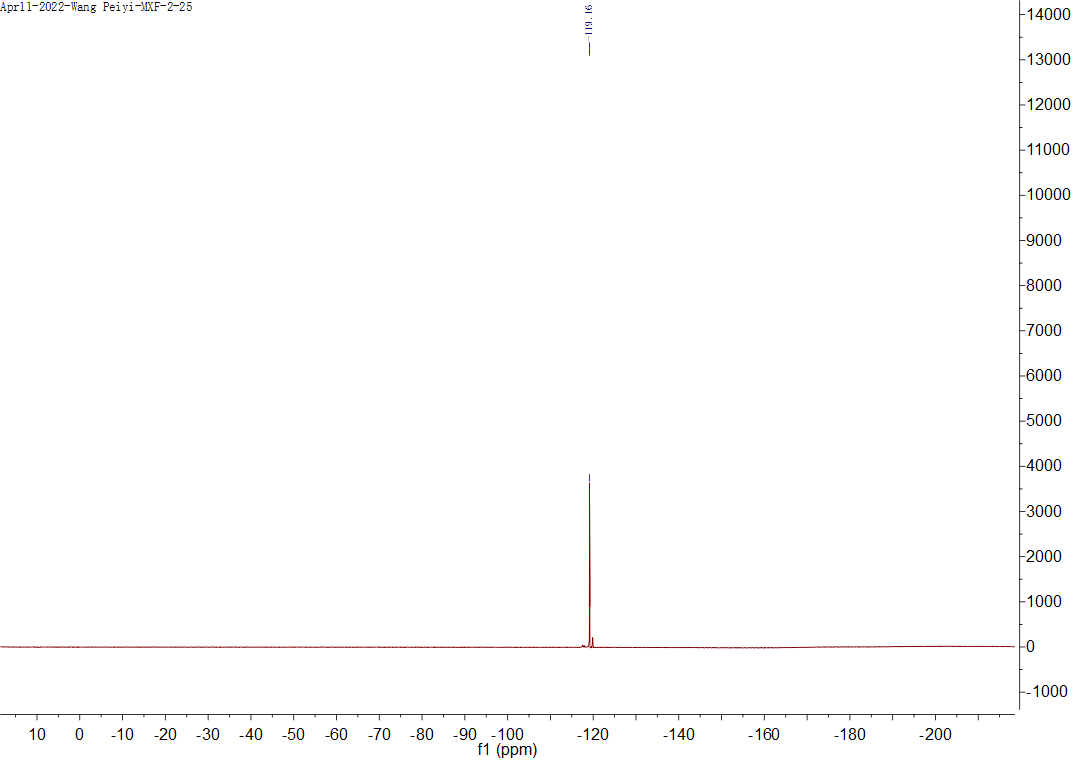
**

**Figure S101.** ^19^F NMR Spectrum (CDCl_3_, 376 MHz) of **BtP18**

**Figure S102.** HRMS Spectrum of Target Compound **BtP18**


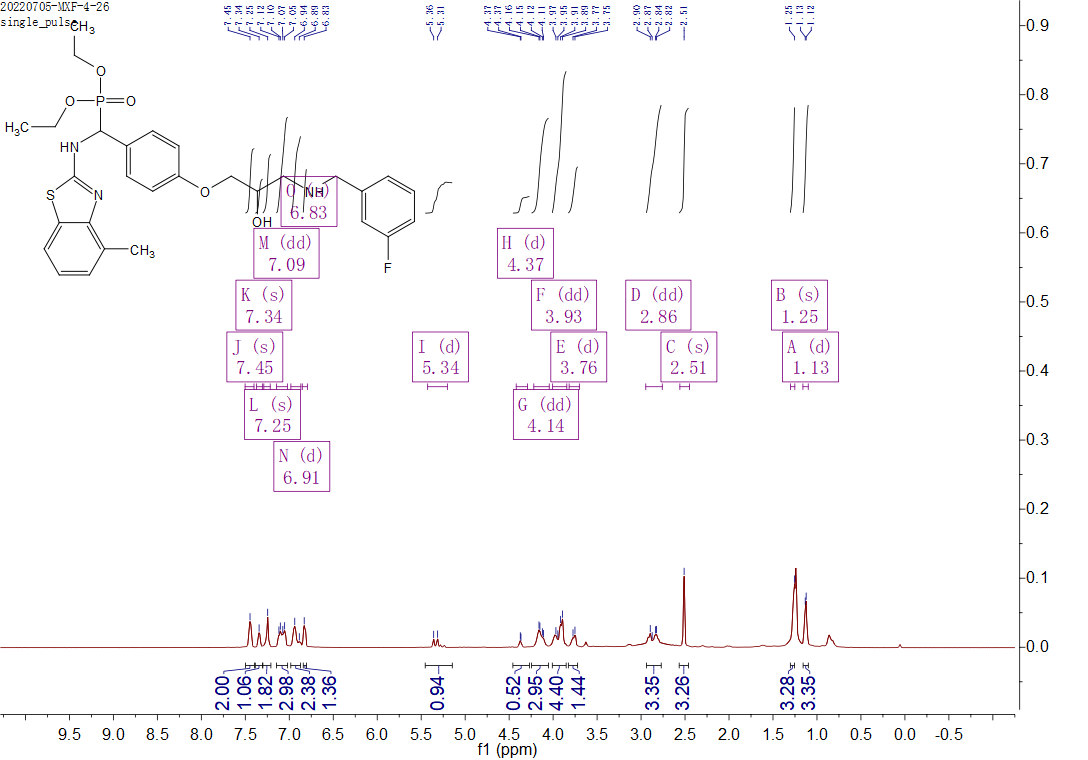
**Figure S103.** ^1^H NMR Spectrum (CDCl_3_, 500 MHz) of **BtP19**

**_
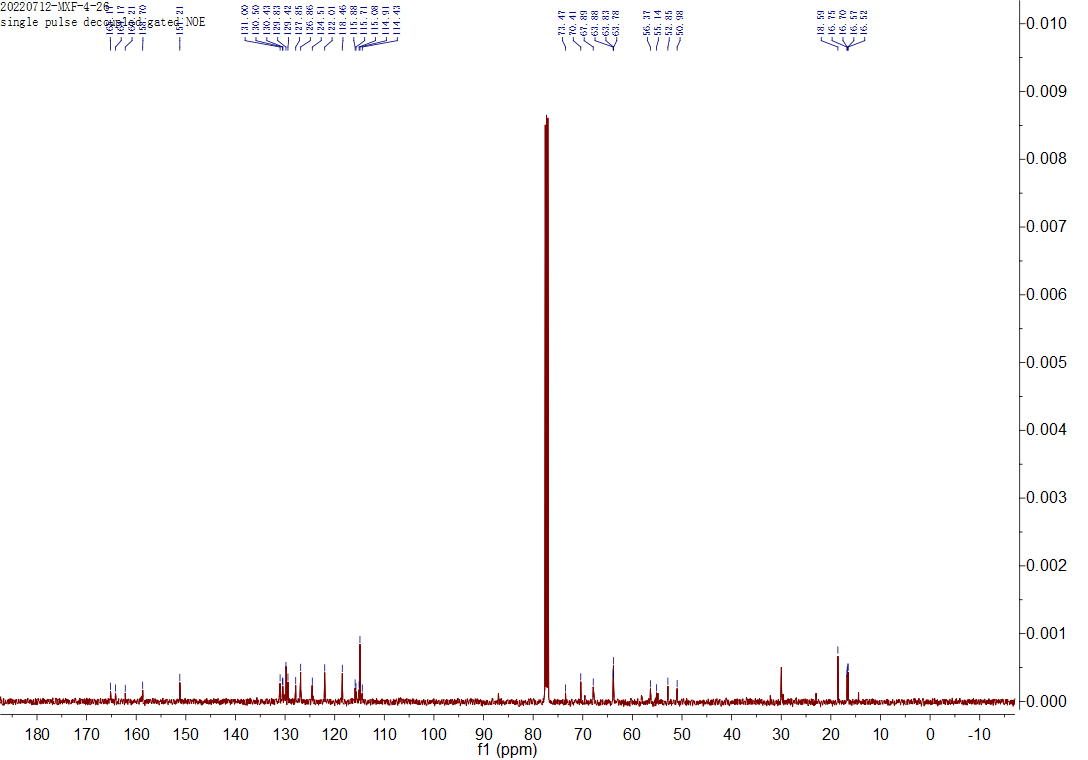
_Figure S104.** ^13^C NMR Spectrum (CDCl_3_, 126 MHz) of **BtP19**


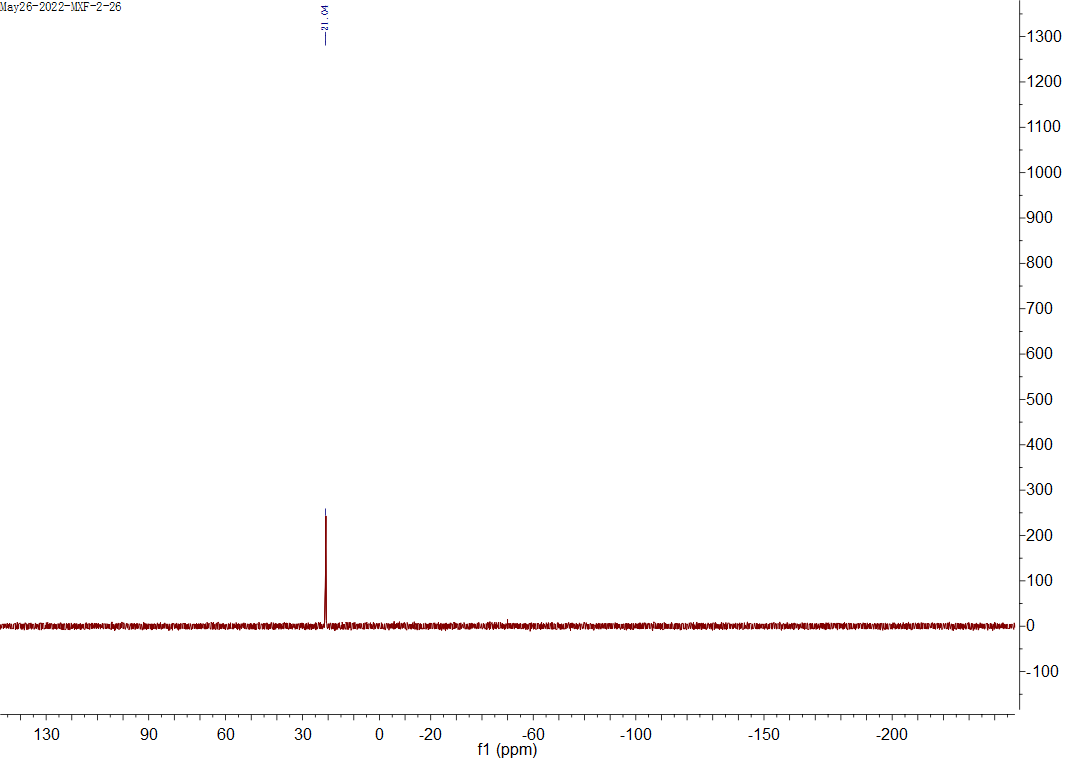


**Figure S105.** ^31^P NMR Spectrum (CDCl_3_, 162 MHz) of **BtP19**

**
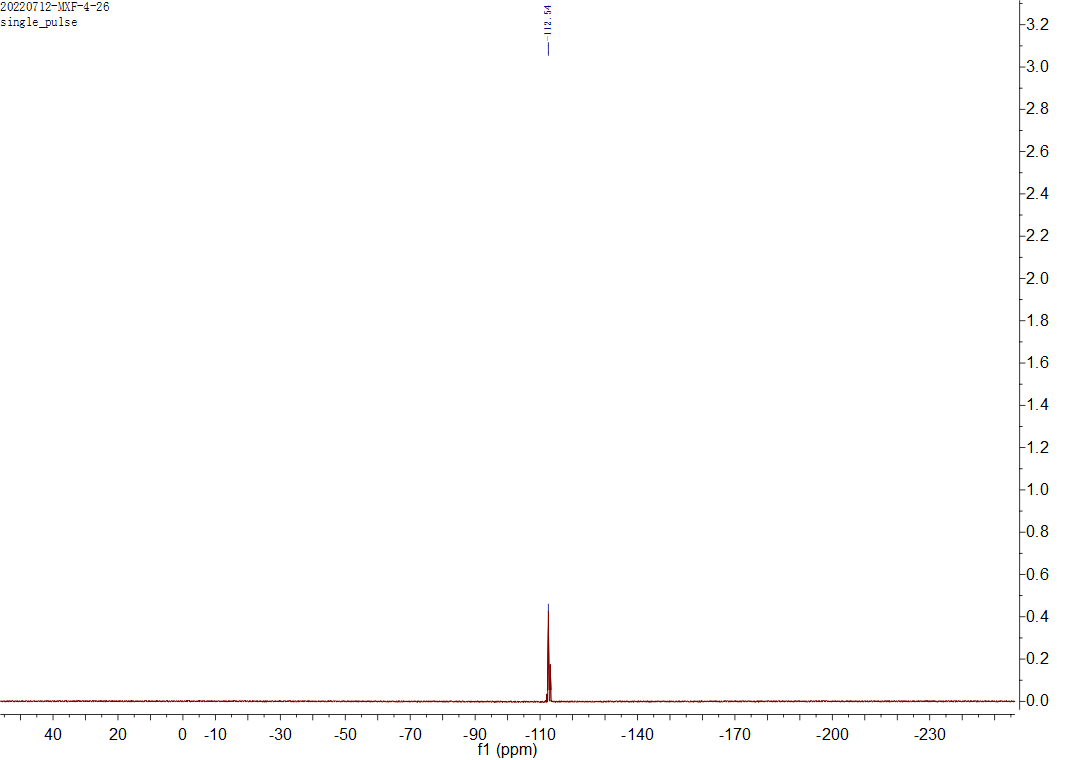
**

**Figure S106.** ^19^F NMR Spectrum (CDCl_3_, 376 MHz) of **BtP19**

**Figure S107.** HRMS Spectrum of Target Compound **BtP19**


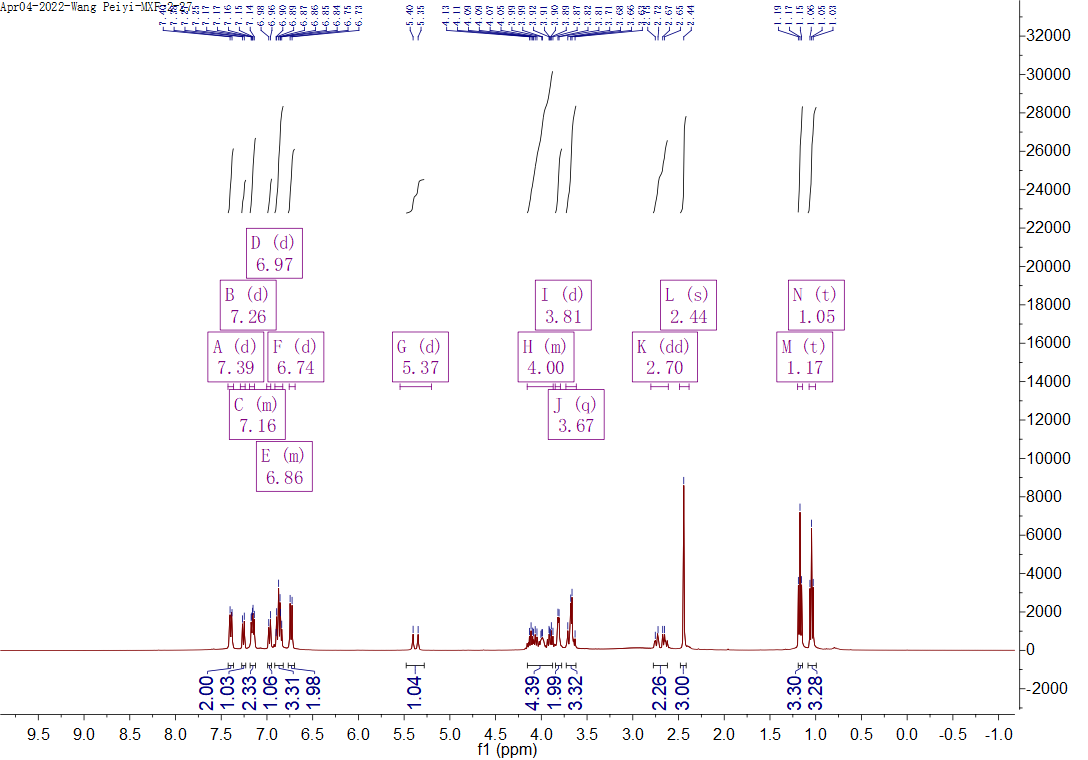
**Figure S108.** ^1^H NMR Spectrum (CDCl_3_, 400 MHz) of **BtP20**

**_
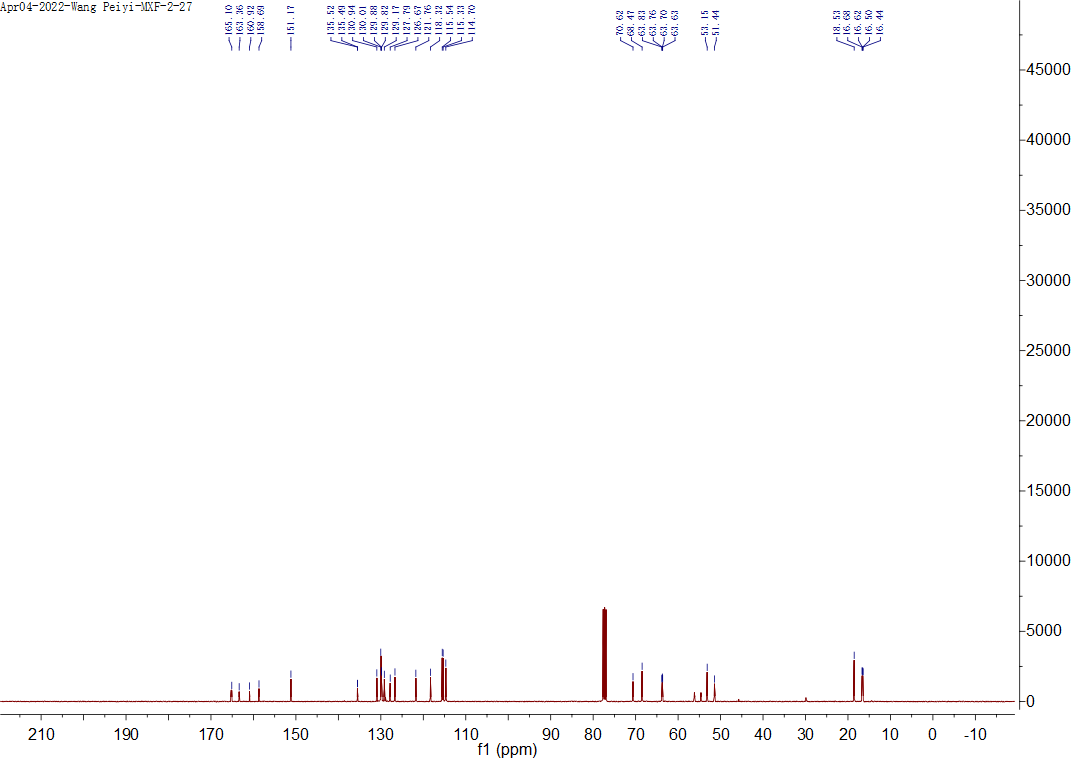
_Figure S109.** ^13^C NMR Spectrum (CDCl_3_, 101 MHz) of **BtP20**


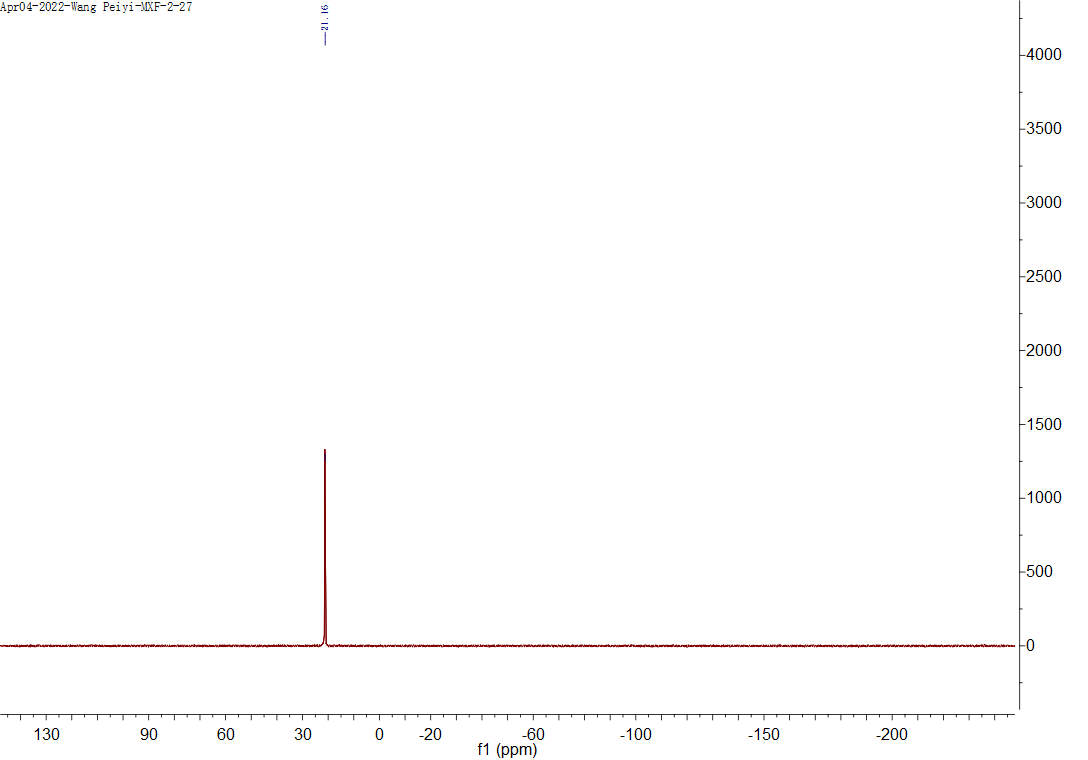


**Figure S110.** ^31^P NMR Spectrum (CDCl_3_, 162 MHz) of **BtP20**

**
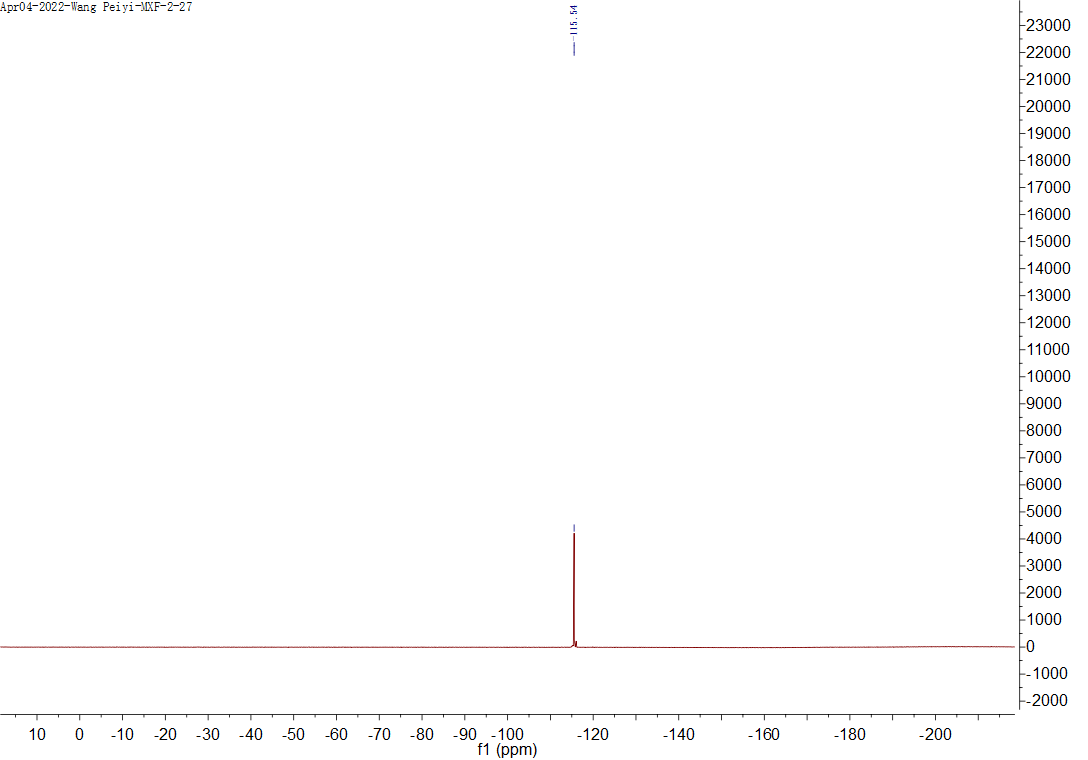
**

**Figure S111.** ^19^F NMR Spectrum (CDCl_3_, 376 MHz) of **BtP20**

**Figure S112.** HRMS Spectrum of Target Compound **BtP20**


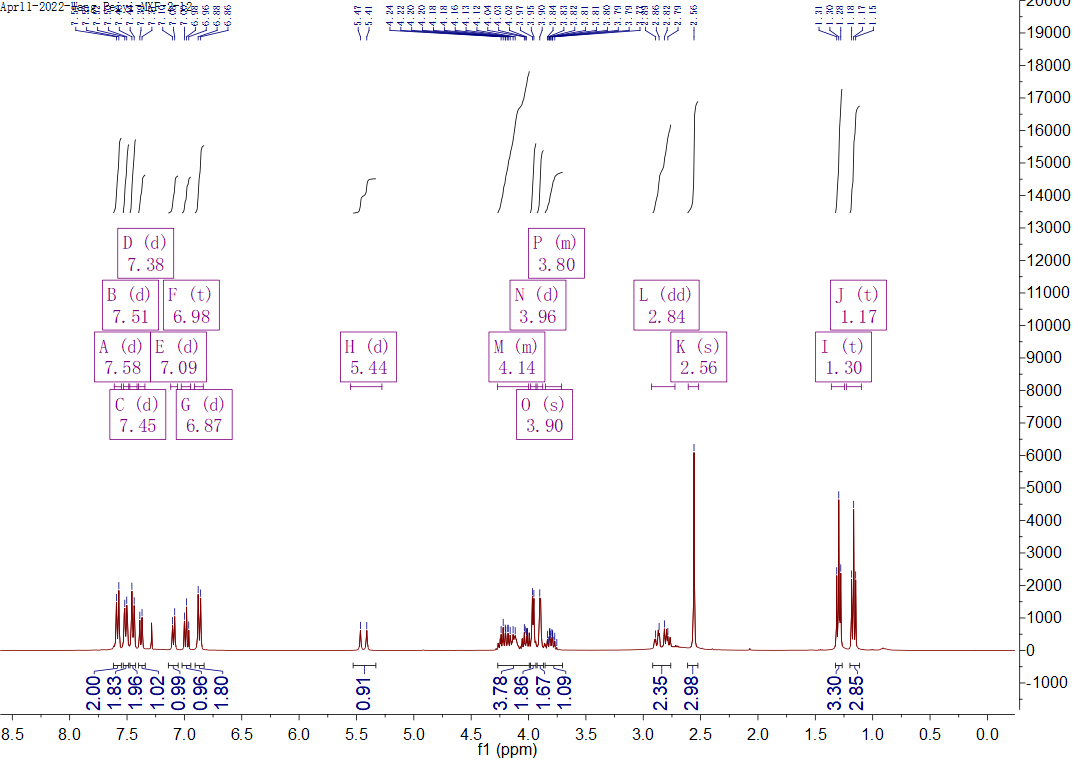
**Figure S113.** ^1^H NMR Spectrum (CDCl_3_, 400 MHz) of **BtP21**

**_
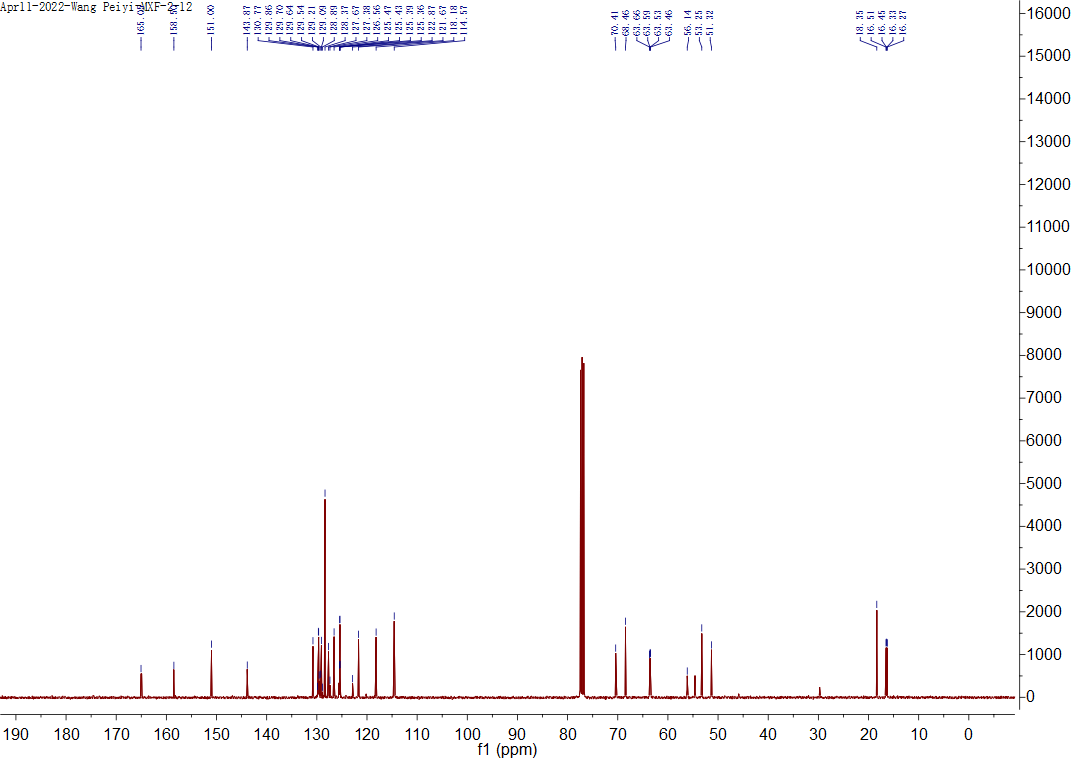
_**

**Figure S114.** ^13^C NMR Spectrum (CDCl_3_, 101 MHz) of **BtP21**


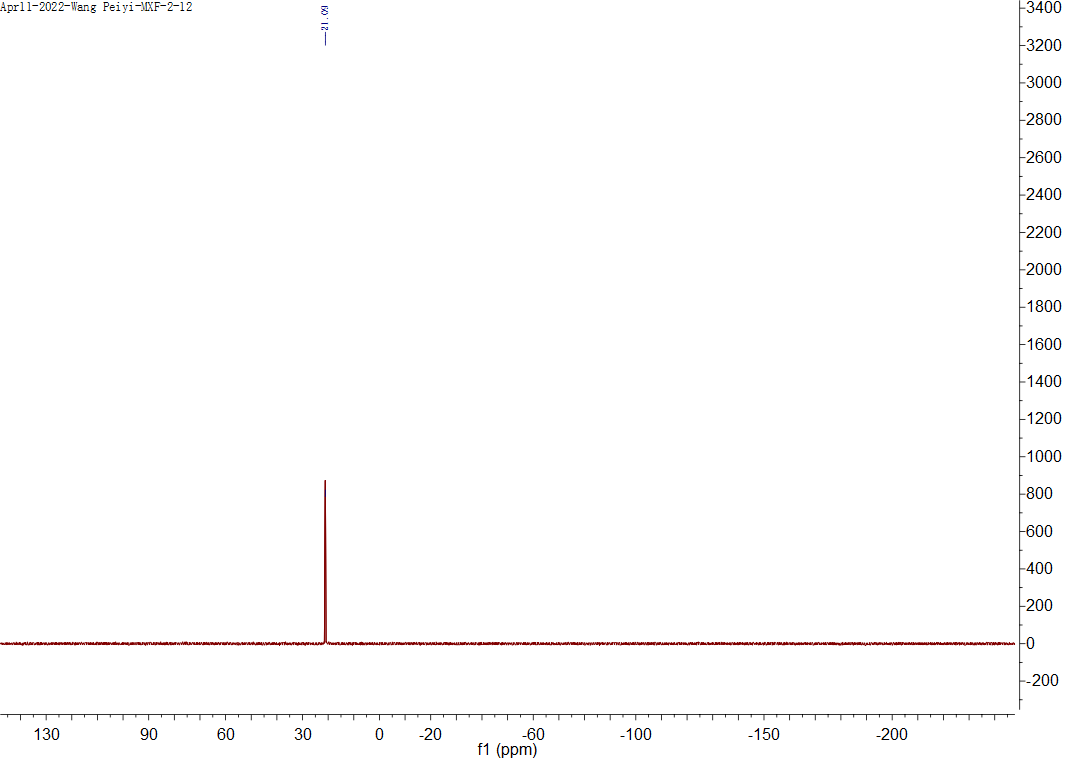


**Figure S115.** ^31^P NMR Spectrum (CDCl_3_, 162 MHz) of **BtP21**

**
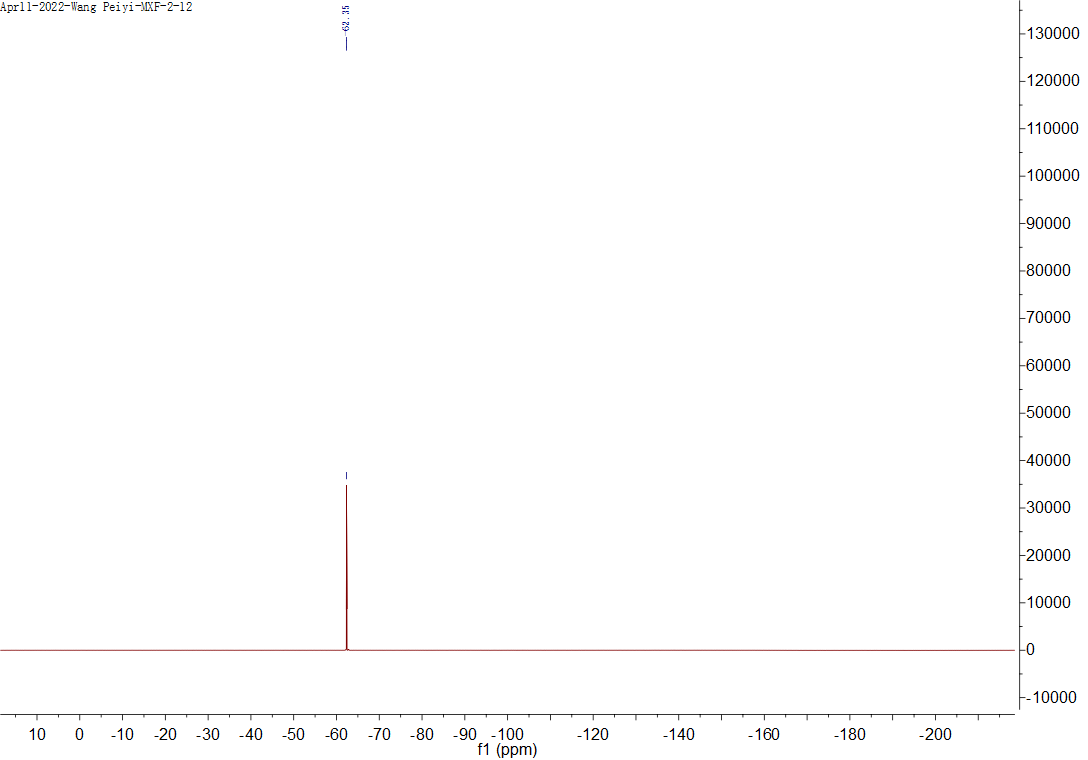
**

**Figure S116.** ^19^F NMR Spectrum (CDCl_3_, 376 MHz) of **BtP21**

**Figure S117.** HRMS Spectrum of Target Compound **BtP21**


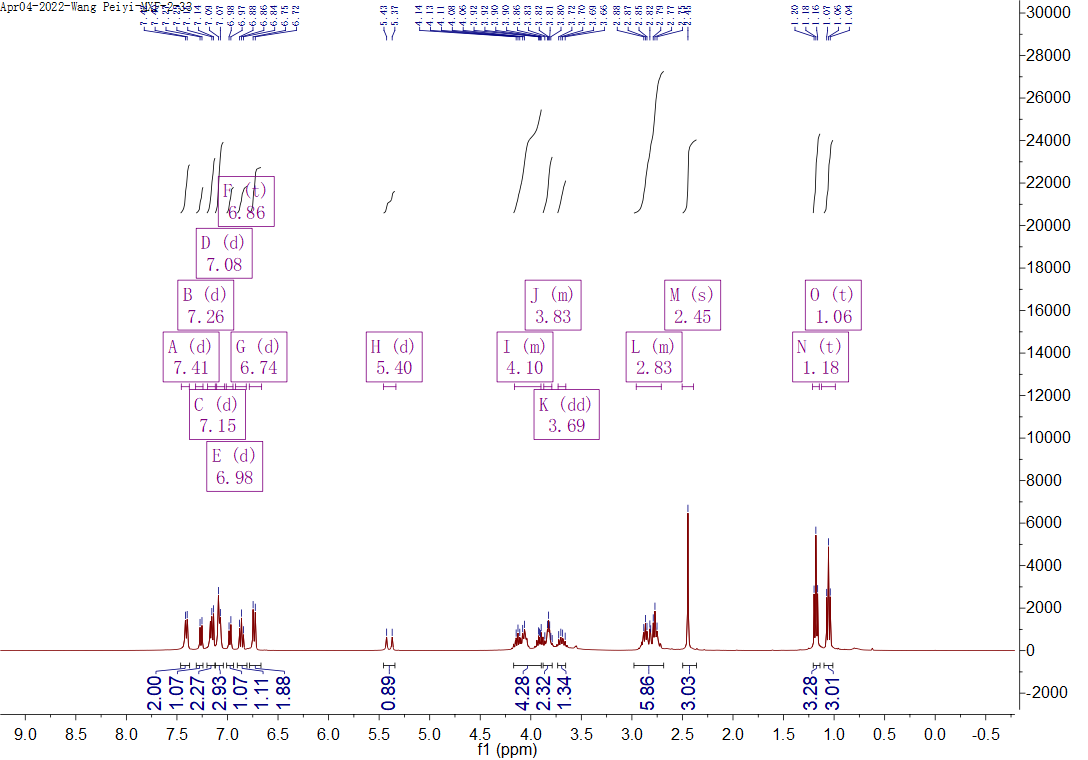
**Figure S118.** ^1^H NMR Spectrum (CDCl_3_, 400 MHz) of **BtP22**

**_
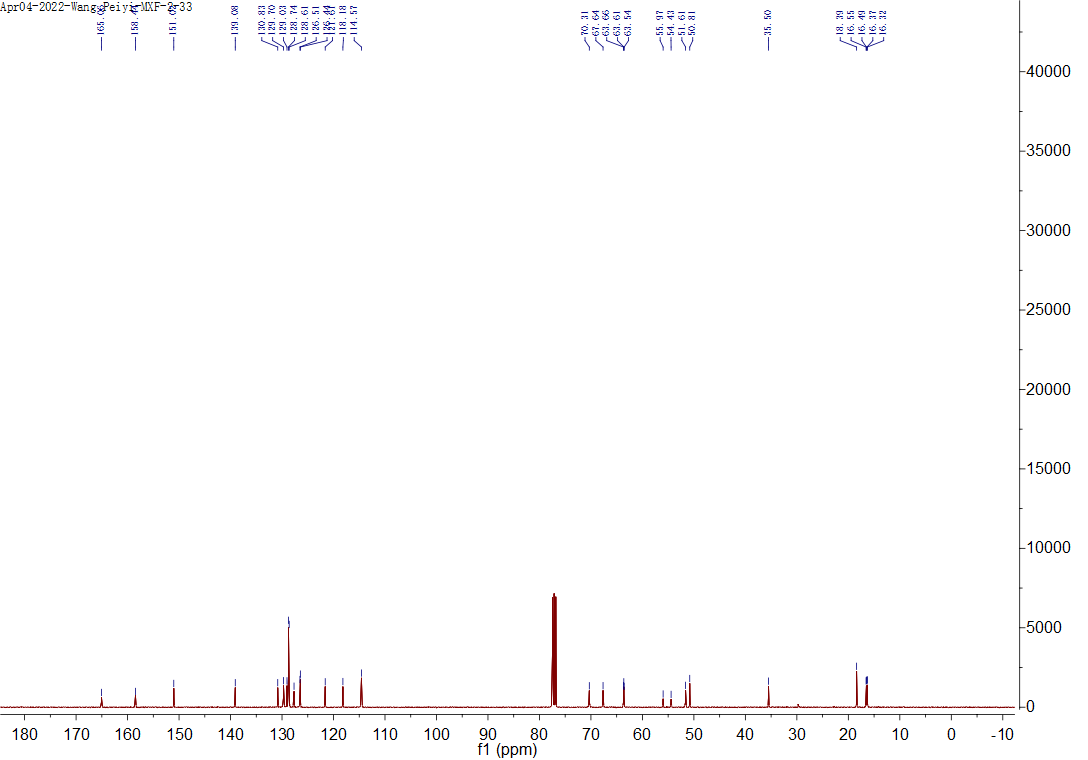
_Figure S119.** ^13^C NMR Spectrum (CDCl_3_, 101 MHz) of **BtP22**


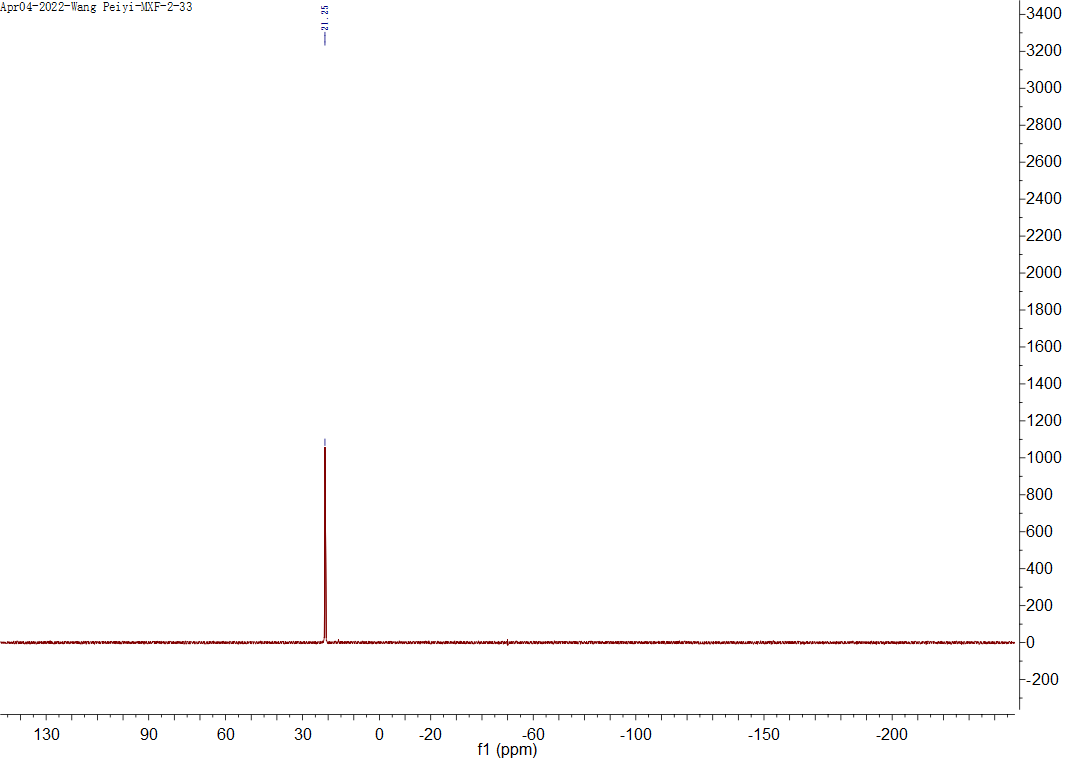


**Figure S120.** ^31^P NMR Spectrum (CDCl_3_, 162 MHz) of **BtP22**

**Figure S121.** HRMS Spectrum of Target Compound **BtP22**


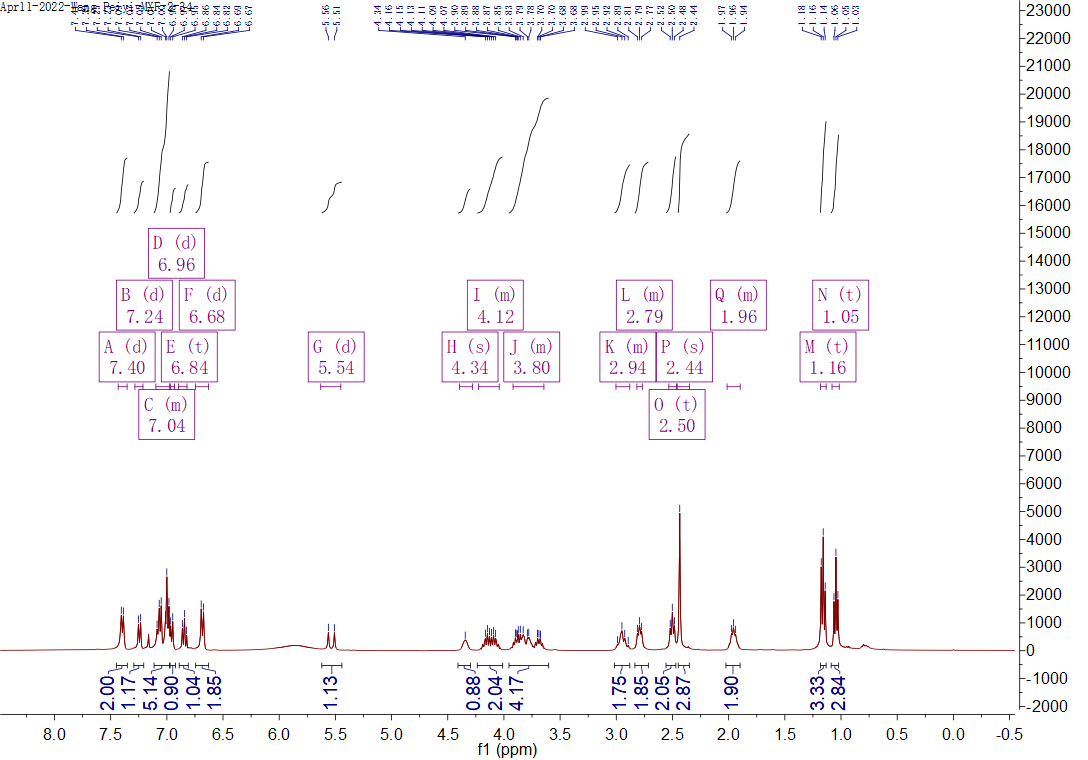
**Figure S122.** ^1^H NMR Spectrum (CDCl_3_, 400 MHz) of **BtP23**

**_
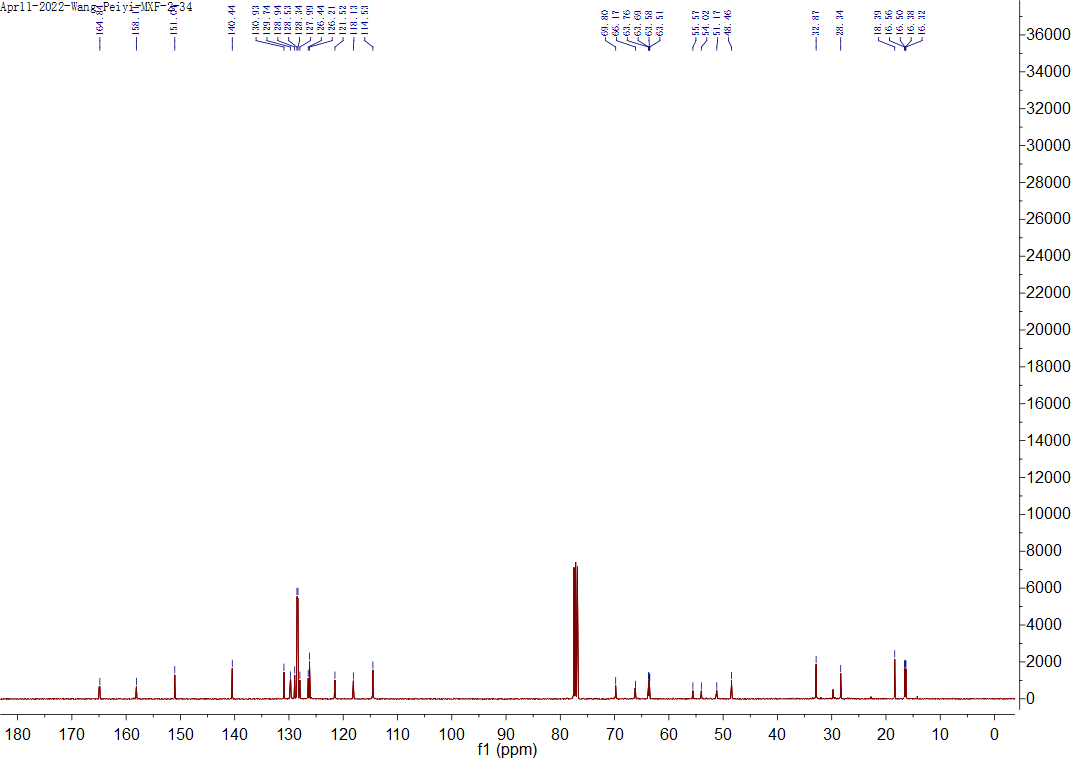
_Figure S123.** ^13^C NMR Spectrum (CDCl_3_, 101 MHz) of **BtP23**


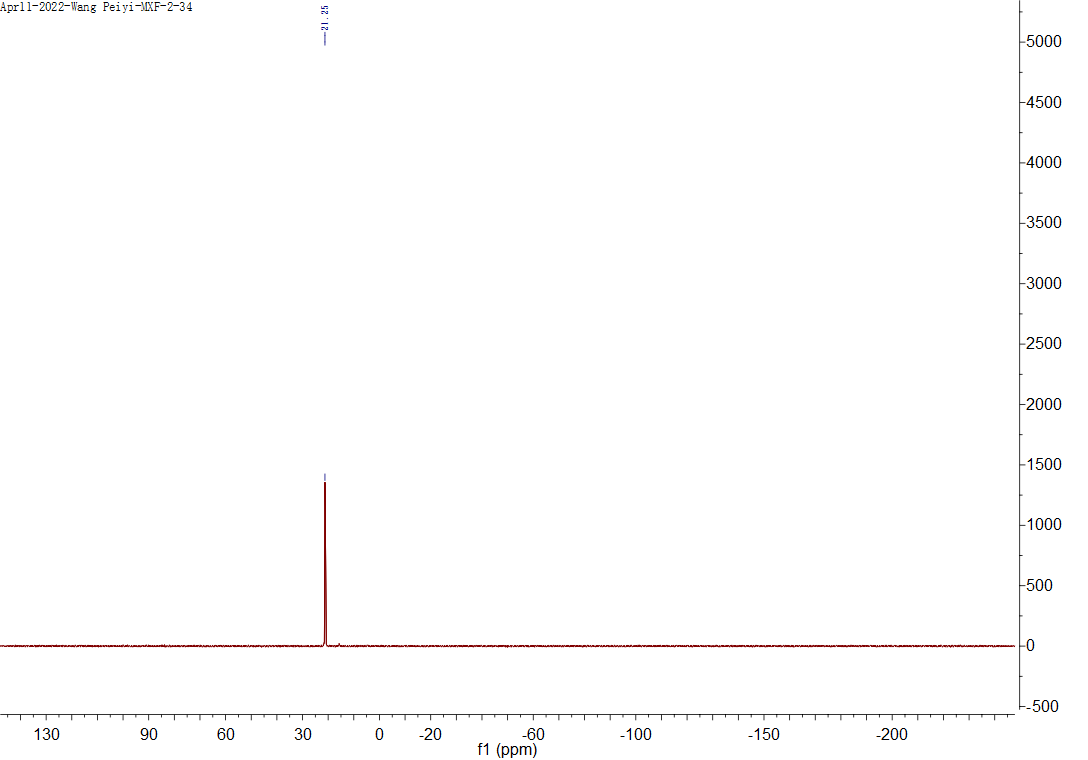


**Figure S124.** ^31^P NMR Spectrum (CDCl_3_, 162 MHz) of **BtP23**

**Figure S125.** HRMS Spectrum of Target Compound **BtP23**


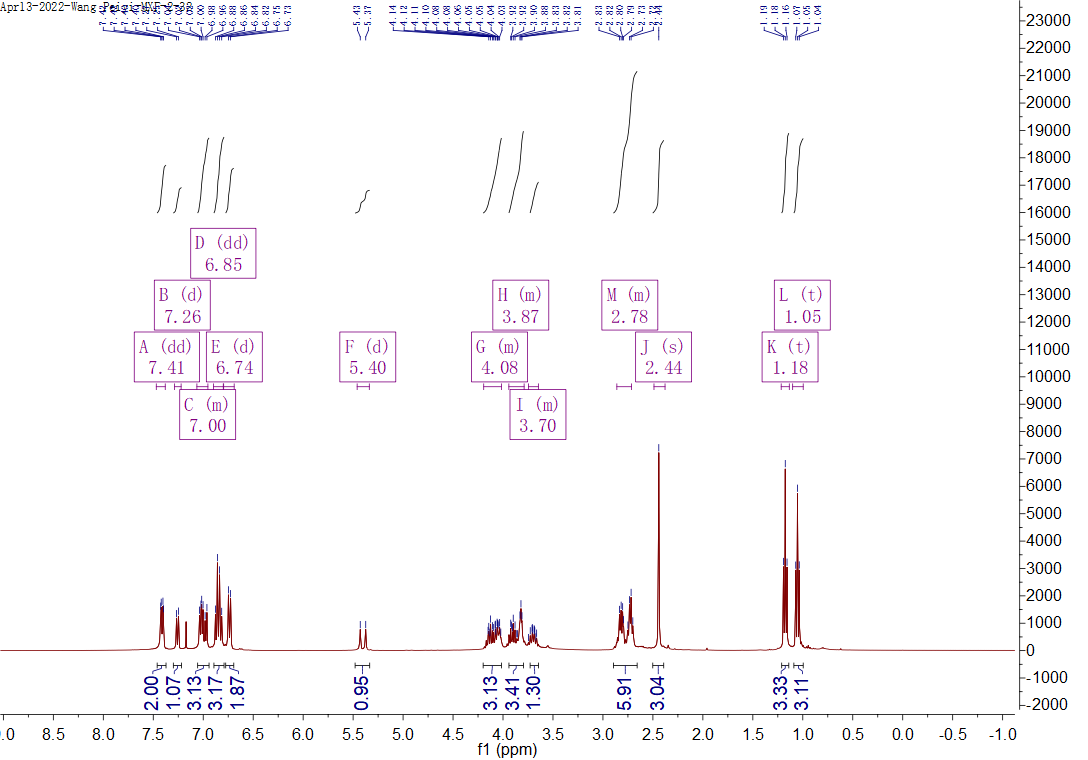
**Figure S126.** ^1^H NMR Spectrum (CDCl_3_, 400 MHz) of **BtP24**

**_
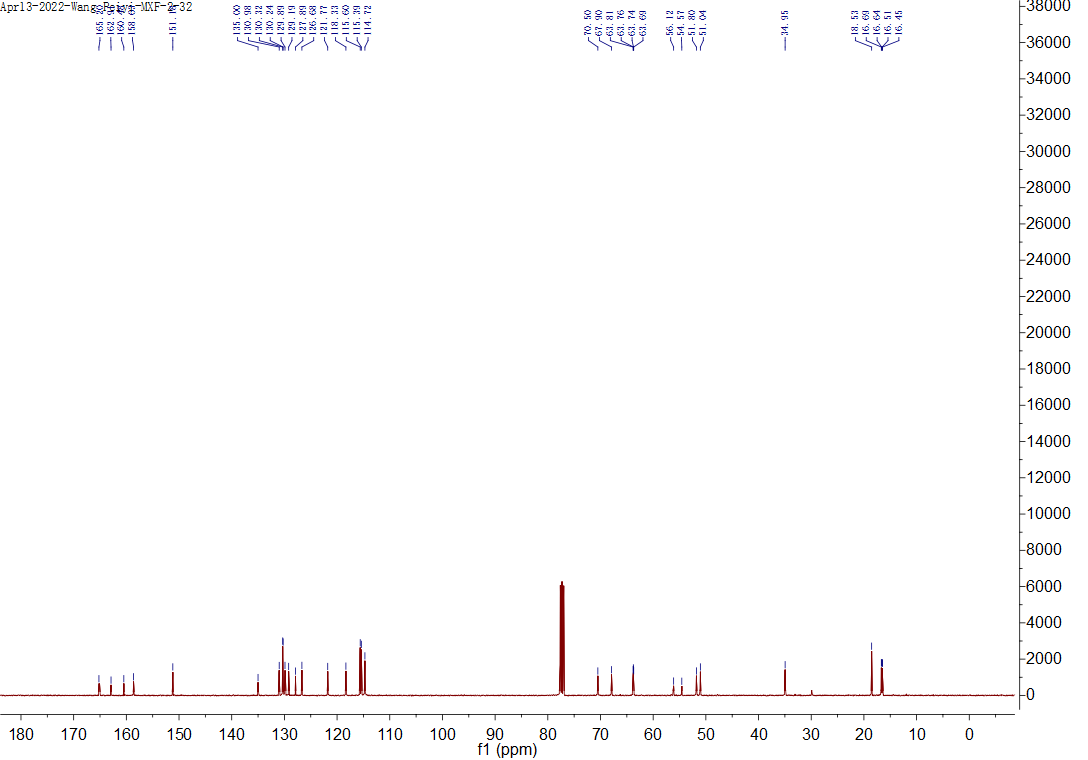
_Figure S127.** ^13^C NMR Spectrum (CDCl_3_, 101 MHz) of **BtP24**


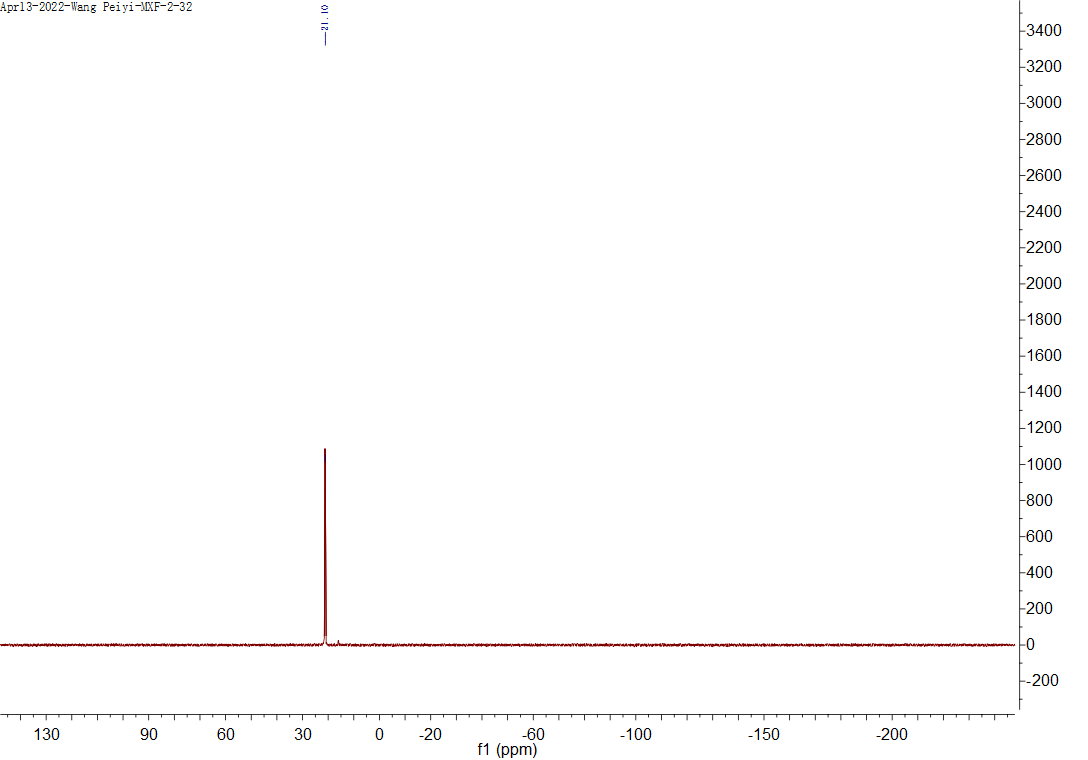


**Figure S128.** ^31^P NMR Spectrum (CDCl_3_, 162 MHz) of **BtP24**

**
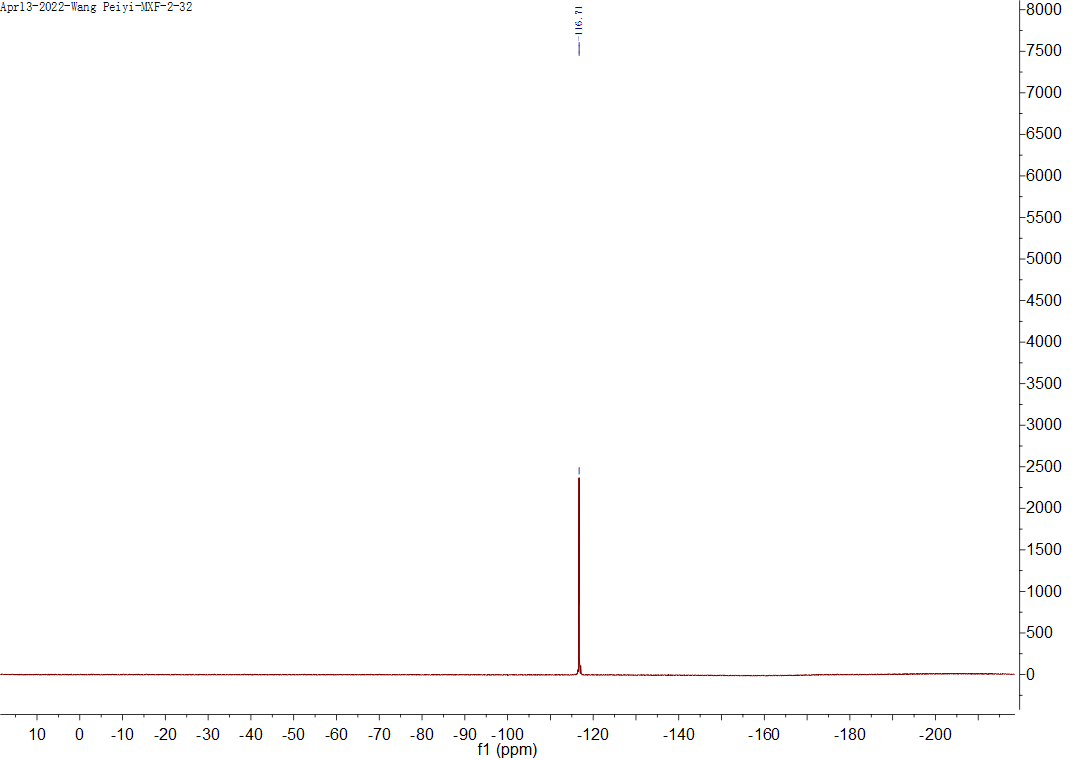
**

**Figure S129.** ^19^F NMR Spectrum (CDCl_3_, 376 MHz) of **BtP24**

**Figure S130.** HRMS Spectrum of Target Compound **BtP24**


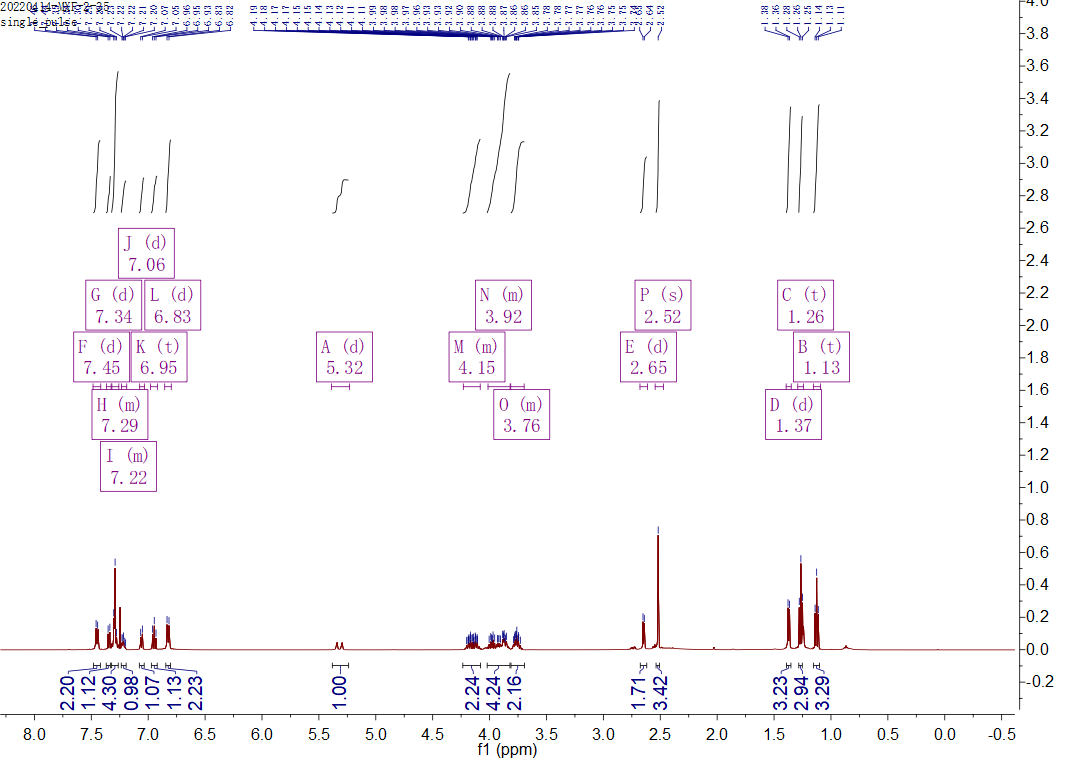
**Figure S131.** ^1^H NMR Spectrum (CDCl_3_, 400 MHz) of **BtP25**

**Figure S132.** ^13^C NMR Spectrum (CDCl_3_, 101 MHz) of **BtP25**

**Figure S133.** ^31^P NMR Spectrum (CDCl_3_, 162 MHz) of **BtP25**

**Figure S134.** HRMS Spectrum of Target Compound **BtP25**

**Figure S135.** ^1^H NMR Spectrum (CDCl_3_, 500 MHz) of **BtP26**

**Figure S136.** ^13^C NMR Spectrum (CDCl_3_, 126 MHz) of **BtP26**

**Figure S137.** ^31^P NMR Spectrum (CDCl_3_, 202 MHz) of **BtP26**

**Figure S138.** HRMS Spectrum of Target Compound **BtP26**

**Figure S139.** ^1^H NMR Spectrum (CDCl_3_, 400 MHz) of **BtP27**

**Figure S140.** ^13^C NMR Spectrum (CDCl_3_, 101 MHz) of **BtP27**

**Figure S141.** ^31^P NMR Spectrum (CDCl_3_, 162 MHz) of **BtP27**

**Figure S142.** ^19^F NMR Spectrum (CDCl_3_, 376 MHz) of **BtP27**

**Figure S143.** HRMS Spectrum of Target Compound **BtP27**

**Figure S144.** ^1^H NMR Spectrum (CDCl_3_, 400 MHz) of **BtP28**

**Figure S145.** ^13^C NMR Spectrum (CDCl_3_, 101 MHz) of **BtP28**

**Figure S146.** ^31^P NMR Spectrum (CDCl_3_, 162 MHz) of **BtP28**

**Figure S147.** ^19^F NMR Spectrum (CDCl_3_, 376 MHz) of **BtP28**

**Figure S148.** HRMS Spectrum of Target Compound **BtP28**

**Figure S149.** ^1^H NMR Spectrum (CDCl_3_, 400 MHz) of **BtP29**

**Figure S150.** ^13^C NMR Spectrum (CDCl_3_, 101 MHz) of **BtP29**

**Figure S151.** ^31^P NMR Spectrum (CDCl_3_, 162 MHz) of **BtP29**

**Figure S152.** HRMS Spectrum of Target Compound **BtP29**

**Figure S153.** ^1^H NMR Spectrum (CDCl_3_, 400 MHz) of **BtP30**

**Figure S154.** ^13^C NMR Spectrum (CDCl_3_, 101 MHz) of **BtP30**

**Figure S155.** ^31^P NMR Spectrum (CDCl_3_, 162 MHz) of **BtP30**

**Figure S156.** HRMS Spectrum of Target Compound **BtP30**

# 6. References

[1] W. Gu, Q. Li, Y. Li, *J. Hazard. Mater.* **2020**, *393*, 122339.

[2] L. Heimfarth, K. S. dos Anjos, Y. M. B. G. de Carvalho, B. L. dos Santos, M. R. Serafini, A. G. de Carvalho Neto, P. S. Nunes, J. I. A. Beserra Filho, S. P. da Silva, A. M. Ribeiro, D. P. Bezerra, R. N. Marreto, J. de Souza Siqueira Quintans, A. A. de Souza Araújo, H. D. Melo Coutinho, M. T. Scotti, L. Scotti， L. J. Quintans-Júnior, *Carbohydr. Polym.* **2020**, *244*, 116448.

[3] L. Kagami, A. Wilter, A. Diaz, W. Vranken, A. Elofsson, *Bioinformatics*. **2023**, *39*, 1367.

[4] F. Neese, *WIREs Comput. Mol. Sci.* **2017**, *8*, 1759.

[5] T. Lu, F. Chen, *J. Comput. Chem.* **2011**, *33*, 580.

[6] L. Martínez, R. Andrade, E. G. Birgin, J. M. Martínez, *J. Comput. Chem.* **2009**, *30*, 2157.

[7] S. Miyamoto, P. A. Kollman, *J. Comput. Chem.* **2004**, *13*, 952.

[8] T. Darden, D. York, L. Pedersen, *The Journal of Chemical Physics*. **1993**, *98*, 10089.

[9] M. Parrinello, A. Rahman, *J. Appl. Phys.* **1981**, *52*, 7182.
