# Supplementary material for: From De Novo Conceived Small Molecules to Multifunctional Supramolecular Nanoparticles: Dual Biofilm and T3SS Intervention, Enhanced Foliar Affinity, and Effective Rice Disease Control
Source: Adv Sci (Weinh). 2025 Mar 27;12(20):2410878. doi: 10.1002/advs.202410878 (PMC12120793; doi:10.1002/advs.202410878)
Supplement: Supplementary file 4 — Supporting Information [file ADVS-12-2410878-s004.pdf]

ADVANCED SCIENCE
Open Access

Supporting Information

for *Adv. Sci.*, DOI 10.1002/advs.202410878

From *De Novo* Conceived Small Molecules to Multifunctional Supramolecular Nanoparticles: Dual Biofilm and T3SS Intervention, Enhanced Foliar Affinity, and Effective Rice Disease Control

Xianfu Mu, Kongjun Liu, Jinghan Yang, Juan Liu, Fengpei Du, Gefei Hao and Peiyi Wang**

Predictive Data Tables of BtP27 using ProTox-3.0

ProTox-3.0 - Prediction of TOXicity of chemicals

Classification	Target	Shorthand	Prediction	Probability
Organ toxicity	Hepatotoxicity	dili	Inactive	0.60
Organ toxicity	Neurotoxicity	neuro	Inactive	0.54
Organ toxicity	Nephrotoxicity	nephro	Active	0.58
Organ toxicity	Respiratory toxicity	respi	Active	0.79
Organ toxicity	Cardiotoxicity	cardio	Inactive	0.6
Toxicity end points	Carcinogenicity	carcino	Inactive	0.64
Toxicity end points	Immunotoxicity	immuno	Active	0.68
Toxicity end points	Mutagenicity	mutagen	Inactive	0.57
Toxicity end points	Cytotoxicity	cyto	Inactive	0.59
Toxicity end points	BBB-barrier	bbb	Active	0.52
Toxicity end points	Ecotoxicity	eco	Inactive	0.50
Toxicity end points	Clinical toxicity	clinical	Active	0.59
Toxicity end points	Nutritional toxicity	nutri	Active	0.65
Tox21-Nuclear receptor signalling pathways	Aryl hydrocarbon Receptor (AhR)	nr_ahr	Inactive	0.76
Tox21-Nuclear receptor signalling pathways	Androgen Receptor (AR)	nr_ar	Inactive	0.95
Tox21-Nuclear receptor signalling pathways	Androgen Receptor Ligand Binding Domain (AR-LBD)	nr_ar_lbd	Inactive	0.95
Tox21-Nuclear receptor signalling pathways	Aromatase	nr_aromatase	Inactive	0.87
Tox21-Nuclear receptor signalling pathways	Estrogen Receptor Alpha (ER)	nr_er	Inactive	0.77
Tox21-Nuclear receptor signalling pathways	Estrogen Receptor Ligand Binding Domain (ER-LBD)	nr_er_lbd	Inactive	0.93
Tox21-Nuclear receptor signalling pathways	Peroxisome Proliferator Activated Receptor Gamma (PPAR-Gamma)	nr_ppar_gamma	Inactive	0.94
Tox21-Stress response pathways	Nuclear factor (erythroid-derived 2)-like 2/ antioxidant responsive element (nrf2/ARE)	sr_are	Inactive	0.91
Tox21-Stress response pathways	Heat shock factor response element (HSE)	sr_hse	Inactive	0.91
Tox21-Stress response pathways	Mitochondrial Membrane Potential (MMP)	sr_mmp	Inactive	0.79
Tox21-Stress response pathways	Phosphoprotein (Tumor Supressor) p53	sr_p53	Inactive	0.86
Tox21-Stress response pathways	ATPase family AAA domain-containing protein 5 (ATAD5)	sr_atad5	Inactive	0.88
Molecular Initiating Events	Thyroid hormone receptor alpha (THRα)	mie_thr_alpha	Inactive	0.79
Molecular Initiating Events	Thyroid hormone receptor beta (THRβ)	mie_thr_beta	Inactive	0.71
Molecular Initiating Events	Transtyretrin (TTR)	mie_ttr	Inactive	0.65
Molecular Initiating Events	Ryanodine receptor (RYR)	mie_ryr	Inactive	0.71
Molecular Initiating Events	GABA receptor (GABAR)	mie_gabar	Inactive	0.79
Molecular Initiating Events	Glutamate N-methyl-D-aspartate receptor (NMDAR)	mie_nmdar	Inactive	0.91
Molecular Initiating	alpha-amino-3-hydroxy-5-methyl-4-	mie_ampar	Inactive	0.96

Classification	Target	Shorthand	Prediction	Probability
Events	isoxazolepropionate receptor (AMPAR)			
Molecular Initiating Events	Kainate receptor (KAR)	mie_kar	Inactive	0.98
Molecular Initiating Events	Achetylcholinesterase (AChE)	mie_ache	Inactive	0.74
Molecular Initiating Events	Constitutive androstane receptor (CAR)	mie_car	Inactive	0.99
Molecular Initiating Events	Pregnane X receptor (PXR)	mie_pxr	Inactive	0.65
Molecular Initiating Events	NADH-quinone oxidoreductase (NADHOX)	mie_nadhox	Inactive	0.83
Molecular Initiating Events	Voltage gated sodium channel (VGSC)	mie_vgsc	Inactive	0.52
Molecular Initiating Events	Na+/I- symporter (NIS)	mie_nis	Inactive	0.87
Metabolism	Cytochrome CYP1A2	CYP1A2	Inactive	0.79
Metabolism	Cytochrome CYP2C19	CYP2C19	Inactive	0.72
Metabolism	Cytochrome CYP2C9	CYP2C9	Inactive	0.64
Metabolism	Cytochrome CYP2D6	CYP2D6	Inactive	0.52
Metabolism	Cytochrome CYP3A4	CYP3A4	Inactive	0.69
Metabolism	Cytochrome CYP2E1	CYP2E1	Inactive	0.94
